# The past and future impact of climate change on childhood malaria in Africa

**DOI:** 10.1038/s41586-026-10840-w

**Published:** 2026-07-29

**Authors:** Colin J. Carlson, Tamma Carleton, Romaric C. Odoulami, Cullen D. Molitor, Christopher H. Trisos

**Affiliations:** 1 Department of Epidemiology of Microbial Diseases, Yale University School of Public Health, New Haven, CT USA; 2 Public Health Modeling Unit, Yale University School of Public Health, New Haven, CT USA; 3 Yale Center on Climate Change and Health, Yale University School of Public Health, New Haven, CT USA; 4 Department of Agricultural and Resource Economics, University of California, Berkeley, Berkeley, CA USA; 5 National Bureau of Economic Research, Cambridge, MA USA; 6 Center for Effective Global Action, University of California, Berkeley, Berkeley, CA USA; 7 African Climate and Development Initiative, University of Cape Town, Cape Town, South Africa

**Keywords:** Environmental health, Attribution, Projection and prediction, Malaria

## Abstract

Despite recent advances in climate change attribution, many health impacts remain unmeasured^1^. Here we leverage over a century of clinical data^2^ to investigate whether human-caused climate change has increased the burden of childhood malaria across sub-Saharan Africa. We find a robust effect of temperature and extreme precipitation on prevalence, consistent with previous findings at local scales and in laboratory experiments. We estimate that rising temperatures have probably increased malaria in East and southern Africa, but averted a comparable number of cases in West Africa, with a net impact of 1 excess case per 1,000 children (95% confidence interval (CI) −4 to 6) across the continent. Over the coming century, we project that climate change could marginally accelerate the elimination of malaria in West and central Africa, where the present-day burden is highest; across the continent, this could avert 1 (low greenhouse gas emissions: 95% CI −2 to 6) to 20 (high greenhouse gas emissions: 95% CI 0–52) cases per 1,000 children by the end of the century. However, reducing future global warming from 3 °C to under 2 °C could prevent an average of 5 excess cases per 1,000 in high-elevation (more than 1 km) East Africa and in southern Africa (95% CIs −3 to 13 and −4 to 14, respectively) by 2100. Our study resolves a decades-old debate about one of the first suspected health impacts of climate change, providing a template for future work measuring its true global burden.

## Abstract (web summary)

Climate and econometric modelling reveal that human-induced climate change is shifting the burden of childhood malaria in Africa.

## Main

Despite progress towards global eradication, malaria remains the single deadliest climate-sensitive infectious disease. Malaria transmission is highly responsive to temperature, driven by both the life cycle of the ectothermic mosquito vectors (*Anopheles* spp.) and the thermal sensitivity of the parasites (*Plasmodium* spp.) themselves^3,4^. In laboratory conditions, *Plasmodium falciparum* transmission by *Anopheles gambiae* peaks around 25 °C and becomes negligible below approximately 16 °C or above approximately 34 °C (refs. ^3–5^). Given these biological constraints, climate change has become a major concern for populations potentially at risk of malaria in southern and high-elevation East Africa, where temperatures may no longer be prohibitive to malaria transmission^6,7^. Conversely, in West and central Africa—where the burden of malaria is highest—many studies have suggested that climate change will reduce or eventually preclude transmission^6,8,9^.

These risks were among the first proposed health impacts of climate change^10,11^, but have been surprisingly contentious, and even described as ‘hot air’^12^ and ‘dangerous pseudoscience’^13^. At the turn of the century, many malaria experts argued that observed warming trends were incompatible with long-term reductions in malaria prevalence across Africa, and warned that other factors such as drug resistance and funding instability posed a more serious threat to malaria eradication^12,14,15^. Malaria resurgence in the East African highlands became a particular point of contention, with over a dozen studies arguing for^16–20^ or against^21–27^ climate change as a substantial driver. Today, malaria experts generally agree that climate change has contributed to elevational shifts in malaria epidemics^28,29^ and the geographical ranges of mosquito vectors^30^. However, the cumulative effect of climate change on the burden of malaria is still an open question: recently, Snow et al.^2^ examined the past century of continent-wide changes in malaria prevalence and concluded that observed trends could not be neatly explained by climate change, but did so based only on visual correspondence between moving averages of rainfall, minimum temperature and malaria prevalence over the entire continent.

In this study, we revisited these debates by applying state-of-the-art methods from detection and attribution, an area of climate science that quantifies the historical and real-time climate impacts of anthropogenic greenhouse gas emissions^31^. These methods underpin the scientific consensus on human-caused climate change, and are regularly used to identify the role of climate change in the intensity, frequency and distribution of specific extreme events (for example, heatwaves, heavy precipitation and droughts)^32–34^. However, attribution remains challenging for the downstream effects of anthropogenic climate change on people and ecosystems, and methodological frameworks for impact attribution are still comparatively underdeveloped^35,36^. Applications to infectious disease dynamics are especially challenging, as relationships between climate and disease transmission are often complex, nonlinear and confounded by human intervention, and few epidemiological datasets exist with sufficient spatial and temporal scope to resolve these relationships. As a result, hundreds of studies have tested for correlations between climate and observed changes in disease incidence or prevalence, but very few have shown that these changes are causally attributable to anthropogenic climate change^1^.

**Fig. 1:**
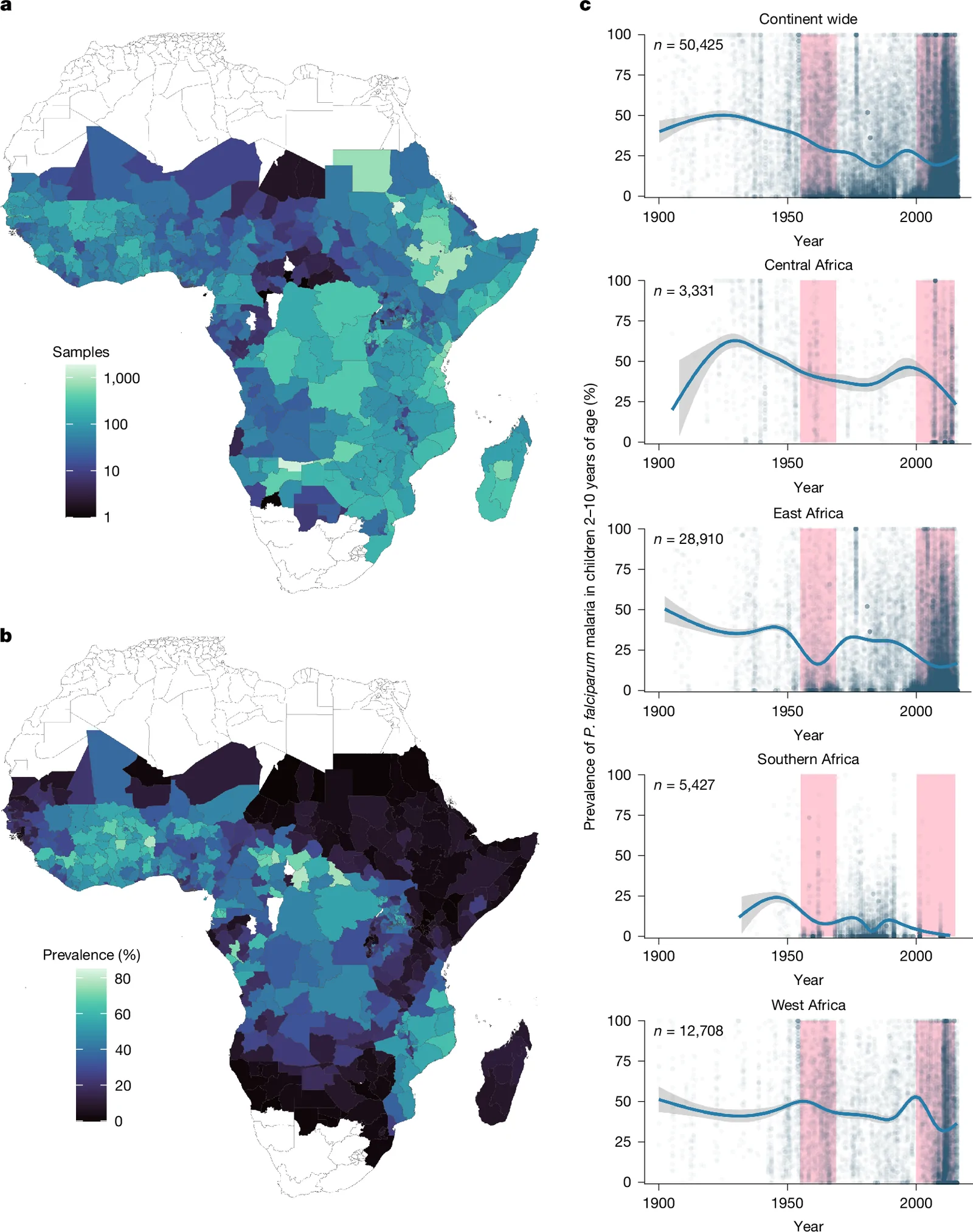
Malaria prevalence observations from 1900 to 2016. **a**, The total number of malaria prevalence surveys in children 2–10 years of age in the twentieth and early twenty-first century, as measured by Snow et al.^2^ and aggregated to the first administrative unit. **b**, Mean reported prevalence of childhood malaria over the entire sample (1900–2016, with temporal coverage varying across space). **c**, Observed trends in malaria prevalence, broken down by Global Burden of Disease study regions (see main text): each point is a single survey in the original dataset (and the sample size, *n*, is the total number of surveys) and generalized additive models were used to construct estimated trend lines (shown in solid blue, with the grey shading showing the 95% CI of the model). The pink vertical bars indicate notable periods of successful malaria prevention intervention: the Global Malaria Eradication Programme (1955–1969) and the modern period, including the Roll Back Malaria programs and Global Technical Strategy (2000–2015).

**Fig. 2:**
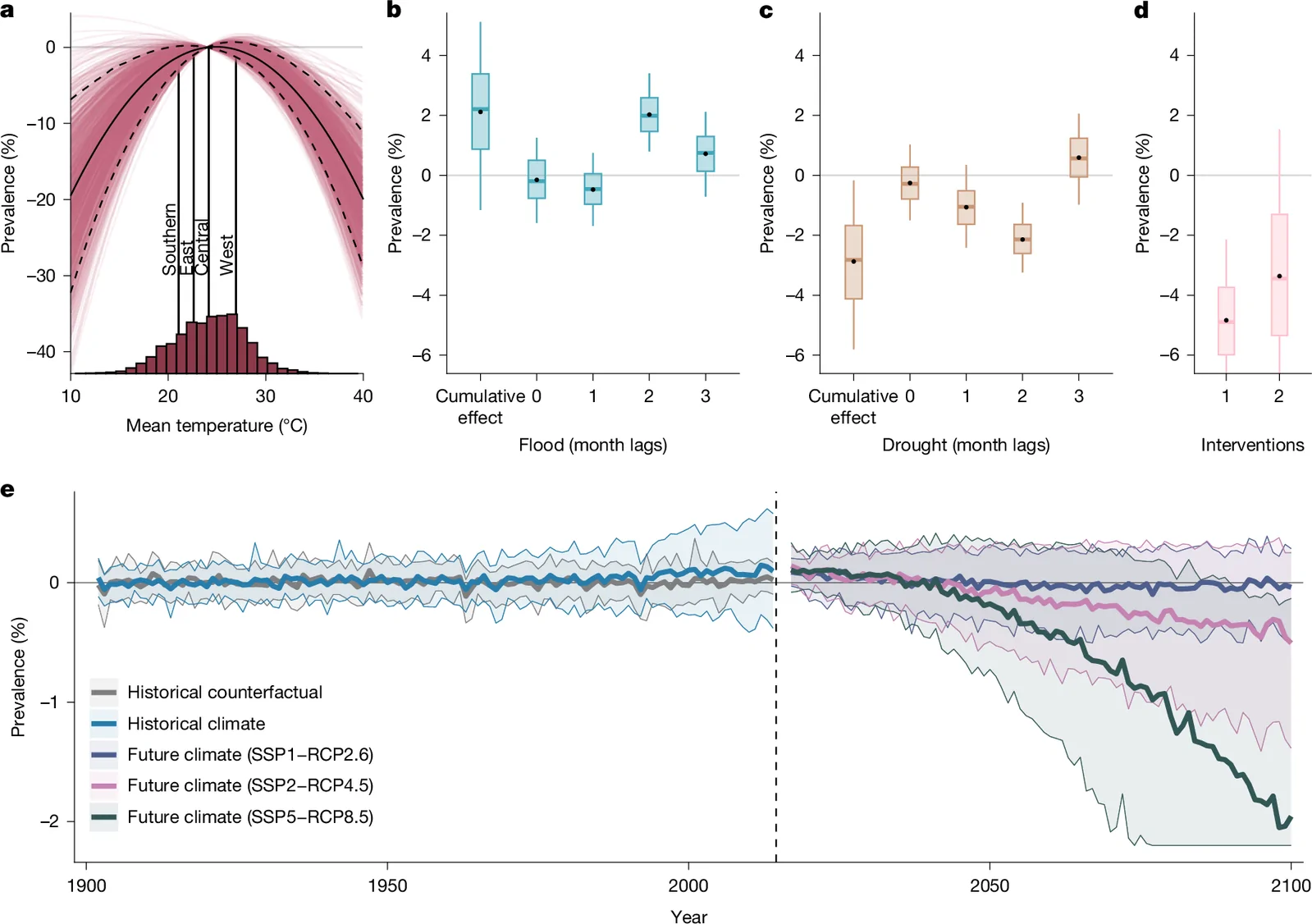
Empirical estimates of prevalence–climate relationships and predictions of climate change impacts from 1901 to 2100. **a**, The estimated relationship between temperature and prevalence (point estimate is the solid black line; 90% CI is the dashed black line; and 1,000 random draws from the variance–covariance estimate are in red). The histogram shows the distribution of monthly temperatures across the estimation sample, with regional median temperatures shown as vertical lines. **b**,**c**, The effects of extreme precipitation events (flood (**b**) and drought (**c**)) on prevalence in the month the events occur (0 lag) and after time has passed (1-month, 2-month and 3-month lags), as well as the cumulative impact across the first 4 months. **d**, The effects on prevalence of two key intervention periods (1 refers to 1955–1969; and 2 refers to 2000–2015) during which large-scale malaria prevention programmes were implemented across the sub-continent. In panels **b**–**d**, point estimates (black) are accompanied by resampled draws shown as boxplots, where the box spans the 25–75th percentiles, the centre line indicates the median, and the whiskers extend to the 5th and 95th percentiles (blue, brown and pink). Grey horizontal lines show the zero isocline (no estimated effect on prevalence). **e**, Predicted change in prevalence attributable to anthropogenic climate change in the recent past (real historical climate is given in blue; counterfactual without anthropogenic warming is in grey) and in the future for low (SSP1–RCP2.6 in dark blue), intermediate (SSP2–RCP4.5 in pink) and high (SSP5–RCP8.5 in green) emission scenarios. The thick lines are the mean estimates accounting for both statistical and climate uncertainty; the shading indicates the 5th and 95th percentiles and is truncated at the lower axis limits for visualization purposes only (the full interval is shown in Extended Data Fig. 9). Historical estimates are shown relative to an average baseline across 1901–1930. Future estimates are shown relative to a baseline across 2015–2020, added to the end-of-historical baseline (2010–2014). The dashed grey line indicates 2014, the end of the historical baseline. Years with incomplete predictions due to lag effects (1901 and 2015) are not displayed.

**Fig. 3:**
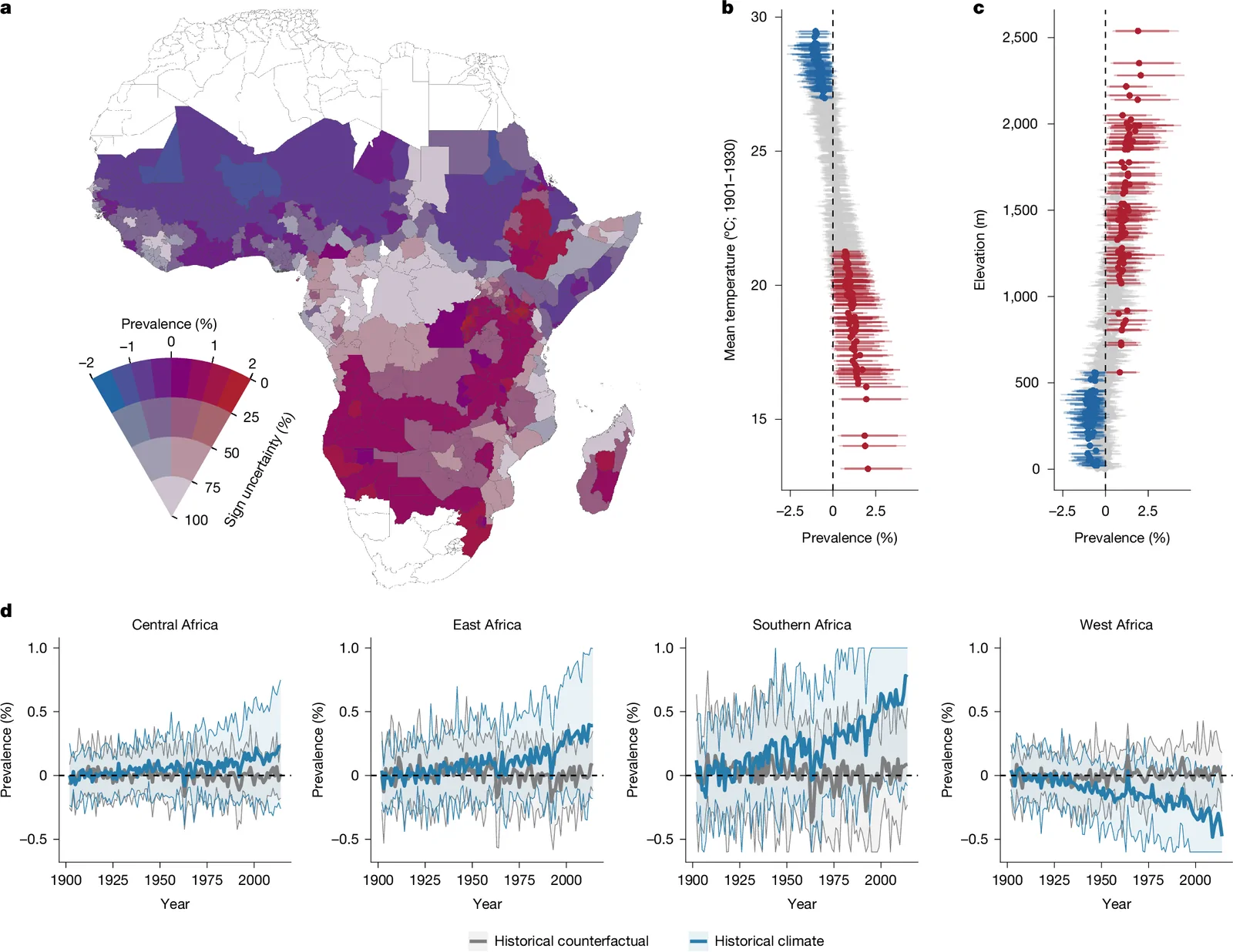
Historical changes in malaria prevalence attributable to anthropogenic climate change from 1901 to 2014. **a**, Estimated change in prevalence attributable to anthropogenic climate change in each administrative unit, based on the difference between the historical climate in 2010–2014 and a counterfactual scenario for the same period simulated without anthropogenic warming. Sign uncertainty is the percentage of the 10,000 simulations that estimate an increase (for positive point estimates) or decrease (for negative point estimates) in prevalence due to anthropogenic climate change. An uncertainty of 0% implies that all simulations predict a positive or negative trend, whereas an uncertainty close to 100% indicates a near-even split of simulations showing an increase or decrease in prevalence. **b**, Estimated change in prevalence attributable to anthropogenic climate change (difference between factual and counterfactual scenario in 2010–2014) in each administrative polygon, compared with the baseline mean temperature at the start of the twentieth century (averaged over 1901–1930); the error bars indicate both 90% (thicker lines) and 95% (thinner lines) CIs. Points and lines are coloured based on the 90% CI: blue for negative effects, red for positive effects and grey for non-significant effects. **c**, Estimated change in prevalence attributable to anthropogenic climate change in 2010–2014 in each administrative polygon, compared with average elevation; the error bars indicate both 90% and 95% CIs, coloured as in panel **b**. **d**, Predicted historical changes in prevalence by year, broken down by region. As in Fig. 2, predictions based on true historical climate (blue) are compared with counterfactual predictions without anthropogenic warming (grey), relative to a 1901–1930 baseline. The thick lines are the mean estimate across all 10,000 simulations; for visualization purposes, the shading indicates the 90% CI and is truncated at the upper and lower axis limits. Plots begin in 1902 with the first full year of predictions (due to lag effects). Black dashed lines in **b**–**d** show the zero isocline (no change in prevalence).

**Fig. 4:**
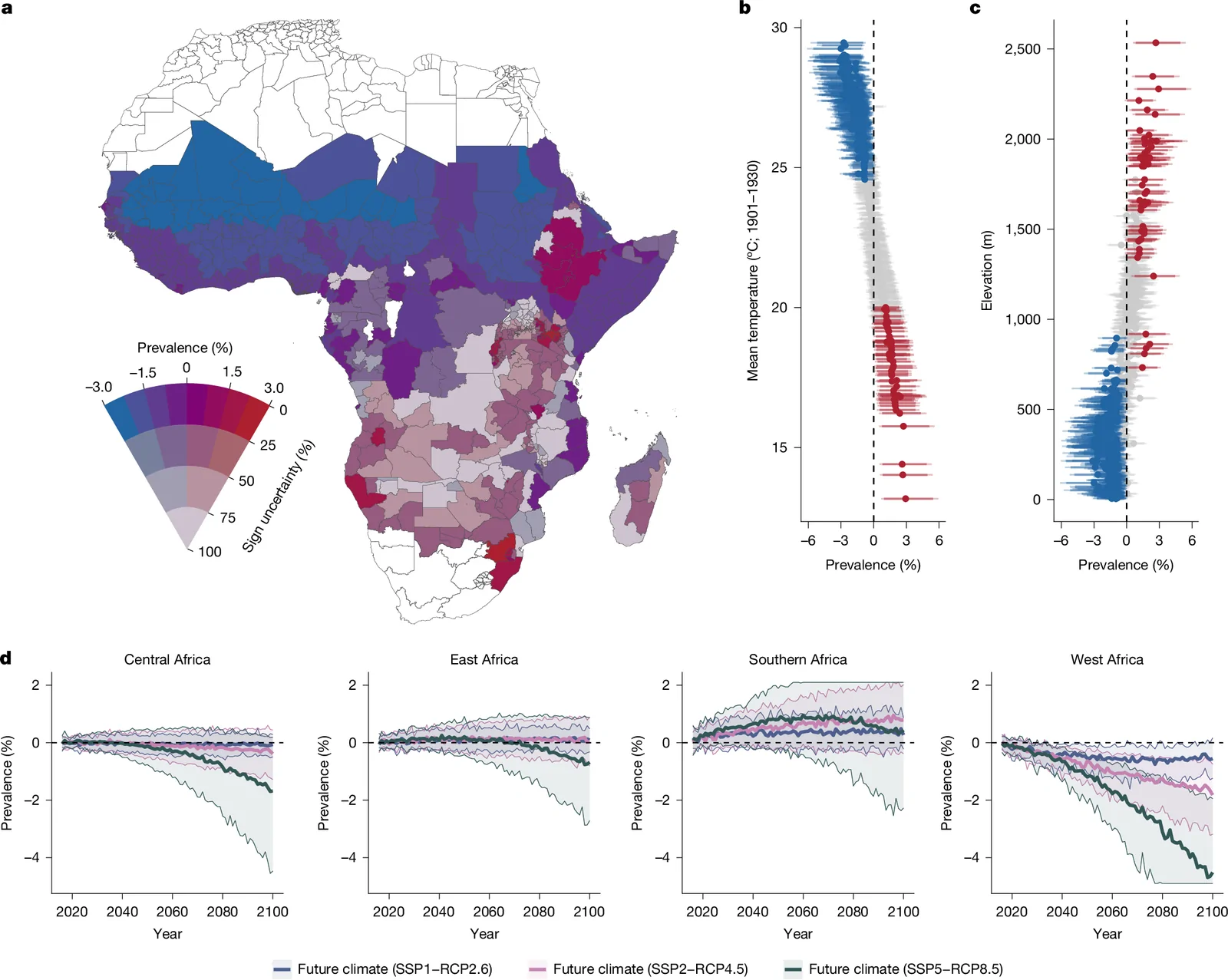
Projected future changes in malaria prevalence driven by climate change from 2015 to 2100. **a**, Projected climate-driven changes in prevalence by the end of the century (2096–2100), compared with the present day (2015–2020), for an intermediate emissions scenario (SSP2–RCP4.5). Sign uncertainty is the percentage of the 10,000 simulations that estimate an increase (for positive point estimates) or decrease (for negative point estimates) in prevalence due to future climate change. An uncertainty of 0% implies that all simulations predict a positive or negative trend, whereas an uncertainty close to 100% indicates a near-even split of simulations showing an increase or decrease in prevalence. **b**, Projected change in prevalence due to climate change by the end of the century (2096–2100) in each administrative polygon, estimated for SSP2–RCP4.5, compared with the baseline mean temperature at the start of the twentieth century; the error bars indicate both 90% (thicker lines) and 95% (thinner lines) CIs. The points and lines are coloured based on the 90% CI: blue for negative effects, red for positive effects and grey for non-significant effects. **c**, Projected change in prevalence due to climate change by the end of the century (2096–2100) in each administrative polygon, estimated for SSP2–RCP4.5, compared with average elevation; the error bars indicate both 90% and 95% CIs, coloured as in panel **b**. **d**, Projected changes in prevalence by year across all scenarios by region. Projections are given relative to the mean from 2015 to 2020, and as in Fig. 2, and the line colour indicates scenario (SSP1–RCP2.6 in blue, SSP2–RCP4.5 in pink and SSP5–RCP8.5 in green). The thick lines are the mean estimate across all 10,000 simulations; for visualization purposes, the shading indicates the 90% CI, and is truncated at the upper and lower axis limits. Plots begin in 2016 with the first full year of predictions (due to lag effects). Black dashed lines in **b**–**d** show the zero isocline (no change in prevalence).

Here we drew on frameworks from climate science, econometrics and epidemiology to conduct an end-to-end impact attribution study (per ref. ^36^), measuring the direct effect of anthropogenic climate change on long-term trends in the burden of an infectious disease. We applied this framework to estimates of *P.* *falciparum* malaria prevalence in children 2–10 years of age in sub-Saharan Africa (*Pf*PR_2−10_), which experiences roughly 95% of the global burden of malaria (with 80% of deaths in children under 5 years of age)^37^. We analysed a recently published dataset with unparalleled resolution and scope (Fig. 1), consisting of 50,425 surveys spanning more than a century (1900–2016)^2^, which we aggregate to 9,875 monthly average values at the first administrative (state or province) level. These data capture a snapshot of population-wide prevalence at a moment in time (that is, cases of active malaria infection per child; versus, for example, incidence rate: new cases per child per year). Leveraging climate econometric methods^38–40^, we developed a panel regression model that isolates the role of temperature and extreme precipitation from other confounding factors that also shape malaria endemicity (Fig. 2; see Methods for details). Nonparametric controls in the model (that is, fixed effects) account for regional differences in seasonality, time periods with concerted elimination efforts and other spatiotemporal variation not explained by identifiable factors, such as socioeconomic or ecological differences between populations. We applied this econometric model to make predictions based on ten sets of paired historical climate simulations with and without anthropogenic climate forcing, and estimate the effect of anthropogenic climate change on malaria prevalence from 1901 to 2014, accounting for both statistical and climatological uncertainty (Fig. 3). Finally, we projected how future climate change could further alter malaria prevalence between 2015 and 2100, based on three future climate change scenarios for low (shared socioeconomic pathway 1–representative concentration pathway 2.6 (SSP1–RCP2.6)), intermediate (SSP2–RCP4.5) and high (SSP5–RCP8.5) future greenhouse gas concentrations (Fig. 4).

### A robust signal of climate sensitivity

**Extended Data Fig. 1:**
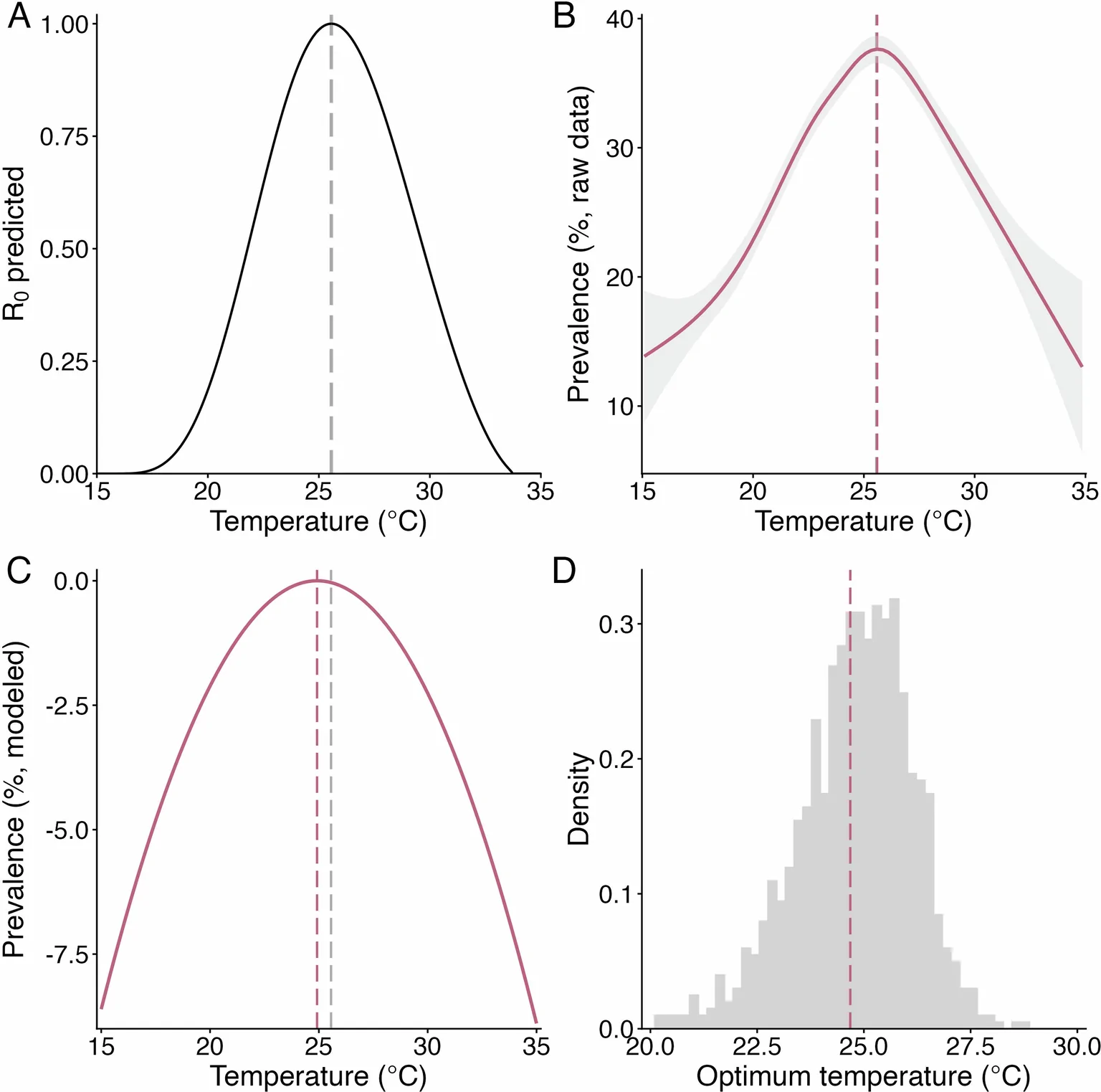
Malaria prevalence follows biological expectations. (A) The theoretical expectation for *R*_0_(*T*), the scaled partial response of the basic reproduction number to temperature, estimated based on laboratory experiments (black line)^3^. Transmission peaks around an estimated optimum of 25.6 °C (grey dashed line). (B) Observed malaria prevalence data from Snow et al.^2^ matched to monthly temperature from Climatic Research Unit (CRU) weather station data, summarized and smoothed using a generalized additive model. The observed optimum temperature (red dashed line) closely matches expectations based on laboratory experiments (grey dashed line). (C) Main panel regression estimate for prevalence response to temperature (also shown in Fig. 2a). The modeled optimum temperature (red dashed line) is slightly lower than in laboratory experiments (grey dashed line). (D) Histogram of optimum temperatures derived from 1,000 estimates resampled from the variance-covariance matrix of the panel regression model shown in panel (C). The mean optimum temperature across all samples (red dashed line) is identical to the optimum shown in panel C.

Over the past century, the prevalence of childhood malaria has exhibited a strong concave relationship with temperature (Fig. 2a). Closely aligning with theoretical expectations that *P. falciparum* transmission by *A. gambiae* mosquitoes should peak around 25.6 °C (ref. ^3^), observed values of *Pf*PR_2−10_ in our dataset peak around a monthly mean temperature of 25.8 °C (Extended Data Fig. 1). On the basis of these biological expectations, we estimated the effect of temperature as a quadratic response in a panel regression model, and found that prevalence peaks at 24.9 °C (95% CI 21.0–27.1 °C). These results confirm that laboratory-based studies approximate malaria epidemiology in real populations quite well, and that temperature has a substantial role in transmission dynamics: a 10 °C increase or decrease from the optimal temperature lowers prevalence by approximately 8 percentage points (p.p.).

The relationship between precipitation and malaria prevalence is more complex, and probably less consequential for historical trends (Fig. 2b,c). Contemporaneous monthly precipitation exhibits a nonlinear, but highly uncertain, relationship to prevalence (Supplementary Fig. 1). To parsimoniously capture nonlinear effects and disentangle divergent impacts of low and high precipitation, we defined precipitation shocks with two binary indicator variables, equal to one when monthly precipitation falls below the 10th percentile (we labelled this a ‘drought shock’) or above the 90th percentile (‘flood shock’) of monthly precipitation calculated for each subnational unit. Although drought and flood events are complex phenomena, which develop from the combination of multiple factors (for example, soil conditions and topography) in addition to rainfall over varying timescales, we used this drought–flood terminology as shorthand to indicate extremely low or high precipitation months. Most effects are statistically insignificant, but we found that drought shocks tend to decrease malaria prevalence 1–2 months later, whereas conversely, flood shocks have a positive effect on prevalence 2–3 months later. These effects and their timing are broadly consistent with expectations about how precipitation mediates the availability of a mosquito breeding habitat: dry out kills larvae and eggs^41^, whereas inundation creates a new breeding habitat^42^. Sensitivity analyses were also weakly suggestive of another established mechanism^43^, in which floods may wash away eggs and larvae, reducing transmission in the shorter term (Supplementary Figs. 1 and 2). Overall, extreme precipitation has a measurable effect on malaria prevalence, but may be less important than temperature; however, given the sparsity of weather station data^44^ and the uncertainty of precipitation reconstructions^45^, it is also possible that our analysis unavoidably underestimates the effect of precipitation due to measurement error.

**Extended Data Fig. 2:**
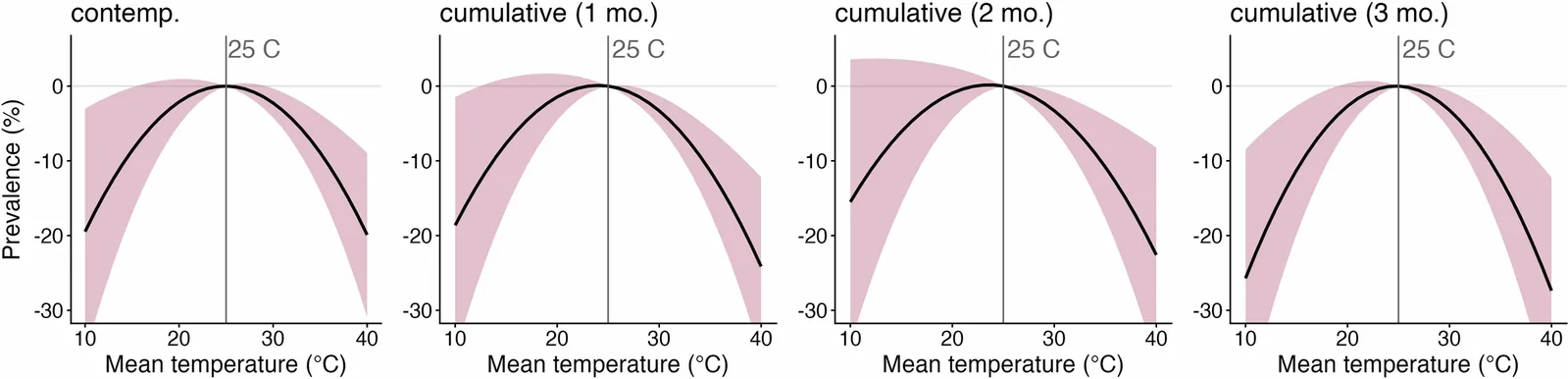
Cumulative effect of contemporaneous and lagged temperature on *Pf*PR_2−10_. All panels show the estimated relationship between malaria prevalence for children aged 2-10 and monthly average temperature and are plotted relative to a monthly average temperature of 25 °C. The first panel shows the effect of monthly average temperature on the same month’s average prevalence (this is the main estimate used throughout the main text). The second panel shows the cumulative effect of contemporaneous temperature and the prior month’s temperature on prevalence, while the last two columns show analogous results for two and three months of lags, respectively. In all specifications, three months of lagged precipitation extremes are included, as well as all other controls shown in equation (4). All standard errors are clustered at the country-by-5-year group level.

**Extended Data Fig. 3:**
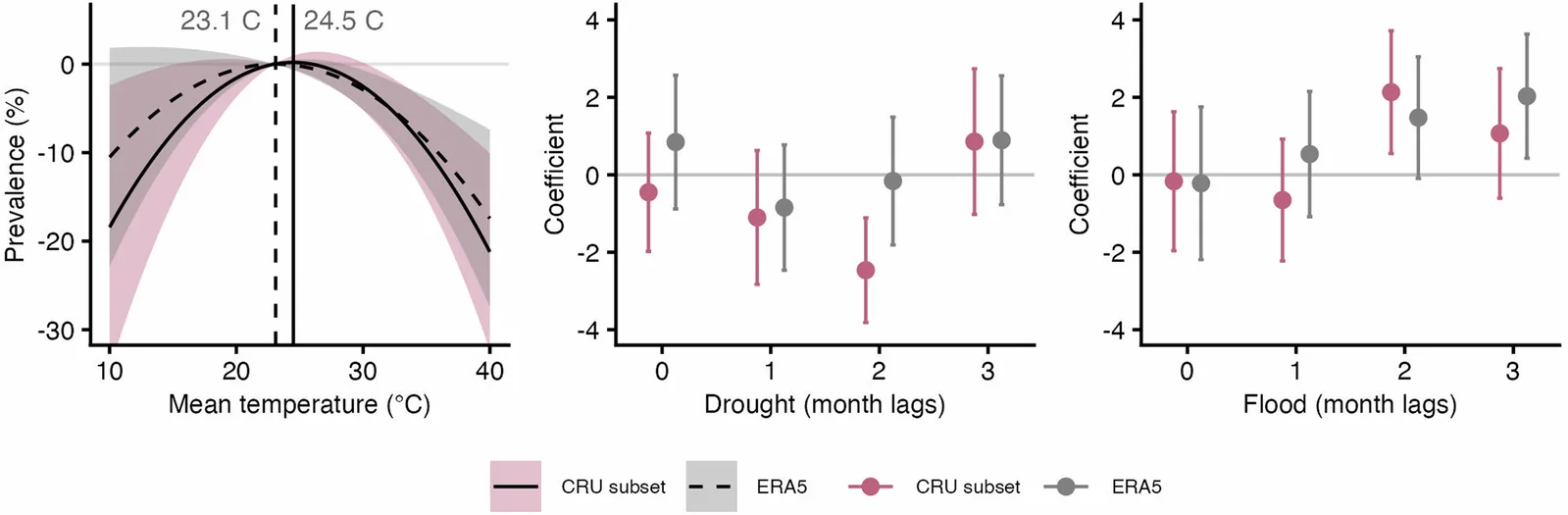
Empirical estimates of PfPR_2-10_-climate relationships using alternative climate input datasets. Panels show the estimated relationship between malaria prevalence for children aged 2-10 and monthly average temperature (left), drought (middle), and flood (right). Regressions are estimated separately for our main model specification using Climatic Research Unit (CRU) climate data or an alternative climate dataset, the European Centre for Medium-Range Weather Forecasts Reanalysis v5 (ERA5). Both regressions are limited to the years 1940-2016, the time overlap of the two datasets and available malaria prevalence data. (Left) The relationship between prevalence and mean monthly temperature for a CRU-based model (black solid line and pink shading) and for an ERA5-based model (dashed line and grey shading), with shading indicating 95% confidence intervals. (Middle, right) Estimated coefficients and 95% confidence intervals for the effects of drought (middle) and flood (right) events across contemporaneous and lagged months for both model specifications. Positive coefficients indicate higher malaria prevalence associated with extreme climate events. All standard errors are clustered at the country-by-5-year group level.

**Extended Data Fig. 4:**
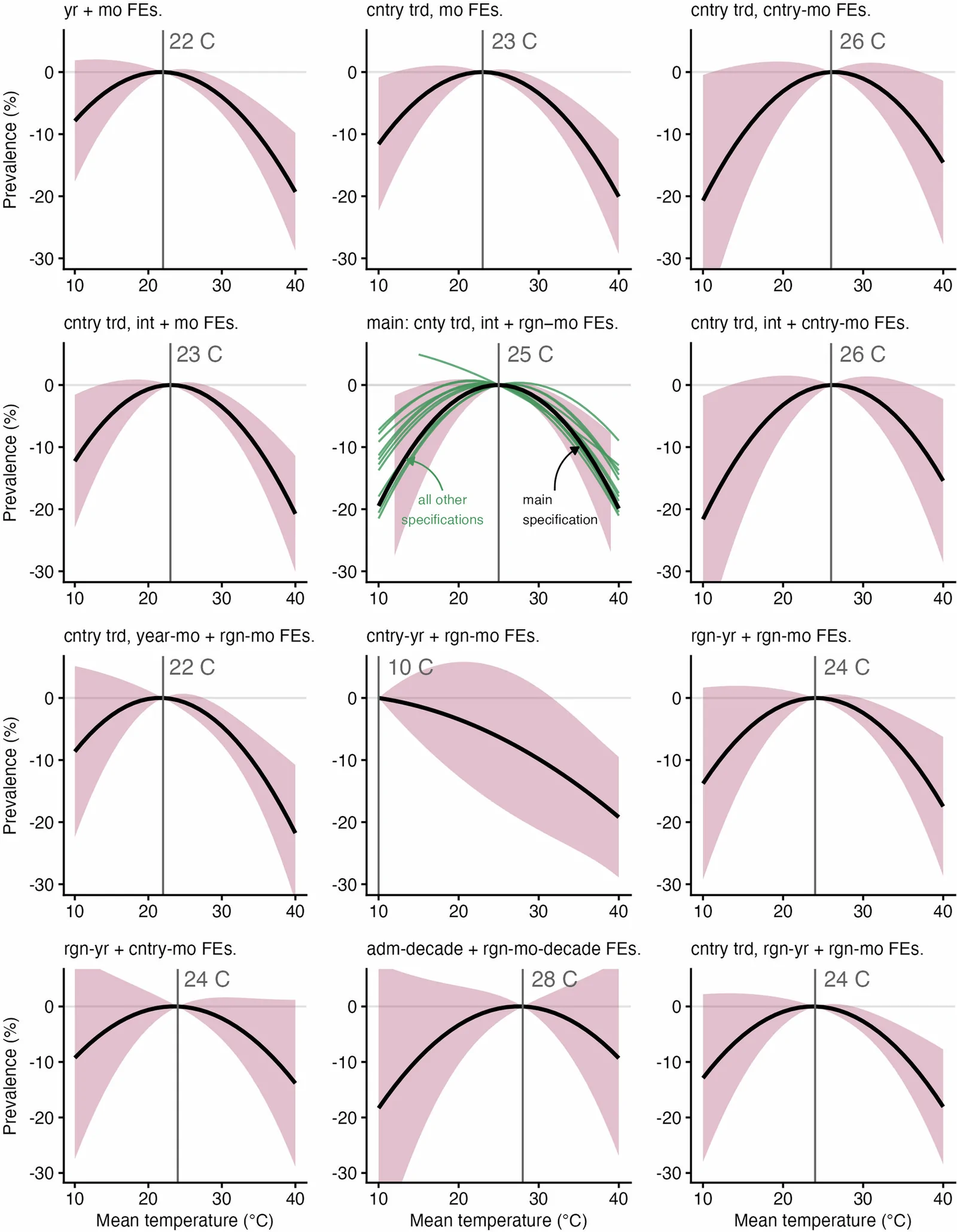
Sensitivity of the *Pf*PR_2−10_-temperature relationship to alternative spatiotemporal controls. All panels show the estimated relationship between malaria prevalence for children aged 2-10 and monthly average temperature and all include fixed effects (i.e., dummy variables) at the scale of the first administrative unit (i.e., ADM1). All temperature responses are plotted relative to the model-specific temperature at which prevalence is maximized; this peak temperature is indicated in grey text and with a vertical grey line in each panel. From top-left to bottom-right, model controls are: year and month fixed effects (“FEs”); country-specific quadratic time trends (“country trends”) and month FEs; country trends and country-by-month FEs; country trends and intervention period and month FEs; country trends and intervention period and region-by-month FEs; country trends and intervention period and country-by-month FEs; country trends and year-by-month and region-by-month FEs; country-by-year and region-by-month FEs; region-by-year and region-by-month FEs; region-by-year and country-by-month FEs; ADM1-by-decade and region-by-month-by-decade FEs; and region-by-year and region-by-month FEs with country-specific linear time trends. The preferred specification used throughout the main text is the fifth panel, where the main specification is shown in black (with pink shaded 95% confidence intervals) and all alternative specifications from other panels are overlaid in green. “Region” refers to the Global Burden of Disease definitions of West, Southern, Central, and East Africa. All standard errors are clustered at the country-by-5-year level.

**Extended Data Fig. 5:**
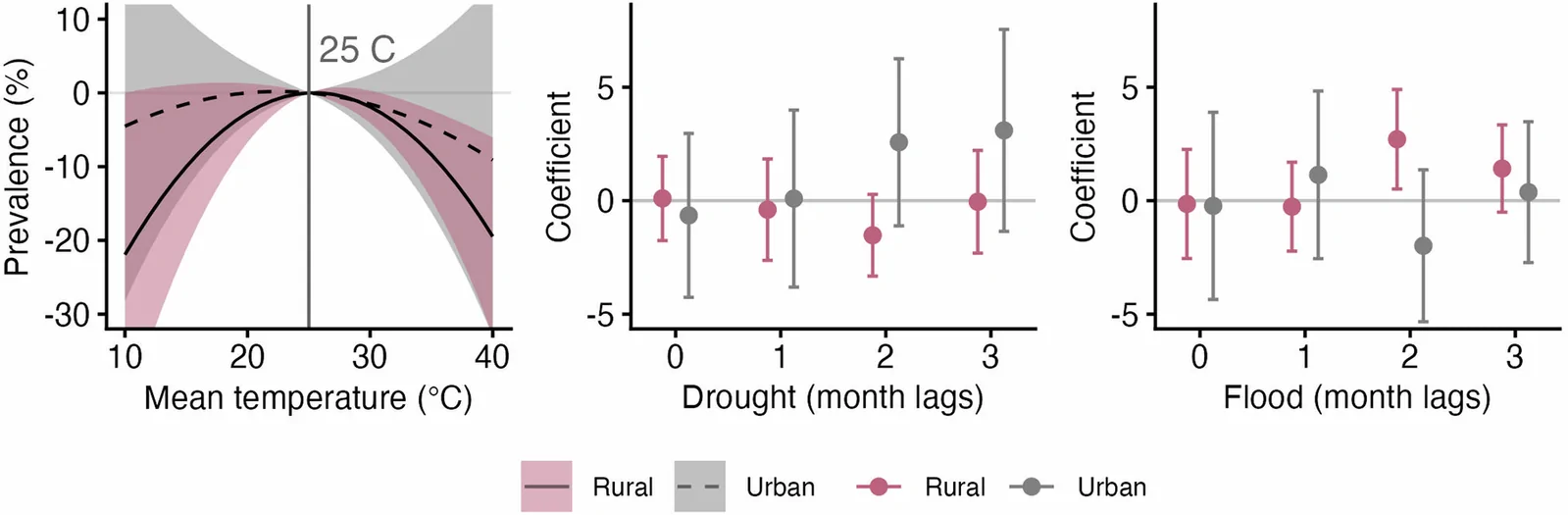
Heterogeneous effects of temperature and extreme precipitation on malaria prevalence by urban and rural status. All panels show the estimated relationship between malaria prevalence for children aged 2-10 estimated separately for rural (pink) and urban (grey) areas. (Left) The relationship between prevalence and mean monthly temperature with shading indicating 95% confidence intervals. (Middle, Right) Estimated coefficients and 95% confidence intervals for the effects of drought (middle) and flood (right) events across contemporaneous and lagged months. Positive coefficients indicate higher malaria prevalence associated with extreme climate events. Model estimates include fixed effects for administrative units and regional seasonality, with standard errors clustered at the country-by-5-year group level. Urban-rural classifications are based on the Global Human Settlement Layer Urban Centre Database^93^.

Additional sensitivity analyses reinforce that these prevalence–climate relationships are both statistically robust and biologically consistent. Key findings are stable through time (Supplementary Fig. 3) and are generally insensitive to alternative model specifications, such as the inclusion of lagged effects of temperature (Extended Data Fig. 2); higher-order polynomial effects of temperature (Supplementary Fig. 4); alternative definitions of drought and flood shocks (Supplementary Figs. 2, 5 and 6); alternative climate data (Extended Data Fig. 3 and Supplementary Table 1); controlling for the diagnostic test type (Supplementary Table 2); alternative methods of capturing uncertainty (Supplementary Table 3 and Supplementary Fig. 7); and alternative spatiotemporal controls, which account differently for variation over space (at region, country and state levels), time (including yearly and monthly variation) and interactions among space and time (Extended Data Table 1 and Extended Data Fig. 4). Results are robust to dropping individual countries, years or months from the sample (Supplementary Fig. 8) and to estimating a grid-level regression that does not aggregate prevalence or weather across space (Supplementary Fig. 9). Temperature and flood effects are strongest in rural areas (Extended Data Fig. 5), consistent with negative direct relationships between urbanization and malaria prevalence identified in previous work^46,47^, as well as specific risks associated with proximity to natural water bodies or rain-fed cropland in rural areas^48^. However, drought effects are highly uncertain, particularly in urban areas. Finally, our statistical model recovers large reductions in mean prevalence during two key intervention periods (1955–1969 and 2000–2015) that saw substantial malaria prevention programmes across the continent (Fig. 2d).

Overall, we found a robust relationship between malaria prevalence and climate, including both temperature and rainfall, that is consistent with expectations based on experimental and ecological evidence. Although climate change is unlikely to be the strongest driver of global trends in malaria endemicity (see the next section), at a local scale, the month-to-month and year-to-year impacts of climate variability can be comparable in scale to the impacts of major interventions (Fig. 2d). These findings suggest that malaria programmes should be responsive to climate at local and national scales, and weather-based early-warning systems could be useful to anticipate near-term disease dynamics^49^.

### Historical impacts of climate change

**Extended Data Fig. 6:**
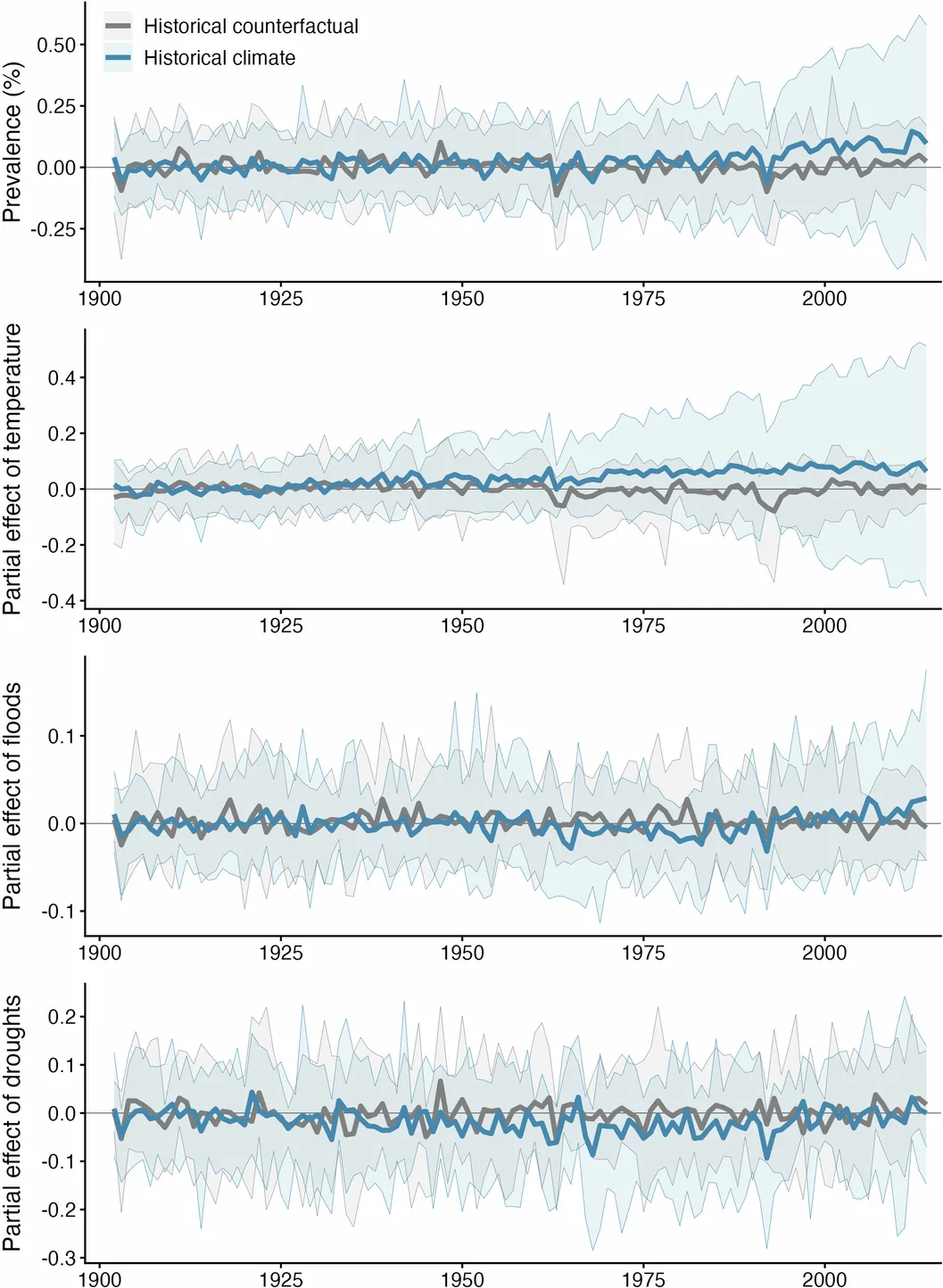
Historical impacts of climate change decomposed by variable. Partial predictions of changes in malaria attributable to anthropogenic climate change are made based on all climate variables (top row), temperature (second row), flood shocks (third row), and drought shocks (fourth row). As in Fig. 2, predictions based on true historical climate (blue) are compared to counterfactual predictions without anthropogenic warming (grey), relative to a 1901 to 1930 baseline. Thick lines are the mean estimate across all 10,000 simulations; shading indicates the 5^th^ and 95^th^ percentiles. Plots begin in 1902 with the first full year of predictions (due to lag effects).

We found that anthropogenic climate change has, more likely than not, been responsible for a small increase in the average prevalence of childhood malaria across sub-Saharan Africa since 1901 (Fig. 2e). Compared with counterfactual simulations without anthropogenic climate forcing, we estimated that by 2010–2014, anthropogenic climate change had caused an increase in continental mean *Pf*PR_2−10_ of 0.07 p.p. (95% CI −0.41 to 0.60 p.p.). Simulations with an attributable increase in continent-wide mean prevalence outnumber those with losses by a factor of 1.4 (proportion P_+_ of 10,000 paired factual versus counterfactual simulations with a positive difference in prevalence = 0.59). These increases are almost entirely driven by rising temperatures from anthropogenic climate forcing; the effects of drought and flood events on prevalence show no distinguishable signal from anthropogenic climate forcing over time (Extended Data Fig. 6).

**Extended Data Fig. 7:**
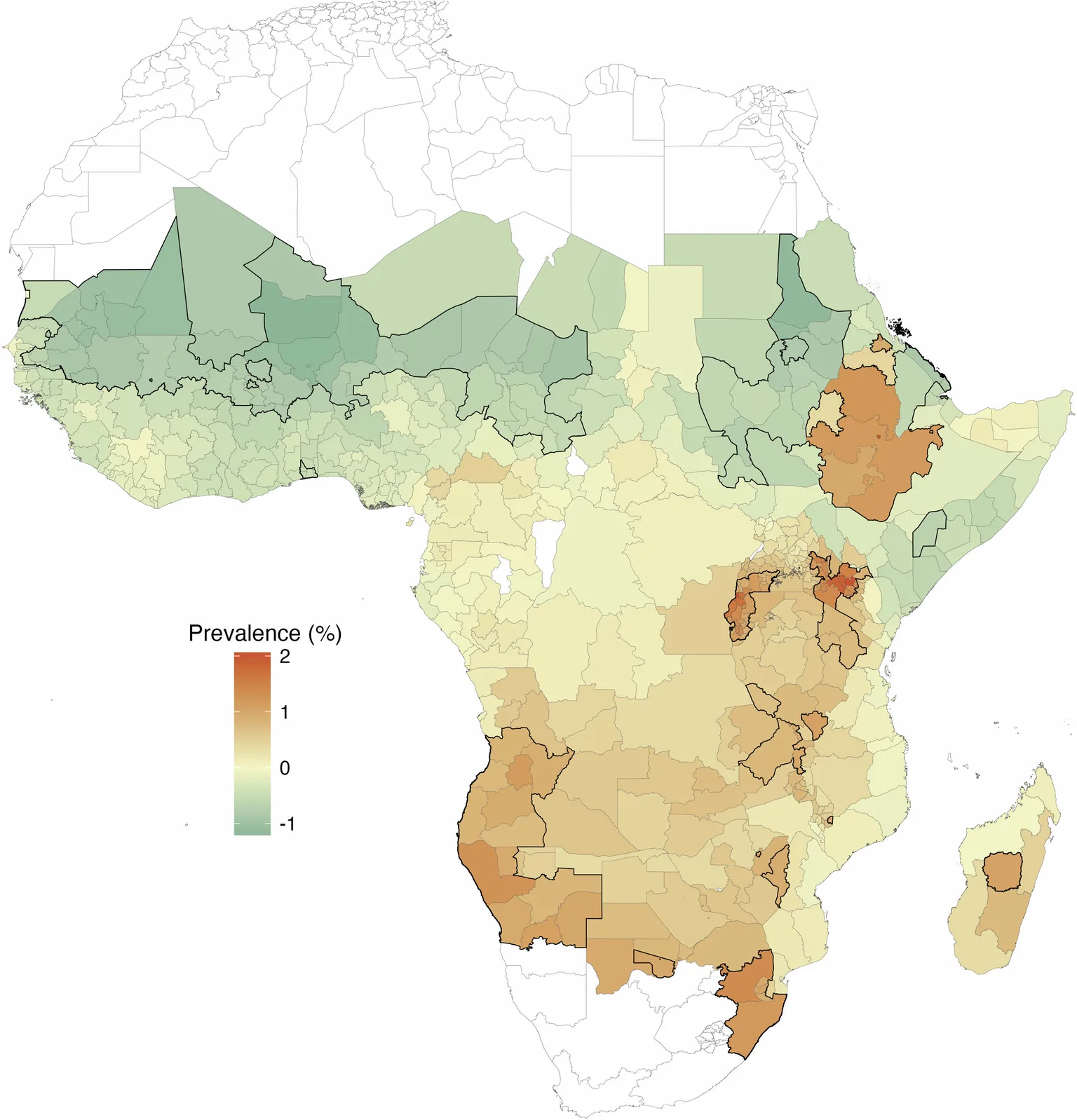
Historical changes in malaria prevalence attributable to anthropogenic climate change from 1901 to 2014. Map shows the estimated change in prevalence attributable to anthropogenic climate change in each administrative unit, based on the difference between the historical climate in 2010-2014 and a counterfactual scenario for the same period simulated without anthropogenic warming. Polygons with a black solid outline indicate areas with changes that were statistically significant (*α* = 0.05) based on the sign of 10,000 simulations. Mean estimates shown here provide the same information as in Fig. 3, but on a single color scale (*i.e*., no uncertainty visualization).

This overall trend masks substantial regional heterogeneity in historical climate change impacts (Fig. 3a and Extended Data Fig. 7), driven almost entirely by elevational and latitudinal gradients in temperature (Fig. 3b,c). For example, attributable changes in prevalence across southern Africa are high in both magnitude and certainty, with an overall increase of 0.60 p.p. (95% CI −0.24 to 1.61 p.p.; P_+_ = 0.91), nearly an order of magnitude greater than the continental mean (Fig. 3d). By contrast, climate change has contributed to much lower malaria prevalence in West Africa (mean = −0.40 p.p.; 95% CI −0.96 to 0.04 p.p.; P_+_ = 0.04), where temperatures already often exceed the biological optimum for transmission. In the central African basin, a stronghold of malaria endemicity with average temperatures close to the 25 °C optimum, the change in prevalence attributable to anthropogenic climate change is positive, relatively small and somewhat uncertain (mean = 0.17 p.p.; 95% CI −0.30 to 0.70 p.p.; P_+_ = 0.75). Finally, we estimated a meaningful overall increase in prevalence attributable to anthropogenic climate change in East Africa (mean = 0.32 p.p.; 95% CI −0.30 to 1.00 p.p.; P_+_ = 0.84), but note that changes in prevalence are distributed unevenly along the steep elevational gradient: increases of up to 1–2 p.p. in the Ethiopian highlands and the greater Rift Valley region are accompanied by small but significant local declines throughout lowland areas in Ethiopia, Sudan, South Sudan, Eritrea and Djibouti. On average across high-elevation East Africa (defined as the first administrative units with average elevation more than 1 km), anthropogenic climate change has raised prevalence by 0.81 p.p. (95% CI −0.07 to 1.88; P_+_ = 0.94).

**Extended Data Fig. 8:**
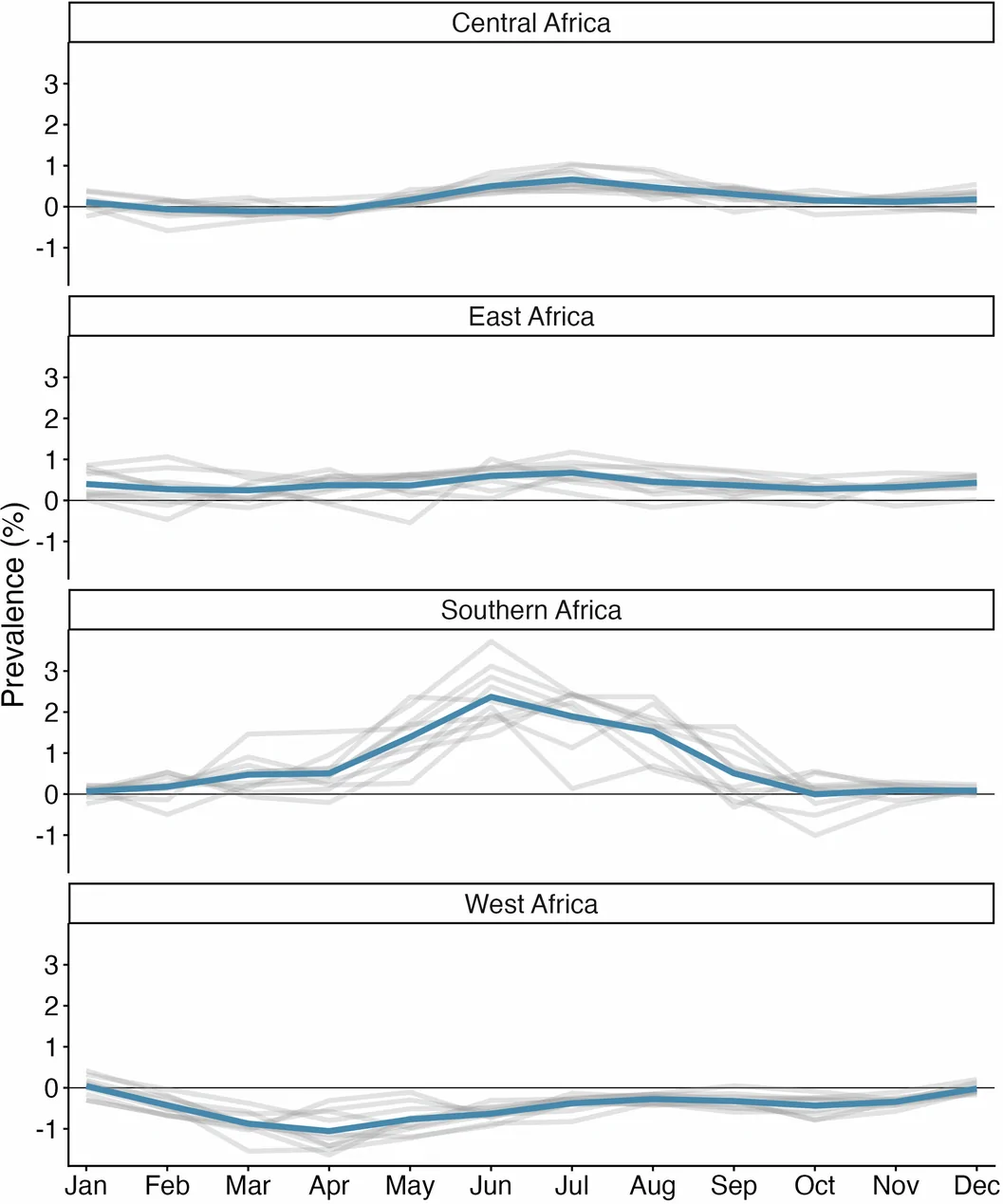
Seasonality in the impacts of anthropogenic climate change on malaria prevalence. Line plots show, for each Global Burden of Disease region, the difference between predicted monthly prevalence under the true historical climate and a counterfactual climate without anthropogenic warming. Positive values indicate that historical climate change elevated malaria prevalence during the indicated month. Impacts are shown as averages by month over the last 5 years (2010-2014) of the historical analysis. Each grey line represents one climate model projection, with the blue line showing the mean across all models. Associated tabular summaries are shown in Supplementary Table 4.

Decomposing these impacts to the monthly level reveals an interplay between space, seasons and shifting burdens (Extended Data Fig. 8 and Supplementary Table 4). In southern Africa, rising temperatures have extended the potential tail end of the malaria season into the winter months (June and July). This supports the long-standing idea that climate change-driven poleward expansion of vector-borne diseases can emerge from shifting season lengths and the constraints they impose on endemicity^6,50^. In the rest of sub-Saharan Africa, however, climate change impacts generally align with existing seasonality: for example, in lowland central and East Africa, climate change impacts are distributed much more evenly across the year, but have a stronger peak in July and August and a weaker peak in December to February^51^. Conversely, in West Africa, the negative effects of temperature are concentrated in the hottest months (April and May), at the lowest point in the transmission cycle.

Although these effects are meaningful, we caution that they are also far smaller than the reduction achieved through healthcare, mosquito nets, vector control and economic development; previous work with the same dataset has estimated a reduction since 1900 of 16 p.p. (that is, a continent-wide decline in average *Pf*PR_2−10_ from 40% in 1900–1929 to 24% by 2010–2015 (ref. ^2^)), whereas our estimates of historical climate change-attributable changes rarely exceed 1.5 p.p. for any individual administrative region. In addition, we estimated that average reductions in prevalence realized during the Global Malaria Eradication Program (1955–1969; estimated reduction averaged over the entire period −4.84 p.p.) and recent programmes such as Roll Back Malaria and the Global Technical Strategy (2000–2015; estimated reduction averaged over the entire period −3.36 p.p.) were substantially larger than the cumulative effects of anthropogenic climate change (Extended Data Table 1). Relatively small and spatially differentiated climate-related changes in burden could have been easily concealed by the greater impact of these programmes, highlighting both the success of elimination programs and the importance of using an empirical approach to isolate the effect of climate from other co-evolving factors.

### Future impacts of climate change

**Extended Data Fig. 9:**
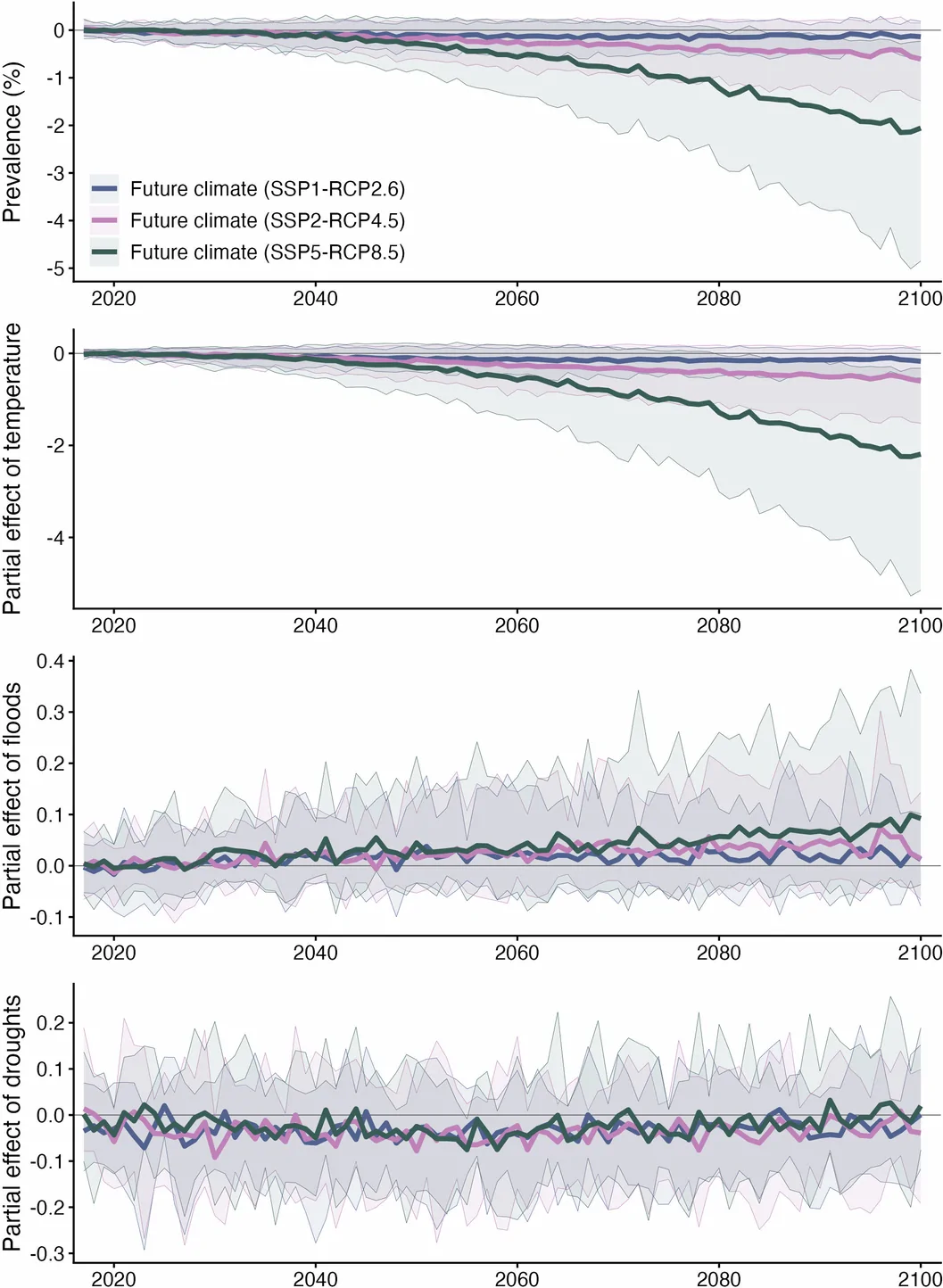
Future impacts of climate change decomposed by variable. Partial predictions of changes in malaria attributable to future climate change are made based on all climate variables (top row) temperature (second row), flood shocks (third row), and drought shocks (fourth row). Projections are shown relative to the mean prevalence from 2015-2020, and as in Fig. 2, line color indicates emissions scenario (blue: SSP1-RCP2.6; pink: SSP2-RCP4.5; green: SSP5-RCP8.5). Thick lines are the mean estimate across all 10,000 simulations; shading indicates the 5^th^ and 95^th^ percentiles. Plots begin in 2016 with the first full year of predictions (due to lag effects).

Despite contemporary trends, we project that within the next quarter-century, anthropogenic climate change will begin to reduce the prevalence of *P.* *falciparum* malaria in sub-Saharan Africa (Fig. 2d and Extended Data Table 2). This trend is largely driven by rising temperatures in lowland areas north of the equator, with greater possible reductions in scenarios of higher greenhouse gas emissions (Fig. 4). In these scenarios, temperature-related declines are slightly offset by floods, which will become more frequent across Africa^31^, although their effect on overall trends is trivial when compared with temperature (Extended Data Fig. 9). Even in a future low-emissions scenario (SSP1–RCP2.6: average global warming across models of +1.8 °C in 2048–2052; +1.9 °C in 2096–2100), increases in prevalence due to historical anthropogenic climate change are projected to essentially be offset by mid-century, stabilizing around an −0.11 p.p. (95% CI −0.48 to 0.20 p.p.) projected decline across sub-Saharan Africa, relative to 2015–2020. In a high-emissions scenario (SSP5–RCP8.5: +2.4 °C in 2048–2052; +5.2 °C in 2096–2100), we project that decreases in prevalence would accelerate over time, reaching an average of −0.29 p.p. (95% CI −0.94 to 0.32 p.p.) by mid-century and −2.04 p.p. (95% CI −5.21 to 0.00 p.p.) by the end of the century, a projected reduction that begins to approach the magnitude of some historical eradication programmes.

**Extended Data Fig. 10:**
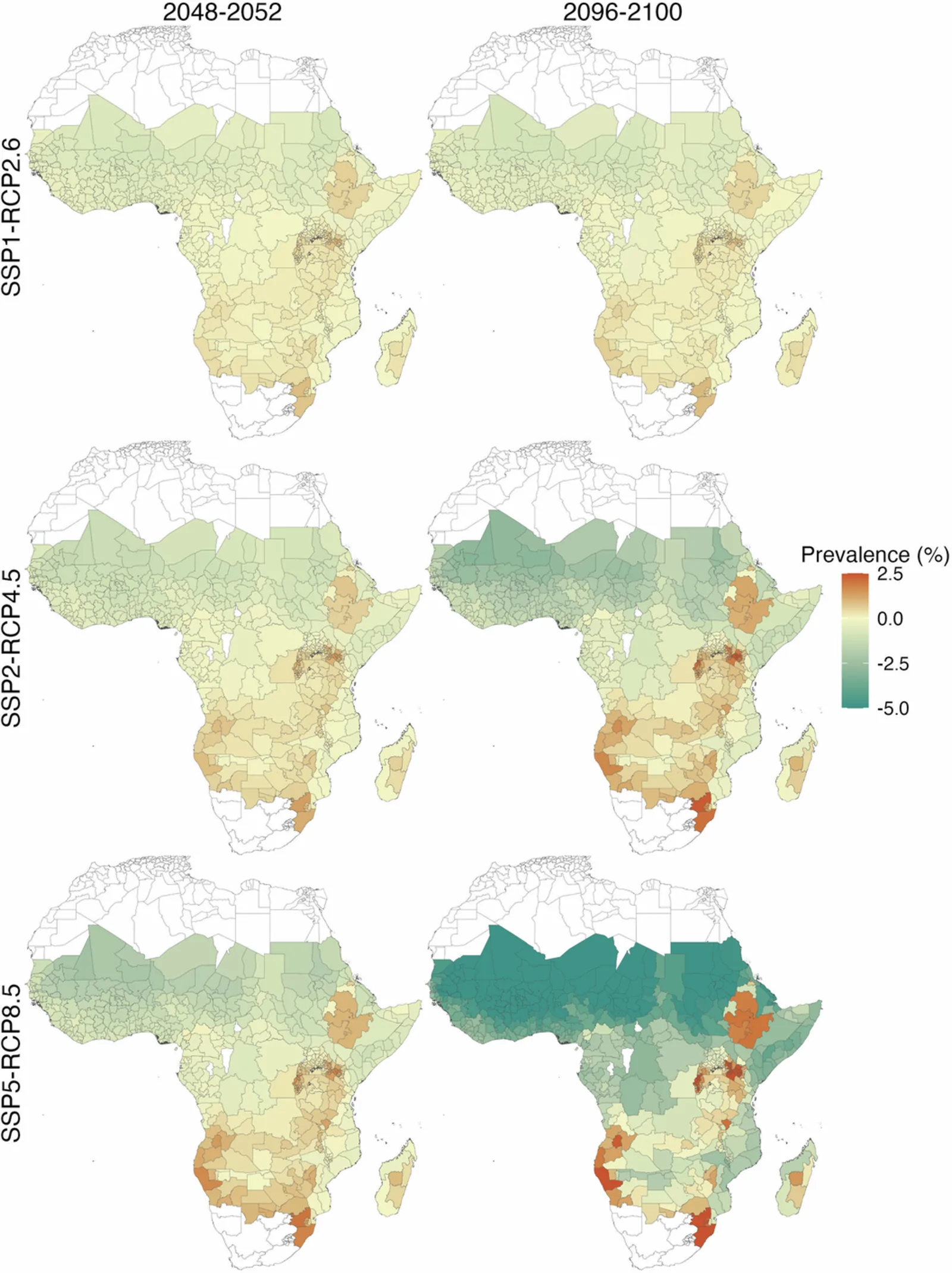
Projected future changes in malaria prevalence driven by climate change from 2015 to 2100. Maps show the estimated change in prevalence due to anthropogenic climate change (in percentage points) in low-emissions (SSP1-RCP2.6; row 1), moderate-emissions (SSP2-RCP4.5; row 2), and high-emissions (SSP5-RCP8.5; row 3) scenarios, projected to mid-century (2048-2052; column 1) or the end of the century (2096-2100; column 2). Projections are reported as differences relative to a present-day baseline (2015-2020). Mean estimates in row 2 column 2 provide the same information as in Fig. 4, but on a single color scale (*i.e*., no uncertainty visualization). The color bar is winsorized for display purposes only, the full range across all regions is -8.5 *p.p*. to 5.7 *p.p*.

Although the balance across regions will begin to shift, the geographical pattern of future changes in malaria prevalence is likely to reproduce present-day heterogeneity in impacts, as malaria transmission continues to shift along latitudinal and elevational clines in temperature (Fig. 4 and Extended Data Fig. 10). West Africa is projected to experience the most dramatic transformation, especially in a high-emissions scenario (SSP5–RCP8.5), with a projected decline of −1.13 p.p. (95% CI −2.00 to −0.38 p.p.) by mid-century and a staggering −4.50 p.p. (95% CI −9.44 to −1.63 p.p.) decrease by 2100. Similar but shallower declines are projected in central Africa, where end-of-century reductions could reach between −0.09 p.p. (SSP1–RCP2.6; 95% CI −0.51 to 0.27 p.p.) and −1.56 p.p. (SSP5–RCP8.5; 95% CI −4.72 to 0.48 p.p.). Conversely, localized increases in prevalence will continue in the cooler parts of the Ethiopian highlands, the greater Rift Valley region and coastal southern Africa, potentially reaching 5 p.p. or more in some areas. The overall effect across East and southern Africa is a projected increase in prevalence, except in the highest emissions scenario (SSP5–RCP8.5), where both regions start to experience declines by mid-century, with East Africa eventually falling −0.68 p.p. (95% CI −3.00 to 1.10 p.p.) below present-day levels by 2100.

Broadly, our results suggest that the main effect of climate change mitigation will be to keep average temperatures in sub-Saharan Africa closer to the optimum range for malaria transmission. However, for many cooler localities, such as in parts of East and southern Africa, greenhouse gas emission reductions may prevent substantial climate change-driven increases in malaria prevalence, although uncertainty is high. For example, by mid-century, limiting global warming to below the +2 °C limit in the Paris Agreement (achieved under SSP1–RCP2.6) is projected to prevent an estimated 2 cases of malaria per 1,000 children in southern Africa (95% CI −2 to 6) compared with an intermediate-emissions scenario (SSP2–RCP4.5 +2.0 °C in 2048–2052; +3.0 °C in 2096–2100). By the end of the century, these benefits could be even greater, with 5 excess cases averted per 1,000 children in southern Africa (95% CI −4 to 14) and in high-elevation (more than 1 km) East Africa (95% CI −3 to 13; Extended Data Table 2 and Extended Data Fig.  10).

### Discussion

In this study, we applied an end-to-end impact attribution framework to a century of malaria surveillance, allowing us to estimate the historical and projected future effect of anthropogenic climate change on childhood malaria in sub-Saharan Africa. We found a 59% likelihood that anthropogenic climate change since 1901 has increased malaria burden; on average across Africa, a small and uncertain number of excess malaria cases (mean of 1 per 1,000 children with 95% CI of −4 to 6) can be attributed to historical human-caused climate change. However, this burden falls disproportionately on southern and East Africa; we estimated a 91% and 84% likelihood, respectively, that anthropogenic climate change has increased present-day malaria prevalence in these regions. We have projected that prevalence in both southern and East Africa will remain elevated in the future: even in a low-emissions scenario likely to limit global warming below +2 °C (SSP1–RCP2.6), we have estimated that these regions will face 3 (95% CI −2 to 10) and 1 (95% CI −4 to 5) excess cases of malaria per 1,000 children by 2100 compared to the present day, respectively. By contrast, across many other regions of Africa, we have projected that the overall impact of future climate change will be a net reduction in malaria: these changes are projected to be most dramatic in West and central Africa, where future climate change could reduce prevalence by up to 45 (West Africa; 95% CI −94 to −16) and 16 (central Africa; 95% CI −47 to 5) cases per 1,000 children in a high-emissions scenario (SSP5–RCP8.5). Our results suggest that climate change could be synergistic with eradication efforts in countries such as Nigeria and the Democratic Republic of the Congo, where the present-day burden of malaria is highest, but will continue to create new risks in countries such as Ethiopia and South Africa.

Spanning multiple centuries, our analysis is the most comprehensive look to date at the effect of climate change on any infectious disease, and brings new clarity to a decades-long debate in malaria research. Whereas some work has questioned the plausibility that overall declines in continent-wide prevalence would conceal a climate-linked increase^2,15^, the 0.074 p.p. increase in *Pf*PR_2−10_ that we attributed to historical anthropogenic climate change could easily be masked by the more than 200-fold greater overall reduction observed across sub-Saharan Africa over the same period^2^. Our regional estimates also generally align with previous laboratory-based or site-specific empirical work, which suggests that East and southern Africa are experiencing shifts towards temperatures that are newly permissive to transmission or have longer malaria seasons^6^, whereas in West and central Africa, climate change impacts have been harder to detect, and future warming might exceed the physiological limits of malaria transmission^6,9^. Of note, our study does provide robust, empirical evidence that human-caused climate change has at least marginally contributed to malaria resurgence in high-altitude Kenya and Ethiopia, consistent with local epidemic time series and simulated dynamics based on local weather station data^17,20,29^.

Our study therefore reconciles three long-standing ideas that are sometimes treated as paradoxical: anthropogenic climate change is not the primary force shaping past, or probably future, trends in malaria prevalence^2,12,15^. However, it is more likely than not that anthropogenic climate change has increased the burden of malaria in sub-Saharan Africa^10,11,17,20^, and at high elevations and latitudes, will continue to for several more decades^6^. Nevertheless, rising temperatures at lower latitudes and elevations in Africa will mostly align with future efforts to eradicate *P. falciparum* from sub-Saharan Africa^6,15,52^. Future work will be needed to situate these global trends in local contexts, particularly through work that leverages longitudinal data from malaria-endemic communities. Similarly, our study provides a long view of future climate change impacts under different emissions scenarios, but cannot be used as a forecast of year-to-year variation in malaria transmission. Future work should explore emerging methods for near-term climate prediction, which could give public health decision-makers information about what to expect over the next year^53^ to decade^54^, the timescale most relevant to malaria control.

In spite of climate change, elimination campaigns have already achieved substantial reductions in malaria endemicity over the past century. Our study underscores that the combined benefits of disease surveillance, healthcare, vector control and economic development can easily counter-balance climate change impacts in most places, and that malaria elimination within the next generation remains plausible, even in the face of climate change. Another recent study has found that passive changes in climate, land use and development will lead to modest reductions in malaria prevalence over the next 25 years, but with 80% effective coverage of chemotherapy, indoor residual spraying and insecticide-treated nets, malaria could be nearly eliminated in sub-Saharan Africa by 2050 (ref. ^55^). In the past few years, the odds of success have become substantially higher thanks to the new RTS,S and R21 malaria vaccines: a four-dose R21 schedule could prevent between one-third and one-half of all malaria cases in children under 5 years of age^56^. Even in places where climate change is increasing malaria transmission, the combined use of classic and new interventions should have a much greater effect, provided that these interventions are able to continue.

At the time of writing, progress towards malaria elimination hangs in the balance, as global health financing faces an unprecedented moment of resource scarcity. Several recent anecdotes have raised relevant concerns about the fragility of elimination, such as the resurgence of malaria in Ecuador and Peru associated with migration from Venezuela^57^, or the estimated 10,000 excess deaths due to malaria—and 3.5 million untreated cases—caused by healthcare disruptions during the 2014 Ebola virus epidemic in West Africa^58^. Concerns about climate-linked resurgence are also more credible given the ongoing invasion of the *Anopheles stephensi* mosquito, which thrives in cities, has already been reported in several locations in East Africa and may be able to transmit *P. falciparum* up to much higher temperatures (approximately 37 °C) than *A. gambiae* can (approximately 30 °C)^5^. Although our data provide suggestive evidence that temperature has historically had a smaller effect on prevalence in urban areas than in rural areas (Extended Data Fig. 5), if *A. stephensi* were to become a dominant vector across the continent, climate change might become an even more pressing concern^59,60^. These risks only add more urgency to the global goals of eliminating both malaria and greenhouse gas emissions.

## Methods

### Malaria prevalence data

We used a recently published database of *P. falciparum* prevalence in sub-Saharan Africa^2^. This compendium, compiled by Snow et al. over more than two decades, is one of the most spatially and temporally complete publicly available databases of infectious disease burden. The database covers the period from 1900 to 2016, although sampling has increased substantially since the turn of the century (pre-2000: *n* = 32,533; post-2000: *n* = 17,892). Most prevalence surveys used microscopy for diagnostics (*n* = 36,805) but a substantial portion of data also derive from rapid diagnostic tests (*n* = 11,154). The data have been compiled from a mix of archival research through public health documents, including the records of colonial governments and elimination campaigns from different periods; national survey data; electronic records published in peer-reviewed journals and grey data sources (for example, World Health Organization technical documents); and a mix of other sources compiled by international organizations. Records were georeferenced in the original study using a standard set of protocols, with a 5-km grid uncertainty threshold for point data, and broader areas stored as administrative polygons. In total, the data include a total of 50,425 prevalence surveys at a total of 36,966 unique georeferenced locations.

The Snow et al. data cover all available prevalence surveys, including all age ranges, but were converted by the authors of the original study to a standardized estimate of prevalence in children 2–10 years of age (*Pf*PR_2−10_), using a catalytic conversion Muench model. We chose to use these standardized estimates of childhood malaria prevalence because *falciparum* malaria has the highest mortality in children and pregnant women. The trends that we infer should generally be representative of broader transmission across age groups. In some cases, we note that declines in early-life exposure can lead to increases in incidence in adults^61^; however, these impacts are likely to be small, particularly given that active and passive improvements in malaria prevention, control and treatment much more directly determine trends in adult malaria risk.

### Climate data

We used two sets of climate data in this study. The first is an observational dataset from the Climatic Research Unit (CRU-TS; v4.03 for model training and bias correction), which is constructed from monthly observations from extensive networks of meteorological stations from around the globe^62^. CRU-TS provides land-only climatic variables at a spatial resolution of 0.5° × 0.5° extending from 1901 to present (although our analysis is limited to the period 1901–2016). The second set of data is from ten global climate models (GCMs) selected from the sixth phase of the Coupled Model Intercomparison Project (CMIP6): ACCESS-CM2, ACCESS-ESM1-5, BCC-CSM2-MR, CanESM5, FGOALS-g3, GFDL-ESM4, IPSL-CM6A-LR, MIROC6, MRI-ESM2-0 and NorESM2-LM. In our historical analysis, we analysed (per GCM) one model realization of the ‘historical’ simulation, which includes anthropogenic greenhouse gas emissions, and one realization from the ‘historical-natural’ simulation, which includes only solar and volcanic climate forcing. For both the historical and historical-natural (hereafter and in the main text, ‘historical climate’ and ‘historical counterfactual’, respectively) simulations, we analysed the period 1901–2014.

To investigate the continued effect of climate change on malaria prevalence between 2015 and 2100, we analysed three CMIP6 future climate change simulations from each of the 10 GCMs. SSPs refer to the level of potential future global development (social, economic and technological) and the implication for climate change mitigation and/or adaptation actions or policy^63,64^. SSPs are combined with various possible future radiative forcings (RCPs) to form the climate change scenarios used in CMIP6. Of the available SSP–RCP scenarios, we selected and used three. The first two suggest enhanced human development outcomes with increased potential towards a more sustainable (SSP1)^65^ or a less sustainable (SSP5)^66^ economy. The third, SSP2 (ref. ^67^), is a mid-way scenario, which assumes a future that mostly follows historical trends^64^. We selected these scenarios in combination with a low (SSP1–RCP2.6), intermediate (SSP2–RCP4.5) and high (SSP5–RCP8.5) greenhouse gas concentration scenario.

We applied a standard quantile–quantile (Q–Q) bias-correction^68,69^ to the CMIP6 precipitation and temperature datasets for both of the historical simulations for the period 1901–2014, and all three future simulations for the period 2015–2100. Before the bias correction, we first remapped all simulated CMIP6 precipitation and temperature datasets to the same grid cell size (0.5° × 0.5°) as the CRU-TS observation data. We then performed for each CMIP6 model, the Q–Q bias correction at each grid point by mapping the quantile values (*q*_*i*_) for the empirical cumulative distribution functions for each of the 12 months over the period 1901–2014 (for each grid point) onto the corresponding quantiles in the observational dataset (CRU-TS), so that the observed precipitation or temperature values associated with *q*_*i*_ become the bias-corrected value in the simulations. For the counterfactual (and future) simulations, we first determined, at each grid point, for each value of precipitation or temperature (for each month) over the period 1901–2014 (2015–2100), the equivalent quantile (*q*_*j*_) in the factual simulation and then identified the precipitation or temperature value associated with *q*_*j*_ in the observational dataset as the bias-corrected value. We detrended both precipitation and temperature datasets before applying the bias-correction procedure, and then added the trends back after^69^.

### Spatial data aggregation

Our statistical analysis is designed to isolate variation in the weather that is uncorrelated with other socioeconomic and/or environmental factors that influence malaria prevalence. As detailed in the next section, we build on a large body of climate econometrics research^38,40,70^ to do so, estimating a model that leverages variation over time in weather conditions within the same location. To estimate such a model, we required observations of malaria prevalence covering the same region in multiple time periods. By contrast, the raw prevalence data that we obtained from ref. ^2^ are point data observations from individual surveys conducted at different times, such that single geolocations are not observed repeatedly over time. Therefore, we aggregated the point-level data from ref. ^2^ by averaging *Pf*PR_2−10_ observations to the first administrative level within each country (that is, state or province level, or as shorthand, ADM1), using shapefiles provided by the Database of Global Administrative Areas dataset v3.6 (www.gadm.org). This level of aggregation provides sufficient granularity to capture differences in climate impacts within countries and to control for local heterogeneity in confounders, while ensuring sufficient data coverage within these units. This aggregation scale has also been conducted in previous work that models this dataset at the same spatial resolution^2^. For robustness, we also show results from a statistical model that does not aggregate data, and instead uses the prevalence data at its native resolution (see below for details).

To compute average prevalence values at the scale of ADM1, we used an unweighted arithmetic mean over all prevalence surveys observed in the corresponding ADM1 month. This approach imposes minimal assumptions on the spatiotemporal process of malaria transmission and requires no additional high-resolution data (for example, population) for use as weights, which are unavailable for sub-Saharan Africa for years as early as 1901. Although previous work aiming to construct comprehensive high-resolution estimates of health outcomes using point data often uses spatiotemporal smoothing methods (for example, ref. ^71^), doing so here would artificially introduce spatial and temporal correlations that could bias recovered regression coefficients and threaten inference^72^.

We similarly aggregate monthly 0.5° grid-level weather data (from all CRU-TS and CMIP6 models) to the ADM1-month level. To do so without introducing aggregation biases, we applied methods from previous research demonstrating that it is possible to statistically recover nonlinear relationships that take place at high spatial and temporal resolution, even when the resolution of available outcome data is relatively coarse (that is, ADM1-month-level average malaria prevalence)^40,73–76^. In our setting, this is achieved by computing nonlinear polynomial transformations of temperature at the grid-cell-by-month level before aggregating these values across administrative units. Such an approach ensures the temperature variables used for estimation reflect the full distribution of temperatures experienced across administrative regions of varying sizes and terrains. For example, many of the 12 ADM1 regions in Ethiopia include both hot low-elevation zones and cold highlands, such that temperatures can vary substantially within an ADM1 during the same month. We computed second-order polynomials at each grid cell before aggregating across such diverse landscapes to ensure the regressor variables capture both extreme cold and extreme heat, even when they occur simultaneously within the boundaries of ADM1.

To see this method in practice, let *Pf*PR_*git*_ denote average malaria prevalence in children 2–10 years of age in grid cell *g* located within administrative unit *i* during month *t* and let *T*_*git*_ indicate temperature observed at the same spatiotemporal scale. Following previous studies recovering local-level quadratic responses between malaria prevalence and temperature^77,78^, we assumed that prevalence in grid cell *g* in month *t* is a quadratic function of the temperature experienced in that same grid cell and month (noting that we show results relaxing this assumption, such as other nonlinear functional forms and the possibility of temporal lags):

1 $$Pf{{\rm{P}}{\rm{R}}}_{git}={\beta }_{1}{T}_{git}+{\beta }_{2}{T}_{git}^{2},$$

where *β*_1_ and *β*_2_ are constant average coefficients. As discussed above, we cannot empirically estimate a model like equation (1) reliably because we did not observe prevalence over multiple months *t* for the same grid cell *g*. Instead, our empirical specification relies on average ADM1-month-level prevalence variables *Pf*PR_*it*_. Thus, we must aggregate equation (1) in a manner that allows us to recover the same *β*_1_ and *β*_2_ coefficients that describe the local-level temperature response, and which we would have recovered had we been able to estimate equation (1) directly. Specifically, average ADM1-month prevalence can be written as:

2 $$\begin{array}{r}Pf{{\rm{PR}}}_{it}=\sum _{g\in i}Pf{{\rm{PR}}}_{git}{\omega }_{gi}=\sum _{g\in i}({\beta }_{1}{T}_{git}+{\beta }_{2}{T}_{git}^{2}){\omega }_{gi}\\ \,=\,{\beta }_{1}\sum _{g\in i}{T}_{git}{w}_{gi}+{\beta }_{2}\sum _{g\in i}{T}_{git}^{2}{w}_{gi},\end{array}$$

where *ω*_*gi*_ denotes a grid-by-ADM1 weight. In our setting, we estimated an area-weighted average prevalence value by setting *ω*_*gi*_ equal to the share of administrative unit *i*’s area that falls into grid cell *g*, as the lack of high-resolution population data make population weighting infeasible.

Equation (2) shows that a regression of average prevalence in ADM1 unit *i* and month *t* on variables that are ADM1-month weighted aggregates of the nonlinear temperature terms *T*_*git*_ and ${T}_{git}^{2}$ will, in expectation, recover the same coefficients *β*_1_ and *β*_2_ that describe the fundamental grid-level relationship described by equation (1). This same procedure has been used to estimate the relationship between: monthly administrative-level dengue incidence and daily grid-level temperature^76^; annual all-cause mortality and daily grid-level temperature^75,79^; annual country-level crop yields and daily grid-level soil moisture^80^; among many other examples. As in these other cases, our approach mitigates aggregation bias up to the level of the grid resolution of the climate data. Although climate and prevalence probably vary across space within each grid cell, we cannot resolve such dynamics here, given the lack of reliable higher-resolution weather data in Africa over the extended time frame of our analysis^62^.

We note that one could, alternatively, construct nonlinear weather variables after aggregating to the administrative unit, thus estimating:

3 $$Pf{\mathrm{PR}}_{it}={\widetilde{\beta }}_{1}\sum _{g\in i}{T}_{git}{\omega }_{gi}+{\widetilde{\beta }}_{2}{(\sum _{g\in i}{T}_{git}{\omega }_{gi})}^{2},$$

where recovered parameters ${\widetilde{\beta }}_{1}$ and ${\widetilde{\beta }}_{2}$ are biased relative to the fundamental relationship in equation (1) because ${({\sum }_{g\in i}{T}_{git}{\omega }_{gi})}^{2} < {\sum }_{g\in i}{T}_{git}^{2}{\omega }_{gi}$, due to Jensen’s inequality.

Thus, we constructed a vector of ADM1-month temperature variables by computing nonlinear transformations at the grid level before aggregating across space, following equation (2). Although we could, in principle, follow the same procedure for precipitation, we instead computed drought and flood variables at the ADM1-month level, due to high rates of mismeasurement in grid-level rainfall estimates^62^ and due to the likelihood that prevalence–precipitation relationships occur over larger spatial scales than a single grid cell (that is, water flows through hydrological systems linking precipitation in one location to water availability and prevalence downstream). We show in a robustness exercise detailed below that using grid-level precipitation data generates similar results as our aggregated model, but increases uncertainty, consistent with evidence on rainfall mismeasurement in Africa.

All main results rely on the spatial aggregation procedure described above. However, we additionally estimated an alternative model that leverages the point-level prevalence data directly, introducing no spatial aggregation beyond the resolution of the weather data (0.5°). As we detail below and show in Supplementary Fig. 9, the recovered prevalence–temperature results are very similar using these two distinct methods.

### Statistical model

The influence of climatic conditions on malaria prevalence has been heavily studied using transmission models based in vector ecophysiology and calibrated using laboratory experiments^3,4^. The important benefit of this approach is that the mechanistic links between a particular environmental condition (for example, temperature) and malaria prevalence in the human population, such as effects on biting rate and survival probability, can be independently isolated. However, this approach is limited in its ability to generalize to real-world contexts, in which complex socioeconomic factors interact with modelled relationships based on laboratory conditions. Clinical data, which measures malaria prevalence in human populations, have been used to validate modelled results^3^, but inconsistent findings arise due to challenges in statistically isolating the role of climate from the many correlated factors influencing prevalence, such as public health interventions, drug resistance, conflict and social instability, and economic shocks^15,52,81–83^.

This study seeks to provide generalizable population-scale evidence of the malaria–climate link across sub-Saharan Africa using field-collected clinical data and a statistical approach designed to isolate changing environmental conditions from spatiotemporal confounding factors. Specifically, we drew on the climate econometrics literature^40^, which has developed causal inference approaches to quantify and project the effects of anthropogenic climate change on a host of socioeconomic outcomes, from agricultural yields^73^, to civil conflict^84^, to all-cause mortality^79^. This approach is designed to approximate controlled experiments by semi-parametrically accounting for unobservable spatial and temporal confounding factors, isolating variation in the climate system that is less likely to be correlated with other socioeconomic factors^85^. This approach is often referred to as ‘reduced form’, as it allows for a plausibly causal interpretation of recovered relationships between socioeconomic conditions and the climate, but it does not easily enable the researcher to isolate individual mechanisms linking a changing climate to shifts in outcomes (for example, mosquito population dynamics or parasite development rates). However, causal estimates enable counterfactual simulation in which climate is changed and all other factors are held constant; this is the exercise conducted here and in many applications of climate econometric frameworks, including estimating the effects of climate change on dengue cases^76^, international human migration^86^, all-cause mortality^75,79^ and more. Moreover, these relationships can be used to calibrate more structured transmission models by providing empirical grounding from observational data.

We developed a statistical model using monthly survey-based malaria *Pf*PR_2−10_ covering all of sub-Saharan Africa over 116 years. Our outcome variable is the average prevalence for each ADM1 *i* (for example, province or state) in country *c* during month–year *t*, which we denote as *Pf*PR_*it*_. We estimated prevalence as a flexible function of monthly temperature and precipitation variables as follows:

4 $$\begin{array}{l}Pf{{\rm{PR}}}_{it}={\beta }_{1}\sum _{g\in i}{T}_{git}{\omega }_{gi}+{\beta }_{2}\sum _{g\in i}{T}_{git}^{2}{\omega }_{gi}\\ \,\,\,\,+\mathop{\sum }\limits_{{\ell }=0}^{L}{\rho }_{{\ell }}{\mathbb{1}}\{{{\rm{drought}}}_{i,t-{\ell }}\}+\mathop{\sum }\limits_{{\ell }=0}^{L}{\psi }_{{\ell }}{\mathbb{1}}\{{{\rm{flood}}}_{i,t-{\ell }}\}\\ \,\,\,\,+\,{\alpha }_{i}+{\gamma }_{rm}+\sum _{c\in C}[{\phi }_{1c}t+{\phi }_{2c}{t}^{2}]\\ \,\,\,\,+\,{\delta }_{1}{\mathbb{1}}{\{{\rm{intervention\; 1}}\}}_{t}+{\delta }_{2}{\mathbb{1}}{\{{\rm{intervention\; 2}}\}}_{t}+{\varepsilon }_{it},\end{array}$$

where *g* subscripts denote grid cells, which fall within administrative units *i*, and *ω*_*gi*_ are area weights equal to the share of unit *i*’s area covered by grid cell *g*, such that ∑_*g*∈*i*_*T*_*git*_*ω*_*gi*_ equals the area-weighted average monthly temperature across all grid cells falling within administrative unit *i*. Together, parameters *β*_1_ and *β*_2_ recover a quadratic response between prevalence and monthly average temperature. As described above, polynomials are computed before aggregating across grid cells to preserve local nonlinearities and avoid aggregation bias. Precipitation extremes are captured by a vector of dummy variables ${\mathbb{1}}\{{{\rm{drought}}}_{i,t-{\ell }}\}$ and ${\mathbb{1}}\{{{\rm{flood}}}_{i,t-{\ell }}\}$, which indicate whether an administrative unit’s monthly rainfall total can be categorized as drought (defined as 10% or lower of the long-run location-specific and month-specific mean) or flood (defined as 90% or higher of the long-run location-specific and month-specific mean) during month–year *t* − *ℓ*. We allowed for up to 3 months of lags (that is, *L* = 3) for these extreme precipitation conditions in our main specification, based on hypotheses from previous literature regarding the timescales of larvae drying and of ‘flushing’^42,43^. Various sensitivity analyses detailed below demonstrate that key findings are robust to: including lags for temperature as well as precipitation (Extended Data Fig. 2); the drought and flood cut-offs used for precipitation (Supplementary Figs. 2, 5 and 6); alternative functional forms of temperature (Supplementary Fig. 4); and the estimation of a grid-level regression that does not aggregate prevalence or weather across space (Supplementary Fig. 9).

Equation (4) uses a suite of semi-parametric spatiotemporal controls to isolate variation in climatological conditions that is independent from other disease transmission factors, following standard practices in the climate econometrics literature^38,40^. First, *α*_*i*_ is a vector of indicator variables for each of 853 ADM1 units across our multi-country sample. These spatial ‘fixed effects’ control for all time-invariant characteristics of an administrative unit that may confound the relationship between temperature, rainfall and prevalence. For example, higher-altitude regions may exhibit cooler temperatures, but they also may be more geographically isolated communities with limited access to malaria prevention interventions. By controlling for mean conditions in each location, these spatial fixed effects avoid conflating climate conditions with other geographical correlates.

Second, *γ*_*rm*_ is a vector of region-by-month-of-year indicator variables, where regions *r* are defined using the Global Burden of Disease regional definitions of West, southern, central and East Africa (see figure 2 in ref. ^87^). Note that the subscript *m* indicates month of the year (for example, February), whereas the month–year index *t* indicates the month–year time index (for example, February, 1998). These spatiotemporal fixed effects *γ*_*rm*_ account for region-specific seasonality in prevalence that may spuriously relate to seasonally varying climatological conditions. We allowed these seasonal controls to vary by region because of large differences in climatological seasonality and in malaria cyclicality across sub-Saharan Africa^88^, and we show below that our main findings are robust to more stringent seasonality controls defined at the country level (Extended Data Fig. 4). Third, *ϕ*_1*c*_ and *ϕ*_2*c*_ are coefficients estimating, for each country *c* in the full set of countries *C*, a nonlinear, country-specific quadratic in the month–year time index, which adjusts the regression for country-specific gradual trends that may confound the malaria–climate relationship, particularly under historical conditions of anthropogenic climate change. Extended Data Fig. 4 shows that our results are robust to multiple alternative approaches to controlling for long-run trends that may vary across space.

Finally, the indicator variables ${\mathbb{1}}{\{{\rm{intervention\; 1}}\}}_{t}$ and ${\mathbb{1}}{\{{\rm{intervention\; 2}}\}}_{t}$ are equal to one when an observation falls into the 1955–1969 or 2000–2015 period, respectively. These two periods saw substantial malaria intervention programmes across the subcontinent, leading to considerable declines in malaria that were unrelated to changes in the climate^2,89^. These indicator variables control for shocks to prevalence during these two periods, and the coefficients *δ*_1_ and *δ*_2_ allow for differential effectiveness of the two distinct intervention periods. Although these variables are strongly correlated with average prevalence and the first is highly statistically significant (Extended Data Table 1), our main findings are robust to their exclusion (Extended Data Fig. 4).

Together, these set of flexible controls imply that the residual variation in temperature and precipitation events used to identify the coefficients *β*_1_, *β*_2_, *ρ*_*ℓ*_ and *ψ*_*ℓ*_ is month-to-month variation over time within the same location, after controlling for gradual country-specific trends, regional seasonality and the aggregate effects of two substantial malaria prevention intervention programmes.

We estimated equation (4) using the lfe package in R. In estimation, we clustered standard errors *ε*_*imt*_ at the country-by-5-year group level to account for spatial correlation within a country and serial correlation within a 5-year time span (see Supplementary Table 5 and the associated discussion on spatiotemporal structure in model residuals below for details on this choice). When computing probabilistic historical and future climate change simulations, we repeatedly resampled coefficients from the clustered variance–covariance matrix so that this same spatial and temporal correlation was accounted for when computing estimates of the impacts of climate change. These resampled draws of both temperature and precipitation coefficients are plotted in Fig. 2. We additionally show sensitivity to alternative methods of capturing uncertainty in Supplementary Table 3 and Supplementary Fig. 7. In Supplementary Fig. 10, we show that model residuals are close to normally distributed, although with slightly heavier tails, making the application of ordinary least squares appropriate in this context.

### Statistical model robustness

In this section, we describe a set of model sensitivity analyses that probe the robustness of our empirical model. Specifically, we investigated the sensitivity of our key findings to: alternative spatiotemporal controls; inclusion of dynamic temperature effects; alternative definitions of extreme rainfall events; alternative functional forms for the prevalence–temperature relationship; and the estimation of a survey-level regression in which the point-level nature of the raw prevalence data are used directly, with minimal spatial aggregation. Finally, we have provided a set of diagnostics investigating the spatiotemporal structure of our model residuals.

#### Spatiotemporal controls

Our preferred empirical specification in equation (4) includes ADM1 fixed effects (that is, indicator variables), region-by-month-of-year fixed effects, country-specific quadratic time trends and two indicator variables for each of two malaria intervention periods (1955–1969 and 2000–2015). Extended Data Fig. 4 shows that our estimated prevalence–temperature relationship is highly robust to many alternative spatial and temporal controls. All panels in this figure include ADM1 fixed effects to control for time-invariant characteristics that may confound the relationship between prevalence and temperature, but each panel varies in the additional spatial and/or temporal controls included in the regression. A tabular version of these results is shown in Extended Data Table 1. Although the temperature at which prevalence peaks changes slightly across model specifications, it remains within a degree of the 24.9 °C value from our preferred specification for most models, particularly those including time trends that are spatially differentiated (note that peak temperatures indicated in Extended Data Fig. 4 are rounded to the nearest degree for display purposes). Predictably, stringent controls, such as region-by-year and country-by-month fixed effects, tend to increase statistical uncertainty. The specification without an expected inverted U shape includes country-by-year fixed effects, which absorb nearly all residual variation in temperature. However, overall, the estimated shape and magnitude of the prevalence–temperature relationship remain robust to alternative spatial and temporal controls.

#### Dynamic temperature effects

Our preferred empirical specification estimates contemporaneous (within 1 month) and lagged (up to 3 months) effects of extreme rainfall on malaria prevalence, but only contemporaneous effects of temperature. Although it is possible that temperature also exhibits lagged effects, we show in Extended Data Fig. 2 that the cumulative effect of temperature on *Pf*PR_2−10_ is similar whether 0, 1, 2 or 3 months of lagged temperatures are accounted for. The prevalence response to temperature does become stronger with 3 months of lags, suggesting that our historical and future climate predictions shown throughout the main text may be somewhat conservative. However, overall, these findings suggest that climate change impact predictions are unlikely to change meaningfully under different assumptions of the lag structure of temperature exposure.

#### Definitions of extreme rainfall events

Our main empirical specification defines drought as months for which total precipitation is less than or equal to 10% of the long-run location-specific and month-specific mean. Flood is analogously defined as months for which total precipitation is greater than or equal to 90% of the long-run location-specific and month-specific mean. Here we investigated the sensitivity of our main findings to these definitions. To do so, we systematically varied both the drought and flood cut-off values, ranging from less than 1% to less than 20% for drought and from more than 85% to more than 95% for flood, respectively. Supplementary Fig. 6 shows that the relationship between malaria prevalence and temperature is insensitive to the definition of drought and flood events. Supplementary Fig. 5 shows that under most drought and flood definitions, extremely low precipitation events have a negative effect on prevalence with a lag of 1–2 months. However, this effect is rarely statistically significant. Supplementary Fig. 2 shows that extremely high rainfall events increase prevalence with a lag of 2–3 months, a result that is statistically significant and generally robust to alternative drought and flood definitions. In general, these sensitivity analyses show that our main findings are not sensitive to the specific definitions of drought and flood used in estimation of equation (4).

#### Temperature’s functional form

Following from theoretical and laboratory-based literature (for example, refs. ^3,4^), we modelled the prevalence–temperature relationship as quadratic. However, Supplementary Fig. 4 shows that this relationship is similar when more flexible functional forms are used. In particular, the temperature at which prevalence peaks changes little when higher-order polynomials are estimated. Estimating higher-order polynomials increases uncertainty, particularly in the tails of the temperature distribution, but point estimates are similar across the majority of the observed temperature range.

#### Estimating a grid-level regression

As described above, our main analysis relies on an ADM1-level regression in which malaria prevalence and weather data are aggregated from higher spatial resolutions to the ADM1 scale. Here we show the results from an alternative approach, in which point-level malaria prevalence survey observations are matched to corresponding 0.5° resolution CRU climate data grid cells and the regression is estimated at this grid level.

Using these disaggregated data, we estimated a regression model analogous to equation (4), but modified to fit the spatial scale of the data. Specifically, we estimated:

5 $$\begin{array}{l}Pf{{\rm{PR}}}_{git}={\eta }_{1}{T}_{git}+{\eta }_{2}{T}_{git}^{2}\\ \,\,\,\,+\mathop{\sum }\limits_{{\ell }=0}^{L}{\lambda }_{{\ell }}{\mathbb{1}}\{{{\rm{drought}}}_{gi,t-{\ell }}\}+\mathop{\sum }\limits_{{\ell }=0}^{L}{\xi }_{{\ell }}{\mathbb{1}}\{{{\rm{flood}}}_{gi,t-{\ell }}\}\\ \,\,\,\,+{\alpha }_{i}+{\gamma }_{rm}+\sum _{c\in C}[{\phi }_{1c}t+{\phi }_{2c}{t}^{2}]\\ \,\,\,\,+{\delta }_{1}{\mathbb{1}}{\{{\rm{intervention\; 1}}\}}_{t}+{\delta }_{2}{\mathbb{1}}{\{{\rm{intervention\; 2}}\}}_{t}+{\varepsilon }_{git},\end{array}$$

where all variables are defined as above for equation (4). In particular, *Pf*PR_*git*_ represents average prevalence for all surveys located in grid *g* falling within ADM1 unit *i* during month *t*, *T*_*git*_ denotes temperature in the same grid and month, and precipitation extremes are captured via dummy variables ${\mathbb{1}}\{{\mathrm{drought}}_{gi,t{\ell }}\}$ and ${\mathbb{1}}\{{\mathrm{flood}}_{gi,t{\ell }}\}$ that indicate when the monthly rainfall total of each grid cell is less than 10% of its long-run month-specific mean (drought) or more than 90% (flood). As for the main model, when estimating equation (5), we clustered standard errors at the country-by-5-year group level.

Two features render this estimating equation distinct from the main analysis. First, temperature and precipitation variables are matched exactly to the grid cell within which the malaria survey was conducted, such that no aggregation is necessary. This increases the precision of the match between weather and outcome variables. Second, the spatial ‘fixed effects’ denoted by *α*_*i*_—that is, indicator variables for each of the ADM1 units in our sample—are estimated at a lower spatial resolution (ADM1) than the data itself (grid). This implies that the weather variation used to identify coefficient vectors **η**, **λ** and **ξ** includes both variation over time within an ADM1, but also across survey locations located within the same ADM1. Thus, although this model has the benefit of leveraging higher spatial resolution in weather, it potentially suffers from omitted variables bias, as weather conditions in different survey locations may be correlated with other unobservable determinants of malaria prevalence (for example, access to healthcare, rates of poverty and proximity to water bodies).

In Supplementary Fig. 9, we show that the malaria prevalence–temperature relationship recovered from estimation of equation (5) is very similar to that estimated from our main regression model in equation (4), suggesting that aggregation of the underlying survey data does not influence the key results of the paper. By contrast, the estimated drought and flood coefficients recovered from the grid-level regression are highly imprecise, consistent with substantial measurement error in local-level precipitation datasets in Africa for much of the twentieth century^62^.

#### Correlations in model residuals

Here we evaluated the extent to which our model residuals are correlated over space and time by calculating correlations between residuals across various subsets of our data, following similar tests in ref. ^90^. Specifically, we calculated correlations across observations that are: (1) within the same ADM1 unit but from different time periods; and (2) from different ADM1 units but within the same time period. For temporal correlations, we computed correlations between temporally consecutive observations within windows of up to 5 years, whereas for spatial correlations, we investigated correlations within countries, across countries, within Global Burden of Disease multi-country regions and based on physical distance (using ADM1 centroids).

Supplementary Table 5 reports mean correlations, as well as the first and third quartiles of the distribution of correlations across different pairs of units. Rows labelled ‘temporal’ quantified correlations over time, whereas rows labelled ‘spatial’ quantified correlations over space. We note that these tests should be interpreted with care, as the malaria prevalence data are highly unbalanced in space and time, often leaving few observations with which to estimate correlations and/or few regional or temporal pairs over which to summarize a distribution of correlations. To ensure interpretability, we restricted analysis to correlations with at least ten observations. The last column in Supplementary Table 5, labelled *N*, indicates the number of pairs for which sufficient data were available to construct correlations for a given grouping. These results reveal moderate serial correlation in residuals, especially for consecutive observations (row 2; mean *ρ* = 0.40 for consecutive month–years within the same ADM1 unit). Spatial correlations range from negligible (mean *ρ* = 0.04 across ADM1s from different countries or regions) to moderate (mean *ρ* = 0.28 and *ρ* = 0.30 for ADM1s within the same country and ADM1s with centroids less than 500 km of one another, respectively). Country boundaries are critical for determining spatial correlations: ADM1s with centroids less than 500 km of one another have high mean correlations within countries (mean *ρ* = 0.30), but low mean correlations when crossing country borders (mean *ρ* = 0.08).

Given these results, we clustered standard errors at the country-by-5-year group level, accounting for correlation in model residuals across all ADM1s within the same country and across all months within the same 5-year window. However, in Supplementary Table 3 and Supplementary Fig. 7, we show sensitivity of our recovered confidence intervals to alternative approaches to standard error estimation.

### Predictions

In both historical and future simulations, we applied the estimated panel regression to calculate the effect of climate change on *Pf*PR_2−10_. Our predictions capture the full range of statistical uncertainty (1,000 model estimates resampled from the clustered variance–covariance matrix) and climate model uncertainty (10 climate models), producing a total of 10,000 estimates of historical or future impacts in any given scenario. Each of these 10,000 estimates was normalized to a long-run baseline (past: 1901–1930; present: 2015–2020) before estimates are averaged, creating an estimate of climate change impacts relative to that baseline. In our historical analyses, we only used these models to estimate changes in prevalence attributable to climate change: although the panel regression model accounts for other historical drivers through the fixed effects structure, these are not the focus of our analysis, and so we choose not to estimate total prevalence including these effects. Similarly, we elected not to make assumptions about non-climate drivers of malaria prevalence in the future, and thus do not apply the model to predict future trends in overall prevalence.

For overall trends (for example, reported in Figs. 2d, 3d and 4d), we generated continent-wide averages or four regional averages using the unweighted average of estimates for each ADM1 unit. This is a deliberate oversimplification, as we did not adjust averages based on either ADM1 units’ land area or the estimated population they contain; we made this decision based on the challenges of reconstructing historical population density at fine scales, as well as the need to otherwise make assumptions about how disease burden is allocated over space (for example, the distribution of transmission across rural or urban areas). For similar reasons, we chose not to estimate the effect of prevalence changes on overall malaria incidence. Although some studies have attempted this using a linear conversion with total population^91^, proper estimation of incidence (and the effects of treatment variables, through prevalence, on case burden) requires malaria transmission models that require substantially more demographic assumptions^89^. Future work could explore both of these methodologically complex directions, and potentially generate finer-scale estimates of how many cases of childhood malaria, and resulting deaths, are attributable to climate change.

### Reporting summary

Further information on research design is available in the Nature Portfolio Reporting Summary linked to this article.

## Online content

Any methods, additional references, Nature Portfolio reporting summaries, source data, extended data, supplementary information, acknowledgements, peer review information; details of author contributions and competing interests; and statements of data and code availability are available at https://doi.org/10.1038/s41586-026-10840-w.

## Supplementary information

Supplementary Information This file contains Supplementary Figures 1–10 and Supplementary Tables 1–5

Reporting Summary

Peer Review File

## Figures and Tables

**Extended Data Table 1:**
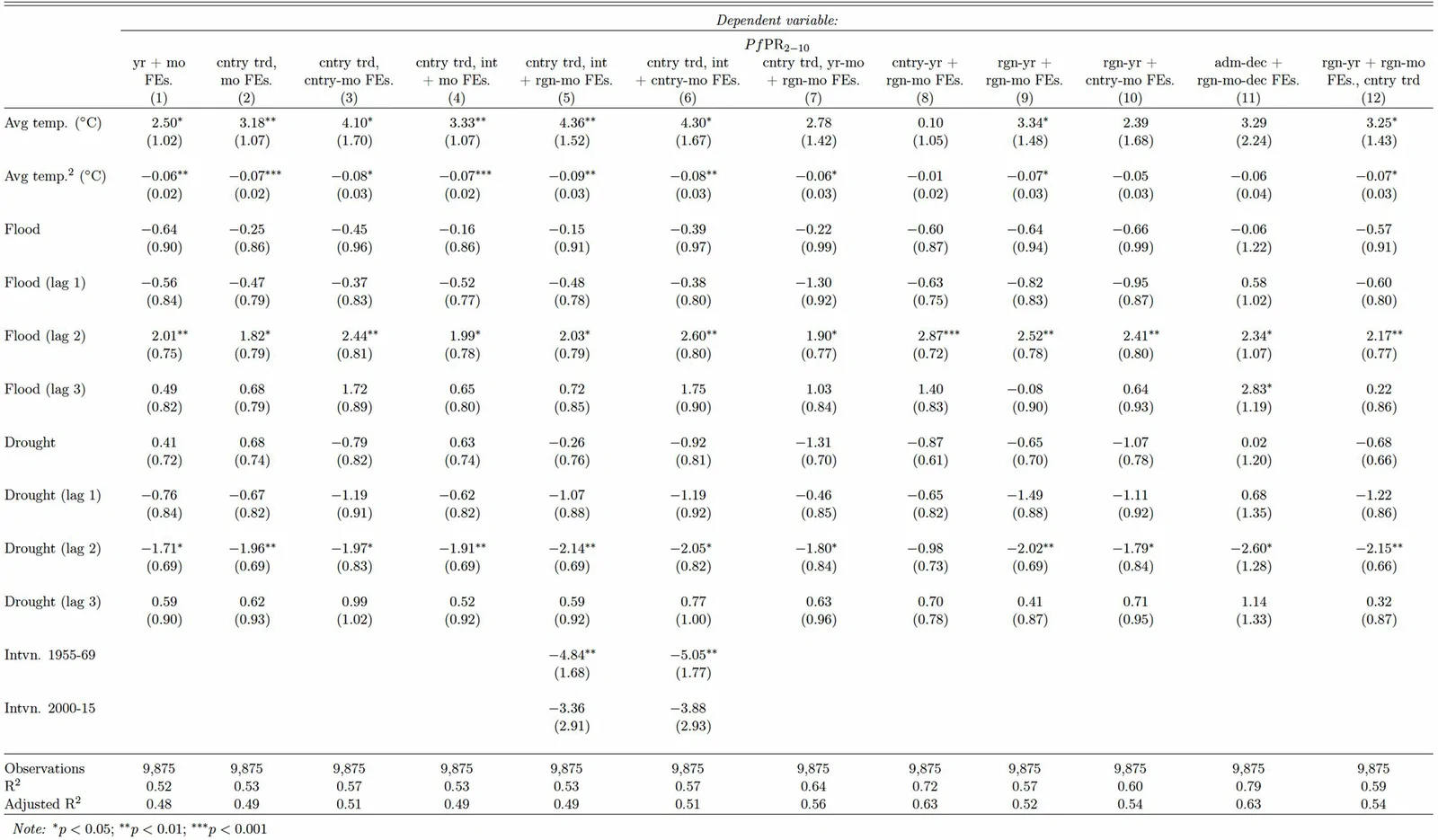
Sensitivity of estimated effects of temperature and rainfall on *Pf*PR_2−10_ to alternative spatiotemporal controls

All columns show regression results for a dependent variable of malaria prevalence for children aged 2-10 (*Pf*PR_2−10_). All include fixed effects (i.e., dummy variables) at the scale of the first administrative unit (i.e., ADM1). Other model controls by column are: (1) year and month fixed effects (“FEs”); (2) country-specific quadratic time trends (“country trends”) and month FEs; (3) country trends and country-by-month FEs; (4) country trends and intervention period and month FEs; (5) country trends and intervention period and region-by-month FEs; (6) country trends and intervention period and country-by-month FEs; (7) country trends and year-by-month and region-by-month FEs; (8) country-by-year and region-by-month FEs; (9) region-by-year and region-by-month FEs; (10) region-by-year and country-by-month FEs; (11) ADM1-by-decade and region-by-month-by-decade FEs; and (12) region-by-year and region-by-month FEs and country-specific linear time trends. The preferred specification used throughout the main text is column (5). “Region” refers to the Global Burden of Disease definitions of West, Southern, Central, and East Africa. All standard errors are clustered at the country-by-5-year group level.

**Extended Data Table 2:**
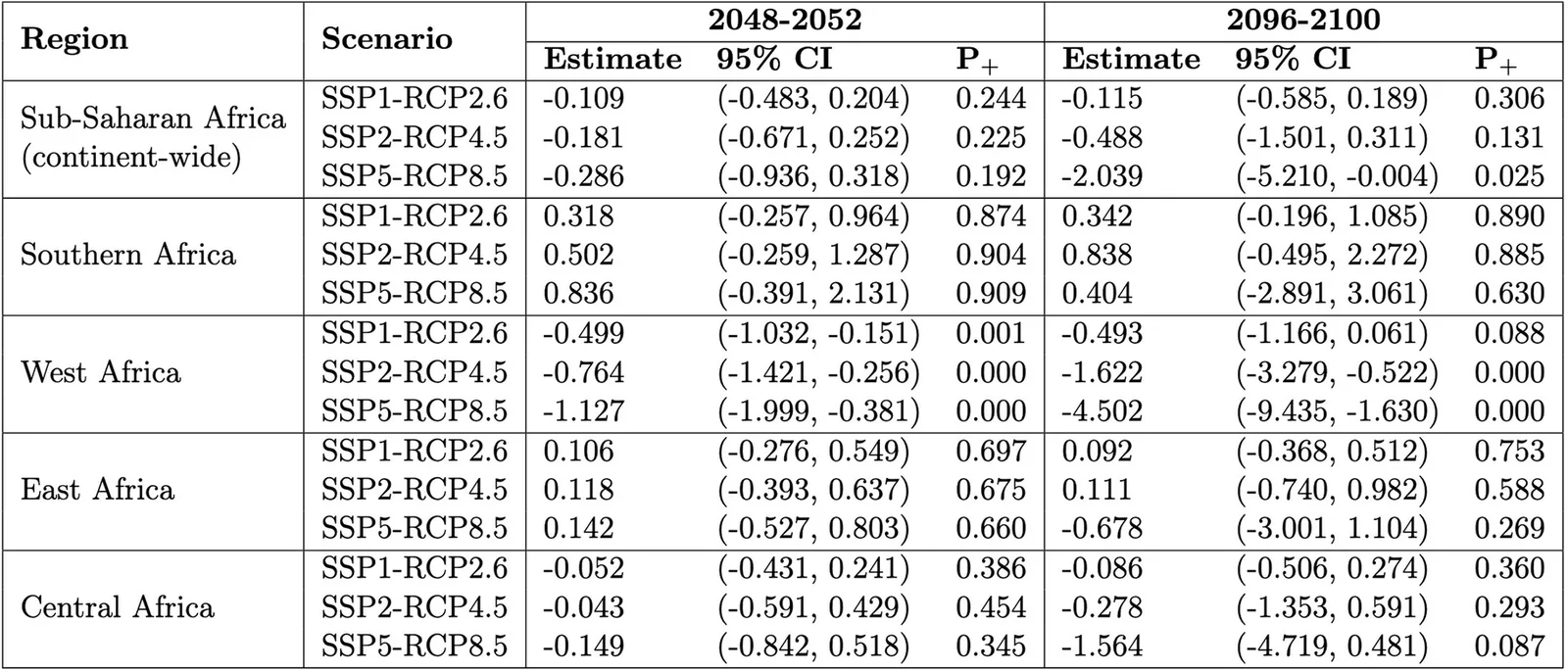
Projected future impacts of climate change on *Pf*PR_2−10_

Estimates and confidence intervals are all given as percentage point changes from a 2015-2020 baseline, estimated across 10,000 simulations. P_+_ indicates the proportion of simulations showing an increase in malaria prevalence relative to the baseline period.

## Data Availability

No original data were generated or reported in our study. All data used in the analyses, including both malaria and climate data, are freely available and referenced in the Methods. For replication purposes, we have made our intermediate data files and analysis-ready pre-processed dataset available on Zenodo^92^. Climate model simulation data are too large for standard public data repositories, but are available on request.

## Code availability

All code is available on GitHub (github.com/cjcarlson/falciparum).
