# Supplementary material for: The past and future impact of climate change on childhood malaria in Africa
Source: Nature. 2026 Jul 29;657(8131):487–94. doi: 10.1038/s41586-026-10840-w (PMC13558053; doi:10.1038/s41586-026-10840-w)
Supplement: Supplementary file 1 [file 41586_2026_10840_MOESM1_ESM.pdf]

nature portfolio

https://doi.org/10.1038/s41586-026-10840-w

Supplementary information

The past and future impact of climate change on childhood malaria in Africa

In the format provided by the authors and unedited

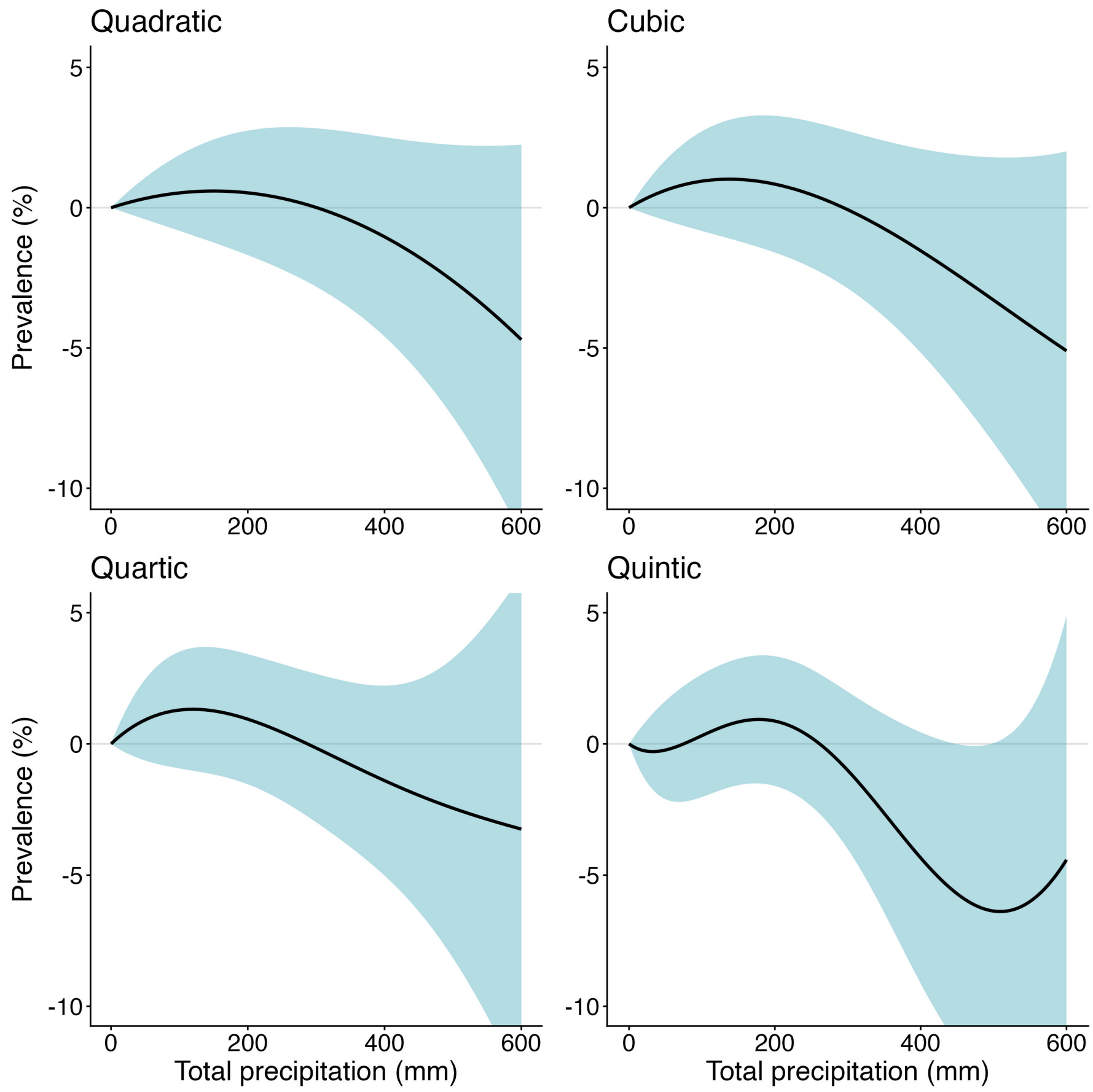

Supplementary Figure 1: **Non-linearities in the PfPR$_{2-10}$-precipitation relationship.** All panels show the estimated relationship between malaria prevalence for children aged 2-10 and monthly cumulative precipitation and are plotted relative to a month with no rainfall. All relationships are estimated for contemporaneous monthly precipitation. All standard errors are clustered at the country-by-5-year group level.

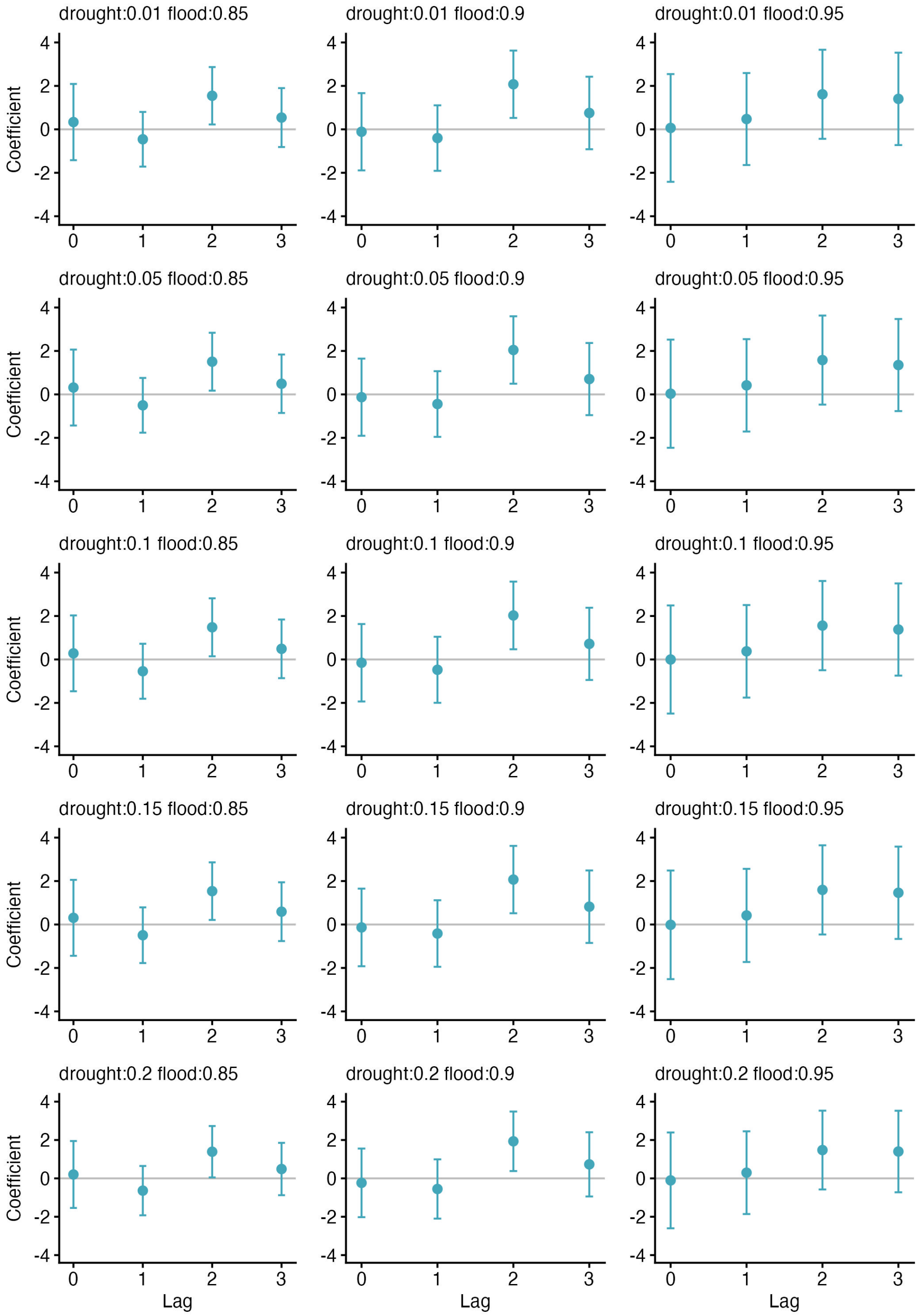

Supplementary Figure 2: **Sensitivity of PfPR$_{2-10}$-flood relationship to alternative drought and flood definitions.** All panels show the estimated relationship between malaria prevalence for children aged 2-10 and contemporaneous and lagged flood events. Point estimates are given by solid circles, while vertical bars indicate 95% confidence intervals. Cutoff values for drought and flood definitions are given in the titles of each panel. For example, the first panel in the upper left defines drought as monthly total precipitation that falls below 1% of the long-run location- and month-specific mean, and defines flood as monthly total precipitation that falls above 85% of the long-run location- and month-specific mean. In all specifications, three months of lagged precipitation extremes are included, as well as all other controls shown in Equation 4. The main specification used throughout the paper uses a drought cutoff of 10% and a flood cutoff of 90%. All standard errors are clustered at the country-by-5-year group level.

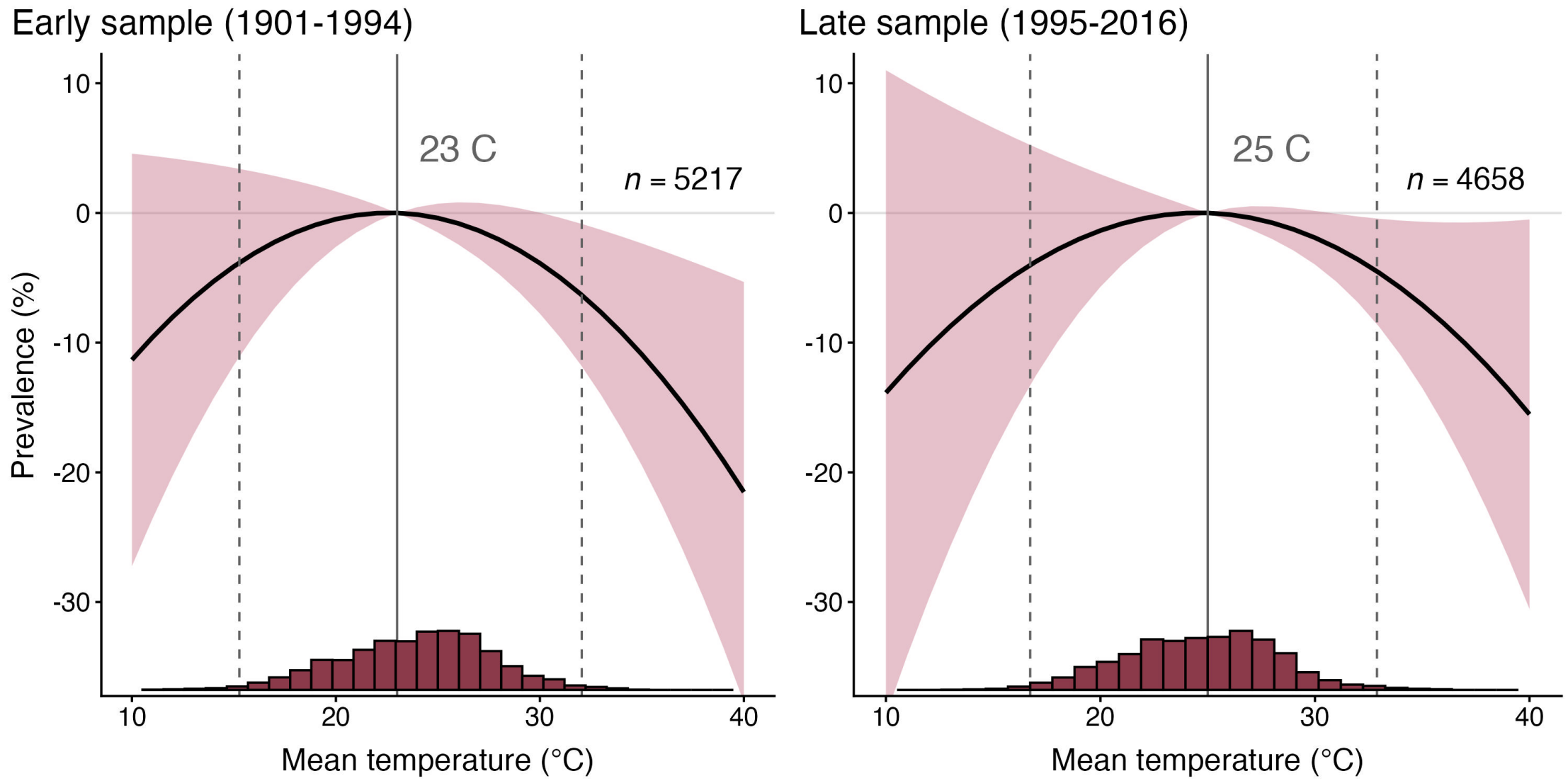

Supplementary Figure 3: **Heterogeneous effects of temperature on $Pf\mathbf{PR}_{2-10}$ over time.** Panels show the estimated relationship between malaria prevalence for children aged 2-10 and monthly average temperature along with a histogram of the associated mean monthly temperature data with the 1st and 99th percentiles of the temperature distributions shown as dashed vertical lines. (Left) The relationship between prevalence and mean monthly temperature for years 1901 to 1995. (Right) The relationship between prevalence and mean monthly temperature for years 1995 to 2016. Both regressions include fixed effects for administrative units and regional seasonality, with standard errors clustered at the country-by-5-year group level.

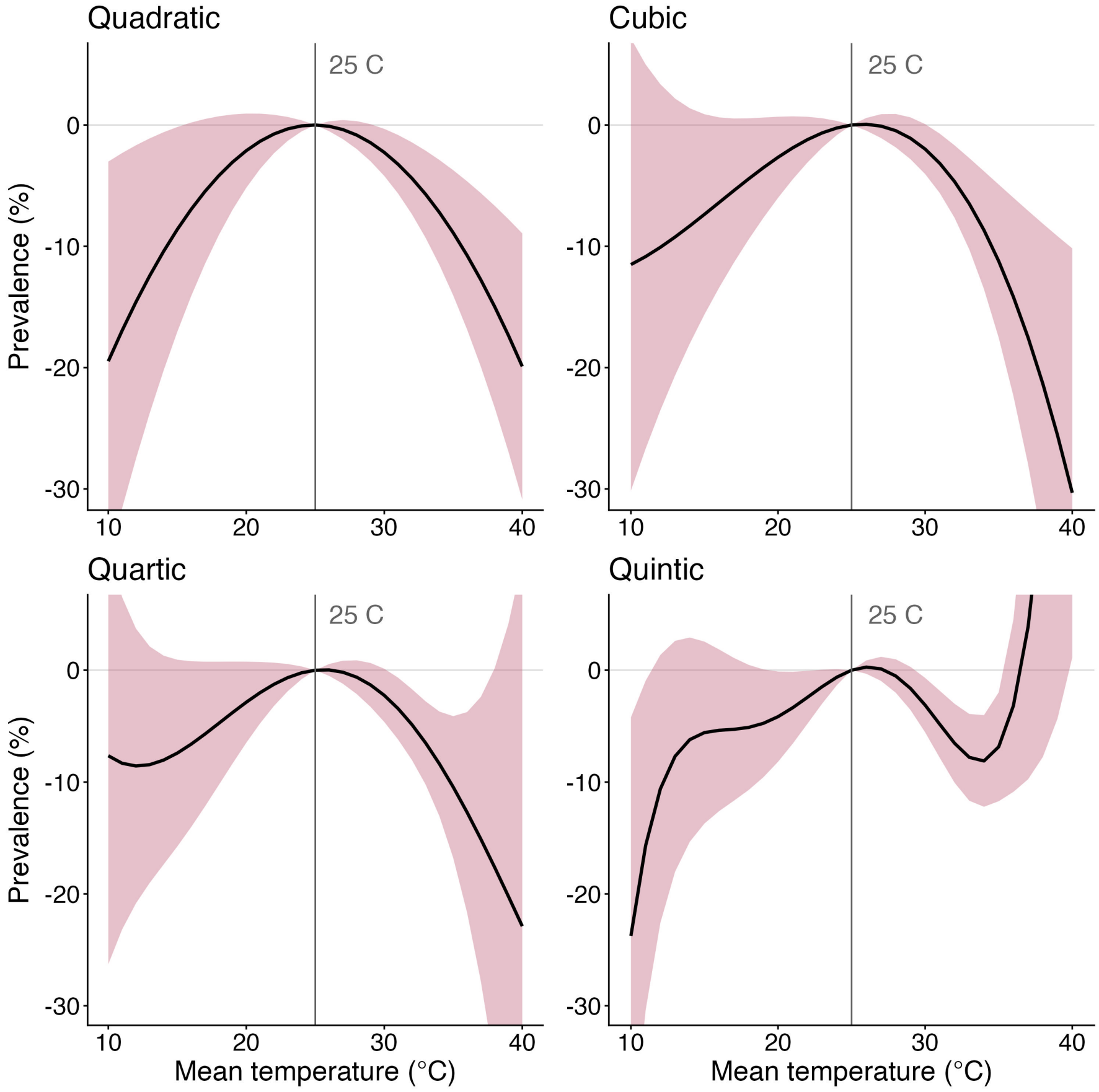

Supplementary Figure 4: **Alternative functional forms for the $\mathit{Pf}\mathbf{PR}_{2-10}$-temperature relationship.** All panels show the estimated relationship between malaria prevalence for children aged 2-10 and monthly average temperature and are plotted relative to a monthly average temperature of 25°C. Starting in the upper left, the first panel shows the paper's main specification, a quadratic functional form for the prevalence-temperature relationship. The second panel shows a cubic functional form, the third a quartic, and the fourth a quintic. All standard errors are clustered at the country-by-5-year group level.

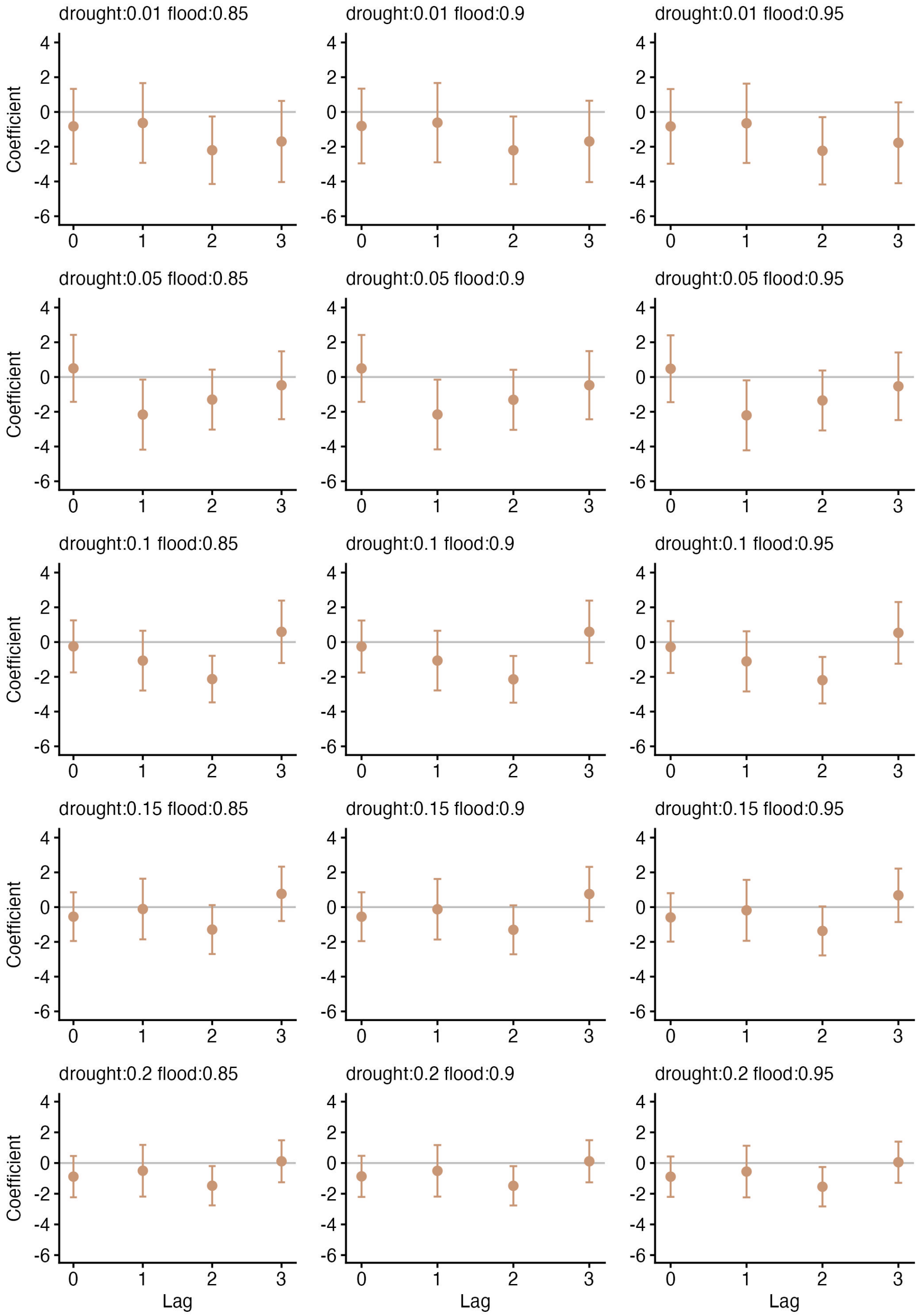

Supplementary Figure 5: **Sensitivity of PfPR$_{2-10}$-drought relationship to alternative drought and flood definitions.** All panels show the estimated relationship between malaria prevalence for children aged 2-10 and contemporaneous and lagged drought events. Point estimates are given by solid circles, while vertical bars indicate 95% confidence intervals. Cutoff values for drought and flood definitions are given in the titles of each panel. For example, the first panel in the upper left defines drought as monthly total precipitation that falls below 1% of the long-run location- and month-specific mean, and defines flood as monthly total precipitation that falls above 85% of the long-run location- and month-specific mean. In all specifications, three months of lagged precipitation extremes are included, as well as all other controls shown in Equation 4. The main specification used throughout the paper uses a drought cutoff of 10% and a flood cutoff of 90%. All standard errors are clustered at the country-by-5-year group level.

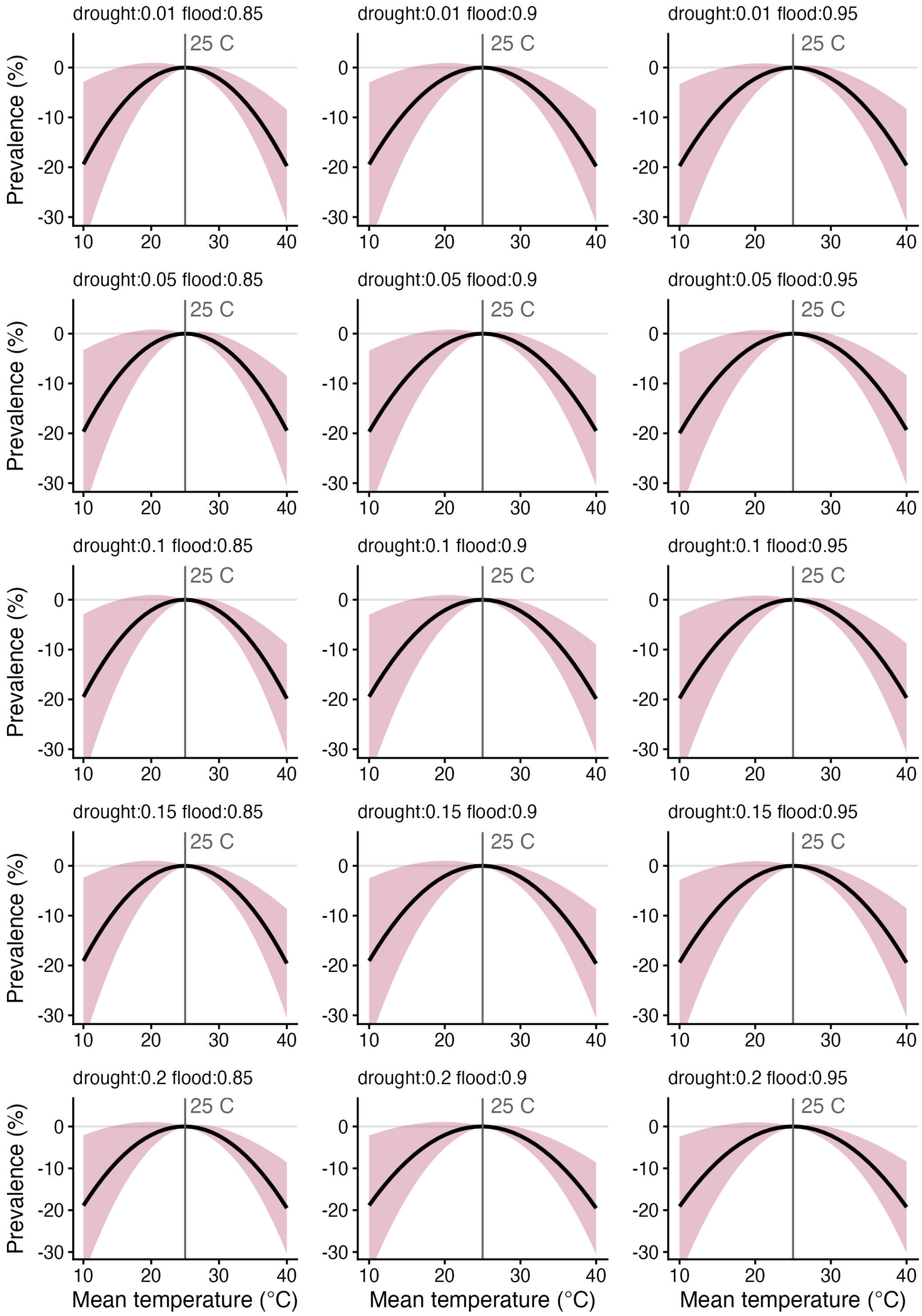

Supplementary Figure 6: **Sensitivity of PfPR$_{2-10}$-temperature relationship to alternative drought and flood definitions.** All panels show the estimated relationship between malaria prevalence for children aged 2-10 and monthly average temperature and are plotted relative to a monthly average temperature of 25°C. Cutoff values for drought and flood definitions are given in the titles of each panel. For example, the first panel in the upper left defines drought as monthly total precipitation that falls below 1% of the long-run location- and month-specific mean, and defines flood as monthly total precipitation that falls above 85% of the long-run location- and month-specific mean. In all specifications, three months of lagged precipitation extremes are included, as well as all other controls shown in Equation 4. The main specification used throughout the paper uses a drought cutoff of 10% and a flood cutoff of 90%. All standard errors are clustered at the country-by-5-year group level.

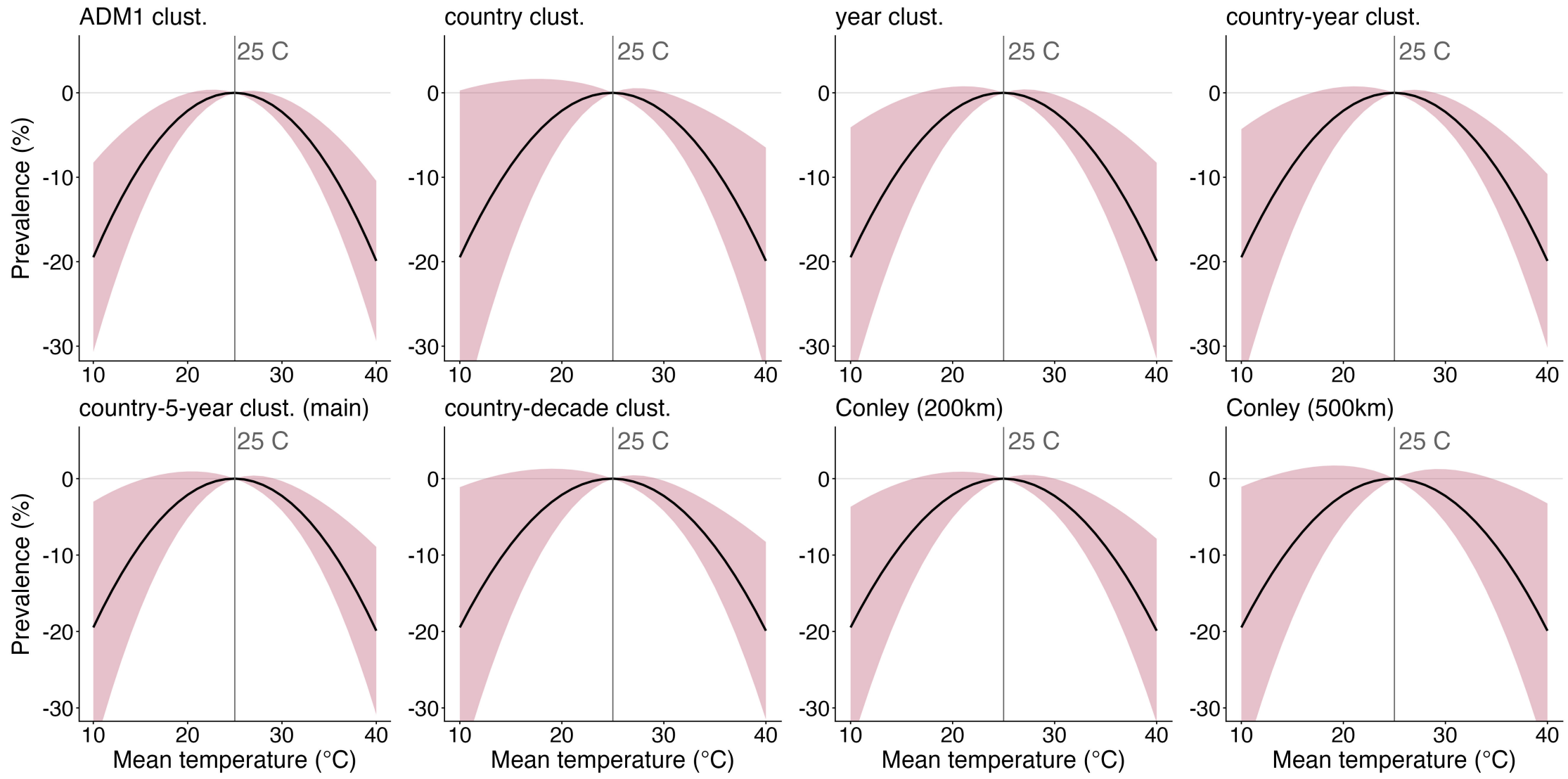

Supplementary Figure 7: **Alternative estimates of model uncertainty in the PfPR$_{2-10}$-temperature relationship.** All panels show the estimated relationship between malaria prevalence for children aged 2-10 and monthly average temperature as estimated following Equation 4. All temperature responses are plotted relative to 25°C, the temperature at which prevalence is maximized. From top-left to bottom-right, standard errors are estimated using: ADM1-level clustering; country clustering; year clustering; country-by-year level clustering; country-by-5-year level clustering; country-by-10-year level clustering; Conley standard errors with a cutoff of 200 km; and Conley standard errors with a cutoff of 500 km. Tabular results, including associated estimates of precipitation extremes, are shown in Supplementary Table 3.

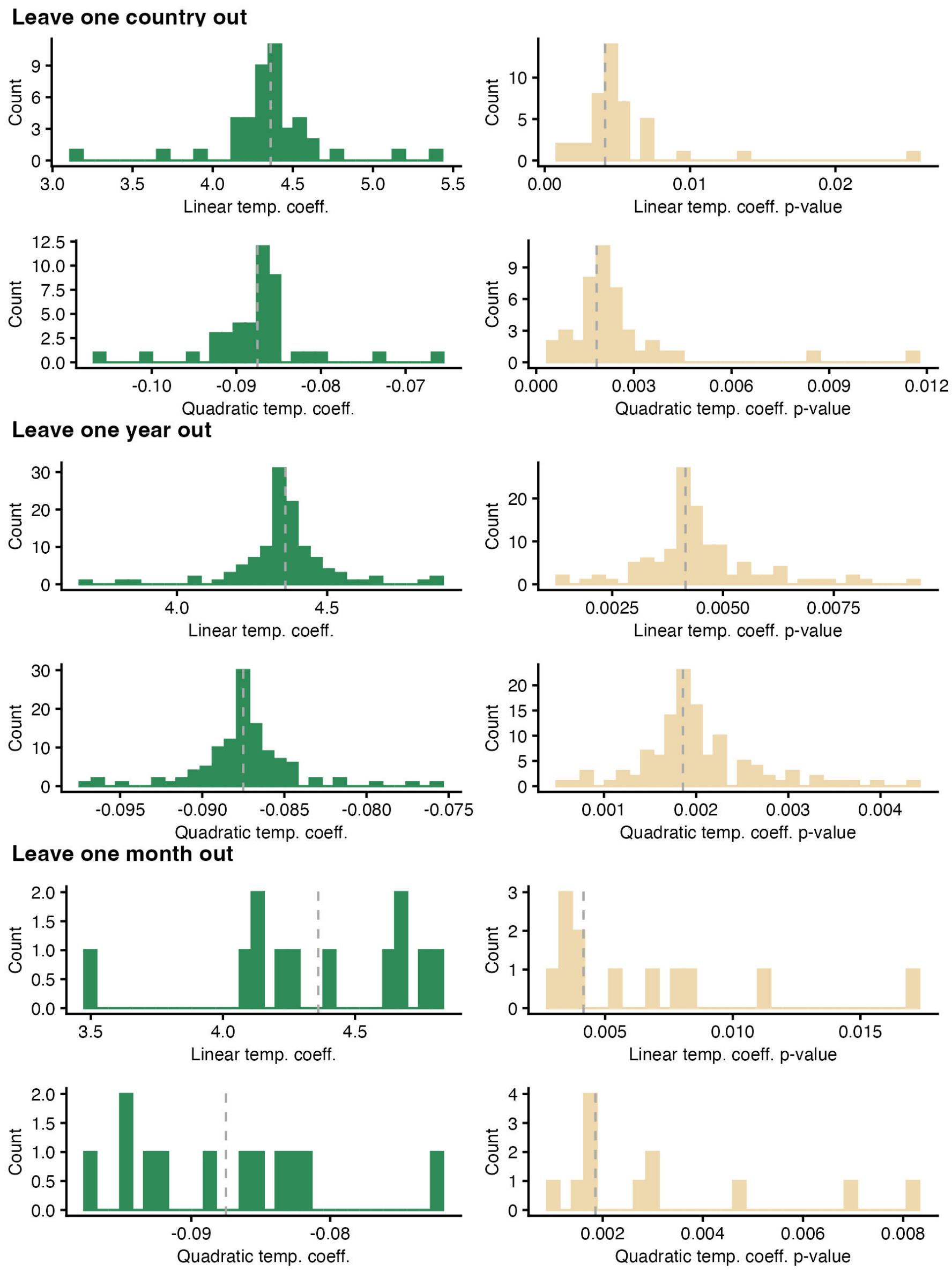

Supplementary Figure 8: **Influence of individual countries, years, and months on the** Pf**PR**$_{2-10}$**-temperature relationship.** All plots show the results from influence analyses in which individual countries (top two rows), years (middle two rows), or calendar months (bottom two rows) are omitted from the sample and the statistical model in Equation 4 is re-estimated. Plots in green show the distribution of regression coefficients on the linear and quadratic temperature terms across all restricted samples, with the full-sample point estimate indicated by the grey dashed line. Plots in beige in the second column show the distribution of associated p-values on each temperature coefficient across all restricted samples, again with the full-sample p-value indicated by the grey dashed line. All regressions were run with standard errors clustered by country-by-5-year group.

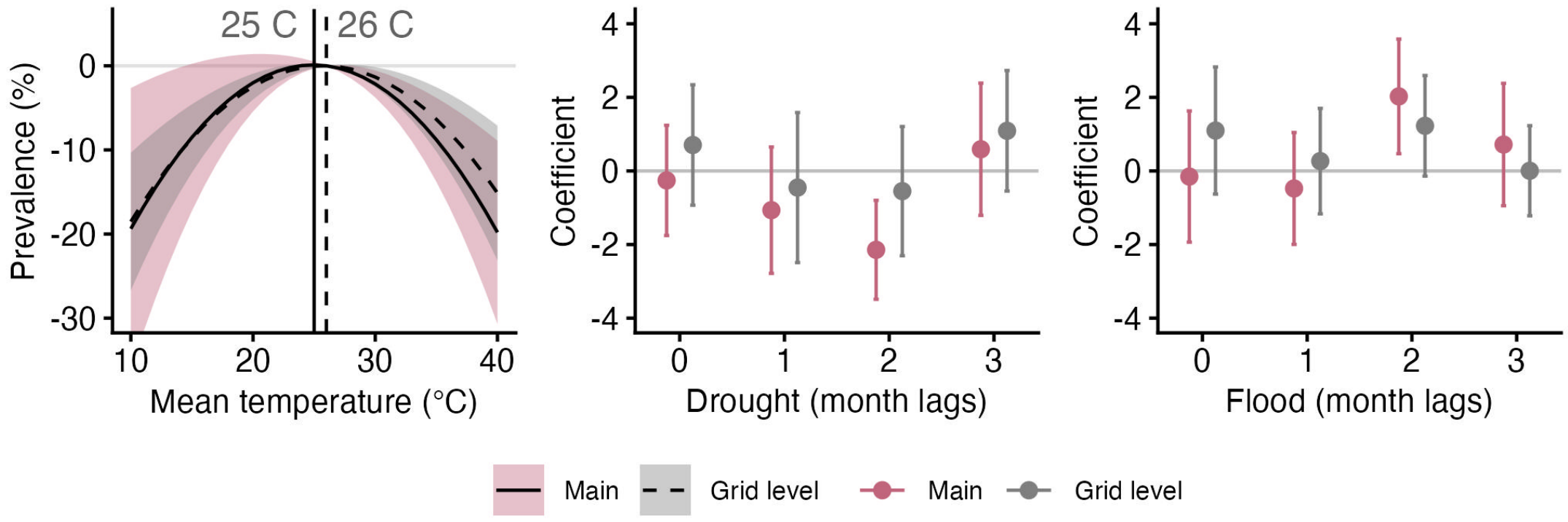

Supplementary Figure 9: **Empirical estimates of prevalence-climate relationships at administrative versus grid cell spatial resolution.** Panels show the estimated relationship between malaria prevalence for children aged 2-10 and monthly average temperature (left), drought (middle), and flood (right), estimated separately for our main model specification, aggregated to the first level of administrative division, and for a high-resolution model aggregated to the CRU climate data grid (resolution 0.5°). (Left) The relationship between prevalence and mean monthly temperature for the main ADM1 model (black solid line and pink shading) and high resolution grid-level model (dashed line and grey shading), with shading indicating 95% confidence intervals. (Middle, right) Estimated coefficients and 95% confidence intervals for the effects of drought (middle) and flood (right) events across contemporaneous and lagged months for both model specifications. Positive coefficients indicate higher malaria prevalence associated with extreme climate events. Models are estimated following Equation 4 (main) and Equation 5 (grid level) and all standard errors are clustered at the country-by-5-year group level.

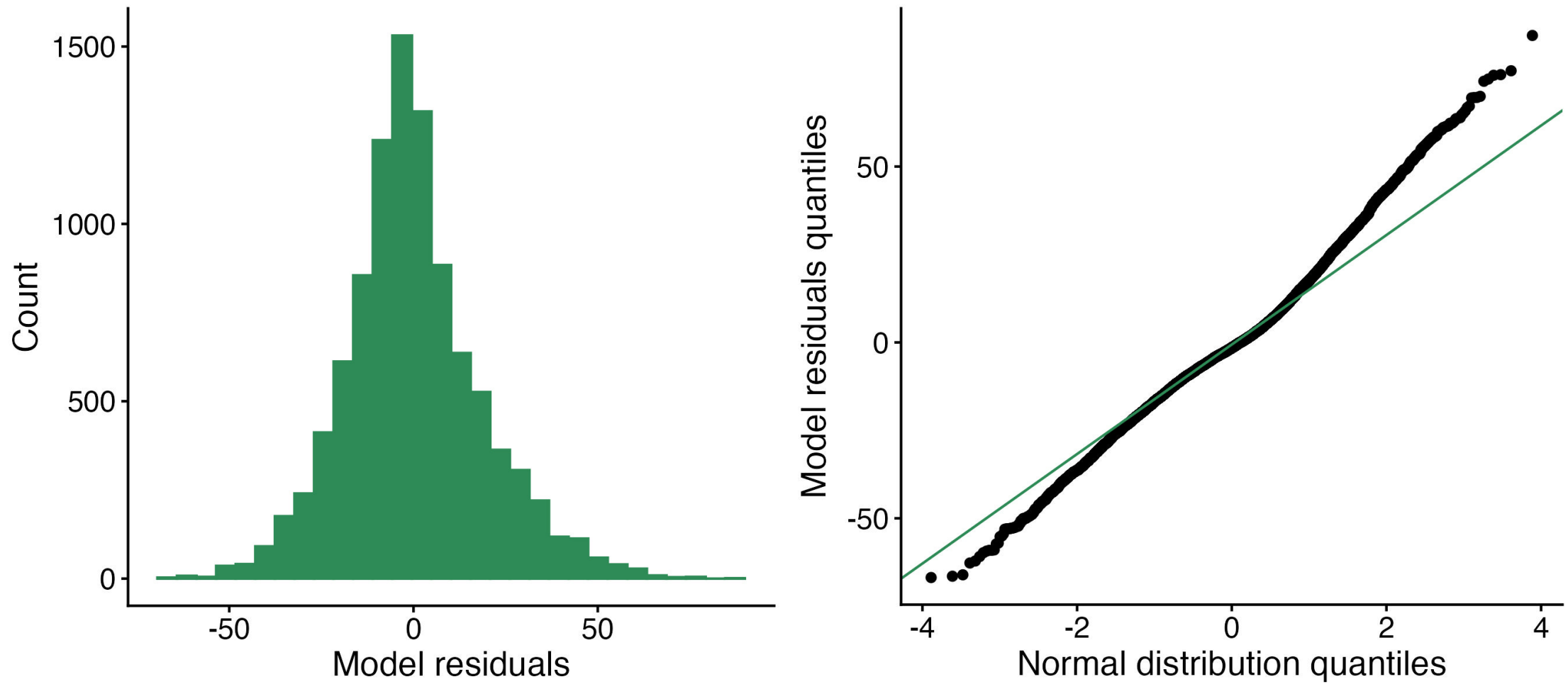

Supplementary Figure 10: **Distribution of model residuals.** (Left) The distribution of regression model residuals from estimating the statistical model in Equation 4. (Right) Q-Q plot showing how the quantiles of the model residuals distribution compare to those from a normal distribution. Residuals are close to normal, with moderately heavier tails indicated by residual quantiles falling below the normal distribution on the lower end, and vice versa on the higher end.

	Dependent variable:		
	$PfPR_{2-10}$		
	CRU (full sample)	CRU (post 1940)	ERA5 (post 1940)
	(1)	(2)	(3)
Avg temp. (°C)	4.36**	4.35**	2.83*
	(1.52)	(1.56)	(1.25)
Avg temp.^{2} (°C)	−0.09**	−0.09**	−0.06*
	(0.03)	(0.03)	(0.02)
Flood	−0.15	−0.17	−0.22
	(0.91)	(0.92)	(1.01)
Flood (lag 1)	−0.48	−0.65	0.53
	(0.78)	(0.80)	(0.82)
Flood (lag 2)	2.03*	2.13**	1.48
	(0.79)	(0.81)	(0.80)
Flood (lag 3)	0.72	1.07	2.03*
	(0.85)	(0.85)	(0.82)
Drought	−0.26	−0.45	0.84
	(0.76)	(0.78)	(0.88)
Drought (lag 1)	−1.07	−1.10	−0.85
	(0.88)	(0.88)	(0.83)
Drought (lag 2)	−2.14**	−2.46***	−0.16
	(0.69)	(0.69)	(0.84)
Drought (lag 3)	0.59	0.86	0.89
	(0.92)	(0.96)	(0.85)
Intvn. 1955-69	−4.84**	−3.75*	−3.67*
	(1.68)	(1.65)	(1.65)
Intvn. 2000-15	−3.36	−0.82	−0.72
	(2.91)	(2.94)	(2.95)
Observations	9,875	9,293	9,293
R^2	0.53	0.54	0.54
Adjusted R^2	0.49	0.50	0.50

Note: $^{*}p < 0.05$; $^{**}p < 0.01$; $^{***}p < 0.001$

Supplementary Table 1: **Sensitivity of estimated effects of temperature and rainfall on $PfPR_{2-10}$ to climate input data.** All columns show regression results for a dependent variable of malaria prevalence among children aged 2–10 ($PfPR_{2-10}$). Column 1 reproduces the preferred specification from Extended Data Table 1, column 5. Column 2 subsets the Climate Weather Unit (CRU) data to the same time span as the European Centre for Medium-Range Weather Forecasts Reanalysis v5 (ERA5) data to make a more direct comparison. Column 3 replaces CRU temperature and precipitation aggregates with those derived from ERA5 monthly data from 1940 to present. Standard errors are clustered at the country-by-5-year group level.

	Dependent variable:		
	$PfPR_{2-10}$		
	(1)	(2)	(3)
Avg temp. (°C)	4.36**	4.18**	4.30**
	(1.52)	(1.51)	(1.52)
Avg temp.^{2} (°C)	−0.09**	−0.08**	−0.09**
	(0.03)	(0.03)	(0.03)
Flood	−0.16	0.05	−0.11
	(0.91)	(0.92)	(0.92)
Flood (lag 1)	−0.46	−0.46	−0.49
	(0.78)	(0.76)	(0.77)
Flood (lag 2)	2.02*	2.12**	2.15**
	(0.79)	(0.79)	(0.79)
Flood (lag 3)	0.72	0.79	0.73
	(0.85)	(0.85)	(0.85)
Drought	−0.25	−0.18	−0.12
	(0.76)	(0.74)	(0.76)
Drought (lag 1)	−1.06	−1.16	−1.05
	(0.88)	(0.88)	(0.88)
Drought (lag 2)	−2.13**	−2.15**	−2.15**
	(0.69)	(0.68)	(0.69)
Drought (lag 3)	0.59	0.47	0.53
	(0.92)	(0.91)	(0.92)
I(intervention)1	−4.86**	−5.02**	−4.80**
	(1.68)	(1.70)	(1.70)
I(intervention)2	−3.38	−3.20	−3.61
	(2.91)	(2.83)	(2.87)
Diag. Method Microscopy - PCR Confirmed		5.99	
		(4.65)	
Diag. Method PCR		27.89***	
		(3.08)	
Diag. Method RDT		2.13	
		(1.77)	
Diag. Method RDT - PCR Confirmed		10.40	
		(11.16)	
Diag. Method RDT - Slide Confirmed		−8.96***	
		(1.95)	
Diag. Method (simplified) PCR			27.66***
			(3.10)
Diag. Method (simplified) RDT			−0.59
			(1.72)
Observations	9,874	9,874	9,874
R^2	0.53	0.54	0.53
Adjusted R^2	0.49	0.50	0.50

Note: $^{*}p < 0.05$; $^{**}p < 0.01$; $^{***}p < 0.001$

Supplementary Table 2: **Sensitivity of estimated effects of temperature and rainfall on $PfPR_{2-10}$ to controlling for malaria diagnostic method.** All columns show regression results for a dependent variable of malaria prevalence among children aged 2–10 ($PfPR_{2-10}$). Column 1 reproduces the preferred specification from Extended Data Table 1, column 5 with a single observation that uses loop-mediated isothermal amplification (LAMP) dropped to make for a direct comparison with columns 2 and 3. Column 2 adds a full set of indicators for the dominant diagnostic method observed in each ADM1–year–month: Microscopy–PCR confirmed, PCR, RDT, RDT–PCR confirmed, RDT–Slide confirmed, and Slide confirmed (Microscopy is the omitted category; a single LAMP observation is dropped). Column 3 replaces these with a simplified method classification that retains only PCR and RDT indicators, again leaving Microscopy as the reference and leaving out the LAMP observation. Standard errors are clustered at the country-by-5-year group level.

	Dependent variable:							
	$PfPR_{2-10}$							
	ADM1 clust. (1)	Cntry clust. (2)	Yr clust. (3)	Cntry-yr clust. (4)	Cntry-5-yr clust. (5)	Cntry-10-yr clust. (6)	Conley (200 km) (7)	Conley (500 km) (8)
Avg temp. (°C)	4.36***	4.36*	4.36**	4.36**	4.36**	4.36**	4.36**	4.36*
	(1.09)	(1.84)	(1.46)	(1.40)	(1.52)	(1.69)	(1.48)	(1.72)
Avg temp.^{2} (°C)	−0.09***	−0.09*	−0.09**	−0.09***	−0.09**	−0.09**	−0.09**	−0.09**
	(0.02)	(0.03)	(0.03)	(0.03)	(0.03)	(0.03)	(0.03)	(0.03)
Flood	−0.15	−0.15	−0.15	−0.15	−0.15	−0.15	−0.15	−0.15
	(0.77)	(0.67)	(0.92)	(0.94)	(0.91)	(0.88)	(0.95)	(1.14)
Flood (lag 1)	−0.48	−0.48	−0.48	−0.48	−0.48	−0.48	−0.48	−0.48
	(0.75)	(0.73)	(0.74)	(0.81)	(0.78)	(0.78)	(0.76)	(0.99)
Flood (lag 2)	2.03**	2.03*	2.03*	2.03*	2.03*	2.03**	2.03*	2.03
	(0.68)	(0.95)	(0.84)	(0.83)	(0.79)	(0.79)	(0.84)	(1.10)
Flood (lag 3)	0.72	0.72	0.72	0.72	0.72	0.72	0.72	0.72
	(0.72)	(1.06)	(0.77)	(0.79)	(0.85)	(0.90)	(0.88)	(1.13)
Drought	−0.26	−0.26	−0.26	−0.26	−0.26	−0.26	−0.26	−0.26
	(0.81)	(0.82)	(0.89)	(0.81)	(0.76)	(0.85)	(0.95)	(1.15)
Drought (lag 1)	−1.07	−1.07	−1.07	−1.07	−1.07	−1.07	−1.07	−1.07
	(0.83)	(0.78)	(1.04)	(0.88)	(0.88)	(0.88)	(0.90)	(0.92)
Drought (lag 2)	−2.14**	−2.14**	−2.14**	−2.14**	−2.14**	−2.14**	−2.14**	−2.14***
	(0.68)	(0.66)	(0.69)	(0.74)	(0.69)	(0.71)	(0.66)	(0.62)
Drought (lag 3)	0.59	0.59	0.59	0.59	0.59	0.59	0.59	0.59
	(0.79)	(0.80)	(0.86)	(0.85)	(0.92)	(0.90)	(0.83)	(0.90)
Intvn. 1955-69	−4.84***	−4.84*	−4.84**	−4.84**	−4.84**	−4.84**	−4.84**	−4.84*
	(1.27)	(2.21)	(1.51)	(1.51)	(1.68)	(1.78)	(1.79)	(2.25)
Intvn. 2000-15	−3.36*	−3.36	−3.36	−3.36	−3.36	−3.36	−3.36	−3.36
	(1.61)	(2.71)	(3.49)	(2.15)	(2.91)	(2.89)	(2.06)	(2.06)
Observations	9,875	9,875	9,875	9,875	9,875	9,875	9,858	9,858
R^2	0.53	0.53	0.53	0.53	0.53	0.53	0.53	0.53
Adjusted R^2	0.49	0.49	0.49	0.49	0.49	0.49	0.49	0.49

Note: $^{*}p < 0.05$; $^{**}p < 0.01$; $^{***}p < 0.001$

Supplementary Table 3: **Alternative estimates of model uncertainty in $PfPR_{2-10}$-weather relationships.** All columns show regression results for a dependent variable of malaria prevalence for children aged 2-10 ($PfPR_{2-10}$) and all are estimated following Equation 4. In each column, standard errors are estimated under different assumptions of spatial and/or temporal correlation in model residuals. Specifications are, from left to right: ADM1-level clustering; country clustering; year clustering; country-by-year level clustering; country-by-5-year level clustering; country-by-10-year level clustering; Conley standard errors with a cutoff of 200 km (column 3); and Conley standard errors with a cutoff of 500 km (column 4). Supplementary Fig. 7 plots the resulting temperature response functions and associated uncertainty.

Region	Month of Peak Impact	Impact Size (% points)	Average Annual Impact (% points)
Central Africa	Jul	0.66 (-0.19, 1.74)	0.17 (-0.30, 0.70)
East Africa	Jul	0.67 (-0.45, 1.97)	0.33 (-0.30, 1.00)
Southern Africa	Jun	2.37 (0.18, 5.14)	0.61 (-0.24, 1.61)
West Africa	Apr	-1.06 (-2.58, 0.37)	-0.40 (-0.96, 0.04)

Supplementary Table 4: **Seasonal peaks in the impacts of historical anthropogenic climate change on malaria prevalence.** Table summarizes key statistics from line plots in Extended Data Fig. 8. For each Global Burden of Disease region, the month of peak impact indicates the month with the largest absolute difference between predicted prevalence with (historical true climate) and without (counterfactual climate) anthropogenic climate change. Impact size represents the increase or decrease in predicted prevalence due to climate change during the month of peak impact, averaged over the 2010-2014 time period. 95% confidence intervals are shown in parentheses. For comparison, average annual impacts and their confidence intervals are shown in the last column (these values correspond to those shown at the end of the annual time series for each region in Fig. 3D).

Kind	Group	$\overline{\rho}$	Q25	Q75	N
temporal	all	0.18	-0.09	0.46	511
temporal	1	0.40	0.20	0.63	60
temporal	2	0.16	-0.07	0.43	33
temporal	3	0.22	-0.05	0.43	16
temporal	within 5 years	0.21	-0.05	0.54	38
temporal	$>$ 5 years	0.17	-0.09	0.45	473
spatial	all	0.24	-0.01	0.54	635
spatial	same country	0.28	0.02	0.56	537
spatial	different country	0.04	-0.19	0.29	98
spatial	same region	0.04	-0.18	0.20	51
spatial	different region	0.04	-0.20	0.32	47
spatial	$<$ 500km	0.30	0.03	0.60	395
spatial	$<$ 500km and different country	0.08	-0.12	0.27	6
spatial	$<$ 500km and same country	0.30	0.04	0.60	389
spatial	$>$ 1000km	0.07	-0.19	0.33	103
spatial	$>$ 500km and different country	0.04	-0.19	0.26	92
spatial	$>$ 500km and same country	0.21	-0.02	0.46	148

Supplementary Table 5: **Summary statistics of temporal and spatial correlations of model residuals.** Values show mean correlation ($\overline{\rho}$), interquartile range (Q25, Q75), and sample size (N) for different groupings temporally (Kind = "temporal") or spatially (Kind = "spatial"), following Schötz (*Nature*, 2025). For temporal groupings, correlations between month-year observations within the same ADM1 unit are estimated. For spatial groupings, correlations between month-year sequences of residuals from different ADM1 units are estimated. Here, "region" indicates one of four Global Burden of Disease regions. Means, first quartiles, and third quartiles of the distribution of correlations from each kind of grouping are shown. N indicates the number of pairs for which correlations could be estimated; pairs with less than 10 matching observations were dropped.
