# Supplementary material for: The past and future impact of climate change on childhood malaria in Africa
Source: Nature. 2026 Jul 29;657(8131):487–94. doi: 10.1038/s41586-026-10840-w (PMC13558053; doi:10.1038/s41586-026-10840-w)
Supplement: Supplementary file 2 [file 41586_2026_10840_MOESM2_ESM.pdf]

nature portfolio

Corresponding author(s): Colin Carlson

Last updated by author(s): May 14, 2026

Reporting Summary

Nature Portfolio wishes to improve the reproducibility of the work that we publish. This form provides structure for consistency and transparency in reporting. For further information on Nature Portfolio policies, see our Editorial Policies and the Editorial Policy Checklist.

Statistics

For all statistical analyses, confirm that the following items are present in the figure legend, table legend, main text, or Methods section.

n/a	Confirmed	
☐	☒	The exact sample size (*n*) for each experimental group/condition, given as a discrete number and unit of measurement
☐	☒	A statement on whether measurements were taken from distinct samples or whether the same sample was measured repeatedly
☐	☒	The statistical test(s) used AND whether they are one- or two-sided<br>*Only common tests should be described solely by name; describe more complex techniques in the Methods section.*
☐	☒	A description of all covariates tested
☐	☒	A description of any assumptions or corrections, such as tests of normality and adjustment for multiple comparisons
☐	☒	A full description of the statistical parameters including central tendency (e.g. means) or other basic estimates (e.g. regression coefficient) AND variation (e.g. standard deviation) or associated estimates of uncertainty (e.g. confidence intervals)
☐	☒	For null hypothesis testing, the test statistic (e.g. *F*, *t*, *r*) with confidence intervals, effect sizes, degrees of freedom and *P* value noted<br>*Give P values as exact values whenever suitable.*
☒	☐	For Bayesian analysis, information on the choice of priors and Markov chain Monte Carlo settings
☒	☐	For hierarchical and complex designs, identification of the appropriate level for tests and full reporting of outcomes
☐	☒	Estimates of effect sizes (e.g. Cohen's *d*, Pearson's *r*), indicating how they were calculated

Our web collection on statistics for biologists contains articles on many of the points above.

Software and code

Policy information about availability of computer code

Data collection	All code used to clean and process data are written in the R software environment (version 4.5.2) and is available at github.com/cjcarlson/falciparum.
Data analysis	All code uses the R software environment (version 4.5.2). All package dependencies can be found at github.com/cjcarlson/falciparum.

For manuscripts utilizing custom algorithms or software that are central to the research but not yet described in published literature, software must be made available to editors and reviewers. We strongly encourage code deposition in a community repository (e.g. GitHub). See the Nature Portfolio guidelines for submitting code & software for further information.

Data

Policy information about availability of data

All manuscripts must include a data availability statement. This statement should provide the following information, where applicable:

- Accession codes, unique identifiers, or web links for publicly available datasets
- A description of any restrictions on data availability
- For clinical datasets or third party data, please ensure that the statement adheres to our policy

No original data are generated or reported in our study. All data used in analyses, including both malaria and climate data, are freely available and referenced in the Methods. Data are publicly available at https://doi.org/10.5281/zenodo.20399793

Research involving human participants, their data, or biological material

Policy information about studies with human participants or human data. See also policy information about sex, gender (identity/presentation), and sexual orientation and race, ethnicity and racism.

Reporting on sex and gender	Results are not specific to sex or gender, nor was sex or gender considered in data aggregation or analysis.
Reporting on race, ethnicity, or other socially relevant groupings	Race, ethnicity, and other social groupings are not considered in this analysis.
Population characteristics	All models use data from children ages 2 through 10.
Recruitment	No recruitment process was conducted.
Ethics oversight	No ethical approval was required.

Note that full information on the approval of the study protocol must also be provided in the manuscript.

Field-specific reporting

Please select the one below that is the best fit for your research. If you are not sure, read the appropriate sections before making your selection.

☒ Life sciences ☐ Behavioural & social sciences ☐ Ecological, evolutionary & environmental sciences

For a reference copy of the document with all sections, see nature.com/documents/nr-reporting-summary-flat.pdf

Life sciences study design

All studies must disclose on these points even when the disclosure is negative.

Sample size	We only reuse publicly available data, and so are limited to the sample size that is available in the Snow et al. (2017) dataset.
Data exclusions	No data are excluded from the analysis.
Replication	This is an observational study using existing datasets, so replicability refers to the stability of the statistical findings rather than repeated experimental trials. All analyses have been reproduced several times by the authors, all of which were successful. Readers can replicate findings using publicly available code.
Randomization	This is an observational study and therefore randomization is not applicable. As detailed in the Methods section, a rich set of parametric and nonparametric controls are used to ensure regression coefficients can be interpreted causally. Specifically, the panel regression model relates monthly malaria prevalence to temperature and precipitation with covariates controlled by first administrative unit (ADM1) "fixed effects" (i.e., indicator variables), region-by-month-of-year fixed effects, country-specific time trends, and indicators for two major intervention periods (1955–1969; 2000–2015).
Blinding	No blinding was conducted or necessary.

Reporting for specific materials, systems and methods

We require information from authors about some types of materials, experimental systems and methods used in many studies. Here, indicate whether each material, system or method listed is relevant to your study. If you are not sure if a list item applies to your research, read the appropriate section before selecting a response.

Materials & experimental systems

n/a	Involved in the study
☒	☐ Antibodies
☒	☐ Eukaryotic cell lines
☒	☐ Palaeontology and archaeology
☒	☐ Animals and other organisms
☒	☐ Clinical data
☒	☐ Dual use research of concern
☒	☐ Plants

Methods

n/a	Involved in the study
☒	☐ ChIP-seq
☒	☐ Flow cytometry
☒	☐ MRI-based neuroimaging

Plants

Seed stocks	*Report on the source of all seed stocks or other plant material used. If applicable, state the seed stock centre and catalogue number. If plant specimens were collected from the field, describe the collection location, date and sampling procedures.*
Novel plant genotypes	*Describe the methods by which all novel plant genotypes were produced. This includes those generated by transgenic approaches, gene editing, chemical/radiation-based mutagenesis and hybridization. For transgenic lines, describe the transformation method, the number of independent lines analyzed and the generation upon which experiments were performed. For gene-edited lines, describe the editor used, the endogenous sequence targeted for editing, the targeting guide RNA sequence (if applicable) and how the editor was applied.*
Authentication	*Describe any authentication procedures for each seed stock used or novel genotype generated. Describe any experiments used to assess the effect of a mutation and, where applicable, how potential secondary effects (e.g. second site T-DNA insertions, mosiacism, off-target gene editing) were examined.*
