# Supplementary material for: The past and future impact of climate change on childhood malaria in Africa
Source: Nature. 2026 Jul 29;657(8131):487–94. doi: 10.1038/s41586-026-10840-w (PMC13558053; doi:10.1038/s41586-026-10840-w)
Supplement: Supplementary file 3 [file 41586_2026_10840_MOESM3_ESM.pdf]

nature portfolio

Peer Review File

The past and future impact of climate change on childhood malaria in Africa

Corresponding Author: Professor Colin Carlson

Any redactions in this file are there to maintain patient confidentiality, the confidentiality of unpublished data, or to remove third-party material.

This file contains all reviewer reports in order by version, followed by all author rebuttals in order by version.

Version 0:

Reviewer comments:

Referee #1

(Remarks to the Author)
This paper is well written and is on a topic that has a long history of research. Unfortunately I do not find this papers conclusions to be convincing and find their analysis to be superficial. They claim to use "state-of-the-art" methods but really are using widely accepted established approaches with old data that has many issues. There are no novel contributions on the methods or data side.

My first major issue is the authors confounding of population at risk and transmission. Climate and temperature affect the mosquito life cycle in a dynamic manner, and these define a broad area, and hence population at risk, but this is not the same as transmission. The step to transmission is highly non-linear and requires information in vector control, drugs, cultural differences, socioeconomic differences, developmental differences (like infrastructure) and so on. The authors make no attempts to account for any of these and therefore are not contributing much new to this problem. Their analysis on climate scenarios could show the changes in population at risk, something others have done at length, but it is simply not possible with their methods to jump from risk to transmission intensity based only on climate and have a sensible counterfactual or forecast.

While previously published, the Snow et al dataset is inappropriate for a number of reasons. It is profoundly biased in terms of its sampling and incredibly sparse for what is supposed to cover over 100 years. The authors present no new prevalence data, and do not utilise recent incidence data from DHIS2 or other forms of survey data (e.g. on pregnant women).

The climate data is monthly, which is what most people in the field have been using for decades, I would have hoped for some more high resolution data for a paper trying to achieve what the authors want. Also the spatial resolution at 50k is incredibly coarse given most prevalence modelling is done at 5km/10km.

The statistical model is far from the "state-of-the-art' methods that the authors claim. Most geostatistical methods (See Peter Diggles work on this) account for both spatial and temporal residuals (or confounding factors as the authors use) through a Gaussian process, which is a more powerful approach than what the authors use. The "causal interpretation" is not causal, its associative, there is no DAG being estimated, and no estimands being inferred (e.g. ATE via targeted maximum likelihood or double machine learning). This is an associative model and the authors should not claim otherwise. Moreover, the g-computation is very superficial, why have they not created an estimand as is done in econometrics or health (e.g. the work of Hernan).

Given point spatial data, why are the authors using admin1? How are they aggregating to get the admin1 estimates, what weighting are they using (see https://www.ncbi.nlm.nih.gov/pmc/articles/PMC8055469/ for example).

I am unclear what the likelihood function is and how equation 1 is estimated. Are they accounting for over-dispersion, why are they bootstrapping as opposed to using Bayesian models that are far more common in this area (again see Model Based Geostatistics by Diggle).

My biggest issue again is what is missing from equation 1, all the factors i described above. The malaria data and methods have existed for a long time, the reason others have not adopted simplistic approaches such as those in this paper is that they are not suitable for the question they are looking to answer.

i am unsure from the paper what are g() and h(), how are the authors accounting for known dynamics of rainfall? for example, rainfall is heavy tailed (https://journals.aps.org/pre/abstract/10.1103/PhysRevE.66.036120) does their monthly mean even make sense in this case? how are these extreme events accounted for in their simple regression model.

Minor comments
line 60 - Gething et al Nature also did this analysis and found the same conclusion as Snow, and therefore the authors attempt to dismiss Snow et als conclusion is not warranted.

line 320 - if using the snow data, it is not clear if the authors have also adjusted for diagnostic type (PCR/RDT) or differences in testing such as the infamous DRC 2006 PCR survey.

line 25 - no uncertainty for these excess cases

(Remarks on code availability)

Referee #2

(Remarks to the Author)
Please find my comments in the attached PDF.

(Remarks on code availability)
The code for the statistical analysis is available. But reproduction seems laborious as only raw data can be downloaded but not pre-processed data, which is the input to the statistical analysis. (Or the description is lacking, so that I was not able to find it).

Referee #3

(Remarks to the Author)
This paper is an ambitious analysis of a large data set on the important question of climate change attribution of malaria burden in Sub-Saharan Africa (SSA). The authors analyze a recently published data set on Plasmodium falciparum clinical prevalence for the last century and for SSA which encompasses most of the global disease burden. The analysis combines a statistical model with observational and simulated climate variables for temperature and precipitation, to consider attribution of change up to 2015 and in simulated future scenarios. Main conclusions are that climate change has had a historical impact, which is regionally differentiated and most evident in East African highlands and South Africa, but small when measured as relative percent change and in comparison to previous reported assessment of intervention effects. Furthermore, future climate change should decrease malaria prevalence in West Africa and only moderately continue to increase it in East and South Africa.

(I am not a climate modeling expert and will therefore only comment on the attribution analysis).

My main concern is whether the question posed here can be addressed in a meaningful way with the malaria survey data, given the spatial and temporal averaging over large areas and across multiple years and transmission seasons. This issue is not convincingly addressed. We are told that the spatial “aggregation scale is supported by previous work that models this data set at the same spatial resolution”. This is not a sufficient argument as the goal of the other study was different, and so were the models. On space, one would want to consider what is the degree of variation in temperature over the areas considered for the main transmission seasons. For example, in regions like the East African highlands the analysis may be smoothing considerable variation in both monthly temperature and malaria prevalence. Perhaps there is no way to do better given the spatial extent that can be considered and the nature of the survey data. But there is no consideration of how the averaging can reduce climate effects. This is a difficult question of course because the global data set itself that is used for the observational climate has also a coarse resolution relative to changes in malaria transmission dynamics in some of these regions.

Similarly, when computing the % increase or decrease attributable to climate change, the monthly malaria prevalence is averaged across months and therefore seasons, and over a window of years, for the baseline and the most recent period respectively. The locations where climate variables are expected to have the strongest effects will be necessarily epidemic and seasonal. Isn’t this approach averaging peaks and throughs in a way that washes out the interesting signal? Wouldn’t the % change be larger if considered only for the months of the transmission season in that location? Asynchrony of seasonality adds a further potential source of smoothing of the signal. One could argue that it is valid to ask the question of attribution at any scale of choice, but I am not clear that this is so and independent from the spatial and temporal scales of malaria population dynamics and temperature variation.

Is it possible to focus on particular regions and address whether the survey data allows higher resolution, and/or whether the

topography and associated variation of climate parameters justifies the spatial aggregation in that location? CRU data may not allow an answer. I agree with the authors that attribution remains “challenging for the downstream impacts of anthropogenic climate change” including in epidemiology. While the authors do formulate the statistical model in ways that should account for effects other than climate, this is done within the limitation of granularity. I am not convinced the question itself is meaningful at the studied granularity, and would have liked to see either convincing discussion of the limitations, or some analysis of the effects of averaging.

Other comments:

1) A country-specific quadratic time trend is included in the model. What is the justification for this functional form? Or at least its interpretation?
2) What criterium was used to select the final form among the possibilities?
3) What is regional seasonality meant to capture?
4) We are told in several places that Figure S6 shows that other specifications of the model do not make a difference. I am not clear on how one should consider the evidence in this figure to reach that conclusion. While the form of the curves is similar, the maximum does move and the uncertainty at the extremes also varies considerably among the panels.
5) It would be valuable to include some Discussion of malaria control in the context of climate change, decadal and interannual variability, instead of emphasizing the “climate vs. control” view.
6) Also, some discussion of the temporal scales of variation that are not considered here, specifically for effects of climate variability, given that climate change will alter decadal and interannual variation as well as extreme events. These are the time scales at which climate will most directly impact public health. Given the approach, this kind of variation is not taken into account here.

(Remarks on code availability)

Referee #4

(Remarks to the Author)
As the authors point out, consideration of the likely impact of climate change on malaria incidence has generated diverse views, often because broad conclusions have been drawn from data collected in a limited area and/or over only a limited period. Thus, the study described in this paper, the most comprehensive attempt to address this issue, that makes use of the unique data set collected by Prof Snow and his colleagues is important and will be of great interest to the malaria community and to others interested in the potential impact of climate change on other vector borne diseases.

The overall conclusions of the study on the likely future impact of climate change on malaria in sub-Saharan Africa are in agreement with the overall perceptions of most malaria epidemiologists with broad experience of sub-Saharan Africa, namely that there will be an increase in incidence of malaria in some areas of East and Southern Africa, especially mountainous areas, and a reduction in incidence in the already hot and dry areas or West Africa. However, it is very helpful to have these suppositions supported by detailed modelling based on results from a very large data set that has been comprehensively analysed, including the analyses of the relative importance of changes in temperature or changes in rainfall.

This reviewer is not qualified to comment on the details of the methods used for the climate science, statistical and modelling approaches that have been used. However, these are clearly described in the methods section of the paper and thus accessible to other reviewers better qualified to comment on these issues. My comments and suggestions are restricted primarily to issues that might be raised by those involved in the more practical aspects of malaria control and how the findings of this study have relevance to their work.

a. Parasite prevalence and age. The prevalence of malaria parasitaemia in children aged 2-10 years has been used widely in epidemiological studies as a measure of the intensity of malaria transmission in a community because in most parts of sub-Saharan Africa a high proportion of infections occur in children in this age group. However, as the intensity of infection declines or increases, the proportion of all infections in a community in those aged 2-10 years will increase or decline but the overall incidence in the community may remain unchanged. This is a potential risk in using the 2-10 age group as the variable of interest and this could be mentioned.
b. Parasite prevalence and clinical malaria. As mentioned above, parasite prevalence in children aged 2-10 years has proved a useful marker for epidemiological studies but the variable of most interest to the malaria control community is the number of cases. The parasite prevalence in children aged 2-10 years is used as the indicator for most of the analyses described in the paper but on page 6 changes in numbers of cases are mentioned. How were these numbers generated – was this an extrapolation from prevalence and, if so, how was this done?
c. Urbanization. Urbanization is likely to continue to have an increasingly important impact on the malaria burden in the coming decades. Movement of rural residents to cities which have little malaria transmission, as is happening in many parts of sub-Saharan Africa, sometimes as a result of climate change, can have a major impact on the parasite rate of a district or regional population. In the past, it could be assumed that moving to a city would reduce the chance of a malaria infection but, as the authors mention in the discussion, the worrying emergence of Anopheles stephensi in African cities may change this assumption. Was urbanization taken into account in the climate change predictions in some way?

d. Heterogeneity of effect. It is helpful in the analyses undertaken to characterize the major ecologically different regions of sub-Sharan with different climate change impacts predicted across a wide region. However, the intensity of malaria transmission can vary substantially over a small area depending on local characteristics, which side of a mountain one lives on, how near a river etc. so that climate change may have a very different impact on communities that are living quite near to each other, and it might be helpful to make this point in the discussion.
e.Magnitude of the climate change effects. Overall, the expected changes in parasite prevalence, although statistically significant and important, are relatively modest in relation to the changes that are likely to occur as a consequence of other factors. For example deployment of a highly effective malaria vaccine could reduce malaria prevalence in 2-10 from 50% to 5% whilst invasion of many large African cities by An. stephensi could have a major deleterious effect. It would be helpful to stress this it in the discussion for the benefit of the general reader so that the relatively modest, although important, changes likely to be induced by climate change can be put into a broader perspective of what is likely to happen to the incidence of malaria in sub-Saharan Africa in the coming decades. It is important that the predicted changes in malaria incidence are presented in an appropriate way in general discussions on the need for mitigation of climate change.

(Remarks on code availability)

Version 2:

Reviewer comments:

Referee #1

(Remarks to the Author)
I thank the authors for their time on this revision. It is quite rare to see such a detailed and long rebuttal, and I realise the effort put into this. I am persuaded that most of my concerns are fully addressed. I really like how the authors have looked at the spatial residuals both in lags and plotting the overall residual (R2). I am still not a fan of how biased the snow data is historically, but I will concede there is nothing the authors can do about this, and it is the only dataset available for this.

My only suggestion is to put alot of these analyses in the supplement, it was helpful for me and I think it is essential for the general readership

(Remarks on code availability)

Referee #2

(Remarks to the Author)
please find my review report in the attached PDF

(Remarks on code availability)

Referee #3

(Remarks to the Author)
The authors have addressed my major comments. I appreciate the additions and clarifications.
I have three minor additional comments concerning the writing:

1) I understand the relevance of pointing out the debate on the effect of climate change in East African highlands. It is not completely accurate to say that the highest profile studies focused on a single data set. It was indeed the case that there was a focus on the Kericho data set because of the sparsity of long retrospective records in the form of time series at a given location. There were additional data for Ethiopia and those studies are cited but only in the Discussion. This is a bit of a mis-representation of what has been done and whether the debate was resolved. The study by Siraj et al. (Science 2014) did put an end to the debate of whether warmer temperatures explained trends in highland falciparum malaria, and to whether one could explain those trends with drug resistance (since a similar trend was observed in vivax malaria , in the absence of such resistance). The study by Rodo et al. (Nature communications 2021) for these same data further show the relevance of temperatures to the temporal patterns of the disease up to the time of the enhanced intervention efforts around 2005.
I would suggest that the following text be edited some:
"Malaria experts have often claimed that observed warming trends are 53 incompatible with long-term reductions in malaria prevalence across Africa, and warned 54 that other factors like drug resistance and funding instability pose a more serious threat to malaria eradication16,18,19 55 . Empirical evidence to test these assumptions is sparse, 56 with the highest profile studies focusing on a single dataset of malaria incidence over 57 several decades at a tea plantation in Kericho, Kenya. Since 2000, over a dozen studies have argued that these data either support 20,21,22,23,24 or undermine25,26,27,28,29,30,31 58 the 59 broader hypothesis that climate change is responsible for a resurgence of malaria in the east African highlands 28,32,33,34,3"

2) The authors have included text on the effect of climate variability as requested. It may be useful to point out in the Discussion that future climate scenarios do not allow the consideration of changes in climate variability at interannual (and decadal) time scales, and that those scales are the ones most relevant to public health interventions. Given the visibility of publications in this journal and the importance of the subject, it would be detrimental to public health efforts for the message to be mis-interpreted as saying that the effects of climate "changes" at the time scales most relevant to their interplay with control will not matter. It will also be useful to point out for readers not familiar with climate science, that climate variability and extremes will also change with climate change, and that the effects of such changes are not examined here.

3) Finally, I understand that the spatial resolution of CRU is the best one can do for the time span of interest here, and I appreciate the addition of the analysis down to that resolution, without the spatial aggregation. I still believe that spatial resolution can be a limiting factor in this study, especially for highlands, but also understand the response of the authors underscoring that the goal is to capture the trends over broad spatial extents. It will be interesting to consider in future studies how these findings compare to conclusions from more detailed studies of longitudinal records (rather than assembled surveys) for shorter periods of time but for higher spatial resolution.

(Remarks on code availability)

Referee #4

(Remarks to the Author)
Other reviewers have raised substantial issues in relation to the methodologies supporting the paper and the authors have made major efforts to respond to their comments. My comments on the more practical issues related to the findings of this study have been addressed appropriately.

(Remarks on code availability)

Version 3:

Reviewer comments:

Referee #2

(Remarks to the Author)
The authors have addressed all of my previous points. The effort they have made to verify the robustness of the climate change-prevalence relationship is exemplary, and the temperature effect is a statistically significant and important finding.

I have one remaining concern. While the temperature response function is well-supported, several downstream attribution claims are not statistically significant, and the current reporting of these in the abstract and discussion risks being misunderstood. I want to flag three passages.

Passage 1, Abstract:
"Comparing historical climate reconstructions to counterfactual simulations without anthropogenic climate forcings, we find a ~60% likelihood that human-caused climate change has increased the overall prevalence of childhood malaria across sub-Saharan Africa since 1901, with >90% likelihood in southern Africa and high-elevation east Africa."

The word "likelihood" implies a probability that the claim is true in the Bayesian sense. The supporting statistic is P+, the fraction of paired simulations showing a positive effect, derived from a frequentist sampling distribution (the clustered variance-covariance matrix of the estimator) combined with a 10-member GCM ensemble. P+ corresponds approximately to (1 – one-sided p-value) for the null hypothesis of no positive effect.

For the continent-wide claim, P+ = 0.59 corresponds to a point estimate of 0.07 p.p. with a 95% CI of (-0.41, 0.60), clearly not statistically significant. For the southern Africa claim, P+ = 0.91 corresponds to a point estimate of 0.60 p.p. with a 95% CI of (-0.24, 1.61); the one-sided p-value is approximately 0.09, which also does not meet conventional significance thresholds.

Passage 2, Discussion:
"We find a 59% likelihood that anthropogenic climate change since 1901 has increased malaria burden; on average across Africa, an estimated 74 excess malaria cases per 100,000 people can be attributed to historical human-caused climate change."

The same issue with "likelihood" applies. Additionally, the 74 excess cases figure appears without its 95% CI of (-411, 599), which is roughly 14x wider than the point estimate and centered barely above zero. A reader might interpret "74 excess cases" as a clear effect, while in fact the underlying estimate is not statistically distinguishable from zero.

Passage 3, Discussion:
"However, anthropogenic climate change has probably increased the burden of malaria in sub-Saharan Africa, and at high elevations and latitudes, will continue to for several more decades."

Most readers will hear "probably" as substantially stronger than a central estimate of 0.07 p.p. with a 95% CI of (-0.41, 0.60), and as implying a Bayesian probability claim that the underlying frequentist analysis does not support.

I want to be clear that I am not asking the authors to suppress these results. Non-significant findings should be reported, and the regional claims for southern Africa and the east African highlands are genuinely better supported than the continental aggregate. My concern is purely about phrasing. I would recommend:

(i) not reporting P+ as a "likelihood." Instead, describe its meaning directly, e.g., "59% of simulations showed an increase." In the quoted passages, another option would be to report central estimates with confidence intervals directly, in place of P+.

(ii) whenever point estimates are given, confidence intervals should also be reported, particularly for the 74 excess cases figure.

(iii) avoiding "probably" and similar adverbs when reporting frequentist results (particularly when they are not statistically significant) as these imply an unwarranted Bayesian interpretation.

These changes would not weaken the paper. The temperature response function and the regional findings remain compelling, and the honest framing would, if anything, strengthen the study's credibility.

(Remarks on code availability)

Open Access This Peer Review File is licensed under a Creative Commons Attribution 4.0 International License, which permits use, sharing, adaptation, distribution and reproduction in any medium or format, as long as you give appropriate credit to the original author(s) and the source, provide a link to the Creative Commons license, and indicate if changes were made.
In cases where reviewers are anonymous, credit should be given to 'Anonymous Referee' and the source.
The images or other third party material in this Peer Review File are included in the article's Creative Commons license, unless indicated otherwise in a credit line to the material. If material is not included in the article's Creative Commons license and your intended use is not permitted by statutory regulation or exceeds the permitted use, you will need to obtain permission directly from the copyright holder.
To view a copy of this license, visit https://creativecommons.org/licenses/by/4.0/

Referee expertise:

Referee #1: Public health, infectious disease modelling

Referee #2: Climate modelling

Referee #3: Public health, infectious disease modelling

Referee #4: Malaria epidemiology, public health in Sub-Saharan Africa, clinician

Referees' comments:

Referee #1 (Remarks to the Author):

This paper is well written and is on a topic that has a long history of research. Unfortunately I do not find this papers conclusions to be convincing and find their analysis to be superficial. They claim to use "state-of-the-art" methods but really are using widely accepted established approaches with old data that has many issues. There are no novel contributions on the methods or data side.

We respectfully disagree with the reviewer on the lack of novelty of our study. **To our knowledge, there is no prior analysis that quantitatively isolates the observed impact of historical anthropogenic climate change on malaria prevalence.** Our paper reuses existing data to fill this gap, and to project future impacts of anthropogenic climate change. We leverage an econometric statistical approach to address confounding variables; our study is the first to apply this method to detection and attribution of climate change impacts on an infectious disease (and is far from "established" in climate epidemiology more broadly).

My first major issue is the authors confounding of population at risk and transmission. Climate and temperature affect the mosquito life cycle in a dynamic manner, and these define a broad area, and hence population at risk, but this is not the same as transmission. The step to transmission is highly non-linear and requires information in vector control, drugs, cultural differences, socioeconomic differences, developmental differences (like infrastructure) and so on. The authors make no attempts to account for any of these and therefore are not contributing much new to this problem. Their analysis on climate scenarios could show the changes in population at risk, something others have done at length, but it is simply not possible with their methods to jump from risk to transmission intensity based only on climate and have a sensible counterfactual or forecast.

We respectfully disagree, and believe that this perspective is both out of date and inaccurate.

First, the statement that we make "no attempt to account for any" non-climate factors is factually incorrect: as we explain in the manuscript and in response to other comments below, the

purpose of the fixed-effects panel regression approach is explicitly to account for spatial and temporal signals contributed by *all* of these factors – better than we would be able to by relying on incomplete or unavailable predictor data on many of these (e.g., there are no continent- and century-wide geospatial datasets available on vector control or 'cultural differences').

Second, the view that temperature does not have a significant effect on transmission, and only determines the broad geographic area where transmission can take place, is now decades out of date and universally rejected. Other studies have demonstrated that mechanistic models of $R_0(T)$ do predict real-world variation in malaria transmission within endemic areas (e.g., Mordecai et al. 2013 *Ecology Letters*; Mordecai *et al.* 2020 *Lancet Planetary Health*). Our study similarly demonstrates a strong empirical relationship between temperature and prevalence; this is our central finding, and essentially disproves the reviewer's *a priori* expectations. Comparable studies on other mosquito-borne diseases have found even stronger effects on transmission than we find here: a recent preprint estimates that one in every five cases of dengue fever worldwide is the result of climate change (Childs et al. *medRxiv* 2024).

Finally, as the first large-scale empirical study of how malaria prevalence responds to climate, our study does in fact 'contribute something new to this problem' - in precisely the way that the reviewer is suggesting is somehow impossible.

Childs, M. L., Lyberger, K., Harris, M., Burke, M., & Mordecai, E. A. (2024). Climate warming is expanding dengue burden in the Americas and Asia. *medRxiv*.

While previously published, the Snow et al dataset is inappropriate for a number of reasons. It is profoundly biased in terms of its sampling and incredibly sparse for what is supposed to cover over 100 years. The authors present no new prevalence data, and do not utilise recent incidence data from DHIS2 or other forms of survey data (e.g. on pregnant women).

We respectfully disagree. The Snow dataset is in fact unique in its breadth and depth; we are not aware of any other single, openly-available prevalence dataset for any infectious disease that covers a century and an entire continent, nor one that has anywhere near the sample size of the Snow data. We also reuse the Snow dataset for our study specifically because the accompanying publication in *Nature* concluded that it was unlikely there were significant long-term impacts of climate change detectable in the dataset. (We are not able to collect new data to supplement the dataset; we are not clinicians or field epidemiologists.)

The climate data is monthly, which is what most people in the field have been using for decades, I would have hoped for some more high resolution data for a paper trying to achieve what the authors want. Also the spatial resolution at 50k is incredibly coarse given most prevalence modelling is done at 5km/10km.

Daily weather data are available, and higher temporal and spatial resolution analysis would have been feasible for a subset of our full 1901-2014 sample years. However, climate data quality across Africa is notably poor, and finely-gridded historical data or models would have

high potential for error, especially in the first half of the 20th century (Donat et al. *BAMS* 2013). More importantly, we conduct our analysis at the monthly level because this is the resolution provided in the Snow dataset. Similarly, our climate data is aggregated to the ADM1 unit, and the underlying granularity of the data pre-aggregation has a minimal influence on the model.

Donat, M. G., Alexander, L. V., Yang, H., Durre, I., Vose, R., & Caesar, J. (2013). Global land-based datasets for monitoring climatic extremes. *Bulletin of the American Meteorological Society*, *94*(7), 997-1006.

The statistical model is far from the "state-of-the-art' methods that the authors claim. Most geostatistical methods (See Peter Diggles work on this) account for both spatial and temporal residuals (or confounding factors as the authors use) through a Gaussian process, which is a more powerful approach than what the authors use. The "causal interpretation" is not causal, its associative, there is no DAG being estimated, and no estimands being inferred (e.g. ATE via targeted maximum likelihood or double machine learning). This is an associative model and the authors should not claim otherwise. Moreover, the g-computation is very superficial, why have they not created an estimand as is done in econometrics or health (e.g. the work of Hernan).

Text edits: It appears there has been a substantial misinterpretation of our statistical model. Our methodology derives from a recent literature in climate econometrics (Hsiang, *Annual Review of Resource Economics* 2016) which has been developed explicitly with the goal of enabling a causal interpretation. As detailed in our methods, the essence of this approach is to use a suite of nonparametric controls to exploit within-location variation in weather over time, which, once accounting flexibly for temporal trends, isolates the components of the climate that occur randomly. We detail this approach in our Methods (lines 427-463):

> “Equation 1 uses a suite of semi-parametric spatiotemporal controls to isolate plausibly random variation in climatological conditions, following standard practices in the climate impacts literature.[47,45] First, α_i is a vector of indicator variables for each of 853 first administrative units (i.e., “ADM1”' units) across our multi-country sample. These spatial “fixed effects” control for all time-invariant characteristics of an administrative unit that may confound the relationship between temperature, rainfall, and prevalence. For example, higher altitude regions may exhibit cooler temperatures, but they also may be composed of lower-income and more geographically isolated communities with limited access to malaria prevention interventions. By controlling for mean conditions in each location, these spatial fixed effects avoid conflating climate conditions with other geographic correlates.
>
> Second, γ_{rm} is a vector of region-by-month-of-year indicator variables, where regions are defined using the Global Burden of Disease (GBD) regional definitions of western, southern, central, and eastern Africa (see Figure 2 in ref. [83]). These spatiotemporal fixed effects account for region-specific seasonality in prevalence that may spuriously relate to seasonally-varying climatological conditions. We allow these seasonal controls to vary by region because of large differences in climatological seasonality and in malaria

cyclicality across sub-Saharan Africa[84], and we show below that our main findings are robust to more stringent seasonality controls defined at the country level (Figure S6). Third, $h_c(.)$ is a nonlinear, country-specific function that controls for country-specific gradual trends that may confound the malaria-climate relationship, particularly under historical conditions of anthropogenic climate change. In our main specification, we model $h_c(.)$ as a quadratic. Figure S6 shows that our results are robust to multiple alternative approaches to controlling for long-run trends that may vary across space. Finally, the indicator variables $1\{intervention\}_{mt}$ and $1\{intervention\ 2\}_{mt}$ are equal to one when an observation falls into the 1955-1969 or 2000-2015 period, respectively. These two periods saw substantial malaria intervention programs across the subcontinent, leading to considerable declines in malaria that were unrelated to changes in the climate[4,85]. These indicator variables control for shocks to prevalence during these two periods, and the coefficients δ_1 and δ_2 allow for differential effectiveness of the two distinct intervention periods. While these variables are highly statistically significant (Table S2), our main findings are robust to their exclusion (Figure S6).

Together, these set of flexible controls imply that the residual variation in temperature and precipitation events used to identify the functions $f(.)$ and $g(.)$ is month-to-month variation over time within the same location, after controlling for gradual country-specific trends, regional seasonality, and the aggregate effects of two substantial malaria prevention intervention programs."

In other words, there is no comparison being made between, e.g., East African highlands and Central African tropics, nor between January and June weather in the same location, comparisons which have many unobservable confounding variables. Instead, the same population is used as both "treatment" and "control" under slightly different weather realizations (e.g., a particularly hot July is compared to a cooler July in the same place, after conditioning on longer-run spatially explicit trends). These same methods have been leveraged to assess some of the most important problems in climate change, published in some of the world's best journals. For example, similar econometric approaches have been used to causally link dengue cases (Childs et al., *medRxiv* 2024), energy use (Rode et al., *Nature* 2021), aggregate economic growth (Burke et al., *Nature* 2015), all-cause mortality (Carleton et al., *Quarterly Journal of Economics* 2022) and human migration (Missirian and Schlenker, *Science* 2017) to the climate, among other outcomes.

With respect to the reviewer's specific concerns: *(i)* this method does flexibly account for spatial and temporal confounding factors *and* residuals (note that these are two distinct things), and we show robustness to alternative approaches to addressing both; *(ii)* under a very explicit set of assumptions – the Gauss-Markov assumptions (Wooldridge, *MIT Press* 2002), which we rigorously evaluate where possible – it can be interpreted as causal, as has been done in much prior econometric-based literature; *(iii)* the model *is* inferring an estimand – specifically, an average treatment effect via maximum likelihood (noting that double machine learning is not appropriate in this setting, as we do not have high-dimensional confounding variables included

as controls); and *(iv)* it is precisely the type of model estimated in econometrics and in particular in econometric analyses of health outcomes (e.g., Childs et al. (*medRxiv* 2024), Deschenes and Greenstone (*American Economic Journal: Applied Economics* 2011), Heutel et al. (*Review of Economics and Statistics* 2021)).

We can substantially rewrite our methods section to ensure these key features of our methodology are articulated in a manner that is easily interpretable by a broader audience outside economics. As other reviewers have pointed out, our notation made the model difficult to interpret and we will use more explicit notation in a revision. We will elaborate on each of the items *(i)*-*(iv)* articulated above to demonstrate clearly the conditions under which the model can be interpreted causally and to explain how spatiotemporal confounding is addressed (including many robustness checks currently in our Appendix that demonstrate the model's insensitivity to alternative characterizations of spatiotemporal processes).

Burke, M., Hsiang, S. M., & Miguel, E. (2015). Global non-linear effect of temperature on economic production. *Nature*, *527*(7577), 235-239.

Carleton, T., Jina, A., Delgado, M., Greenstone, M., Houser, T., Hsiang, S., ... & Zhang, A. T. (2022). Valuing the global mortality consequences of climate change accounting for adaptation costs and benefits. *The Quarterly Journal of Economics*, *137*(4), 2037-2105.

Childs, M. L., Lyberger, K., Harris, M., Burke, M., & Mordecai, E. A. (2024). Climate warming is expanding dengue burden in the Americas and Asia. *medRxiv*.

Deschênes, O., & Greenstone, M. (2011). Climate change, mortality, and adaptation: Evidence from annual fluctuations in weather in the US. *American Economic Journal: Applied Economics*, *3*(4), 152-185.

Heutel, G., Miller, N. H., & Molitor, D. (2021). Adaptation and the mortality effects of temperature across US climate regions. *Review of Economics and Statistics*, *103*(4), 740-753.

Hsiang, S. (2016). Climate econometrics. *Annual Review of Resource Economics*, *8*(1), 43-75.

Missirian, A., & Schlenker, W. (2017). Asylum applications respond to temperature fluctuations. *Science*, *358*(6370), 1610-1614.

Rode, A., Carleton, T., Delgado, M., Greenstone, M., Houser, T., Hsiang, S., ... & Yuan, J. (2021). Estimating a social cost of carbon for global energy consumption. *Nature*, *598*(7880), 308-314.

Wooldridge, J. M. (2010). *Econometric analysis of cross section and panel data*. MIT press.

Given point spatial data, why are the authors using admin1? How are they aggregating to get the admin1 estimates, what weighting are they using (see https://www.ncbi.nlm.nih.gov/pmc/articles/PMC8055469/ for example).

Text edits and analysis: As we explain above, our statistical model relies on comparisons of weather realizations over time for the same spatial unit, in order to isolate randomness in

climate realizations. The point data do not contain observations of the same geolocation for multiple time periods, making such a model infeasible with point-level observations. Thus, we follow the source of the data (Snow et al., *Nature* 2017) in averaging points across each ADM1 unit for each month, which allows us to construct an estimate of ADM1-level prevalence for multiple months over multiple years, making our statistical approach feasible and avoiding classic challenges of confounding variables when comparisons are made across space in estimation (Auffhammer, *Journal of Economic Perspectives* 2018).

We conduct this averaging using an unweighted arithmetic mean over all observations observed in one ADM1-month. This choice was made because it imposes minimal assumptions on spatiotemporal processes and requires no additional high-resolution data on, e.g., population that we would use as weights; these data are impossible to obtain for sub-Saharan Africa for years as early as 1901. Moreover, employing the spatiotemporal smoothing approaches in the work cited by the reviewer would artificially introduce spatial and temporal correlations that would bias recovered coefficients. We note that the work cited by the reviewer aims to construct ADM2 level mortality rate time series, which is a very different goal than uncovering unbiased estimates of the effects of local temperature and precipitation on malaria prevalence.

We can add text to our Methods section detailing the averaging procedure used and the justification for it.

Auffhammer, M. (2018). Quantifying economic damages from climate change. *Journal of Economic Perspectives*, *32*(4), 33-52.

Snow, R. W., Sartorius, B., Kyalo, D., Maina, J., Amratia, P., Mundia, C. W., ... & Noor, A. M. (2017). The prevalence of Plasmodium falciparum in sub-Saharan Africa since 1900. *Nature*, *550*(7677), 515-518.

I am unclear what the likelihood function is and how equation 1 is estimated. Are they accounting for over-dispersion, why are they bootstrapping as opposed to using Bayesian models that are far more common in this area (again see Model Based Geostatistics by Diggle).

Text edits: We estimate Equation 1 using ordinary least squares (OLS), minimizing the sum of squared residuals using the `lfe` package in R. OLS is equivalent to maximum likelihood estimation under the assumption of normally distributed errors, given that our model is linear in the coefficients, with the following likelihood function (Wooldridge, *MIT Press* 2010):

$$L(\sigma, \beta) = \prod_{i=1}^{n} \left(\frac{1}{\sqrt{2\pi\sigma^2}} \right) exp\left(- \frac{(y_i - \hat{y}_i)^2}{2\sigma^2} \right)$$

If helpful for readers, we can explicitly write out this likelihood function and state its equivalence to OLS in the case of the linear model we employ (an equivalence that is proven in many introductory econometrics texts including the one cited above).

Regarding overdispersion, Figure 1 below shows that the regression model residuals are distributed normally. Unbiasedness of coefficients and efficiency of standard errors in our OLS model are therefore not threatened by overdispersion (Wooldridge, *MIT Press* 2010).

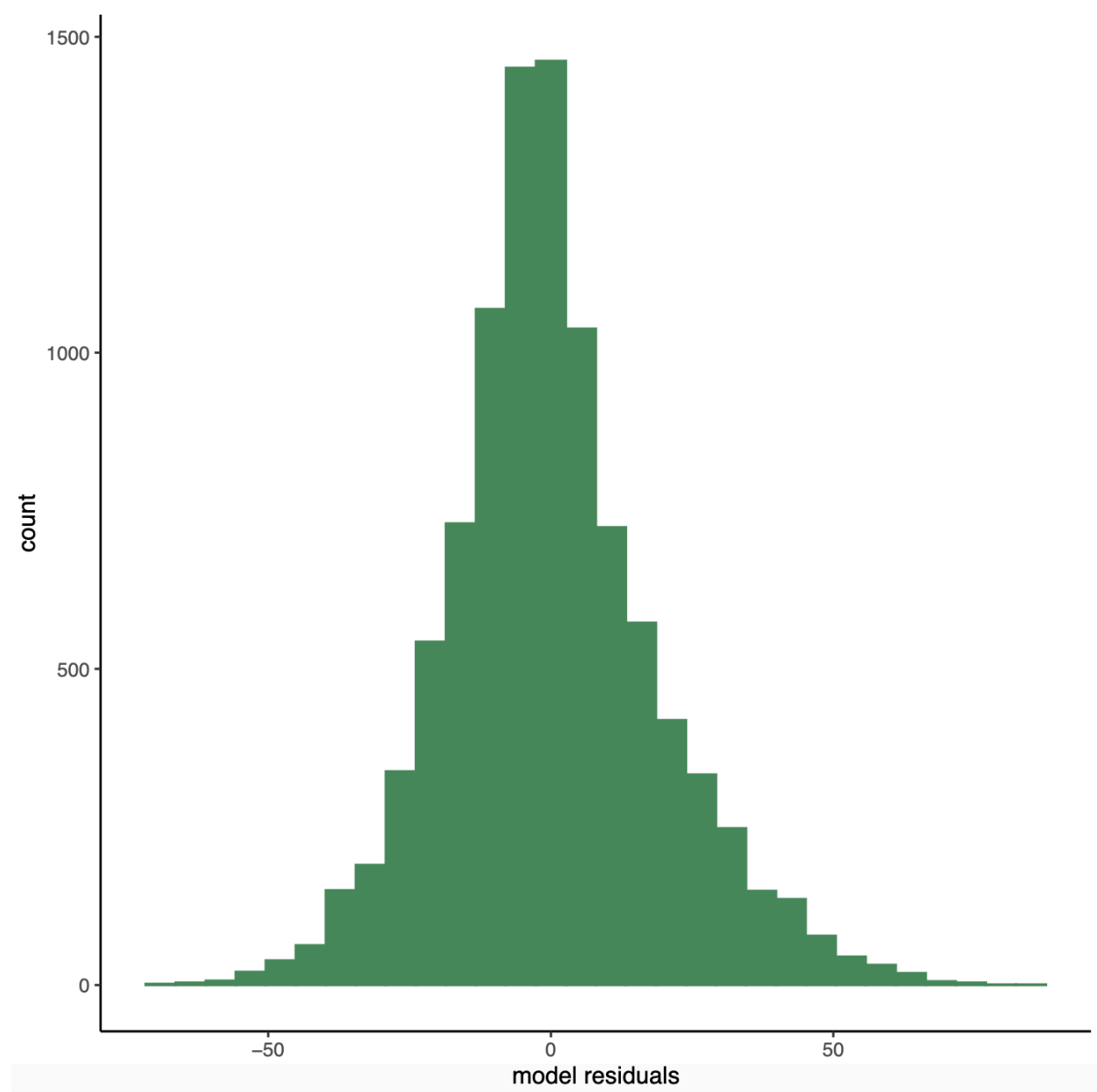

Figure 1: Histogram of regression model residuals.

Bootstrapping is an exceptionally common and theoretically grounded computational method for representing statistical uncertainty. The seminal text on the estimation procedure – Efron and Tibshirani (*Chapman and Hall*, 1994) – has over 54,000 citations. Moreover, this method is very commonly applied in climate impacts and attribution analysis, including, for example: Burke et al. (*Nature*, 2015); Diffenbaugh and Burke (*PNAS* 2019); Heft-Neal et al. (*Nature Sustainability* 2020); Ortiz-Bobea et al. (*Nature Climate Change*, 2021); and many others.

We can add a set of straightforward textual edits clarifying the implementation of our statistical method and its precedence in the climate impacts and attribution literature.

Diffenbaugh, N. S., & Burke, M. (2019). Global warming has increased global economic inequality. *Proceedings of the National Academy of Sciences*, *116*(20), 9808-9813.

Efron, B., & Tibshirani, R. J. (1994). *An introduction to the bootstrap*. Chapman and Hall/CRC.

Heft-Neal, S., Burney, J., Bendavid, E., Voss, K. K., & Burke, M. (2020). Dust pollution from the Sahara and African infant mortality. *Nature Sustainability*, *3*(10), 863-871.

Ortiz-Bobea, A., Ault, T. R., Carrillo, C. M., Chambers, R. G., & Lobell, D. B. (2021). Anthropogenic climate change has slowed global agricultural productivity growth. *Nature Climate Change*, *11*(4), 306-312.

Wooldridge, J. M. (2010). *Econometric analysis of cross section and panel data*. MIT press.

My biggest issue again is what is missing from equation 1, all the factors i described above. The malaria data and methods have existed for a long time, the reason others have not adopted simplistic approaches such as those in this paper is that they are not suitable for the question they are looking to answer.

As articulated above, our statistical method *does* explicitly account for the factors mentioned by the reviewer – e.g., socioeconomic status, infrastructure, vector control, cultural differences – through a set of flexible nonparametric controls in an econometric model that has been widely used to quantify the effects of climate change on many social and economic outcomes.

i am unsure from the paper what are g() and h(), how are the authors accounting for known dynamics of rainfall? for example, rainfall is heavy tailed (https://journals.aps.org/pre/abstract/10.1103/PhysRevE.66.036120) does their monthly mean even make sense in this case? how are these extreme events accounted for in their simple regression model.

Text edits: We apologize that the functions g() and h() were not transparently communicated. Most importantly, g() indicates a set of indicator variables that capture the heavy tails of the rainfall distribution, just as the reviewer suggests. Specifically:

$$g(P_{icm-lt}) = \alpha_1 drought_{icm-2t} + \alpha_2 drought_{icm-1t} + \alpha_3 drought_{icmt} + \delta_1 flood_{icm-2t} + \delta_2 flood_{icm-1t} + \alpha_1 flood_{icmt},$$

where *drought* and *flood* are defined as indicator variables equal to 1 when an ADM1-month experiences total rainfall that is less than the 10th percentile of the location-specific long-run monthly rainfall distribution or greater than the 90th percentile of this distribution, respectively.

We can clarify that monthly precipitation values are sums, not means, and we can write out the functions g() and h() explicitly to ensure that it is clear that rainfall effects are captured via extreme events (droughts and floods).

Minor comments
line 60 - Gething et al Nature also did this analysis and found the same conclusion as Snow, and therefore the authors attempt to dismiss Snow et als conclusion is not warranted.

Our critique is that Snow et al. did not perform a quantitative analysis of the impacts of climate change on malaria transmission before concluding the former could not explain the latter. As we state in the paper (lines 60-64): “More recently, a study by Snow et al. examined the last century of continent-wide changes in malaria prevalence, and concluded that observed trends could not be neatly explained by climate change, but did so based only on visual correspondence between moving averages of rainfall, minimum temperature, and modeled malaria prevalence over the entire continent.”

We believe the study the reviewer is referring to is Gething et al. (2010) "Climate change and the global malaria recession" – which is unfortunately another study that concludes climate change has not influenced malaria prevalence without conducting any statistical analysis. Gething and colleagues map global reductions in malaria transmission, compare them to projections from the literature that climate change would increase R_0 for malaria, and conclude the two are incompatible. This study, which predates the Snow study by seven years, suffers from the same core limitation. In contrast, we address the same topic with a rigorous statistical analysis, demonstrating that climate change has unequivocally increased malaria prevalence in many areas, and that this is compatible with global reductions in malaria transmission.

line 320 - if using the snow data, it is not clear if the authors have also adjusted for diagnostic type (PCR/RDT) or differences in testing such as the infamous DRC 2006 PCR survey.

Analysis: We have re-estimated our model accounting for the diagnostic type of the test. Results are shown in Table 2 below (next page): we find that adding these controls has no effect on our estimates of temperature or precipitation effects on malaria prevalence. Specifically, column (1) shows our main specification, while columns (2) and (3) add controls for the dominant diagnostic method used in each ADM1-month (note that it is rare for more than one method to be used in one ADM1-month). Column (2) uses all diagnostic method categories provided in the Snow et al. (*Nature* 2017) data, while Column (3) simplifies these categories into larger groupings. Coefficients on temperature, flood, and drought are very similar in magnitude and statistical significance across all three columns.

We will add this sensitivity analysis accounting for diagnostic methods to the supplement of the manuscript.

	Dependent variable:		
	PfPR2		
	(1)	(2)	(3)
Avg. temp.	4.15***	3.97***	4.08***
	(1.08)	(1.09)	(1.09)
Avg. temp.^{2}	−0.08***	−0.08***	−0.08***
	(0.02)	(0.02)	(0.02)
Flood	−0.28	−0.06	−0.22
	(0.78)	(0.78)	(0.78)
Flood (lag 1)	−0.45	−0.45	−0.47
	(0.76)	(0.77)	(0.76)
Flood (lag 2)	1.70**	1.81***	1.83***
	(0.68)	(0.67)	(0.67)
Flood (lag 3)	0.96	1.05	0.97
	(0.74)	(0.74)	(0.74)
Drought	0.09	0.18	0.23
	(0.77)	(0.76)	(0.77)
Drought (lag 1)	−1.28	−1.38*	−1.28
	(0.80)	(0.81)	(0.80)
Drought (lag 2)	−1.14*	−1.18*	−1.15*
	(0.68)	(0.68)	(0.68)
Drought (lag 3)	0.19	0.09	0.15
	(0.74)	(0.75)	(0.75)
MICROSCOPY/PCR CONFIRMED		6.13	
		(4.75)	
PCR		27.86***	
		(3.86)	
RDT		2.14	
		(1.35)	
RDT/PCR CONFIRMED		10.29	
		(11.04)	
RDT/SLIDE CONFIRMED		−9.02***	
		(1.34)	
PCR			27.62***
			(3.85)
RDT			−0.60
			(1.12)
Observations	9,874	9,874	9,874
R^2	0.53	0.53	0.53
Adjusted R^2	0.49	0.50	0.50
Note:	*p<0.1; **p<0.05; ***p<0.01		

Table 2: Sensitivity of main specification to controlling for diagnostic method. Column specifications: (1) country-specific quad. trends, intervention year FE, GBOD region-by-month FE; (2) same as (1), but with additional controls for detailed diagnostic method categories; (3) same as (2) but using simplified diagnostic method control categories. In Columns (2) and (3), all diagnostic method coefficients should be interpreted as relative to the Microscopy method.

Snow, R. W., Sartorius, B., Kyalo, D., Maina, J., Amratia, P., Mundia, C. W., ... & Noor, A. M. (2017). The prevalence of Plasmodium falciparum in sub-Saharan Africa since 1900. *Nature*, *550*(7677), 515-518.

line 25 - no uncertainty for these excess cases

Text edits: We can easily add uncertainty for these excess case estimates in the Abstract. We avoided doing so in order to not overburden the reader with many quantitative statements in the Abstract.

Referee #2 (Remarks to the Author):

Summary

The article examines the relationship between climate change and PfPR2−10, the prevalence, of Plasmodium falciparum, a parasite that causes malaria in humans, in children aged 2-10 in sub- Saharan Africa. The authors analyze a dataset of prevalence surveys [Sno+17], which provides a long time span (1900-2014) and subnational spatial resolution, together with the weather station- based climate data CRU-TS. They use panel data regression with fixed effects to identify the influence of temperature and precipitation changes on PfPR2−10. Based on the statistical results, climate model runs are used to attribute historical PfPR2−10 to climate change and to obtain future projections of PfPR2−10 under different climate change scenarios.

The results show a small but statistically significant influence of climate change on PfPR2−10 (Table S2). At the same time, an overall increase in PfPR2−10 due to anthropogenic climate change in the past and in future projections under low and medium greenhouse gas emissions is not significant (Figure 2D). In a more regionally differentiated analysis, the authors find that in some regions rising temperatures reduce PfPR2−10, while in others they increase it.

In my opinion, the topic is relevant, the content seems novel, and many aspects of the analysis appear to be carefully executed. However, further investigation of the validity of the statistical analysis, particularly with respect to uncertainty estimates, is needed to verify the results.

Major Comments

(A) Uncertainties in the results are estimated using a block bootstrap and clustered standard errors. In both cases, the clusters / blocks are the first administrative units, i.e. subnational regions. Thus, temporal correlations are taken into account, but different spatial regions are implicitly assumed to be uncorrelated.

It seems questionable whether this assumption is correct. From the analysis in the manuscript, we see that the analyzed climate variables have only a small influence on PfPR2−10, i.e. there are other factors with a large influence on PfPR2−10. If such a factor causes an increase in PfPR2−10 not only in one region but in several neighboring regions, the observations will be spatially correlated. If this is indeed the case, and the magnitude of this correlation is large, the reported uncertainties in the manuscript could be severely underestimated.

The correlations need to be analyzed to check the validity of the approach used to estimate uncertainties. Note that correlation here means correlations of the errors ε_{icmt} in equation (1) of the article. At least, average correlations (or box plots of correlations) under different clusterings or connections should be reported. Here are some examples of correlations that could be

examined to analyze the dependency structure of the data: Quantify the correlations between ε_{icmt} and $\varepsilon_{i'c'm't'}$ for

- Different regions of the same country: $i \neq i', c = c', m = m', t = t'$;
- regions of the different countries: $i \neq i'$, $c \neq c'$, $m = m'$, $t = t'$;
- close regions $dist(i, i') < 1000km$, $m = m'$, $t = t'$ (with dist() being the distance between the centers of the polygons defining the regions);
- far regions $dist(i, i') \geq 1000km$, $m = m'$, $t = t'$,
- small time lags,e.g,$|m - m'| \leq 3, t = t', i = i'$;
- large time lags,e.g.,$|t - t'| \geq 2, i = i'$;
- same month, different year: $m = m'$, $t \neq t'$, $i = i'$.

These are just examples. With your experience with the data and subject, you may find other structures more relevant to examine.

If, e.g., close regions or different regions of the same country are strongly correlated, the uncertainty estimates (bootstrap, standard errors) have to be adapted using, e.g., different resampling structures for the bootstrap and standard errors with clustering by year, Driscoll Kraay standard errors, or Conley standard errors.

Analysis: We agree with the reviewer that additional sensitivity analysis regarding our measures of uncertainty should be conducted. We can easily implement a set of analyses as articulated by the reviewer to explore whether correlations in our residuals remain and, if so, we can re-estimate our main regression specification using alternative assumptions regarding error structure. Given that we estimate relatively conservative standard errors, we view it as highly unlikely that such analyses would change any of our paper's results.

As an example, we implemented the reviewer's first test – evaluating whether the residuals from our regression model are correlated within countries. Figure 2 below shows a boxplot of residuals for each country. These distributions are centered around zero in nearly all cases; moreover, a regression of residuals on country indicator variables fails to reject a null of zero for every country in our data, indicating there is no correlation across error terms from different regions of the same country.

We can conduct a set of sensitivity analyses like that shown in Figure 2, add them to our Supplement, and adapt our model uncertainty estimates if necessary.

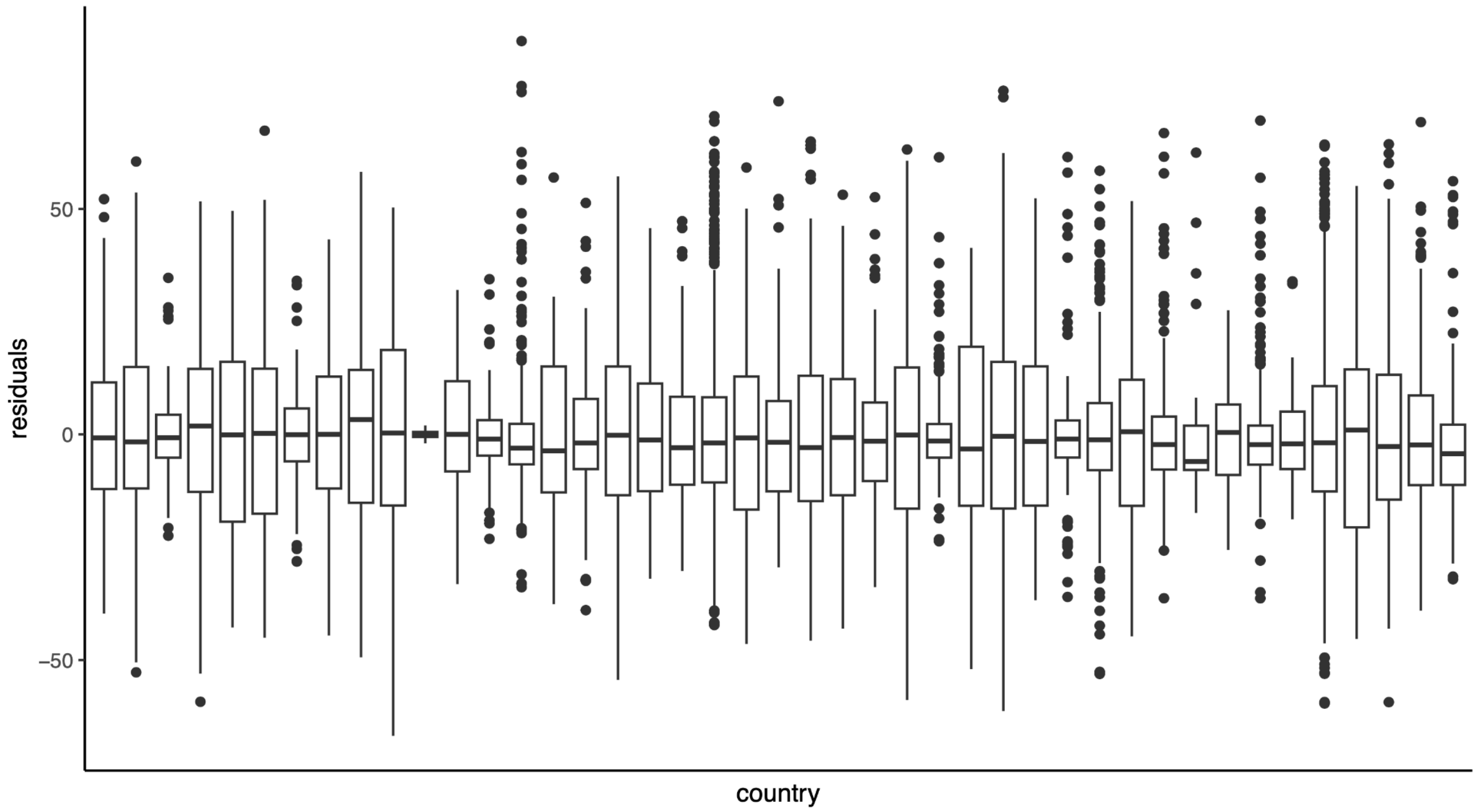

Figure 2: Boxplots of model residuals for each country in the study sample.

(B) The analysis is not easily reproducible. The code for the statistical analysis is publicly available, but the processed input data is not (only the raw data before preprocessing). The processed data that is used as input to the regression should also be made available so that the code can directly be executed without having to invest days to redo the preprocessing.

We agree with the reviewer and can easily add the processed input data to our replication package.

(C) The data is not distributed uniformly over time and space. Furthermore, the data quality between the beginning of the 20th century and the beginning of the 21st century might also change significantly. To investigate whether this is an issue and to further check the robustness of the results, the regression model (main specification) should additionally be applied to certain subsets the full dataset. In particular, fits for data of an early period $t \leq t0$ and a late period $t > t0$ (for an appropriately chosen threshold year t0) would be interesting. For example, one could show a table similar to table S2, with the main model specification and different data restrictions.

Analysis: We agree with the reviewer that such subsampling analysis is interesting and potentially informative of data quality (although it is also important to note that many other things change over time in our sample, including public health interventions that can reshape how prevalence responds to weather). Here, we show an example of the type of additional analysis we could include. Figure 3 shows that the response of prevalence to temperature looks quite similar before (left) versus after (right) the year 1995 (approximately 50% of the sample observations are from before 1995). The temperature at which prevalence peaks is lower in the

first half of the sample, and the impact of warmer temperatures is higher in these early years, although differences cannot be rejected at 95% confidence levels.

We can add results for temporal subsamples of our full dataset to the Supplement, with discussion of implications for model interpretation in the main text.

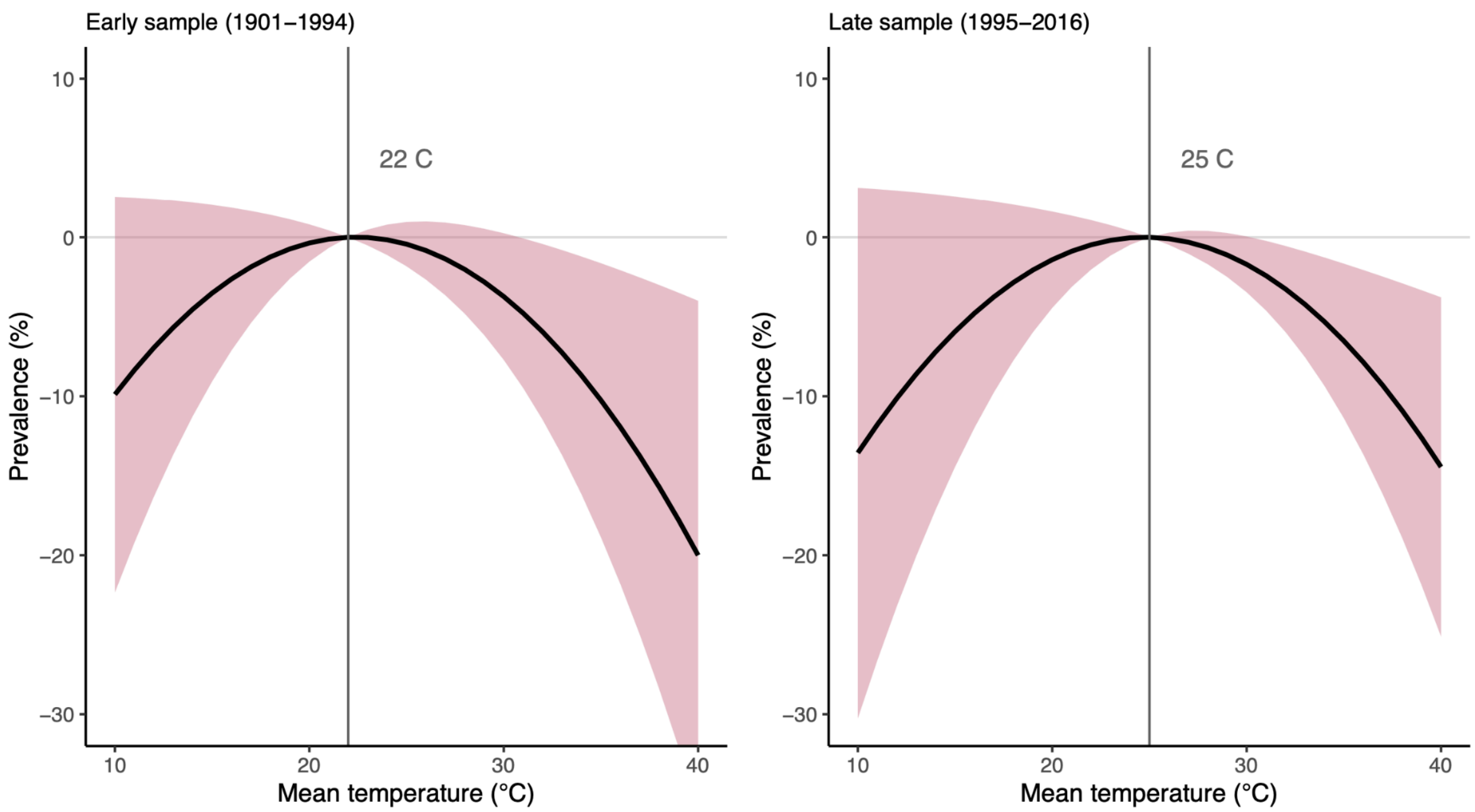

Figure 3: Estimated response of malaria prevalence to temperature in the first half of the sample (before 1995) and in the second half (after 1995).

Minor Comments

(a) line 399 ”as good as randomly assigned”: This is an overstatement. As remarked above, there might be issues with the dependence structure of the data.

Text edits: We can easily adjust our language here to clarify the assumptions under which this claim is true.

(b) Figure 2 A-C: The bootstrap uncertainties are depicted by showing all bootstrap runs. But it is rather difficult to read a quantitative uncertainty value from these plots. For B and C, box plots would be more informative. For A, one could plot, e.g., the 5th and 95th percentile as dashed lines.

Figure edits: We can easily edit the figure display accordingly.

(c) Figure 2D: This plot shows the 5th and 95th percentiles, but the overlapping shaded areas are confusing and it is hard to distinguish which area belongs to which curve. Maybe plot the borders of the shaded areas in thin (dashed?) lines of the same color as the median line.

Figure edits: We can easily edit the figure display accordingly.

(d) Figure 2A: It might be informative to extend Figure 2A similar to [BHM15, Figure 2a] and show a histogram of the observed temperatures below the parabola. The reason is that the parabolic approximation is probably not valid outside (or at the borders of) the observed temperature zone. Furthermore, you could use vertical lines to indicate median temperature (maybe even past and present) of western, southern, central, and eastern Africa.

Figure edits: We can easily edit the figure display accordingly.

(e) line 373: Could you comment on your choice of how you calculate polynomial temperatures? At first glance it seems more intuitive to first aggregate and then calculate the polynomial. In your approach, you start with temperatures that are averages over grid cells, then you make the transformation, and finally average again to the ADM1 region. I.e., the grid resolution seems to have a larger influence in your approach

Text edits: Averaging climatological variables over heterogeneous weather experienced within ADM1 units before computing nonlinear transformations (such as polynomials) would introduce aggregation biases that could threaten the validity of our results. Specifically, doing so would bias the relationship toward a more flat curve, as extreme conditions experienced in some localities are averaged over when aggregating to the ADM1 level (Hsiang, *Annual Review of Resource Economics* 2016). We follow a large climate impacts literature (e.g., Hsiang (*Annual Review of Resource Economics* 2016); Schlenker and Roberts (*PNAS*, 2009); Rode et al. (*Nature*, 2021)) in aggregating the higher-resolution[1] gridded temperature data *after* computing polynomials in order to avoid this bias.

We can add a detailed description of our spatial aggregation procedure and a mathematical depiction of how this approach recovers unbiased estimates of prevalence-weather relationships.

Hsiang, S. (2016). Climate econometrics. *Annual Review of Resource Economics*, *8*(1), 43-75.

Rode, A., Carleton, T., Delgado, M., Greenstone, M., Houser, T., Hsiang, S., ... & Yuan, J. (2021). Estimating a social cost of carbon for global energy consumption. *Nature*, *598*(7880), 308-314.

Schlenker, W., & Roberts, M. J. (2009). Nonlinear temperature effects indicate severe damages to US crop yields under climate change. *Proceedings of the National Academy of sciences*, *106*(37), 15594-15598.

(f) On equation (1):

[1] We use the highest resolution climate data that are available and reliable for our entire sample period, beginning in 1901. These data from the Climatic Research Unit (CRU) are ½ degree resolution and a monthly time step – while higher resolution data would be preferred, limited station data for Africa especially in early years of the sample make this infeasible.

(i) The main specification should be written out in full, i.e., f, g, and hc, should be replaced by what you actually use. Either completely replace Equation (1) by the explicit main specification or have the main specification additionally to the more general form (which you could then also put into the section Statistical model sensitivity and robustness).

Text edits: We can write out Equation 1 in full for clarity.

(ii) You often have four indices for a variable, e.g., Picmt. For the lagged effects, you subtract l from the third variable, Picm−lt. This is hard to read. Maybe add commas: Pi,c,m,t and Pi,c,m−l,t.

Text edits: We can add commas to the subscripts of Equation 1.

(iii) You write gl. But from the description, I seems like g does not depend on l.

Text edits: *g()* does depend on lag length *l* because coefficients on drought and flood variables will differ for each lag length. This will be clear when we write Equation 1 out in full, as the reviewer suggests. **We can write Equation 1 in full for clarity.**

(iv) The interventions are indexed by mt. But from the description, I seems like they only depend on the year t.

Text edits: We can change the subscript to include year only.

(v) The index i determines the ADM1 region and, by that, also the country. So, for all variables which have an index i, you can remove the index c.

Text edits: In these cases, we can change the subscript to include *i* only.

(vi) Consider using t as month-year time index (and keep m as month index only for γrm). Then you can write PfPRi,t = f(Ti,t) + ..., which seems cleaner to me as it better represents the spatio-temporal panel structure of your data.

Text edits: We can change the time indexing following this suggestion.

(g) In your statistical model, previous times only influence the current PfPR2−10 through flood and drought events and you also test robustness against lagged temperature effects (dynamic temperature effects). But from a naive population dynamics perspective, it seems plausible that the prevalence of the previous month(s) could be a strong predictor for the prevalence of the current month. Did you consider such an autoregressive model, i.e. PfPRi,c,m,t = PfPRi,c,m−1,t +...?

We appreciate this suggestion and did indeed consider conducting such an analysis. However, there are two key limitations. The first, and more important, is data availability: lag effects can only be calculated when an ADM1 unit has data in adjacent months, which is not common in most years of the dataset. Using an autoregressive model would force us to drop 7,648 of 9,875

observations (~77% loss of data), significantly reducing our statistical power. Second, autoregressive models are biased when spatial fixed effects are used (Nickell, *Econometrica* 1981), threatening the validity of our regression coefficients. We are ultimately comfortable not including an autoregressive component given these data limitations, and the fact that we do not aim to predict overall prevalence or include these predictions in our findings.

Nickell, S. (1981). Biases in dynamic models with fixed effects. *Econometrica: Journal of the econometric society*, 1417-1426.

Referee #2 (Remarks on code availability):

The code for the statistical analysis is available. But reproduction seems laborious as only raw data can be downloaded but not pre-processed data, which is the input to the statistical analysis. (Or the description is lacking, so that I was not able to find it).

We can easily distribute the pre-processed data as part of the replication package.

Referee #3 (Remarks to the Author):

This paper is an ambitious analysis of a large data set on the important question of climate change attribution of malaria burden in Sub-Saharan Africa (SSA). The authors analyze a recently published data set on Plasmodium falciparum clinical prevalence for the last century and for SSA which encompasses most of the global disease burden. The analysis combines a statistical model with observational and simulated climate variables for temperature and precipitation, to consider attribution of change up to 2015 and in simulated future scenarios. Main conclusions are that climate change has had a historical impact, which is regionally differentiated and most evident in East African highlands and South Africa, but small when measured as relative percent change and in comparison to previous reported assessment of intervention effects. Furthermore, future climate change should decrease malaria prevalence in West Africa and only moderately continue to increase it in East and South Africa.

(I am not a climate modeling expert and will therefore only comment on the attribution analysis).

My main concern is whether the question posed here can be addressed in a meaningful way with the malaria survey data, given the spatial and temporal averaging over large areas and across multiple years and transmission seasons. This issue is not convincingly addressed. We are told that the spatial "aggregation scale is supported by previous work that models this data set at the same spatial resolution". This is not a sufficient argument as the goal of the other study was different, and so were the models. On space, one would want to consider what is the degree of variation in temperature over the areas considered for the main transmission seasons. For example, in regions like the East African highlands the analysis may be smoothing considerable variation in both monthly temperature and malaria prevalence. Perhaps there is no way to do better given the spatial extent that can be considered and the nature of the survey data. But there is no consideration of how the averaging can reduce climate effects. This is a difficult question of course because the global data set itself that is used for the observational climate has also a coarse resolution relative to changes in malaria transmission dynamics in some of these regions.

Text edits: We fully agree with the reviewer that naive averaging of temperature over the ADM1 units in our sample would introduce aggregation biases that could threaten the validity of our results, as substantial heterogeneity in weather is realized across these administrative units. As noted in lines 373-376 in the original manuscript, we follow a large climate impacts literature (e.g., Hsiang (*Annual Review of Resource Economics* 2016); Schlenker and Roberts (*PNAS*, 2009); Rode et al. (*Nature*, 2021)) in carefully constructing the temperature variables used in our statistical models to avoid such aggregation biases.

Specifically, we acknowledge that the nonlinear relationship between malaria prevalence and temperature is *local* – for example, heat shocks in high-elevation areas of an ADM1 may increase prevalence there at the same time that cooler temperatures elevate prevalence in lowland coastal regions of the same ADM1. In order to recover this local relationship with more

aggregate data, we aggregate the higher-resolution[2] gridded temperature data to the ADM1-month level in a way that reflects temperature extremes experienced in individual grid cells within an ADM1 during each month within a year. To do so, we compute nonlinear transformations of temperature (e.g., polynomials of average temperature) at the grid-cell-by-month level before averaging values across grid cells in an ADM1. As has been articulated previously (e.g., see algebra in Appendix A.2.4 in Rode et al. (*Nature* 2021) and Equation 29 in Hsiang (*Annual Review of Resource Economics* 2016)), this method allows us to recover the local-level nonlinear relationships between prevalence and temperature even when the regression is estimated at the ADM1 level. In contrast, if we were to average over temperature conditions across an ADM1 *before* computing nonlinear transformations of weather variables, we would indeed estimate a biased set of parameters, as the reviewer indicates.

We can add a detailed description of our spatial aggregation procedure and reproduce prior mathematical depictions of how this approach recovers unbiased estimates of local-level relationships even when estimated on spatially aggregated data.

Similarly, when computing the % increase or decrease attributable to climate change, the monthly malaria prevalence is averaged across months and therefore seasons, and over a window of years, for the baseline and the most recent period respectively. The locations where climate variables are expected to have the strongest effects will be necessarily epidemic and seasonal. Isn't this approach averaging peaks and throughs in a way that washes out the interesting signal? Wouldn't the % change be larger if considered only for the months of the transmission season in that location? Asynchrony of seasonality adds a further potential source of smoothing of the signal. One could argue that it is valid to ask the question of attribution at any scale of choice, but I am not clear that this is so and independent from the spatial and temporal scales of malaria population dynamics and temperature variation.

On the point that we might recover more dramatic estimates by focusing on the window during which transmission takes place: this unfortunately introduces a complex tautology into the analysis. For example, we could use $R_0(T)$ to estimate months that are "unsuitable for transmission" (below T_{min} or above T_{max}) and exclude them from the analysis. However, these would be derived independently, and - as some of the pioneers of using this method at the monthly level - we have become, over time, convinced that this method is largely inappropriate. This study, in particular, demonstrates that the relationship between climate and transmission is more gradual, and our aggregated estimates capture the impacts of both daily (and finer-scale) variability, and within-areal unit variability, in transmission-climate relationships. **We can, however, add a disclaimer that our analysis cannot distinguish parts of the year where transmission did not occur unless this is reflected in the primary data.**

[2] We use the highest resolution climate data that are available and reliable for our entire sample period, beginning in 1901. These data from the Climatic Research Unit (CRU) are ½ degree resolution and a monthly time step – while higher resolution data would be preferred, limited station data for Africa especially in early years of the sample make this infeasible.

On the suggestion to visualize the full set of predictions, rather than summarize by year: because our focus is on the long-run impacts of climate change, we have not designed visuals or summary statistics around changes in month-to-month variability. We could certainly visualize our predictions at the monthly level, so that intra-annual variability would be visible; however, doing so in the same visual as our key findings would reduce the interpretability of the trends – analogous to, e.g., why climate scientists have moved away from showing monthly temperature data. We can revisit this visualization of our results in a revision, but would like to focus main text figures on the climate change-driven, long-term component of trends.

We can add supplementary figures displaying monthly variation in malaria prevalence due to anthropogenic climate change, as well as a short discussion of these month-to-month changes.

Is it possible to focus on particular regions and address whether the survey data allows higher resolution, and/or whether the topography and associated variation of climate parameters justifies the spatial aggregation in that location? CRU data may not allow an answer. I agree with the authors that attribution remains "challenging for the downstream impacts of anthropogenic climate change" including in epidemiology. While the authors do formulate the statistical model in ways that should account for effects other than climate, this is done within the limitation of granularity. I am not convinced the question itself is meaningful at the studied granularity, and would have liked to see either convincing discussion of the limitations, or some analysis of the effects of averaging.

Text edits: See response above regarding averaging; our method does enable us to recover grid-cell level local relationships, although it is estimated with ADM1-level data. We do not assume homogeneity of weather conditions across heterogeneous ADM1 units.

This comment justifies the same textual edits as articulated above for spatial aggregation.

Other comments:

1) A country-specific quadratic time trend is included in the model. What is the justification for this functional form? Or at least its interpretation?

We include these trends in order to control for possibly nonlinear country-specific long-run trends in prevalence that could be correlated with spatially varying long-run trends in precipitation and/or temperature. The functional form is a parsimonious means of capturing long-run trending behavior that could introduce spurious correlation; however, we show in Appendix Figure S6 that our main results are highly robust to alternative (and more flexible) temporal controls.

2) What criterium was used to select the final form among the possibilities?

Text edits and analysis: The final form was chosen based on the statistical significance of increasingly restrictive spatiotemporal controls, alongside attempts to maximize degrees of freedom while controlling for key plausible confounders. For example, Global Burden of Disease (GBOD) region interactions with month fixed effects were highly statistically significant, indicating that controlling for regional heterogeneity in prevalence seasonality is important to avoid spurious correlations between temperature and prevalence that could arise due to aligned seasonal cycles (this is discussed in more detail in response to the following comment). In contrast, country-month indicators were statistically underpowered, as the median number of observations per country-month is just 11. Similarly, country trends in prevalence were observed to be nonlinear and to statistically differ from average trends within a GBOD region, indicating that country-specific heterogeneity in long-run temporal controls were necessary to fully address the possibility that spatially heterogeneous gradual trends in prevalence coincided with spatially heterogeneous gradual climate change.

We can add text to the methods, supported by additional figures and tables in the Supplement, to detail how we selected the final functional form.

3) What is regional seasonality meant to capture?

Global Burden of Disease (GBOD) region-by-month fixed effects control for average seasonal patterns in malaria prevalence in different (broad) parts of the continent. If prevalence is seasonal for reasons unrelated to temperature or precipitation patterns – for example, different regional seasonality of vector biology or human behavior – this could confound empirical estimates of the effects of temperature and precipitation. We allow these seasonal controls to vary across regions, reflecting the fact that month-specific seasonality in prevalence and in climatological conditions differs across regions (as mentioned above).

4) We are told in several places that Figure S6 shows that other specifications of the model do not make a difference. I am not clear on how one should consider the evidence in this figure to reach that conclusion. While the form of the curves is similar, the maximum does move and the uncertainty at the extremes also varies considerably among the panels.

Text and figure edits: Of course, changing spatiotemporal controls can affect the recovered point estimates of the effects of temperature and precipitation on prevalence. This is particularly the case with extremely restrictive specifications, like those that allow each country to have its own monthly fixed effect (i.e., country-specific seasonal controls) in addition to regional year fixed effects (i.e., controls for regionally heterogeneous annual shocks). Thus, we selected the main specification using criteria described above. However, we also show robustness to alternative sets of controls to demonstrate that key results (namely, the overall effect of climate change, which is determined mostly by peak temperature) do not hinge critically on our selection. We note that other figures and tables additionally show robustness, not only Figure S6, including Figures S7-S12 and Table S2. We reach the conclusion that the prevalence-temperature relationship is robust to alternative specifications because the recovered 95% confidence interval from the main specification contains the point estimate of

other specifications estimated using alternative spatiotemporal controls (see Figure 4 below). Throughout the paper, we clearly communicate that drought and flood results are more uncertain in the main specification and exhibit more variability across specifications (e.g., see Table S2).

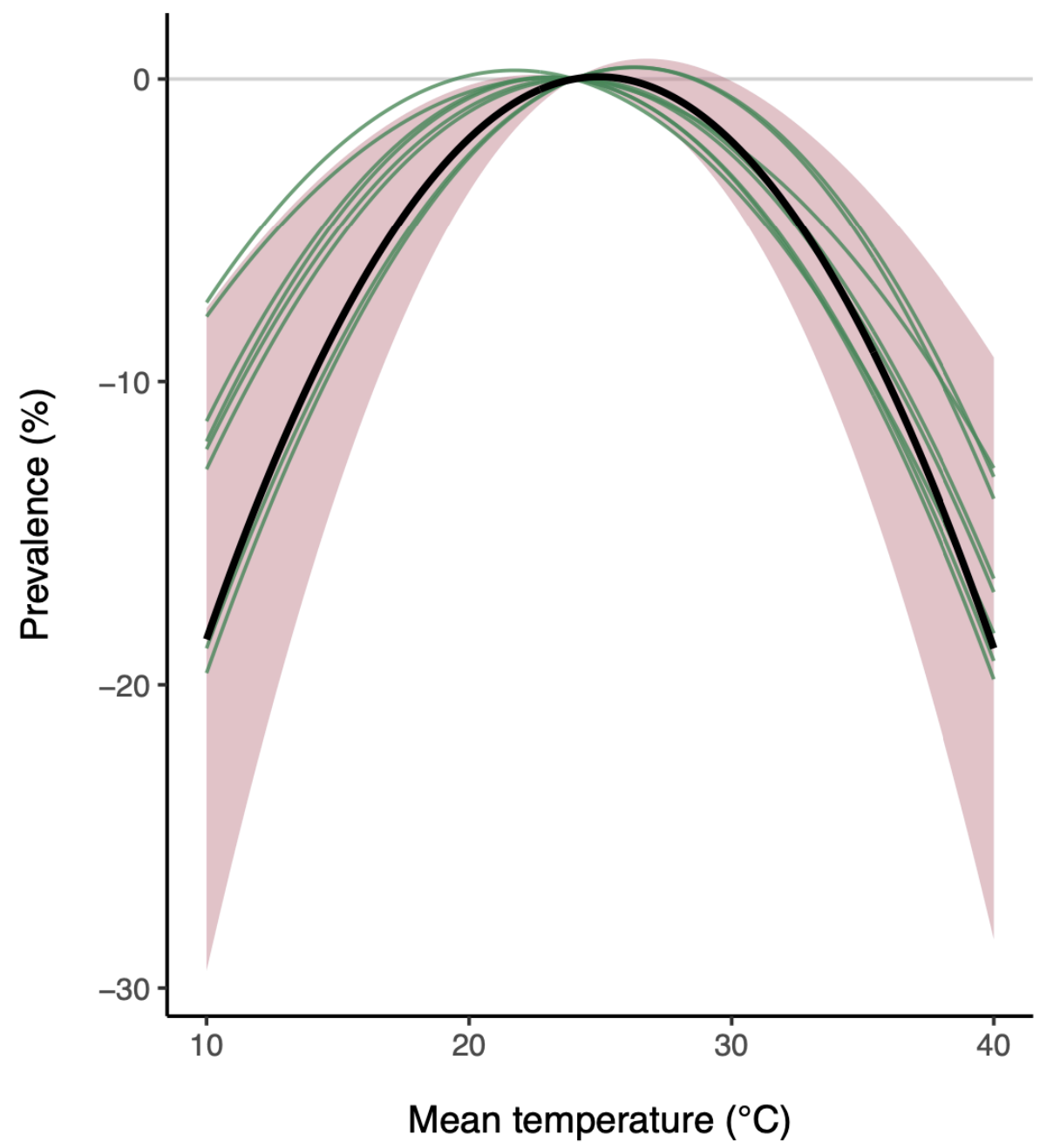

Figure 4: Estimated response of malaria prevalence to temperature using the main specification (black lines, with pink shaded 95% confidence intervals) and using alternative spatiotemporal controls (green lines). Alternative specifications shown in green include all models listed in Table S2 in the manuscript.

We can add the figure above to our Supplement and add text to the main manuscript explaining the evidence for a robust prevalence-temperature relationship in more detail.

Finally, as a point of clarification, we note that unlike $R_0(T)$ models common in the literature, there is no "maximum" estimated to this relationship (no point at which the curve is truncated).

5) It would be valuable to include some Discussion of malaria control in the context of climate change, decadal and interannual variability, instead of emphasizing the "climate vs. control" view.

Text edits: We appreciate this suggestion, and could focus more of the Discussion on (1) the fact that national and subnational trends will probably continue to be mostly driven by malaria control and mobility (very different from e.g., dengue fever), suggesting that the best climate-resilient malaria eradication strategy is to proceed "full steam ahead," focusing efforts based on surveillance data; and (2) the possibility that the cost of malaria control and eradication programs will increase regionally, particularly in east Africa, in the coming decades.

6) Also, some discussion of the temporal scales of variation that are not considered here, specifically for effects of climate variability, given that climate change will alter decadal and interannual variation as well as extreme events. These are the time scales at which climate will most directly impact public health. Given the approach, this kind of variation is not taken into account here.

Text and figure edits: Our method accounts for changes in variability of monthly weather, but cannot resolve any changes in daily variability within months. To the extent that climate change is altering daily extremes conditional on monthly average conditions, these changes would not be captured by our results. This is the only timescale of variation we cannot capture; any changes in seasonality, other intra-annual variability in weather, or longer-run changes in climate are captured. Because reliable daily climate data for Africa dating back to 1901 is not available, we cannot change this feature of our work. As discussed above, we can visually display monthly variability in climate change projections in supplementary figures if desired.

We can add text explaining which forms of changing climate variability (i.e., changes in the variance of daily weather within a month) are not captured by our results. As discussed above, we can add supplementary figures showing changes in month-to-month prevalence variability due to anthropogenic climate change.

Referee #4 (Remarks to the Author):

As the authors point out, consideration of the likely impact of climate change on malaria incidence has generated diverse views, often because broad conclusions have been drawn from data collected in a limited area and/or over only a limited period. Thus, the study described in this paper, the most comprehensive attempt to address this issue, that makes use of the unique data set collected by Prof Snow and his colleagues is important and will be of great interest to the malaria community and to others interested in the potential impact of climate change on other vector borne diseases.

The overall conclusions of the study on the likely future impact of climate change on malaria in sub-Saharan Africa are in agreement with the overall perceptions of most malaria epidemiologists with broad experience of sub-Saharan Africa, namely that there will be an increase in incidence of malaria in some areas of East and Southern Africa, especially mountainous areas, and a reduction in incidence in the already hot and dry areas or West Africa. However, it is very helpful to have these suppositions supported by detailed modelling based on results from a very large data set that has been comprehensively analysed, including the analyses of the relative importance of changes in temperature or changes in rainfall.

This reviewer is not qualified to comment on the details of the methods used for the climate science, statistical and modelling approaches that have been used. However, these are clearly described in the methods section of the paper and thus accessible to other reviewers better qualified to comment on these issues. My comments and suggestions are restricted primarily to issues that might be raised by those involved in the more practical aspects of malaria control and how the findings of this study have relevance to their work.

a. Parasite prevalence and age. The prevalence of malaria parasitaemia in children aged 2-10 years has been used widely in epidemiological studies as a measure of the intensity of malaria transmission in a community because in most parts of sub-Saharan Africa a high proportion of infections occur in children in this age group. However, as the intensity of infection declines or increases, the proportion of all infections in a community in those aged 2-10 years will increase or decline but the overall incidence in the community may remain unchanged. This is a potential risk in using the 2-10 age group as the variable of interest and this could be mentioned.

Text edits: We appreciate this point, and agree that our manuscript could better address this issue. Throughout (e.g., in the title), we have tried to be clear that our data and analysis only addresses childhood malaria. This population experiences the majority of mortality in sub-Saharan Africa, and reductions in parasite prevalence in children do correspond to overall progress towards elimination in the general population. However, the reviewer is correct that compensatory dynamics can emerge from immunity: specifically, high prevalence in children can produce clinical immunity in adults, creating a “see-saw” dynamic between the two. When prevalence decreases from hyperendemic to endemic levels, the loss of childhood-acquired immunity can sometimes lead to greater transmission within the adult population. Theory suggests that this is more likely to happen in the early stages of eradication and it is unlikely that

this would be a fully compensatory shift (i.e., reductions in childhood malaria should correspond to overall reductions in prevalence most of the time, even if incidence in adults increases). Similarly, in the places where climate change has increased malaria prevalence, these increases are so far mostly contained within broader trends of decreasing overall prevalence - and so it is unlikely that there would be a major reduction in adult cases as a result.

Overall, we would suggest that the primary effect of age-structured transmission dynamics is probably not to undermine the overall accuracy of our findings, but to create a secondary effect of note: in hyperendemic parts of central Africa, it is possible that the combined negative effect of elimination efforts and climate change on childhood malaria would be accompanied by some increases in incidence in adults, meaning that climate change might still be having negative effects within populations where it is “helping” eradication efforts move towards their targets.

We will add a discussion of climate change impacts on age-structured transmission dynamics to our Discussion.

b. Parasite prevalence and clinical malaria. As mentioned above, parasite prevalence in children aged 2-10 years has proved a useful marker for epidemiological studies but the variable of most interest to the malaria control community is the number of cases. The parasite prevalence in children aged 2-10 years is used as the indicator for most of the analyses described in the paper but on page 6 changes in numbers of cases are mentioned. How were these numbers generated – was this an extrapolation from prevalence and, if so, how was this done?

Text edits: We are still reporting prevalence, only rescaled, not incidence – i.e., cases per 100,000 children is percent prevalence multiplied by 1,000. Note that we never claim to model total case numbers, which would be a substantially more complicated endeavor.

We will add a sentence to our methods clarifying how we convert prevalence to cases per 100,000, or – following reviewer advice – remove scaled prevalence estimates.

c. Urbanization. Urbanization is likely to continue to have an increasingly important impact on the malaria burden in the coming decades. Movement of rural residents to cities which have little malaria transmission, as is happening in many parts of sub-Saharan Africa, sometimes as a result of climate change, can have a major impact on the parasite rate of a district or regional population. In the past, it could be assumed that moving to a city would reduce the chance of a malaria infection but, as the authors mention in the discussion, the worrying emergence of Anopheles stephensi in African cities may change this assumption. Was urbanization taken into account in the climate change predictions in some way?

Text and analysis edits: This is a fantastic question. Like other sources of spatial heterogeneity, urbanization is currently accounted for through spatial and temporal controls. This partially accounts for different mosquito ecology in urban and rural settings; however, they do not allow for the possibility that urbanization is also a mediator on the prevalence-climate relationship. We cannot explicitly solve this problem for our entire analysis, because (1)

addressing urbanization more explicitly would require us to also develop a more explicit framework for handling population, and untangling the tightly correlated relationship between the two; and (2) historical estimates of population and land cover dating back to 1901 are not reliably available. However, **we could add a supplementary analysis of climate-urbanization interactions using data from Africapolis, dating back to 1950 and projected forward until 2050.** We can also add text to the main manuscript articulating that urbanization may shape how malaria responds to climate change in unforeseen ways, and that our analysis only partially accounts for this possibility, due to data limitations. (We already address this to some degree in our discussion of how Anopheles stephensi may change future dynamics.)

d. Heterogeneity of effect. It is helpful in the analyses undertaken to characterize the major ecologically different regions of sub-S[a]haran with different climate change impacts predicted across a wide region. However, the intensity of malaria transmission can vary substantially over a small area depending on local characteristics, which side of a mountain one lives on, how near a river etc. so that climate change may have a very different impact on communities that are living quite near to each other, and it might be helpful to make this point in the discussion.

Text edits and analysis: We fully agree with the reviewer that there is likely to be heterogeneity in the impacts of climate change that we do not fully resolve in our ADM1-level analysis. **We can easily point this out in the discussion, as suggested by the reviewer.**

If desired, **we can also conduct an additional set of historical and future climate change impact estimates at the native resolution of our analysis – the ½ degree grid cells derived from CRU climate data.** This higher resolution will provide a window into some of the heterogeneity suggested by the reviewer. As noted in lines 373-376 in the original manuscript, we follow a large climate impacts literature (Hsiang (*Annual Review of Resource Economics* 2016); Schlenker and Roberts (*PNAS*, 2009); Rode et al. (*Nature*, 2021)) in carefully constructing the weather variables used in our statistical models so that we can empirically recover local-level nonlinear relationships between prevalence, temperature, and precipitation even when the regression is estimated at the ADM1 level. This enables us to use the recovered regression coefficients to predict prevalence changes at the grid cell level. Of course, ½ degree resolution is still insufficient to reveal some of the differences across space mentioned by the reviewer, and thus additional text discussing these limitations will remain necessary.

e.Magnitude of the climate change effects. Overall, the expected changes in parasite prevalence, although statistically significant and important, are relatively modest in relation to the changes that are likely to occur as a consequence of other factors. For example deployment of a highly effective malaria vaccine could reduce malaria prevalence in 2-10 from 50% to 5% whilst invasion of many large African cities by An. stephensi could have a major deleterious effect. It would be helpful to stress this it in the discussion for the benefit of the general reader so that the relatively modest, although important, changes likely to be induced by climate change can be put into a broader perspective of what is likely to happen to the incidence of malaria in sub-Saharan Africa in the coming decades. It is important that the predicted changes

in malaria incidence are presented in an appropriate way in general discussions on the need for mitigation of climate change.

Text edits: We fully agree with the reviewer. In the current manuscript, we aim to communicate exactly this sentiment at the end of the Results section, where we write (lines 177-188):

> "While these effects are meaningful, we caution that they are also far smaller than the reduction achieved through healthcare, mosquito nets, vector control, and economic development: previous work with the same dataset has estimated a reduction since 1900 of 16 *p.p.* (that is, a continent-wide decline in average $Pf\mathrm{PR}_{2\text{-}10}$ from 40% in 1900-1929 to 24% by 2010-2015[4]), while our estimates of historical climate change-attributable changes rarely exceed 1.5 *p.p.* for any individual administrative region. Additionally, we estimate that average reductions in prevalence realized during the Global Malaria Eradication Program (1955-1969; estimated reduction averaged over the entire period: -4.80 *p.p.*) and recent programs like Roll Back Malaria and the Global Technical Strategy (2000--2014; estimated reduction averaged over the entire period: -3.35 *p.p.*) were substantially larger than the cumulative effects of anthropogenic climate change (Table S2)."

Additionally, in the Discussion we write (lines 259-265):

> Spanning multiple centuries, our analysis is the most comprehensive look to date at the impact of climate change on any infectious disease, and brings new clarity to a decades-long debate in malaria research. Whereas some work has questioned the plausibility that overall declines in continent-wide prevalence would conceal a climate-linked increase[4,19], the 0.087 percentage point increase in $Pf\mathrm{PR}_{2\text{-}10}$ that we attribute to historical anthropogenic climate change could easily be masked by the nearly 200-fold greater overall reduction observed across sub-Saharan Africa over the same period.

We can add to our Discussion additional details regarding how our estimates quantitatively compare to other plausible future drivers of change in malaria prevalence across Africa, including new developments in population-wide vaccination that have happened largely during the preparation and review of our study.

Summary of Revisions and Responses

We thank the four reviewers for their comprehensive feedback on our manuscript.

In addition to smaller general improvements in text and figure clarity, our revision has focused on four major areas of improvement:

1. **Comprehensive uncertainty analysis (Response to Reviewer 2).** Reviewer 2 is primarily concerned with our treatment of uncertainty. Following the reviewer's specific guidance, we conduct a new evaluation of spatial and temporal correlations in model residuals to justify our preferred approach to quantifying statistical uncertainty. We additionally show that our key results are unchanged when we implement alternative methods of uncertainty estimation recommended by Reviewer 2.
2. **Spatial and temporal disaggregation (Response to Reviewers 1 and 3).** Both Reviewers 1 and 3 are concerned with how our model aggregates variables over space and time. We provide a new Methods section detailing our aggregation methodology, and how it is designed precisely to remove aggregation bias, based on prior statistical work. We additionally conduct two new analyses that (i) remove spatial aggregation entirely; and (ii) disaggregate annual results into seasonal predictions, showing our model's ability to represent variability at many spatial and temporal scales.
3. **Multiple additional sensitivity analyses (Response to Reviews 1-4).** Based on reviewer suggestions, we have incorporated several new facets to our analysis including stratifying climate effects in urban versus rural communities; confirming that our results are not biased by long-term changes in the diagnostic method used to detect malaria; and evaluating how climate change impacts are distributed over the course of the year, which notably implicates expanding seasonality as a mechanism of southward geographic expansion. These add several important facets to the story our paper tells, while further underscoring the robustness of our main results.
4. **Updated description and justification of key statistical methodology (Response to Reviewer 1).** Reviewer 1's concerns revolve around a clear justification for the statistical methodology. In response, we have extensively revised the statistical model description (Equation 4, formerly Equation 1), updating its notation and ensuring the method's ability to enable causal interpretation while controlling for confounders is clear to a broad audience. We clarify that our study builds on the same methodological foundation as many leading climate impacts papers, such as the best observational studies to date of climate change impacts on dengue in PNAS (Childs et al., 2025), all-cause mortality in Science Advances (Gould et al., 2025) and human migration in Science (Missirian and Schlenker, 2017).

Referee #1 (Remarks to the Author):

This paper is well written and is on a topic that has a long history of research. Unfortunately I do not find this papers conclusions to be convincing and find their analysis to be superficial. They claim to use "state-of-the-art" methods but really are using widely accepted established approaches with old data that has many issues. There are no novel contributions on the methods or data side.

We respectfully disagree with the reviewer on the novelty of our study, and in our revision, we better clarify the significance of the work for a broad audience of readers. As we detail below, these changes include edits in the: description of our statistical model (Methods lines 514-622); description of the statistical results (lines 140-165); and discussion of malaria control in the context of climate change (lines 314-328). **To our knowledge, there is no prior analysis that quantifies the observed impact of anthropogenic climate change on malaria prevalence–let alone at a continental scale. Our paper uses existing data to fill this gap, and as an added contribution, projects *future* impacts of anthropogenic climate change under different emissions scenarios.** We leverage an econometric statistical approach to address confounding variables; our preprint was the first to apply this method to detection and attribution of climate change impacts on an infectious disease, and – out of the small number of studies who have followed our precedent – remains the only application of these methods to malaria, the single deadliest infectious disease in low-income countries.

My first major issue is the authors confounding of population at risk and transmission. Climate and temperature affect the mosquito life cycle in a dynamic manner, and these define a broad area, and hence population at risk, but this is not the same as transmission. The step to transmission is highly non-linear and requires information in vector control, drugs, cultural differences, socioeconomic differences, developmental differences (like infrastructure) and so on. The authors make no attempts to account for any of these and therefore are not contributing much new to this problem. Their analysis on climate scenarios could show the changes in population at risk, something others have done at length, but it is simply not possible with their methods to jump from risk to transmission intensity based only on climate and have a sensible counterfactual or forecast.

We respectfully disagree, and believe this hesitation is arising due to a misunderstanding of our methods, which we have worked to rectify through improvements to the manuscript. We summarize these possible sources of misunderstanding here, and we detail methodological and textual updates in response to the reviewer's specific statistical comments below.

First, the statement that we make "no attempt to account for any" non-climate factors is factually incorrect: as we explain in the manuscript and in response to other comments below, the purpose of the fixed-effects panel regression approach is *explicitly* to flexibly account for spatial and temporal signals contributed by *all* of these other factors when estimating the effect of climate variables on transmission. This flexibility and lack of parametric structure enables us to control for these factors more comprehensively than we would be able to by relying on

incomplete or unavailable predictor data on many of these other factors (e.g., there are no continent- and century-wide geospatial datasets available on vector control or “cultural differences”). We explain in detail how our method accomplishes this below (see comment starting on page 5 below and lines 514-622 in Methods), and clarify that we build on a methodologically robust, large, and well-recognized body of prior literature in climate econometrics and epidemiology in constructing our approach (e.g., Childs et al. *PNAS 2025*; Gould et al., *Science Advances 2025;* Rode et al., *Nature 2021)*. Importantly, the regression approach we use robustly identifies the marginal effect of climate variables on transmission, enabling projection of the climate-driven changes in transmission in counterfactual and future scenarios; it does not estimate or project total changes in transmission, which would absolutely require that we incorporate information on non-climate drivers.

Second, the view that temperature does not have a significant effect on mosquito-borne disease dynamics, and only determines the broad geographic area where transmission can take place (i.e., population at risk), is not only incompatible with our findings but also several decades of other work. Here, we examine historical dynamics within a fixed region, and show that temperature and extreme precipitation do indeed have a robust effect on prevalence through time. Although our findings point to some climate-driven redistribution of burden through space, we do not approach these as a change in “population at risk.” This is generally in accordance with other work on this subject; other studies have demonstrated that mechanistic models of $R_0(T)$ predict real-world variation in malaria transmission within endemic areas (e.g., Mordecai et al., *Ecology Letters* 2013; Mordecai *et al. Lancet Planetary Health* 2020), and other attribution studies have now demonstrated that climate change impacts on disease transmission can be separated from socioeconomic drivers using the same econometric approach; notably, one recent study estimates that one in every five cases of dengue fever worldwide is the result of climate change (Childs et al. *PNAS 2025*). In contrast, here we find that climate change has had a much smaller cumulative effect on continent-wide dynamics since 1900 than interventions and other drivers (as the reviewer would expect), but at local short-term scales, the effects can be comparable in magnitude.

While previously published, the Snow et al dataset is inappropriate for a number of reasons. It is profoundly biased in terms of its sampling and incredibly sparse for what is supposed to cover over 100 years. The authors present no new prevalence data, and do not utilise recent incidence data from DHIS2 or other forms of survey data (e.g. on pregnant women).

The Snow dataset is unique in its breadth and depth; we are not aware of any other single, openly-available prevalence dataset for any infectious disease that covers a century and an entire continent, nor one that has anywhere near the sample size of the Snow data. Because we aim to assess continental-scale impacts of climate change on malaria over hundreds of years in the past and into the future, the spatial and temporal coverage of this dataset are critical. We also use the Snow dataset for our study specifically because the accompanying publication in *Nature* concluded that it was unlikely there were significant long-term impacts of climate change detectable in the dataset, making our reanalysis worthwhile – particularly given that our findings

are substantially different from their expectations. (Finally, we note that we are not in a position to collect new data instead; we are not clinicians or field epidemiologists.)

The climate data is monthly, which is what most people in the field have been using for decades, I would have hoped for some more high resolution data for a paper trying to achieve what the authors want. Also the spatial resolution at 50k is incredibly coarse given most prevalence modelling is done at 5km/10km.

We use monthly 0.5 degree resolution weather data to maximize both data quality (given poor coverage of weather stations across Africa, especially in early years) and alignment with our malaria outcome data (which is not available consistently over time at the scale of 5 or 10 km and is only recorded monthly). We have added justification for these decisions to the manuscript, as well as a new sensitivity analysis that demonstrates that spatial aggregation has no effect on recovered effects of climate on malaria in our setting. We detail all changes here.

Regarding data quality, daily weather data are available, and higher temporal and spatial resolution analysis would have been feasible to estimate the impact model, but only for a more recent subset of our full 1901-2014 sample years. Climate data quality across Africa is notably poor, and finely-gridded historical data or reanalysis models would have high potential for error, especially in the first half of the 20th century (Donat et al. *BAMS* 2013). We thus rely on monthly 0.5 degree resolution data from CRU, due to its acceptance as a very widely used and well-documented source of historic data for century-long time series and continental-scale analysis in Africa and for which both monthly temperature and rainfall are provided from the same data development methodologies (Harris et al. *Scientific Data* 2020). In addition, counterfactual and future climate simulations from global climate models have coarse spatial resolution and generating 5km or 10km resolution climate simulation precipitation and temperature using statistical downscaling – running dynamic regional downscaling is beyond the scope of this study – would not alter the coarser resolution signal of climate change from the global climate model.

Second, we have added to the manuscript a new analysis that estimates our statistical model at the grid-cell level, as opposed to aggregating prevalence and climate observations to the administrative level before estimation. We detail this analysis below in response to the reviewer's specific comment regarding spatial aggregation (see pages 19-20 of the response document), but highlight here that we recover nearly identical empirical results with this approach (Figure R1). This suggests that higher resolution climate data – were it available with sufficiently high quality for the long time period we assess – would be unlikely to significantly alter our key findings.

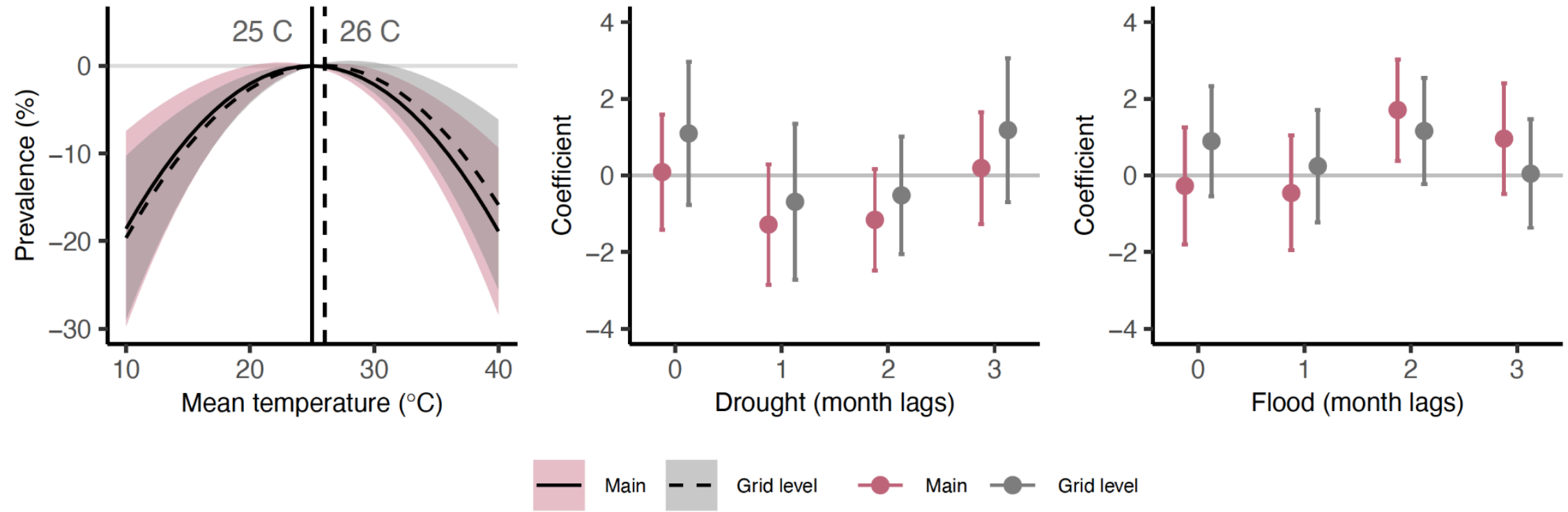

Figure R1 (in text Figure S15): Empirical estimates of climate-prevalence relationships at administrative versus grid cell spatial resolution. Panels show the estimated relationship between malaria prevalence for children aged 2-10 and monthly average temperature (left), drought (middle), and flood (right), estimated separately for our main model specification, aggregated to the first level of administrative division, and for a high-resolution model aggregated to the CRU climate data grid (resolution 0.5°). (Left) The relationship between prevalence and mean monthly temperature for the main ADM1 model (black solid line and pink shading) and high resolution grid-level model (dashed line and gray shading), with shading indicating 95% confidence intervals. (Middle, right) Estimated coefficients and 95% confidence intervals for the effects of drought (middle) and flood (right) events across contemporaneous and lagged months for both model specifications. Positive coefficients indicate higher malaria prevalence associated with extreme climate events. Models are estimated following Equation 4 (main) and Equation 5 (grid level) and all standard errors are clustered at the administrative level (ADM1).

The statistical model is far from the "state-of-the-art' methods that the authors claim. Most geostatistical methods (See Peter Diggles work on this) account for both spatial and temporal residuals (or confounding factors as the authors use) through a Gaussian process, which is a more powerful approach than what the authors use. The "causal interpretation" is not causal, its associative, there is no DAG being estimated, and no estimands being inferred (e.g. ATE via targeted maximum likelihood or double machine learning). This is an associative model and the authors should not claim otherwise. Moreover, the g-computation is very superficial, why have they not created an estimand as is done in econometrics or health (e.g. the work of Hernan).

It appears there has been a substantial misinterpretation of our statistical model. Our methodology derives from a robust, rapidly growing, and increasingly widely adopted literature in climate econometrics (e.g., Auffhammer, *Journal of Economic Perspectives* 2018; Carleton and Hsiang, *Science* 2016; Hsiang, *Annual Review of Resource Economics* 2016) developed explicitly with the goal of enabling a causal interpretation via quasiexperimental methods that flexibly account for spatial and temporal confounding factors and model residuals, while inferring an estimand via ordinary least squares and/or maximum likelihood. To make this clear, in the updated manuscript, we have rewritten the notation of our statistical model and improved the clarity of our language, focusing in particular on communicating the method in a way that is accessible to a broad scientific audience (see updated Statistical Model section on lines 514-622 of the manuscript and pages 7-9 here).

Of course, causal interpretation of any statistical model is always subject to a set of assumptions, which are only partially testable. Throughout the study, we provide numerous robustness and sensitivity analyses to probe the reliability of these assumptions; in the revised manuscript we have added and improved many of these tests (see updated Statistical Model Sensitivity and Robustness section on lines 623-757 of the manuscript).

Here, we: (i) provide intuition for the statistical method we employ; (ii) describe the prior literature leveraging similar methods to recover causal effects of climate change on human health and wellbeing outcomes; and (iii) respond point-by-point to each of the reviewer's specific concerns listed in this comment.

Methodological intuition: The quasiexperimental statistical model we employ – a panel data fixed-effects estimation method – leverages flexible spatial and temporal controls to isolate variation in the weather that is uncorrelated with other socioeconomic or environmental phenomenon. Specifically, the impact of temperatures is *not* being estimated based on a comparison between, for example, the East African highlands and Central African tropics, nor between January and June weather in the same location, comparisons which have many unobservable confounding variables. Instead, the same population is used as both "treatment" and "control" under different temperatures realized in different time periods. For example, a particularly hot July is compared to a cooler July in the same place, *after* controlling for longer-run spatially explicit trends and average seasonal variation. The spatial scope of our analysis provides statistical power to conduct such an estimation by replicating the "experiment" of comparing the "treatment" population versus "control" population many thousands of times.

Prior research leveraging this approach: The panel data fixed-effects estimation method has been leveraged to assess some of the most important problems in climate change research, and these analyses are regularly published in journals with a high standard for robust research methodology. For example, similar econometric approaches have been used to causally link dengue cases (Childs et al., *PNAS 2025*), energy use (Rode et al., *Nature* 2021), aggregate economic growth (Burke et al., *Nature* 2015), all-cause mortality (Gould et al., *Science Advances* 2025; Carleton et al., *Quarterly Journal of Economics* 2022) and human migration (Missirian and Schlenker, *Science* 2017) to the climate, among other outcomes.

Specific methodological concerns: We respond here to each of the reviewer's specific statistical concerns:

Accounting for spatial and temporal confounding factors. Note that spatial and temporal confounding factors (i.e., variables that correlate over space and/or time with both malaria prevalence and climate conditions) differ fundamentally from residuals that are correlated over space and/or time (i.e., correlations in the error term of the regression model). Our statistical method accounts for both, as we explain below. First, for confounding factors, we use "fixed effects" (i.e., a vector of dummy variables that demeans the data by space and/or time) and additional control variables. We use fixed effects at the spatial scale of the ADM1 unit to account for any confounding factors that systematically vary across space but are constant over time,

such as distance from coast or latitude. We also use region-by-month fixed effects to account for any confounding factors that systematically vary across months of the year differentially within each of the four Global Burden of Disease regions, such as seasonality in time spent exposed outside or seasonality in the labor market. We additionally control for country-specific nonlinear time trends – accounting for any confounding factors that are trending over time differentially by country, such as urbanization rates or health services – and for two key malaria intervention periods (1955-1969 and 2000-2015), accounting for the role of two large-scale intervention efforts that dramatically reduced malaria rates across the subcontinent. We show in Figure S7 and Table S2 that alternative fixed effects and controls that address spatiotemporal confounding lead to very similar regression results. The relevant revised text now reads (new text in red):

> **Lines 533-622**
> This approach is designed to approximate controlled experiments by semi-parametrically accounting for unobservable spatial and temporal confounding factors, isolating variation in the climate system that is less likely to be correlated with other socioeconomic factors[99]. This approach is often referred to as "reduced-form", as it allows for a plausibly causal interpretation of recovered relationships between socioeconomic conditions and the climate, but it does not easily enable the researcher to isolate individual *mechanisms* linking a changing climate to shifts in outcomes (e.g., mosquito population dynamics or parasite development rates). However, causal estimates enable counterfactual simulation in which climate is changed and all other factors are held constant; this is the exercise conducted here and in many applications of climate econometric frameworks, including estimating the impacts of climate change on dengue cases[91], international human migration[100], all-cause mortality[90,48], and more. Moreover, these relationships can be used to calibrate more structured transmission models by providing empirical grounding from observational data.
>
> We develop a statistical model using monthly survey-based malaria prevalence data for children aged 2 to 10 (*Pf*PR_{2-10}) covering all of sub-Saharan Africa over 115 years. Our outcome variable is the average prevalence for each first administrative unit *i* (e.g., province or state) in country *c* during month-year *t*, which we denote *Pf*PR_{it}. We estimate prevalence as a flexible function of monthly temperature and precipitation variables as follows:
>
> $$\begin{aligned} PfPR_{it} = &\beta_1 \sum_{g\in i} T_{git}\omega_{gi} + \beta_2 \sum_{g\in i} T^2_{git}\omega_{gi} \\ &+ \sum_{\ell=0}^{L} \rho_\ell \mathbb{1}\{\text{drought}_{i,t-\ell}\} + \sum_{\ell=0}^{L} \psi_\ell \mathbb{1}\{\text{flood}_{i,t-\ell}\} \\ &+ \alpha_i + \gamma_{rm} + \sum_{c\in C} [\phi_{1c}t + \phi_{2c}t^2] \\ &+ \delta_1 \mathbb{1}\{\text{intervention 1}\}_t + \delta_2 \mathbb{1}\{\text{intervention 2}\}_t + \varepsilon_{it}, \end{aligned} \quad (4)$$

where g subscripts denote grid cells, which fall within administrative units i, and ω_{gi} are area weights equal to the share of unit i's area covered by grid cell g, such that $\sum_{g \in i} T_{git} \omega_{gi}$ equals the area-weighted average monthly temperature across all grid cells falling within administrative unit i. Together, parameters β_1 and β_2 recover a quadratic response between prevalence and monthly average temperature. As described above, polynomials are computed before aggregating across grid cells in order to preserve local nonlinearities and avoid aggregation bias. Precipitation extremes are captured by a vector of dummy variables $\mathbb{1}\{drought_{i,t-\ell}\}$ and $\mathbb{1}\{flood_{i,t-\ell}\}$, which indicate whether an administrative unit's monthly rainfall total can be categorized as drought (defined as $\leq$ 10% of the long-run location- and month-specific mean) or flood (defined as $\geq$ 90% of the long-run location- and month-specific mean) during month-year $t - \ell$. We allow for up to three months of lags (i.e., L=3) for these extreme precipitation conditions in our main specification, based on hypotheses from prior literature regarding the timescales of larvae drying and of "flushing"[50,53,101]. A variety of sensitivity analyses detailed below demonstrate that key findings are robust to: including lags for temperature as well as precipitation (Figure S5); the drought and flood cutoffs used for precipitation (Figures S3, S6, and S16); alternative functional forms of temperature (Figure S5); and the estimation of a grid-level regression that does not aggregate prevalence or weather across space (Figure S15).

Equation 4 uses a suite of semi-parametric spatiotemporal controls to isolate variation in climatological conditions that is independent from other disease transmission factors, following standard practices in the climate econometrics literature[45,47]. First, α_i is a vector of indicator variables for each of 853 first administrative units (i.e., "ADM"' units) across our multi-country sample. These spatial "fixed effects" control for all time-invariant characteristics of an administrative unit that may confound the relationship between temperature, rainfall, and prevalence. For example, higher altitude regions may exhibit cooler temperatures, but they also may be composed of lower-income and more geographically isolated communities with limited access to malaria prevention interventions. By controlling for mean conditions in each location, these spatial fixed effects avoid conflating climate conditions with other geographic correlates.

Second, γ_{rm} is a vector of region-by-month-of-year indicator variables, where regions r are defined using the Global Burden of Disease (GBD) regional definitions of western, southern, central, and eastern Africa (see Figure 2 in ref.[102]). Note that the subscript m indicates month of the year (e.g., February), while the month-year index t indicates the month-year time index (e.g., February, 1998). These spatiotemporal fixed effects γ_{rm} account for region-specific

seasonality in prevalence that may spuriously relate to seasonally-varying climatological conditions. We allow these seasonal controls to vary by region because of large differences in climatological seasonality and in malaria cyclicality across sub-Saharan Africa[103], and we show below that our main findings are robust to more stringent seasonality controls defined at the country level (Figure S7). Third, ϕ_{1c} and ϕ_{2c} are coefficients estimating, for each country c in the full set of countries C a nonlinear, country-specific quadratic in the month-year time index, which adjusts the regression for country-specific gradual trends that may confound the malaria-climate relationship, particularly under historical conditions of anthropogenic climate change.

Figure S7 shows that our results are robust to multiple alternative approaches to controlling for long-run trends that may vary across space.

Finally, the indicator variables $\mathbb{1}\{intervention\ 1\}_t$ and $\mathbb{1}\{intervention\ 2\}_t$ are equal to one when an observation falls into the 1955-1969 or 2000-2015 period, respectively. These two periods saw substantial malaria intervention programs across the subcontinent, leading to considerable declines in malaria that were unrelated to changes in the climate[4,104]. These indicator variables control for shocks to prevalence during these two periods, and the coefficients δ_1 and δ_2 allow for differential effectiveness of the two distinct intervention periods. While these variables are highly statistically significant (Table S2), our main findings are robust to their exclusion (Figure S7).

Together, these set of flexible controls imply that the residual variation in temperature and precipitation events used to identify the coefficients β_1, β_2, ρ_ℓ, and ψ_ℓ is month-to-month variation over time within the same location, after controlling for gradual country-specific trends, regional seasonality, and the aggregate effects of two substantial malaria prevention intervention programs.

We estimate Equation 4 using the `lfe` package in `R`. When reporting regression results directly (e.g., in Table S2), we cluster standard errors ε_{imt} at the ADM1 level to account for serial correlation within the same region over time. When computing bootstrap samples (e.g., shown in Figure 2), we repeatedly re-estimate Equation 4 after block-resampling the full dataset using ADM1-level blocks to account for this same serial correlation. In Supplementary Table S5 and Figure S16, we show that model residuals are serially correlated, but exhibit little to no spatial correlation across ADM1 units, justifying this approach to quantifying model uncertainty (see Methods for details). However, we additionally show sensitivity to alternative methods of capturing uncertainty in Figure S17 and Table S6.

Accounting for spatially and temporally correlated residuals. Second, for model residuals, we have added an entirely new section on model uncertainty, which details how we construct estimates of parameter uncertainty via data-driven estimates of spatiotemporal correlations in model residuals. Our approach allows the data to inform the structure of these correlations, versus the Gaussian approach in Peter Diggle's work (e.g., Diggle and Gregori, 2019), which imposes a structural form on such correlations. Our preferred approach accounts for temporal correlation in residuals within an administrative unit over time, but this new section also systematically examines whether additional spatial and/or temporal correlations need to be accounted for. We show that alternative residual correlations are either not present in the data or do not change reported statistical significance substantially. This new section is on lines 720-758, with quantitative assessments of correlation in model residuals found in new Tables S5-6 and Figures S16-17. The revised text now reads (new text in red):

Lines 719-757
Spatiotemporal structure in model residuals

We evaluate the extent to which our model residuals are correlated over space and time using two tests. First, we calculate correlations between residuals across various subsets of our data, following similar tests in ref.[105]. Specifically, we calculate correlations across observations that are: (i) within the same ADM1 unit but from different time periods; and (ii) from different ADM1 units but within the same time period. For temporal correlations, we compute correlations between temporally consecutive observations within up to 3 months of lags, while for spatial correlations we investigate correlations within countries, across countries, within Global Burden of Disease multi-country regions, and based on physical distance (using ADM1 centroids).

Table S5 reports mean correlations, as well as the first and third quartiles of the distribution of correlations across different pairs of units. Rows labeled "temporal" quantify correlations over time while rows labeled "spatial" quantify correlations over space. We note that these tests should be interpreted with care, as the malaria prevalence data are highly unbalanced in space and time, often leaving few observations with which to estimate correlations and/or few regional or temporal pairs over which to summarize a distribution of correlations. To ensure interpretability, we restrict analysis to correlations with at least 10 observations. The last column in Table S5, labeled *N*, indicates the number of pairs for which sufficient data were available to construct correlations for a given grouping. These results reveal moderate serial correlation in residuals, especially for consecutive observations (row 2) (mean ρ=0.40 for consecutive month-years within the same ADM1 unit). In contrast, spatial correlations range from negligible (mean ρ=0.04 across ADM1s from different countries or regions) to modest (mean ρ=0.15 and ρ=0.30 for ADM1s with centroids greater than 500 km and less than 500 km of one another, respectively).

Second, we use a higher-powered test to flexibly investigate spatial correlation by estimating an empirical variogram of our residuals. A variogram estimates the variance of residuals within groups of observations as a function of the distance between them. This is a standard spatial statistics method to systematically assess spatial correlations across a sample[106]. Figure S16 shows the resulting variogram for raw values of the outcome variable (a) and model residuals (b). Each panel shows semi-variances on the *y*-axis and distance between centroids of ADM1 units on the *x*-axis. When spatial correlation is present, semi-variances rise with distance, as in panel (a). In contrast, panel (b) shows essentially a flat line, indicating little to no spatial correlation in model residuals.

Given these results, we cluster standard errors at the ADM1 level, accounting for correlation in model residuals across all month-years for a given administrative unit. However, in Figure S17 and Table S6, we show sensitivity of our recovered confidence intervals to alternative approaches to standard error estimation, including those that account for spatial correlation (e.g., Conley standard errors).

Kind	Group	$\bar{\rho}$	Q25	Q75	N
temporal	all	0.18	-0.09	0.48	511
temporal	1 month lag	0.40	0.20	0.65	60
temporal	2 month lag	0.17	-0.06	0.43	33
temporal	3 month lag	0.22	-0.05	0.43	16
temporal	same month, different year	0.22	-0.04	0.53	41
spatial	all	0.24	-0.01	0.54	635
spatial	same country	0.28	0.04	0.56	537
spatial	different country	0.04	-0.18	0.28	98
spatial	same region	0.04	-0.17	0.19	51
spatial	different region	0.04	-0.19	0.30	47
spatial	$<$ 500km	0.30	0.04	0.60	395
spatial	$>$ 500km	0.15	-0.10	0.39	240
spatial	$>$ 1000km	0.07	-0.17	0.33	103

Table R1 (in text Table S5): Summary statistics for temporal and spatial correlations of model residuals. Values show mean correlation ($\bar{\rho}$), interquartile range (Q25, Q75), and sample size (*N*) for different groupings temporally (Kind = “temporal”) or spatially (Kind = “spatial”), following ref.[105]. For temporal groupings, correlations between month-year observations within the same ADM1 unit are estimated. For spatial groupings, correlations between month-year sequences of residuals from different ADM1 units are estimated. Here, “region” indicates one of four Global Burden of Disease regions. Means, first quartiles, and third quartiles of the distribution of correlations from each kind of grouping are shown. *N* indicates the number of pairs for which correlations could be estimated; pairs with less than 10 matching observations were dropped.

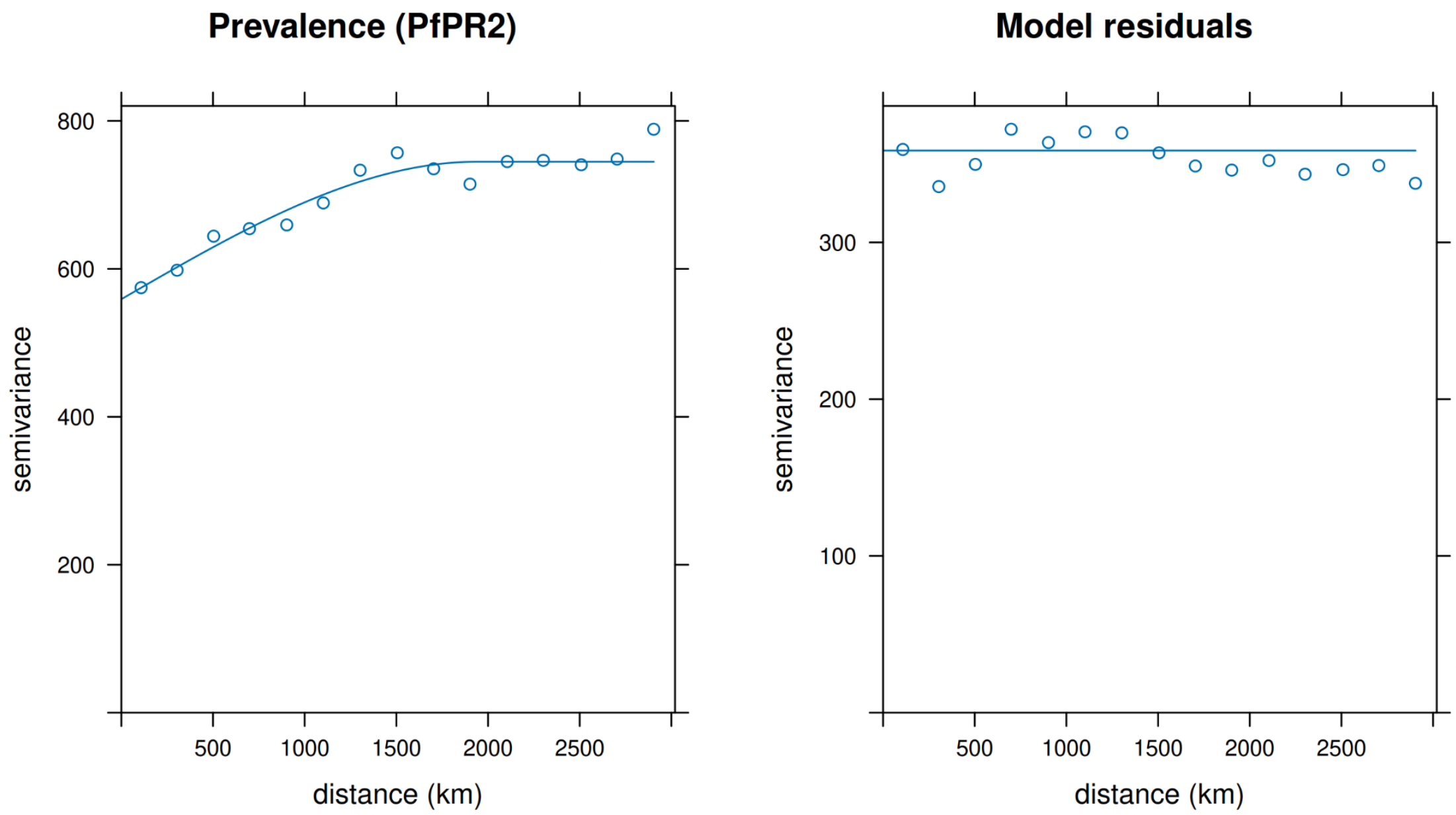

Figure R2 (in text Figure S16): Empirical variogram of malaria prevalence and regression model residuals. Each panel shows estimated semi-variances (*y*-axis) against distance between spatial units (*x*-axis). Semi-variances are defined for each distance *h* as half the variance of the difference between values located within *h* units of each other[106]. When spatial correlation is present, semi-variances rise with distance, as variance grows with the distance between observations. (Left) The variogram for malaria prevalence *Pf*$PR_{2\text{-}10}$. (Right) The variogram for regression model residuals, as estimated following Equation (4). Hollow circles indicate binned scatters while solid lines indicate a spherical variogram model fit. All estimates derived using the `gstat` package in `R`.

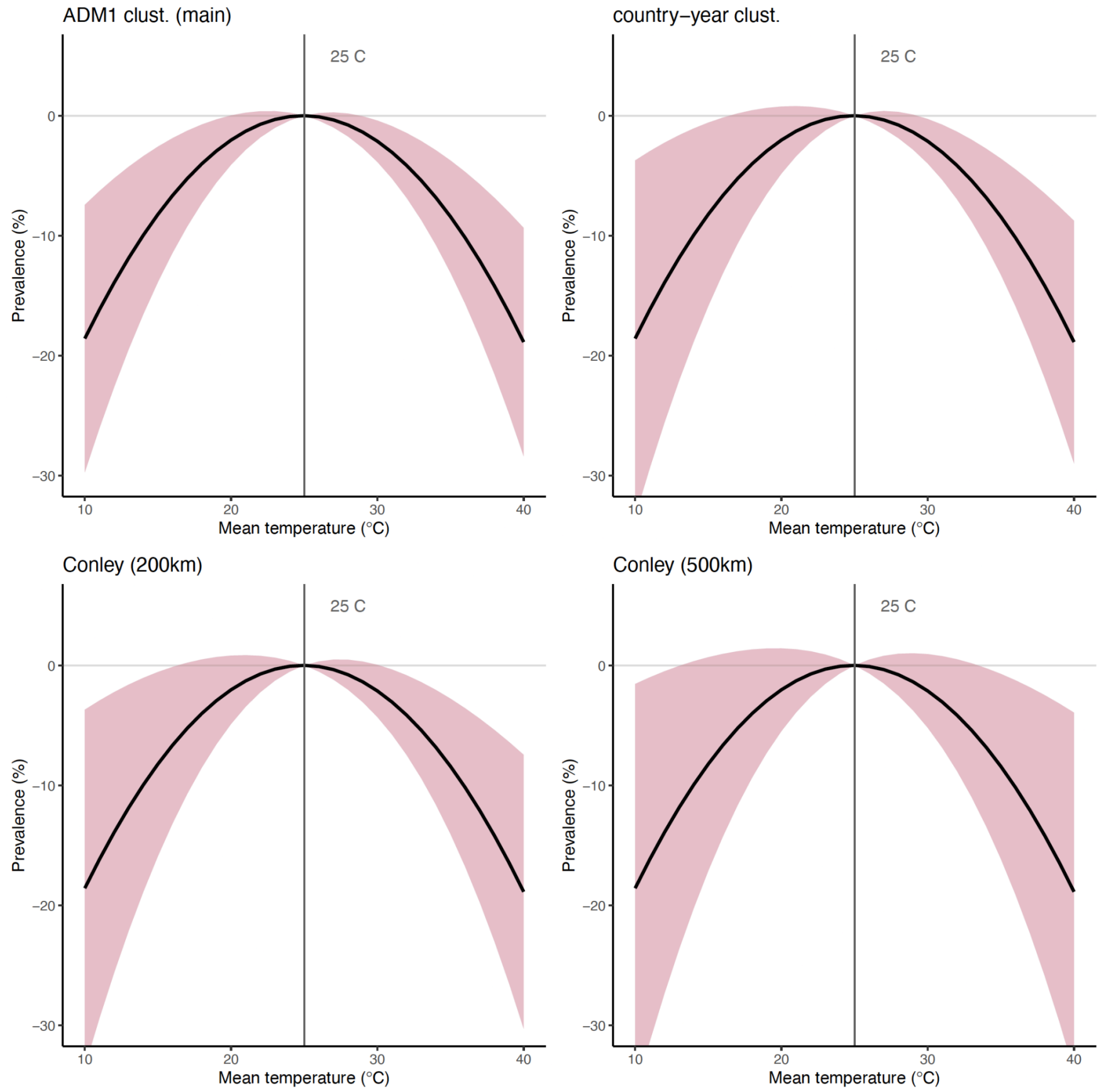

Figure R3 (in text Figure S17): Alternative estimates of model uncertainty in the *Pf*PR$_{2\text{-}10}$-temperature relationship. All panels show the estimated relationship between malaria prevalence for children aged 2-10 and monthly average temperature as estimated following Equation (4). All temperature responses are plotted relative to 25ºC, the temperature at which prevalence is maximized. From top-left to bottom-right, standard errors are estimated using: ADM1-level clustering (main specification), country-by-year level clustering, Conley standard errors with a cutoff of 200 km, and Conley standard errors with a cutoff of 500 km. Tabular results, including associated estimates of precipitation extremes, are shown in Table S6.

Claims of causality. Of course, claims of causality require assumptions. We have updated the manuscript to make this clear, only using the word “causal” minimally, and with qualification. We now use it only once to describe our specific method, stating:

> **Lines 536-540**
> This approach is often referred to as ‘reduced-form’, as it allows for a plausibly causal interpretation of recovered relationships between socioeconomic conditions and the climate, but it does not easily enable the researcher to isolate individual *mechanisms* linking a changing climate to shifts in outcomes (e.g., mosquito population dynamics or parasite development rates).

In our econometric model, a causal interpretation is merited when a very explicit set of assumptions – the Gauss-Markov assumptions (Wooldridge, *MIT Press* 2002) – hold. Importantly, these assumptions include that no confounding factor omitted from the regression correlates with both climate variables and with prevalence. The fixed effects and spatiotemporal controls described above are included in order to render precisely the Gauss-Markov assumptions plausible, and we rigorously evaluate them, as discussed in the point above (see Methods section Statistical model sensitivity and robustness on lines 623-757).

Inferring an estimand via maximum likelihood. The model *is* inferring an estimand – specifically, we minimize the sum of squared residuals to recover an average treatment effect (ATE), which is equivalent to maximizing the following likelihood function under the Gauss-Markov Assumptions (Wooldridge, 2002): $L(\sigma, \beta) = \prod_{i=1}^{n} \left(\frac{1}{\sqrt{2\pi\sigma^2}} \right) exp \left(- \frac{(y_i - \hat{y}_i)^2}{2\sigma^2} \right)$, where *i* denotes observations in the sample. We note that the targeted MLE and double machine learning methods suggested by the reviewer are not appropriate in this setting, as we use a quasiexperimental design to generate random variation in our treatment of interest. That is, the climate system has inherent randomness that leads to unanticipated variability in the specific weather realized in a specific month in a specific place. Our spatiotemporal controls are designed to isolate precisely this variation. In contrast, targeted MLE and double ML are relevant when the statistician has no quasiexperimental design to isolate random variation, but instead uses a large vector of controls and flexible machine learning methods to *learn* the confounding relationships and adjust for them through orthogonalization.

Relationship to econometrics. This panel data fixed-effects estimation strategy is precisely the type of model estimated in econometrics and in particular in econometric analyses of health outcomes (e.g., Childs et al., *PNAS* 2025; Young and Hsiang, *Nature*, 2024; Deschenes and Greenstone, *American Economic Journal: Applied Economics* 2011; Heutel et al., *Review of Economics and Statistics* 2021).

Directed Acyclical Graphs (DAGs). We choose not to show a full (Figure R4) or collapsed (Figure R5) DAG for the conceptual model we are testing. This is because (i) we are not comparing alternate hypotheses about causal networks (i.e., competing DAGs), and (ii) as with many causal problems in climate epidemiology, a full DAG for the system would be overwhelmingly complicated (Figure R4), while a DAG that represents the causal question we are asking here would be so simple as to provide minimal additional value (Figure R5). To illustrate this point, here are two examples from another ongoing project (courtesy of Cole Brookson) showing a full “system map” for malaria and climate change, versus a representation of the causal questions answered in a similar study design. (Here, we are asking similar questions but using data on prevalence, not incidence, and using a regression approach rather than a compartmental [SIR] model.) We could include a version of Figure R5 if the reviewer or editor feels this would be helpful!

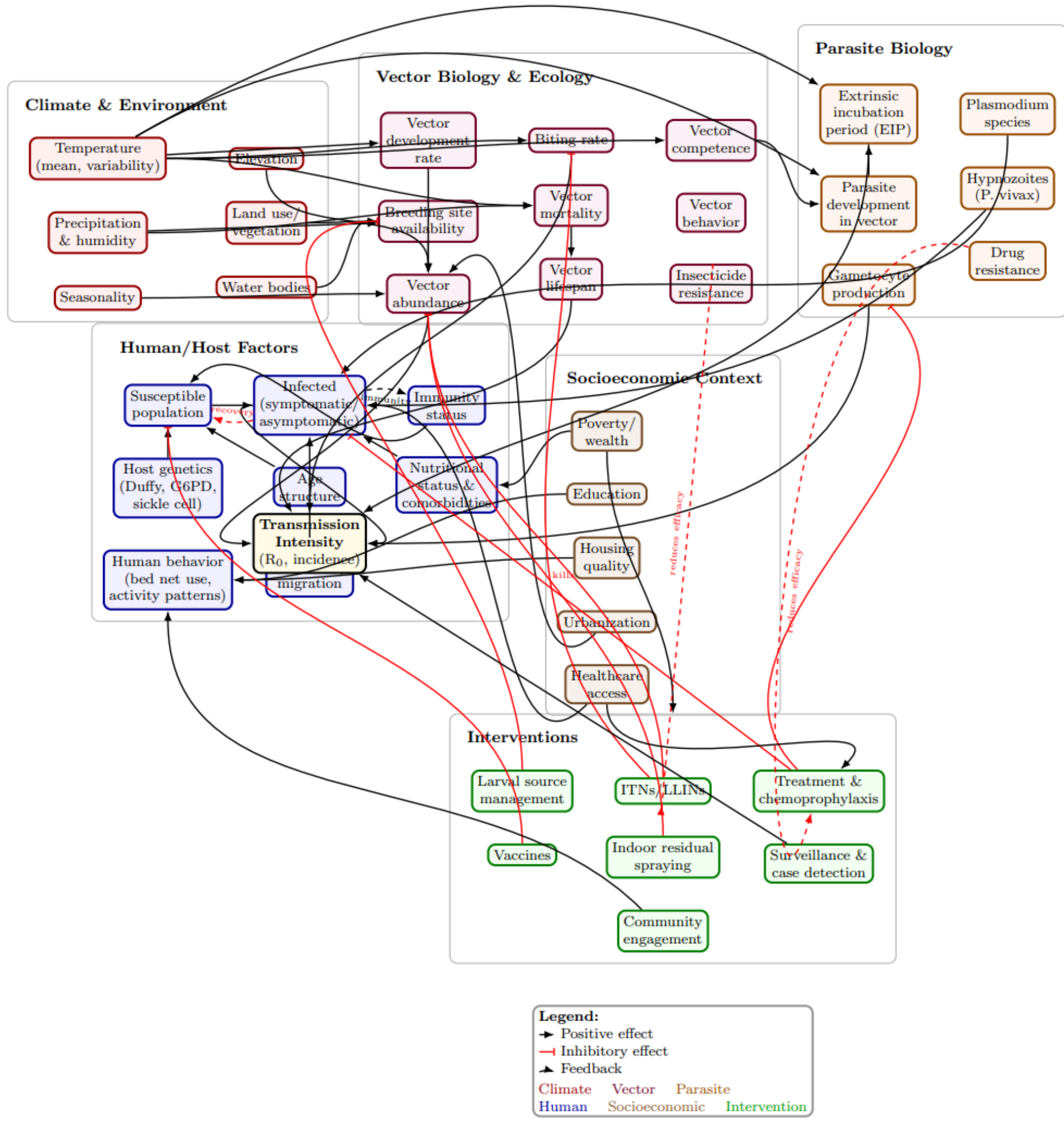

Figure R4. Full Directed Acyclical Graph. This example figure (taken from another project on malaria in the lead author's lab) shows the tremendously complex relationships between the biological and social drivers of malaria transmission, which cannot be accounted for completely in any statistical model.

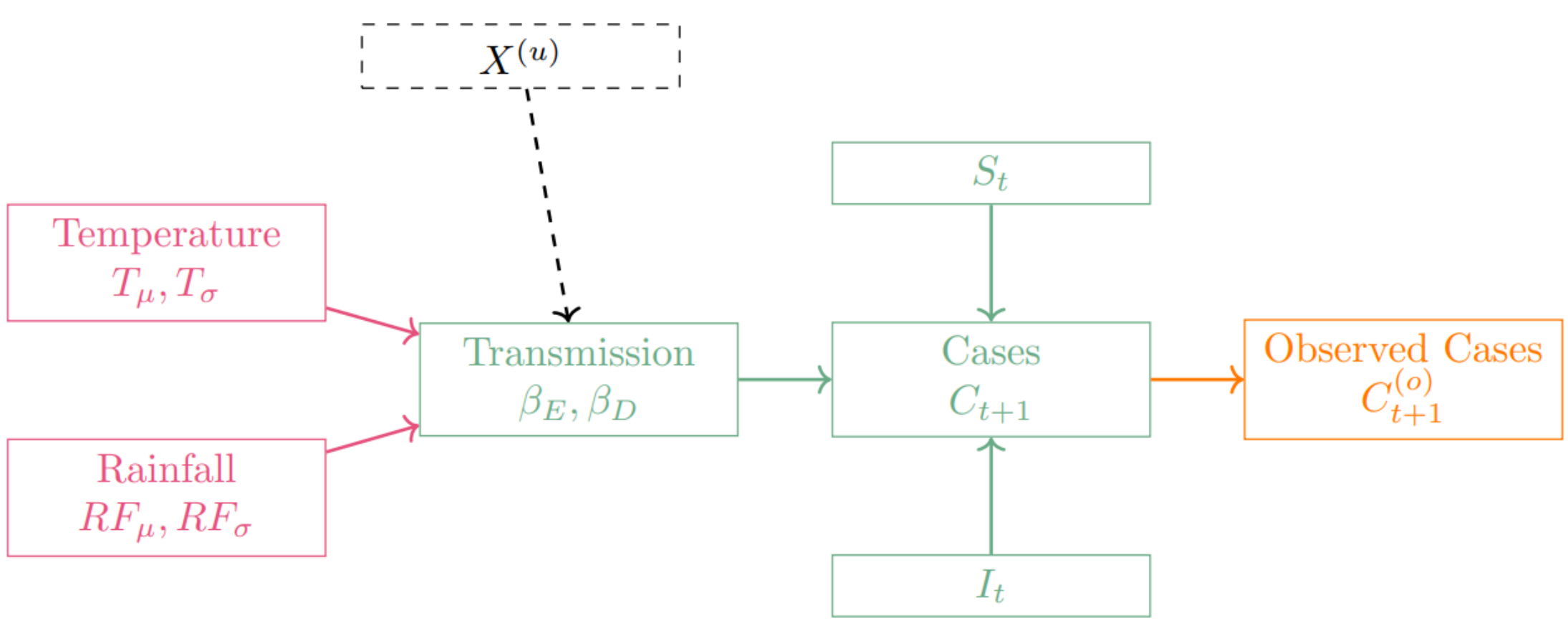

Figure R5. Simplified Directed Acyclical Graph. The reduced form model used by Brookson *et al.*, *in prep*., which suffices for inference of climate effects so long as there are controls for any possible confounders.

Given point spatial data, why are the authors using admin1? How are they aggregating to get the admin1 estimates, what weighting are they using (see https://www.ncbi.nlm.nih.gov/pmc/articles/PMC8055469/ for example).

We thank the reviewer for this question – it is clear from this review and others that the original manuscript was insufficiently clear regarding how and why we spatially aggregate climate and prevalence data. In response, we have made two substantial changes. First, we added a section that depicts exactly how we conduct spatial aggregation using a method explicitly designed to mitigate any spatial aggregation bias (lines 417-513, shown below). Second, we conduct a fully new analysis involving no spatial aggregation above the resolution of the climate data (Figure S15). Before describing these changes, we clarify for the reviewer *why* aggregation was necessary.

Our statistical model relies on comparisons of weather realizations over time for the same spatial unit, in order to isolate variation in weather realizations that are independent from other drivers of malaria prevalence. The point data available from Snow et al. (*Nature*, 2017) do not consistently capture repeated measurements at the same geolocation, making such a model infeasible with point-level observations. Thus, we follow the source of the data (Snow et al., *Nature* 2017) in averaging points across each ADM1 unit for each month, which allows us to construct an estimate of ADM1-level prevalence for multiple months over multiple years, making our statistical approach feasible and avoiding classic challenges of confounding variables when comparisons are made across space in estimation (Auffhammer, *Journal of Economic Perspectives* 2018).

We conduct this averaging using an unweighted arithmetic mean over all observations observed in one ADM1 during the same month. This choice was made because it imposes minimal assumptions on spatiotemporal processes and requires no additional high-resolution data on variables that we would use as weights (e.g., population); these data are impossible to obtain for sub-Saharan Africa for years as early as 1901. Moreover, employing the spatiotemporal smoothing approaches in the work cited by the reviewer would artificially introduce spatial and temporal correlations that could bias recovered coefficients and threaten inference (Muller, 2022). We note that the work cited by the reviewer aims to construct ADM2 level mortality rate time series, which is a very different goal than uncovering unbiased estimates of the *effects* of local temperature and precipitation on malaria prevalence.

To ensure our motivations and implementation for spatial aggregation are clear, we have added a new Methods section titled "Spatial data aggregation" that is dedicated to this component of our analysis. The added text now reads (new text in red):

> **Lines 417-513**
> **Spatial data aggregation**

Our statistical analysis is designed to isolate variation in the weather that is uncorrelated with other socioeconomic and/or environmental factors that influence malaria prevalence. As detailed in the next section, we build on a large body of climate econometrics research[45,47,85] to do so, estimating a model that leverages variation over time in weather conditions within the same location. To estimate such a model, we require observations of malaria prevalence covering the same region in multiple time periods. In contrast, the raw prevalence data we obtain from ref.[4] are point data observations from individual surveys conducted at different times, such that single geolocations are not observed repeatedly over time. Therefore, we aggregate the point-level data from ref.[4] by averaging *Pf*$PR_{2\text{-}10}$ observations to the first administrative level within each country (i.e., state or province level, or as shorthand, ADM1), using shapefiles provided by the Database of Global Administrative Areas dataset version 3.6 (www.gadm.org). This level of aggregation provides sufficient granularity to capture differences in climate impacts within countries and to control for local heterogeneity in confounders, while ensuring sufficient data coverage within these units. This aggregation scale is also supported by previous work that models this dataset at the same spatial resolution[4]. For robustness, we also show results from a statistical model that does *not* aggregate data, and instead uses the prevalence data at its native resolution (see below for details).

To compute average prevalence values at the scale of the first administrative unit (i.e., ADM1), we use an unweighted arithmetic mean over all prevalence surveys observed in the corresponding ADM1-month. This approach imposes minimal assumptions on the spatiotemporal process of malaria transmission and requires no additional high-resolution data (e.g., population) for use as weights, which are unavailable for sub-Saharan Africa for years as early as 1901. Although prior work aiming to construct comprehensive high-resolution estimates of health outcomes using point data often employs spatio-temporal smoothing methods (e.g., ref.[86]), doing so here would artificially introduce spatial and temporal correlations that could bias recovered regression coefficients and threaten inference[87].

We similarly aggregate monthly 0.5° grid-level weather data to the ADM1-month level. To do so without introducing aggregation biases, we apply methods from previous research demonstrating that it is possible to statistically recover nonlinear relationships that take place at high spatial and temporal resolution, even when the resolution of available outcome data is relatively coarse (i.e., ADM1-month level average malaria prevalence)[47,88,89,90,91]. In our setting, this is achieved by computing nonlinear polynomial transformations of temperature at the grid-cell-by-month level *before* aggregating these values across administrative units. Such an approach ensures the temperature variables used for estimation reflect the full distribution of temperatures experienced across

administrative regions of varying sizes and terrains. For example, many of Ethiopia's 12 ADM1 regions include both hot low-elevation zones and cold highlands, such that temperatures can vary substantially within an ADM1 during the same month. We compute second-order polynomials at each grid cell before aggregating across such diverse landscapes to ensure the regressor variables capture both extreme cold and extreme heat, even when they occur simultaneously within the ADM1's boundaries.

To see this method in practice, let $Pf\mathrm{PR}_{git}$ denote average malaria prevalence in children aged 2-10 in grid cell *g* located within administrative unit *i* during month *t* and let T_{git} indicate temperature observed at the same spatiotemporal scale. Following prior studies recovering local-level quadratic responses between malaria prevalence and temperature[92,93], we assume that prevalence in grid cell *g* in month *t* is a quadratic function of the temperature experienced in that same grid cell and month (noting that we show results relaxing this assumption, such as other nonlinear functional forms and the possibility of temporal lags):

$$Pf\mathrm{PR}_{git} = \beta_1 T_{git} + \beta_2 T_{git}, \tag{1}$$

where β_1 and β_2 are constant average coefficients. As discussed above, we cannot empirically estimate a model like Equation 1 reliably because we do not observe prevalence over multiple months *t* for the same grid cell *g*. Instead, our empirical specification relies on average ADM1-month level prevalence variables $Pf\mathrm{PR}_{it}$. Thus, we must aggregate Equation 1 in a manner that allows us to recover the same β_1 and β_2 coefficients that describe the local-level temperature response, and which we would have recovered had we been able to estimate Equation 1 directly. Specifically, average ADM1-month prevalence can be written as:

$$\begin{aligned} Pf\mathrm{PR}_{it} &= \sum_{g \in i} Pf\mathrm{PR}_{git}\omega_{gi} = \sum_{g \in i}(\beta_1 T_{git} + \beta_2 T_{git}^2)\omega_{gi} \\ &= \beta_1 \sum_{g \in i} T_{git} w_{gi} + \beta_2 \sum_{g \in i} T_{git}^2 w_{gi}, \end{aligned} \tag{2}$$

where ω_{gi} denotes a grid-by-ADM1 weight. In our setting, we estimate an area-weighted average prevalence value by setting ω_{gi} equal to the share of administrative unit *i*'s area that falls into grid cell *g*, as the lack of high-resolution population data make population weighting infeasible.

Equation 2 shows that a regression of average prevalence in ADM1 unit *i* and month *t* on variables that are ADM1-month weighted aggregates of the nonlinear temperature terms T_{git} and T_{git}^2 will, in expectation, recover the same coefficients β_1 and β_2 that describe the fundamental grid-level relationship described by Equation 1. This same procedure has been used to estimate the relationship

between: monthly administrative-level dengue incidence and daily grid-level temperature[91]; annual all-cause mortality and daily grid-level temperature[48,90]; annual country-level crop yields and daily grid-level soil moisture[94]; among many other examples. As in these other cases, our approach mitigates aggregation bias up to the level of the grid resolution of the climate data. While climate and prevalence likely vary across space within each grid cell, we cannot resolve such dynamics here, given the lack of reliable higher-resolution weather data in Africa over the extended time frame of our analysis[77].

We note that one could, alternatively, construct nonlinear weather variables *after* aggregating to the administrative unit, thus estimating:

$$PfPR_{it} = \tilde{\beta}_1 \sum_{g \in i} T_{git}\omega_{gi} + \tilde{\beta}_2 (\sum_{g \in i} T_{git}\omega_{gi})^2, \quad (3)$$

where recovered parameters $\tilde{\beta}_1$ and $\tilde{\beta}_2$ are biased relative to the fundamental relationship in Equation 1 because $(\sum_{g \in i} T_{git}\omega_{gi})^2 < \sum_{g \in i} T^2_{git}\omega_{gi}$, due to Jensen's Inequality.

Thus, we construct a vector of ADM1-month temperature variables by computing nonlinear transformations at the grid level before aggregating across space, following Equation 2. While we could, in principle, follow the same procedure for precipitation, we instead compute drought and flood variables at the ADM1-month level, due to high rates of mismeasurement in grid-level rainfall estimates[77] and due to the likelihood that prevalence-precipitation relationships occur over larger spatial scales than a single grid cell (i.e., water flows through hydrological systems linking precipitation in one location to water availability and prevalence downstream). We show in a robustness exercise detailed below that using grid-level precipitation data generates similar results as our aggregated model, but increases uncertainty, consistent with evidence on rainfall mismeasurement in Africa.

All main results rely on the spatial aggregation procedure described above. However, we additionally estimate an alternative model that leverages the point-level prevalence data directly, introducing no spatial aggregation beyond the resolution of the weather data (0.5°). As we detail below and show in Supplementary Figure S15, the recovered prevalence-temperature results are very similar using these two distinct methods.

Second, as mentioned above (see comment on page 4) we conduct an entirely new analysis in which we estimate the regression model at the native grid level of the climate dataset, imposing very minimal spatial aggregation of malaria data (while in theory more than one survey may be present in a given climate grid cell, in practice this is very rare, leading to minimal aggregation

across surveys). While this model is not our preferred specification due to its statistical properties (discussed directly below and in Methods section titled “Estimating a grid-level regression” on lines 685-718), we show that the recovered prevalence-temperature relationship is very similar to that recovered in our main specification, demonstrating that aggregation biases are unlikely to be driving our results. Grid level results are shown in Figure S15. The added text now reads (new text in red):

Lines 685-718
Estimating a grid-level regression

As described above, our main analysis relies on an ADM1-level regression in which malaria prevalence and weather data are aggregated from higher spatial resolutions to the ADM1 scale. Here, we show the results from an alternative approach, in which point-level malaria prevalence survey observations are matched to corresponding 0.5º resolution CRU climate data grid cells and the regression is estimated at this grid level.

Using these disaggregated data, we estimate a regression model analogous to Equation 4, but modified to fit the spatial scale of the data. Specifically, we estimate:

$$\begin{aligned} Pf\text{PR}_{git} &= \eta_1 T_{git} + \eta_2 T_{git}^2 \\ &+ \sum_{\ell=0}^{L} \lambda_\ell \mathbb{1}\{\text{drought}_{gi,t-\ell}\} + \sum_{\ell=0}^{L} \xi_\ell \mathbb{1}\{\text{flood}_{gi,t-\ell}\} \\ &+ \alpha_i + \gamma_{rm} + \sum_{c\in C} \left[\phi_{1c} t + \phi_{2c} t^2\right] \\ &+ \delta_1 \mathbb{1}\{\text{intervention 1}\}_t + \delta_2 \mathbb{1}\{\text{intervention 2}\}_t + \varepsilon_{git}, \end{aligned} \quad (5)$$

where all variables are defined as above for Equation 4. In particular, $Pf\text{PR}_{git}$ represents prevalence reported for a survey located in grid *g* falling within ADM1 unit *i* during month *t*, T_{git} denotes temperature in the same grid and month, and precipitation extremes are captured via dummy variables $\mathbb{1}\{drought_{i,t-\ell}\}$ and $\mathbb{1}\{flood_{i,t-\ell}\}$ that indicate when each grid cell's monthly rainfall total is less than 10% of its long-run month-specific mean (drought) or more than 90% (flood).

Two features render this estimating equation distinct from the main analysis. First, temperature and precipitation variables are matched exactly to the grid cell within which the malaria survey was conducted, such that no aggregation is necessary. This increases the precision of the match between weather and outcome variables. Second, the spatial “fixed effects” denoted by α_i – that is, indicator variables for each of the ADM1 units in our sample – are estimated at a lower spatial resolution (ADM1) than the data itself (grid). This implies that the

weather variation used to identify coefficient vectors $\boldsymbol{\eta}$, $\boldsymbol{\lambda}$ and $\boldsymbol{\xi}$ includes both variation over time within an ADM1, but also across survey locations located within the same ADM1. Thus, while this model has the benefit of leveraging high spatial resolution in weather, it potentially suffers from omitted variables bias, as weather conditions in different survey locations may be correlated with other unobservable determinants of malaria prevalence (e.g., access to health care, rates of poverty, proximity to water bodies).

We show in Figure S15 that the malaria prevalence-temperature relationship recovered from estimation of Equation 5 is incredibly similar to that estimated from our main regression model in Equation 4, suggesting that aggregation of the underlying survey data does not influence the key results of the paper. In contrast, the estimated drought and flood coefficients recovered from the grid-level regression are highly imprecise, consistent with substantial measurement error in local-level precipitation datasets in Africa for much of the 20th century[77].

I am unclear what the likelihood function is and how equation 1 is estimated. Are they accounting for over-dispersion, why are they bootstrapping as opposed to using Bayesian models that are far more common in this area (again see Model Based Geostatistics by Diggle).

We estimate Equation 1 (now Equation 4) using ordinary least squares (OLS), minimizing the sum of squared residuals using the `lfe` package in `R`. We now note this on line 614 in the Methods section (see pages 7-9 of this reviewer document for full changes). OLS is equivalent to maximum likelihood estimation under the assumption of normally distributed errors, given that our model is linear in the coefficients, with the following likelihood function (Wooldridge, *MIT Press* 2010):

$$L(\sigma, \beta) = \prod_{i=1}^{n} \left(\frac{1}{\sqrt{2\pi\sigma^2}} \right) exp\left(- \frac{(y_i - \hat{y}_i)^2}{2\sigma^2} \right)$$

Regarding overdispersion, Figure R6 shows that the regression model residuals are distributed normally. Unbiasedness of coefficients and efficiency of standard errors in our OLS model are therefore not threatened by overdispersion (Wooldridge, *MIT Press* 2010). As mentioned above, an entire section of the text is now dedicated to understanding and estimating model uncertainty (lines 719-757, see response pages 10-11).

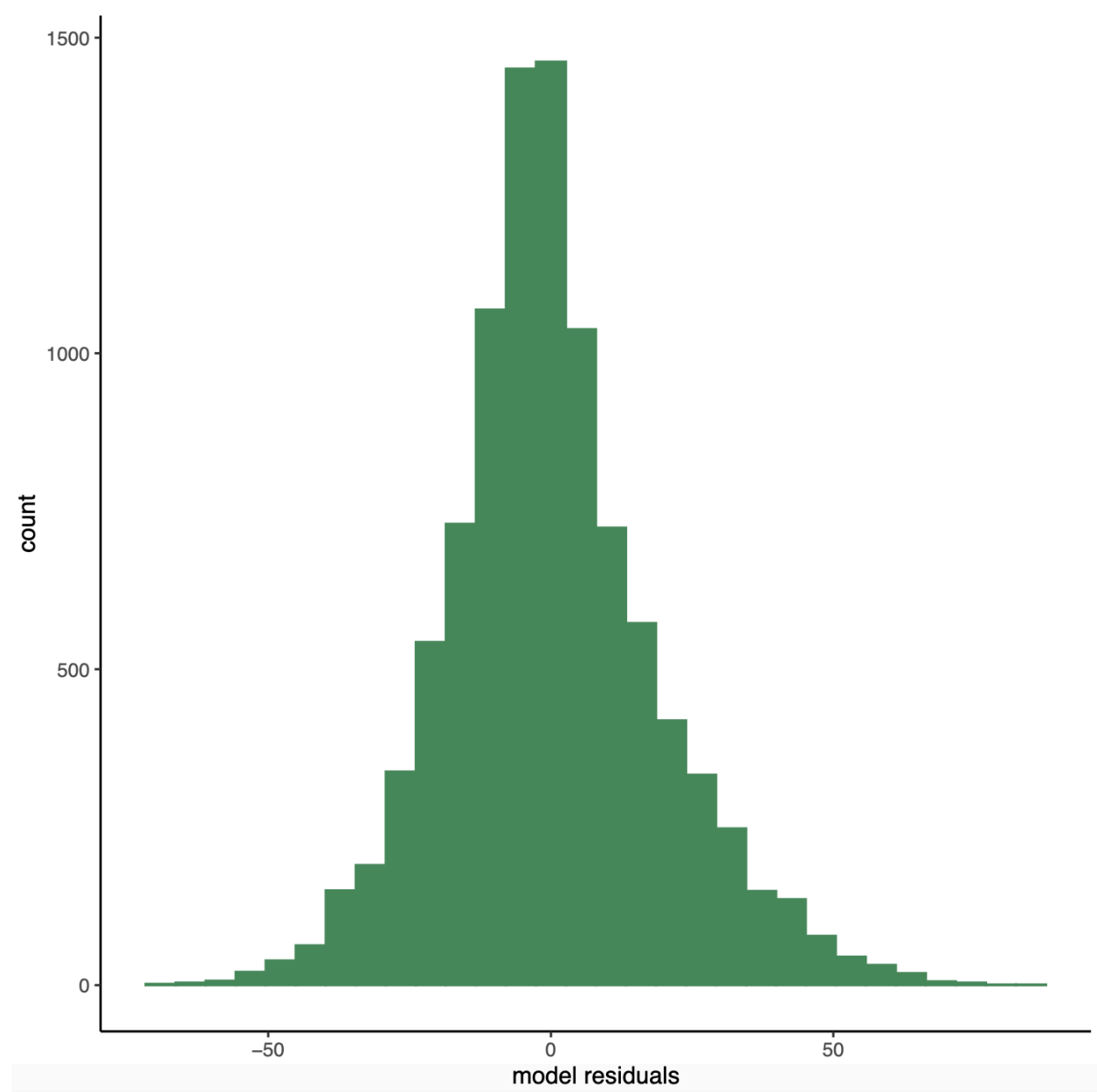

Figure R6. Distribution of residuals from the main regression model (Equation 4, lines 514-622).

Finally, bootstrapping is an exceptionally common and theoretically grounded computational method for representing statistical uncertainty. The seminal text on the estimation procedure – Efron and Tibshirani (*Chapman and Hall*, 1994) – has over 54,000 citations. Moreover, this method is very commonly applied in climate impacts and attribution analysis, including, for example: Burke et al. (*Nature*, 2015); Diffenbaugh and Burke (*PNAS* 2019); Heft-Neal et al. (*Nature Sustainability* 2020); Ortiz-Bobea et al. (*Nature Climate Change*, 2021); and many others.

My biggest issue again is what is missing from equation 1, all the factors i described above. The malaria data and methods have existed for a long time, the reason others have not adopted simplistic approaches such as those in this paper is that they are not suitable for the question they are looking to answer.

As articulated above, our statistical method *does* account for the factors mentioned by the reviewer – e.g., socioeconomic status, infrastructure, vector control, cultural differences – through a set of flexible nonparametric controls called "fixed effects" in an econometric model that has been widely used to quantify the effects of climate change on many social and economic outcomes. This allows us to conduct a robust statistical analysis that has, indeed, been impossible for other researchers using other methods that would require explicit data on these covariates.

i am unsure from the paper what are g() and h(), how are the authors accounting for known dynamics of rainfall? for example, rainfall is heavy tailed (https://journals.aps.org/pre/abstract/10.1103/PhysRevE.66.036120) does their monthly mean

even make sense in this case? how are these extreme events accounted for in their simple regression model.

We apologize that the functions g() and h() were not transparently communicated. In response to this and another reviewer's comments, we now write out Equation 1 (now Equation 4 due to other changes in the text) fully, indicating all components of the previous functions g() and h(). Importantly, this new Equation 4 makes clear that g() is a set of indicator variables that capture the heavy tails of the rainfall distribution, just as the reviewer suggests may be important for malaria prevalence. As we show above (see page 7), Equation 4 now includes *drought* and *flood* which are defined as indicator variables equal to 1 when an ADM1-month experiences total rainfall that is less than the 10th percentile of the location-specific long-run monthly rainfall distribution or greater than the 90th percentile of this distribution, respectively.

Minor comments
line 60 - Gething et al Nature also did this analysis and found the same conclusion as Snow, and therefore the authors attempt to dismiss Snow et als conclusion is not warranted.

A careful comparison of our study with these two previous studies reveals a substantial difference in methodology – one that motivated our study in the first place.

Snow *et al.* did not perform a statistical analysis of the impacts of climate change on malaria transmission before concluding the former could not explain the latter: the two are merely plotted on the same graph. We note this in our Introduction:

> **Lines 60-64**
> More recently, a study by Snow *et al.*[4] examined the last century of continent-wide changes in malaria prevalence, and concluded that observed trends could not be neatly explained by climate change, but did so based only on visual correspondence between moving averages of rainfall, minimum temperature, and modeled malaria prevalence over the entire continent.

We believe the study the reviewer is referring to is Gething et al. (2010) "Climate change and the global malaria recession" – which is unfortunately *another* study that concludes climate change has not influenced malaria prevalence without developing an observational statistical analysis. What Gething and colleagues do in that study is map global reductions in malaria transmission, compare them to projections from the literature that climate change would increase R_0 for malaria, and conclude the two are incompatible. This study, which predates the Snow study by seven years, suffers from the same core limitation.

Unlike these studies, we address the same topic with a rigorous statistical analysis, demonstrating that climate change has unequivocally increased malaria prevalence in many areas. However, we also show that this small increase is compatible with much more substantial reductions in malaria transmission – essentially confirming the hypothesis of both Gething and Snow that if climate change is having an effect, it must be substantially smaller than other

factors. We therefore see our study as a significant new contribution and important follow-up to previous well-cited work.

line 320 - if using the snow data, it is not clear if the authors have also adjusted for diagnostic type (PCR/RDT) or differences in testing such as the infamous DRC 2006 PCR survey.

We thank the reviewer for this suggestion. While we did not originally account for differences in testing type, we have now added a new analysis in which we control for the diagnostic type of the test. Results are shown in Table R2 below and are in the manuscript as Supplementary Table S1. We find that adding these controls has no effect on our estimates of temperature or precipitation effects on malaria prevalence. Specifically, column (1) shows our main specification, while columns (2) and (3) add controls for the dominant diagnostic method used in each ADM1-month (note that it is rare for more than one method to be used in one ADM1-month). Column (2) uses all diagnostic method categories provided in the Snow et al. (*Nature* 2017) data, while Column (3) simplifies these categories into larger groupings. Coefficients on temperature, flood, and drought are very similar in magnitude and statistical significance across all three columns.

We mention this result in the main text (shown below) and include the results in Supplementary Table S1. The updated text now reads (new text in red):

> **Lines 144-152**
> Additional sensitivity analyses reinforce that these prevalence-climate relationships are both statistically robust and biologically consistent. Key findings are stable through time (Figure S8), and are generally insensitive to alternative model specifications, such as: the inclusion of lagged effects of temperature (Figure S4); higher-order polynomial effects of temperature (Figure S5); alternative definitions of drought and flood shocks (Figures S3, S6, and S18); controlling for the diagnostic test type (Table S1); and alternative spatiotemporal controls, which account differently for variation over space (at region, country, and state levels), time (including yearly and monthly variation), and interactions among space and time (Figure S7 and Table S2).

	(1)	(2)	(3)
Avg temp. (°C)	4.15***	3.97***	4.08***
	(1.08)	(1.09)	(1.09)
Avg temp.^{2} (°C)	−0.08***	−0.08***	−0.08***
	(0.02)	(0.02)	(0.02)
Flood	−0.28	−0.06	−0.22
	(0.78)	(0.78)	(0.78)
Flood (lag 1)	−0.45	−0.45	−0.47
	(0.76)	(0.77)	(0.76)
Flood (lag 2)	1.70**	1.81***	1.83***
	(0.68)	(0.67)	(0.67)
Flood (lag 3)	0.96	1.05	0.97
	(0.74)	(0.74)	(0.74)
Drought	0.09	0.18	0.23
	(0.77)	(0.76)	(0.77)
Drought (lag 1)	−1.28	−1.38*	−1.28
	(0.80)	(0.81)	(0.80)
Drought (lag 2)	−1.14*	−1.18*	−1.15*
	(0.68)	(0.68)	(0.68)
Drought (lag 3)	0.19	0.09	0.15
	(0.74)	(0.75)	(0.75)
Intvn. 1955-69	−4.82***	−4.98***	−4.76***
	(1.27)	(1.28)	(1.27)
Intvn. 2000-15	−3.37**	−3.19**	−3.60**
	(1.61)	(1.60)	(1.60)
Diag. Method Microscopy - PCR Confirmed		6.13	
		(4.75)	
Diag. Method PCR		27.86***	
		(3.86)	
Diag. Method RDT		2.14	
		(1.35)	
Diag. Method RDT - PCR Confirmed		10.29	
		(11.04)	
Diag. Method RDT - Slide Confirmed		−9.02***	
		(1.34)	
Diag. Method (simplified) PCR			27.62***
			(3.85)
Diag. Method (simplified) RDT			−0.60
			(1.12)
Observations	9,874	9,874	9,874
R^2	0.53	0.53	0.53
Adjusted R^2	0.49	0.50	0.50

Table R2 (in text Table S1): Sensitivity of estimated effects of temperature and rainfall on *Pf*PR$_{2\text{–}10}$ to controlling for malaria diagnostic method. All columns show regression results for a dependent variable of malaria prevalence among children aged 2–10 (*Pf*PR$_{2\text{-}10}$). Column (1) reproduces the preferred specification from Table S2, column (5) with a single observation that uses loop-mediated isothermal amplification (LAMP) dropped to make for a direct comparison with columns (2) and (3). Column (2) adds a full set of indicators for the dominant diagnostic method observed in each ADM1–year–month:

Microscopy–PCR confirmed, PCR, RDT, RDT–PCR confirmed, RDT–Slide confirmed, and Slide confirmed (Microscopy is the omitted category; a single LAMP observation is dropped). Column (3) replaces these with a simplified method classification that retains only PCR and RDT indicators, again leaving Microscopy as the reference and leaving out the LAMP observation. Standard errors are clustered at the first administrative unit (ADM1) level.

line 25 - no uncertainty for these excess cases

Thank you for this suggestion. We have added confidence intervals, and have removed the results of the 2°C-3°C comparison for east Africa, because we felt on reconsideration that there was too much uncertainty in these results to center them in the Abstract. The revised text now reads (new text in red):

> **Lines 20-35**
> Comparing historical climate reconstructions to counterfactual simulations without anthropogenic climate forcings, we find two-to-one odds that human-caused climate change has increased the overall prevalence of childhood malaria across sub-Saharan Africa since 1901. We estimate that by 2014, human-caused climate change was responsible for an average of 87 excess cases of malaria per 100,000 children ages 2 to 10 (95% confidence interval [CI]: -300, 507), with higher elevation and cooler regions in southern and east Africa experiencing greater increases. Under future climate change, we project that increasing temperatures could marginally accelerate the elimination of malaria in west and central Africa, where the present-day burden is highest, with an average overall reduction of 94 (low greenhouse gas emissions, SSP1-RCP2.6; 95% CI: -497, 160) to 1,890 (high emissions, SSP5-RCP8.5; 95% CI: -4846, 65) cases per 100,000 children in sub-Saharan Africa by the end of the century. However, we find that limiting future global warming to under 2°C (SSP1-RCP2.6) compared to 3°C (SSP2-RCP4.5) could prevent an average of 505 excess cases (95%: -199, 1209) per 100,000 children in southern Africa by 2100.

References

Auffhammer, M. (2018). Quantifying economic damages from climate change. *Journal of Economic Perspectives*, *32*(4), 33-52.

Burke, M., Hsiang, S. M., & Miguel, E. (2015). Global non-linear effect of temperature on economic production. *Nature*, *527*(7577), 235-239.

Carleton, T., & Hsiang, S. M. (2016). Social and economic impacts of climate. Science, 353(6304), aad9837. https://doi.org/10.1126/science.aad9837

Carleton, T., Jina, A., Delgado, M., Greenstone, M., Houser, T., Hsiang, S., ... & Zhang, A. T. (2022). Valuing the global mortality consequences of climate change accounting for adaptation costs and benefits. *The Quarterly Journal of Economics*, *137*(4), 2037-2105.

Childs, M. L., Lyberger, K., Harris, M. J., Burke, M., & Mordecai, E. A. (2025). Climate warming is expanding dengue burden in the Americas and Asia. Proceedings of the National Academy of Sciences, 122(37), e2512350122. https://doi.org/10.1073/pnas.2512350122

Deschênes, O., & Greenstone, M. (2011). Climate change, mortality, and adaptation: Evidence from annual fluctuations in weather in the US. *American Economic Journal: Applied Economics*, *3*(4), 152-185.

Diffenbaugh, N. S., & Burke, M. (2019). Global warming has increased global economic inequality. *Proceedings of the National Academy of Sciences*, *116*(20), 9808-9813.

Diggle, P. J., & Giorgi, E. (2019). *Model-based geostatistics for global public health: methods and applications*. Chapman and Hall/CRC.

Donat, M. G., Alexander, L. V., Yang, H., Durre, I., Vose, R., & Caesar, J. (2013). Global land-based datasets for monitoring climatic extremes. *Bulletin of the American Meteorological Society*, *94*(7), 997-1006.

Efron, B., & Tibshirani, R. J. (1994). *An introduction to the bootstrap*. Chapman and Hall/CRC.

Gething, P. W., Smith, D. L., Patil, A. P., Tatem, A. J., Snow, R. W., & Hay, S. I. (2010). Climate change and the global malaria recession. Nature, 465(7296), 342–345. https://doi.org/10.1038/nature09098

Gould, C. F., Heft-Neal, S., Heaney, A. K., Bendavid, E., Callahan, C. W., Kiang, M. V., Graff Zivin, J., & Burke, M. (2025). Temperature extremes impact mortality and morbidity differently. Science Advances, 11(31), eadr3070. https://doi.org/10.1126/sciadv.adr3070

Harris, I, Osborn, T. J, Jones, P, & Lister, D. (2020) Version 4 of the CRU TS monthly high-resolution gridded multivariate climate dataset. Scientific data 7, 1–18.

Heft-Neal, S., Burney, J., Bendavid, E., Voss, K. K., & Burke, M. (2020). Dust pollution from the Sahara and African infant mortality. *Nature Sustainability*, *3*(10), 863-871.

Heutel, G., Miller, N. H., & Molitor, D. (2021). Adaptation and the mortality effects of temperature across US climate regions. *Review of Economics and Statistics*, *103*(4), 740-753.

Hsiang, S. (2016). Climate econometrics. *Annual Review of Resource Economics*, *8*(1), 43-75.

Missirian, A., & Schlenker, W. (2017). Asylum applications respond to temperature fluctuations. *Science*, *358*(6370), 1610-1614.

Müller, U. K., & Watson, M. W. (2022). Spatial correlation robust inference. *Econometrica*, *90*(6), 2901-2935.

Mordecai, E. A., Paaijmans, K. P., Johnson, L. R., Balzer, C., Ben-Horin, T., de Moor, E., McNally, A., Pawar, S., Ryan, S. J., Smith, T. C., & Lafferty, K. D. (2013). Optimal temperature for malaria transmission is dramatically lower than previously predicted. Ecology Letters, 16(1), 22–30. https://doi.org/10.1111/ele.12015

Mordecai, E. A., Ryan, S. J., Caldwell, J. M., Shah, M. M., & LaBeaud, A. D. (2020). Climate change could shift disease burden from malaria to arboviruses in Africa. The Lancet Planetary Health, 4(9), e416–e423. https://doi.org/10.1016/S2542-5196(20)30178-9

Ortiz-Bobea, A., Ault, T. R., Carrillo, C. M., Chambers, R. G., & Lobell, D. B. (2021). Anthropogenic climate change has slowed global agricultural productivity growth. *Nature Climate Change*, *11*(4), 306-312.

Rode, A., Carleton, T., Delgado, M., Greenstone, M., Houser, T., Hsiang, S., ... & Yuan, J. (2021). Estimating a social cost of carbon for global energy consumption. *Nature*, *598*(7880), 308-314.

Snow, R. W., Sartorius, B., Kyalo, D., Maina, J., Amratia, P., Mundia, C. W., ... & Noor, A. M. (2017). The prevalence of Plasmodium falciparum in sub-Saharan Africa since 1900. *Nature*, *550*(7677), 515-518.

Wooldridge, J. M. (2010). *Econometric analysis of cross section and panel data*. MIT press.

Young, R., & Hsiang, S. (2024). Mortality caused by tropical cyclones in the United States. Nature, 635(8037), 121–128. https://doi.org/10.1038/s41586-024-07945-5

Referee #2 (Remarks to the Author):

Summary

The article examines the relationship between climate change and PfPR2−10, the prevalence of Plasmodium falciparum, a parasite that causes malaria in humans, in children aged 2-10 in sub-Saharan Africa. The authors analyze a dataset of prevalence surveys [Sno+17], which provides a long time span (1900-2014) and subnational spatial resolution, together with the weather station- based climate data CRU-TS. They use panel data regression with fixed effects to identify the influence of temperature and precipitation changes on PfPR2−10. Based on the statistical results, climate model runs are used to attribute historical PfPR2−10 to climate change and to obtain future projections of PfPR2−10 under different climate change scenarios.

The results show a small but statistically significant influence of climate change on PfPR2−10 (Table S2). At the same time, an overall increase in PfPR2−10 due to anthropogenic climate change in the past and in future projections under low and medium greenhouse gas emissions is not significant (Figure 2D). In a more regionally differentiated analysis, the authors find that in some regions rising temperatures reduce PfPR2−10, while in others they increase it.

In my opinion, the topic is relevant, the content seems novel, and many aspects of the analysis appear to be carefully executed. However, further investigation of the validity of the statistical analysis, particularly with respect to uncertainty estimates, is needed to verify the results.

We thank the reviewer for these positive comments.

Major Comments

(A) Uncertainties in the results are estimated using a block bootstrap and clustered standard errors. In both cases, the clusters / blocks are the first administrative units, i.e. subnational regions. Thus, temporal correlations are taken into account, but different spatial regions are implicitly assumed to be uncorrelated.

It seems questionable whether this assumption is correct. From the analysis in the manuscript, we see that the analyzed climate variables have only a small influence on PfPR2−10, i.e. there are other factors with a large influence on PfPR2−10. If such a factor causes an increase in PfPR2−10 not only in one region but in several neighboring regions, the observations will be spatially correlated. If this is indeed the case, and the magnitude of this correlation is large, the reported uncertainties in the manuscript could be severely underestimated.

The correlations need to be analyzed to check the validity of the approach used to estimate uncertainties. Note that correlation here means correlations of the errors ε_{icmt} in equation (1) of the article. At least, average correlations (or box plots of correlations) under different clusterings or connections should be reported. Here are some examples of correlations that could be examined to analyze the dependency structure of the data: Quantify the correlations between ε_{icmt} and $\varepsilon_{i' c' m' t'}$ for

- Different regions of the same country: $i \neq i', c=c', m=m', t=t'$;
- regions of the different countries: $i \neq i'$, $c \neq c'$, $m = m'$, $t = t'$;
- close regions dist(i, i′) < 1000km, $m = m'$, $t = t'$ (with dist() being the distance between the centers of the polygons defining the regions);
- far regions dist(i, i′) ≥ 1000km, $m = m'$, $t = t'$,
- small time lags,e.g,$|m-m'| \leq 3, t=t', i=i'$;
- large time lags,e.g.,$|t-t'| \geq 2, i=i'$;
- same month, different year: $m = m'$, $t \neq t'$, $i = i'$.

These are just examples. With your experience with the data and subject, you may find other structures more relevant to examine.

If, e.g., close regions or different regions of the same country are strongly correlated, the uncertainty estimates (bootstrap, standard errors) have to be adapted using, e.g., different resampling structures for the bootstrap and standard errors with clustering by year, Driscoll Kraay standard errors, or Conley standard errors.

We agree with the reviewer that additional sensitivity analysis regarding our estimates of model uncertainty is warranted. We appreciate the reviewer's careful suggestions in this regard. In response, we have followed the reviewer's direction, implementing the suggested tests (as well as some additional related tests based on the structure of our data) to evaluate the extent to which our model residuals are correlated over space and/or time. We note that because our data form a highly unbalanced panel (e.g., it is not common for two neighboring ADM1 regions to have surveys during the same month and year), these estimated correlations are, in some cases, very noisy estimates of the true structure of the model error and therefore should be interpreted with caution. We therefore reinforce these analyses with some additional tests that have stronger statistical power. We provide our code and a replication file should the reviewer want to explore additional dimensions of the data.

The results of these tests suggest that there exists moderate serial correlation in our model residuals (e.g., mean $\varrho = 0.40$ and lower quartile $\varrho = 0.20$ for temporally consecutive observations), but spatial correlations between residuals from different ADM1 units are not large, particularly for ADM1 pairs that are far apart (e.g., mean $\varrho = 0.07$ and lower quartile $\varrho = -0.17$ for ADM1 units > 1000km apart). However, there are some mixed results, with small but nonzero spatial correlations between ADM1s that are close together (e.g., mean $\varrho = 0.30$ and lower quartile $\varrho = 0.04$ for ADM1 units < 500km apart). Our conclusion, based on these and auxiliary tests described below, is that clustering standard errors at the ADM1 level – accounting for serial correlation in model residuals within an administrative unit but not across administrative units – is the most appropriate choice, particularly due to recent econometrics

research suggesting that conservative clustering of standard errors can be dramatically upward biased (Abadie et al., 2023).[1] However, we now include a section in our Supplement showing results for alternative methods of estimating standard errors, including Conley standard errors that allow for correlation both across spatial units and over time (Figure S17 and Table S6). Of course, if the reviewer interprets the findings of these tests differently – given small and uncertain but definitely nonzero correlation in model residuals across space – we are happy to adjust the main specification of the paper and re-run our historical and future simulations under alternative assumptions (e.g., Conley standard errors up to a 500km radius).

Evaluation of spatial and temporal correlations in residuals

Table R3 below summarizes the results of our calculation of correlations in residuals across various subsets of our data. We follow the approach from a recent paper implementing very similar tests (Schötz, 2025). As suggested by the reviewer, we calculate correlations across observations that are: *(i)* within the same ADM1 unit but from different time periods (all rows in Table R3 labeled as "temporal" in column 1); and *(ii)* from different ADM1 units but within the same time period (all rows in Table R3 labeled as "spatial" in column 1). For temporal correlations, we compute correlations between temporally consecutive observations within up to 3 months of lags, while for spatial correlations we investigate correlations within countries, across countries, within Global Burden of Disease multi-country regions, and based on physical distance. We report mean correlations, as well as the first and third quartiles of the distribution of correlations across different pairs of units.

These tests should be interpreted with care, as our data are highly unbalanced in space and time, often leaving few observations with which to estimate correlations and/or few regional or temporal pairs over which to summarize a distribution of correlations. To ensure interpretability, we restrict analysis to correlations with at least 10 observations (e.g., for spatial correlations we include only ADM1 pairs with at least 10 matching month-year observations). The last column labeled *N* indicates the number of pairs for which sufficient data were available to construct correlations for a given grouping.

[1] We note that the theory and simulation experiments in Abadie et al. (2023) indicate that conservative approaches to clustering can be incredibly biased, but that these results have unclear implications for our and other climate impacts settings where observations within clusters (e.g., month-years) are not sampled randomly. To our knowledge, no extension of this influential paper to the panel data setting yet exists.

Kind	Group	$\bar{\rho}$	Q25	Q75	N
temporal	all	0.18	-0.09	0.48	511
temporal	1 month lag	0.40	0.20	0.65	60
temporal	2 month lag	0.17	-0.06	0.43	33
temporal	3 month lag	0.22	-0.05	0.43	16
temporal	same month, different year	0.22	-0.04	0.53	41
spatial	all	0.24	-0.01	0.54	635
spatial	same country	0.28	0.04	0.56	537
spatial	different country	0.04	-0.18	0.28	98
spatial	same region	0.04	-0.17	0.19	51
spatial	different region	0.04	-0.19	0.30	47
spatial	$<$ 500km	0.30	0.04	0.60	395
spatial	$>$ 500km	0.15	-0.10	0.39	240
spatial	$>$ 1000km	0.07	-0.17	0.33	103

Table R3 (in text Table S5): Summary statistics for temporal and spatial correlations of model residuals. Values show mean correlation ($\bar{\rho}$), interquartile range (Q25, Q75), and sample size (*N*) for different groupings temporally (Kind = "temporal") or spatially (Kind = "spatial"), following ref.[105]. For temporal groupings, correlations between month-year observations within the same ADM1 unit are estimated. For spatial groupings, correlations between month-year sequences of residuals from different ADM1 units are estimated. Here, "region" indicates one of four Global Burden of Disease regions. Means, first quartiles, and third quartiles of the distribution of correlations from each kind of grouping are shown. *N* indicates the number of pairs for which correlations could be estimated; pairs with less than 10 matching observations were dropped.

Table R3 shows that there exists moderate serial correlation in residuals, especially for consecutive observations (row 2) (mean ϱ = 0.40 for consecutive month-years within the same ADM1 unit). In contrast, spatial correlations range from negligible (mean ϱ = 0.04 across ADM1s from different countries or regions) to modest (mean ϱ = 0.15 and ϱ = 0.30 for ADM1s with centroids greater than 500km and less than 500km of one another, respectively). These results imply that serial correlation should be accounted for in model residuals, but that spatial correlation is less clear.

Given that the sample sizes in Table R3 are small, we run two additional tests. First, we estimate a monthly-level auto-regressive distributed lag model in which the residuals from Equation (4), denoted ε_{it} and varying at the ADM1 (*i*) and month-year (*t*) level, are regressed on a set of up to three monthly lags:

$$\varepsilon_{it} = \sum_{s=1}^{3} \rho_s \varepsilon_{i,t-s} + \eta_{it}.$$

We are limited by our data coverage in our ability to estimate many lag lengths. Thus, to investigate longer time lags, as suggested by the reviewer, we additionally collapse our data to the annual level and estimate the following regression:

$$\bar{\varepsilon}_{iy} = \sum_{s=1}^{5} \varsigma_s \bar{\varepsilon}_{i,y-s} + \bar{\eta}_{iy},$$

where $\bar{\varepsilon}_{iy}$ is the average residual for ADM1 unit *i* in year *y*, and where lags *s* are annual instead of monthly. This aggregation leads to far less data loss, allowing us to estimate up to 5 years of lags.

Results are shown in Table R4 below, and indicate robust evidence for serial correlation, consistent with the prior tests. Up to two months of lags are statistically significant in the monthly regressions, while there is evidence of a one-year lag in the annual models. Because these results align with those shown in Table R3 above, we do not include them in the Supplement as they do not provide necessary additional information.

	Monthly auto-regressive lags			Annual auto-regressive lags				
	1 Mn	2 Mn	3 Mn	1 Yr	2 Yr	3 Yr	4 Yr	5 Yr
	(1)	(2)	(3)	(4)	(5)	(6)	(7)	(8)
Res. Lag 1	0.37***	0.36***	0.28***	0.33***	0.31***	0.35***	0.33***	0.36***
	(0.02)	(0.03)	(0.06)	(0.02)	(0.03)	(0.04)	(0.04)	(0.05)
Res. Lag 2		0.16***	0.26***		0.01	0.02	0.05	0.09
		(0.03)	(0.06)		(0.03)	(0.04)	(0.05)	(0.06)
Res. Lag 3			−0.05			−0.0002	0.01	−0.02
			(0.05)			(0.04)	(0.05)	(0.06)
Res. Lag 4							0.01	−0.04
							(0.04)	(0.06)
Res. Lag 5								0.04
								(0.05)
Observations	2,227	855	383	2,521	1,308	803	533	372
R^2	0.13	0.19	0.16	0.10	0.09	0.11	0.13	0.15
Adjusted R^2	0.13	0.18	0.16	0.10	0.09	0.11	0.12	0.14

Table R4. Serial correlation in model errors. The first three columns estimate a monthly auto-regressive distributed lag model with up to three monthly lags. The last five columns aggregate model residuals to the annual level and estimate annual auto-regressive distributed lag models with up to five monthly lags. Sample sizes are shown at the bottom of the table and indicate declining data availability at longer lag lengths.

Second, we flexibly investigate spatial correlation by estimating an empirical variogram of our residuals. A variogram estimates the variance of residuals within groups of observations as a function of the distance between them. This is a standard spatial statistics method to systematically assess spatial correlations across a sample (Cressie, Statistics for spatial data 2015), and complements the distributions of correlations shown above. The key difference here is that the empirical variogram does not condition on time, which constrains available data in

Table R4 – here we simply pool all model residuals and show how variance depends on distance.

Figure R7 shows the resulting variogram for raw values of the outcome variable (a) and model residuals (b). Each panel shows semi-variances on the y-axis, which are defined as $\frac{1}{2}var(\varepsilon_{i+h} - \varepsilon_i)$, where ε_i is the residual for ADM1 unit *i* and ε_{i+h} is the residual for all ADM1 units with centroids within *h* kilometers of ADM1 *i*'s centroid, with *h* on the x-axis. Note that these residuals have no *t* subscripts here as all time periods are pooled when computing spatial correlations in the variogram. When spatial correlation is present, these semi-variances rise with distance, as variance grows with the distance between observations. Panel (a) shows that raw values of our outcome variable – *Pf*PR$_{2\text{-}10}$ – are strongly spatially correlated within a range of about 1,500km. This is expected, given many spatial correlates of prevalence. In contrast, our empirical variogram of model residuals in panel (b) shows essentially a flat line, indicating minimal spatial correlation. While there is some slight increase in variance between points that are ~250km apart and those that are ~800km apart, this increase is small and noisy.

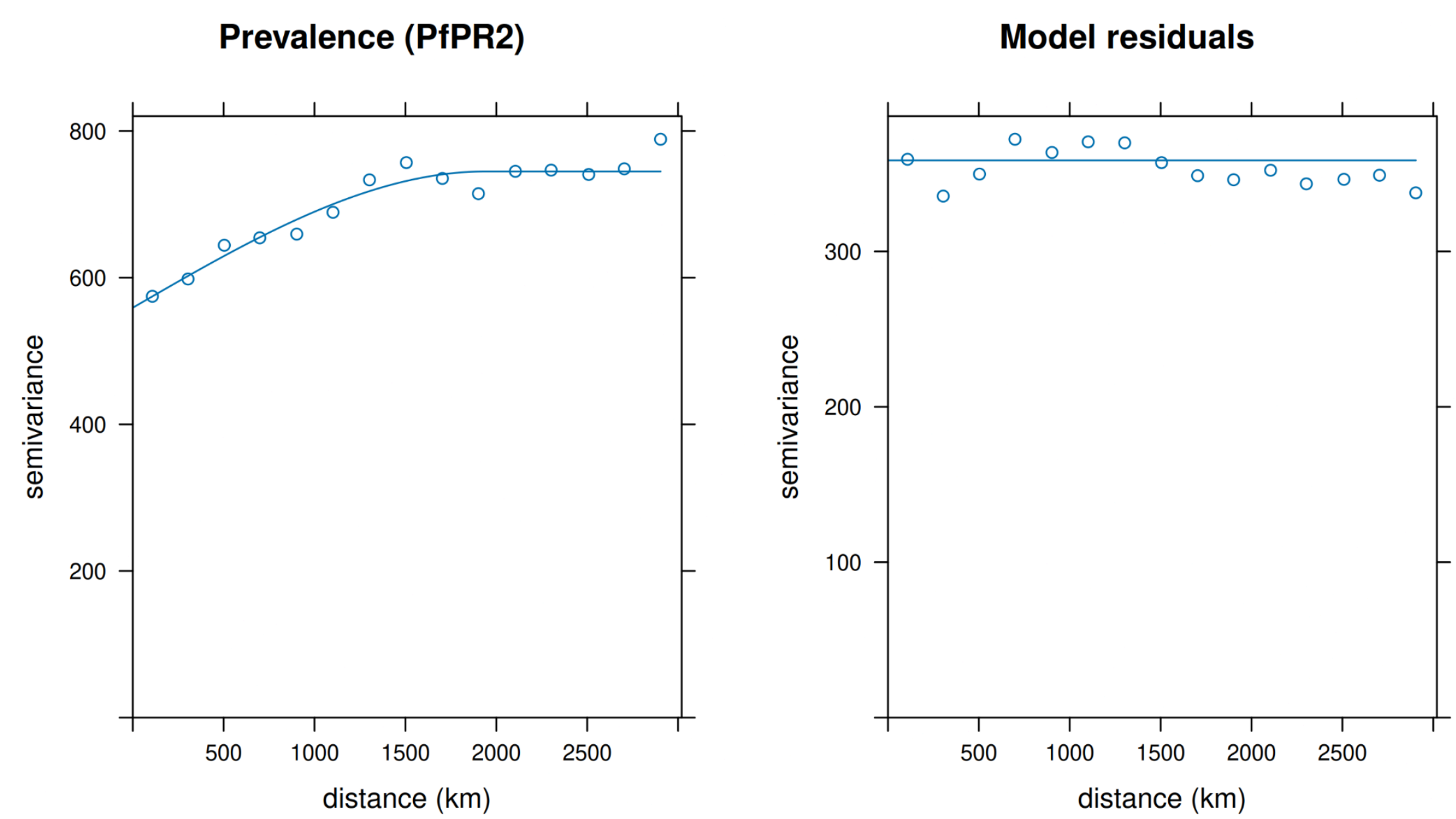

Figure R7 (in text Figure S16): Empirical variogram of malaria prevalence and regression model residuals. Each panel shows estimated semi-variances (*y*-axis) against distance between spatial units (*x*-axis). Semi-variances are defined for each distance *h* as half the variance of the difference between values located within *h* units of each other[106]. When spatial correlation is present, semi-variances rise with distance, as variance grows with the distance between observations. (Left) The variogram for malaria prevalence *Pf*PR$_{2\text{-}10}$. (Right) The variogram for regression model residuals, as estimated following Equation (4). Hollow circles indicate binned scatters while solid lines indicate a spherical variogram model fit. All estimates derived using the `gstat` package in `R`.

Sensitivity to alternative estimates of uncertainty

While we feel that the results above are consistent with our current approach to modeling uncertainty (i.e., ADM1-level clustering which accounts for serial but not spatial correlation in residuals), we acknowledge that such tests are not, particularly in our case, fully conclusive. Thus, we add a sensitivity analysis in which we modify our estimation of standard errors to account for different possible spatial correlations (noting that our main analysis already accounts for the serial correlation we show is present in the data). These alternative uncertainty estimates include: country-by-year clustering (accounting for correlation across ADM1s within a country-year); and Conley standard errors using cutoffs of 200km and 500km (accounting for correlation across ADM1s with centroids up to 500km apart and over years). Figure R8 (now in text as Figure S17) shows that in most cases, the prevalence-temperature relationship remains statistically significant at most temperatures, although confidence intervals do change across estimation methods, as expected. In all cases, an F-test of the joint significance of the linear and second-order polynomial terms rejects the null hypothesis of no effect (*p*-values range from 0.009 in the Conley 500km model to 0.002 in the country-year clustering model).

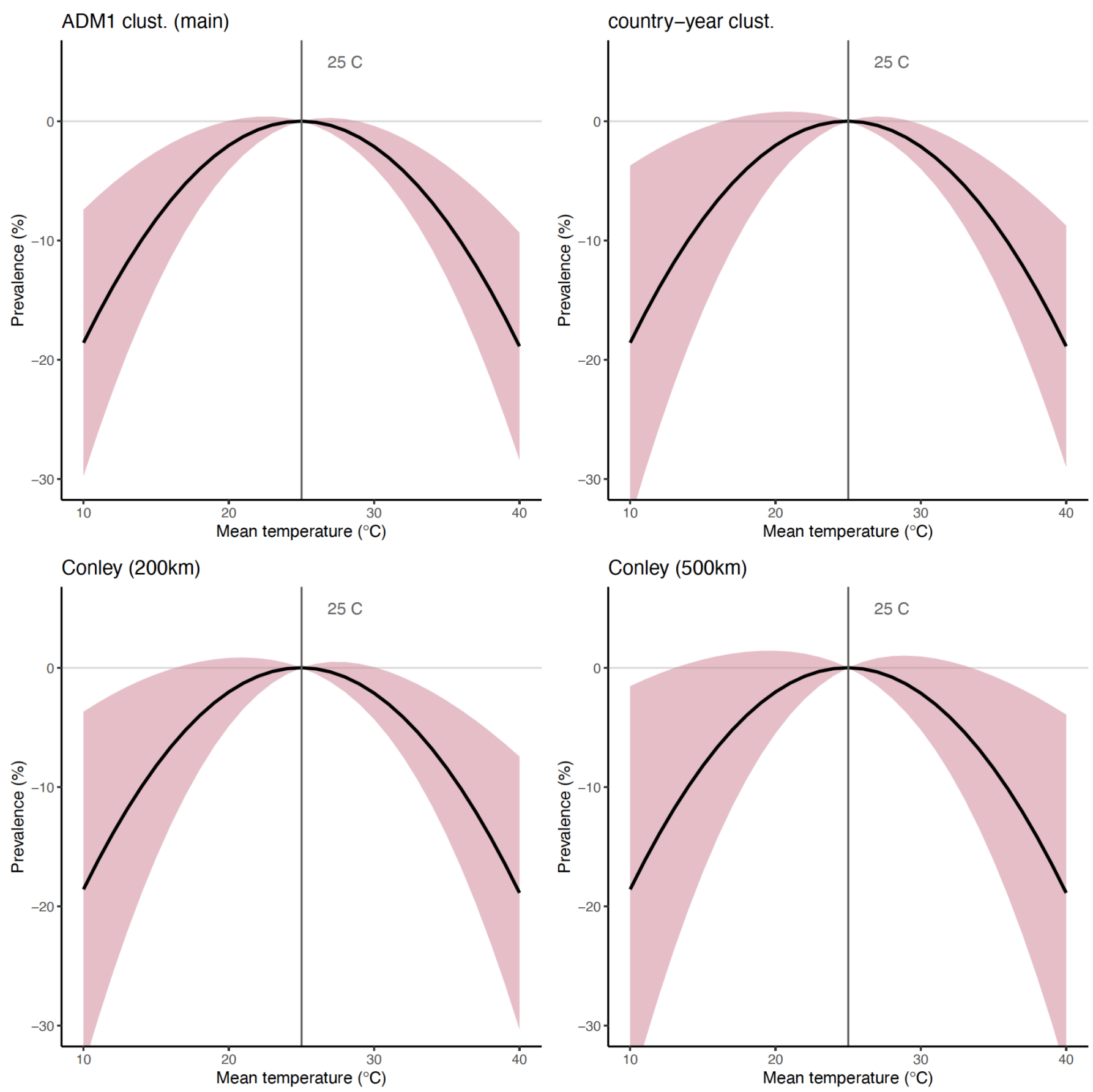

Figure R8 (in text Figure S17): Alternative estimates of model uncertainty in the *Pf*PR$_{2\text{-}10}$-temperature relationship. All panels show the estimated relationship between malaria prevalence for children aged 2-10 and monthly average temperature as estimated following Equation (4). All temperature responses are plotted relative to 25°C, the temperature at which prevalence is maximized. From top-left to bottom-right, standard errors are estimated using: ADM1-level clustering (main specification), country-by-year level clustering, Conley standard errors with a cutoff of 200 km, and

Conley standard errors with a cutoff of 500 km. Tabular results, including associated estimates of precipitation extremes, are shown in Table S6.

Table R5 (in text as Table S6) presents tabular results for these alternative uncertainty estimates for all weather variables, showing that the effects of extreme precipitation are slightly more sensitive to differences in standard error estimation. However, our results for the effects of climate change on malaria prevalence via changes in precipitation throughout the paper are close to zero and highly statistically insignificant. These alternative statistical estimates do not change that key finding.

	Dependent variable:			
	$PfPR_{2-10}$			
	Main spec. (1)	Country-year clust. (2)	Conley (200 km) (3)	Conley (500 km) (4)
Avg temp. (°C)	4.15***	4.15***	4.20***	4.20***
	(1.08)	(1.37)	(1.40)	(1.60)
Avg temp.^{2} (°C)	−0.08***	−0.08***	−0.08***	−0.08***
	(0.02)	(0.03)	(0.03)	(0.03)
Flood	−0.27	−0.27	−0.27	−0.27
	(0.78)	(0.96)	(0.88)	(1.10)
Flood (lag 1)	−0.46	−0.46	−0.46	−0.46
	(0.76)	(0.80)	(0.78)	(1.00)
Flood (lag 2)	1.71**	1.71**	1.70**	1.70
	(0.68)	(0.82)	(0.80)	(1.10)
Flood (lag 3)	0.96	0.96	0.96	0.96
	(0.74)	(0.78)	(0.83)	(1.10)
Drought	0.09	0.09	0.09	0.09
	(0.77)	(0.81)	(0.79)	(1.00)
Drought (lag 1)	−1.28	−1.28	−1.30	−1.30
	(0.80)	(0.86)	(0.85)	(0.87)
Drought (lag 2)	−1.16*	−1.16	−1.20*	−1.20*
	(0.68)	(0.72)	(0.67)	(0.69)
Drought (lag 3)	0.19	0.19	0.19	0.19
	(0.74)	(0.84)	(0.65)	(0.57)
Intvn. 1955-69	−4.80***	−4.80***	−4.80***	−4.80**
	(1.27)	(1.51)	(1.80)	(2.20)
Intvn. 2000-15	−3.35**	−3.35	−3.40*	−3.40*
	(1.61)	(2.15)	(2.00)	(2.00)
Observations	9,875	9,875	9,875	9,875
R^2	0.53	0.53	0.53	0.53
Adjusted R^2	0.49	0.49	0.49	0.49

Table R5 (in text Table S6): Alternative estimates of model uncertainty in *Pf*PR$_{2\text{-}10}$-weather relationships. All columns show regression results for a dependent variable of malaria prevalence for children aged 2-10 (*Pf*PR$_{2\text{-}10}$) and all are estimated following Equation 4. In each column, standard errors are estimated under different assumptions of spatial and/or temporal correlation in model residuals. Specifications are: ADM1-level clustering (column 1, main specification); country-by-year level clustering (column 2); Conley standard errors with a cutoff of 200km (column 3); and Conley standard errors with a cutoff of 500km (column 4). Figure S17 plots the resulting temperature response functions and associated uncertainty.

Changes to the manuscript

We have described and presented the key analyses above in the main text, Methods, and Supplement. Figures R7 and R8 and Table R5 shown above are now Supplementary Figures S16-17 and Table S6. All new analyses are cited and discussed in the main text. The relevant revised main text now reads (new text in red):

> **Lines 613-622**
> We estimate Equation 4 using the `lfe` package in `R`. When reporting regression results directly (e.g., in Table S2), we cluster standard errors ε_{imt} at the ADM1 level to account for serial correlation within the same region over time. When computing bootstrap samples (e.g., shown in Figure 2), we repeatedly re-estimate Equation 4 after block-resampling the full dataset using ADM1-level blocks to account for this same serial correlation. In Supplementary Table S5 and Figure S16, we show that model residuals are serially correlated, but exhibit little to no spatial correlation across ADM1 units, justifying this approach to quantifying model uncertainty (see Methods for details). However, we additionally show sensitivity to alternative methods of capturing uncertainty in Figure S17 and Table S6.

In the Methods section, we have added the following description of this analysis (new text in red):

> **Lines 719-757**
> **Spatiotemporal structure in model residuals**
>
> We evaluate the extent to which our model residuals are correlated over space and time using two tests. First, we calculate correlations between residuals across various subsets of our data, following similar tests in ref.[105]. Specifically, we calculate correlations across observations that are: (i) within the same ADM1 unit but from different time periods; and (ii) from different ADM1 units but within the same time period. For temporal correlations, we compute correlations between temporally consecutive observations within up to 3 months of lags, while for spatial correlations we investigate correlations within countries, across countries, within Global Burden of Disease multi-country regions, and based on physical distance (using ADM1 centroids).

Table S5 reports mean correlations, as well as the first and third quartiles of the distribution of correlations across different pairs of units. Rows labeled "temporal" quantify correlations over time while rows labeled "spatial" quantify correlations over space. We note that these tests should be interpreted with care, as the malaria prevalence data are highly unbalanced in space and time, often leaving few observations with which to estimate correlations and/or few regional or temporal pairs over which to summarize a distribution of correlations. To ensure interpretability, we restrict analysis to correlations with at least 10 observations. The last column in Table S5, labeled *N*, indicates the number of pairs for which sufficient data were available to construct correlations for a given grouping. These results reveal moderate serial correlation in residuals, especially for consecutive observations (row 2) (mean ρ=0.40 for consecutive month-years within the same ADM1 unit). In contrast, spatial correlations range from negligible (mean ρ=0.04 across ADM1s from different countries or regions) to modest (mean ρ=0.15 and ρ=0.30 for ADM1s with centroids greater than 500 km and less than 500 km of one another, respectively).

Second, we use a higher-powered test to flexibly investigate spatial correlation by estimating an empirical variogram of our residuals. A variogram estimates the variance of residuals within groups of observations as a function of the distance between them. This is a standard spatial statistics method to systematically assess spatial correlations across a sample[106]. Figure S16 shows the resulting variogram for raw values of the outcome variable (a) and model residuals (b). Each panel shows semi-variances on the *y*-axis and distance between centroids of ADM1 units on the *x*-axis. When spatial correlation is present, semi-variances rise with distance, as in panel (a). In contrast, panel (b) shows essentially a flat line, indicating little to no spatial correlation in model residuals.

Given these results, we cluster standard errors at the ADM1 level, accounting for correlation in model residuals across all month-years for a given administrative unit. However, in Figure S17 and Table S6, we show sensitivity of our recovered confidence intervals to alternative approaches to standard error estimation, including those that account for spatial correlation (e.g., Conley standard errors).

(B) The analysis is not easily reproducible. The code for the statistical analysis is publicly available, but the processed input data is not (only the raw data before preprocessing). The processed data that is used as input to the regression should also be made available so that the code can directly be executed without having to invest days to redo the preprocessing.

We thank the reviewer for this comment and apologize that this was not included originally. We now include in our replication package the pre-processed dataset used for regression analysis so that analysis can be replicated without computationally expensive geospatial processing of the input data. We include this dataset with our revision, but will make the link live and publicly

available upon publication. Our data availability statement now reads (edited text in red and URL to be filled in upon paper acceptance):

> **Lines 798-802**
> No original data are generated or reported in our study. All data used in analyses, including both malaria and climate data, are freely available and referenced in the Methods. For replication purposes, we make our pre-processed dataset available at {URL}. Raw climate model simulation data are too large for standard public data repositories, but are available upon request.

(C) The data is not distributed uniformly over time and space. Furthermore, the data quality between the beginning of the 20th century and the beginning of the 21st century might also change significantly. To investigate whether this is an issue and to further check the robustness of the results, the regression model (main specification) should additionally be applied to certain subsets the full dataset. In particular, fits for data of an early period $t \leq t0$ and a late period $t > t0$ (for an appropriately chosen threshold year t0) would be interesting. For example, one could show a table similar to table S2, with the main model specification and different data restrictions.

We agree with the reviewer that such subsampling analysis is interesting and potentially informative of data quality differences over time (although it is also important to note that many other things change over time in our sample, including public health interventions that can reshape how prevalence responds to weather). Following the reviewer's suggestion, we conducted a subsampling analysis in which we estimate the response of prevalence to temperature before and after the year 1995, as approximately 50% of the sample observations are from before 1995.

We uncover similar response functions in the two periods, as shown below in Figure R9. The temperature at which prevalence peaks is lower in the first half of the sample, and the impact of warmer temperatures is higher in these early years, although 95% confidence levels overlap substantially. As expected, the smaller sample size increases statistical uncertainty in both subsamples, as compared to the full dataset. Figure R9 is described on lines 144-153 of the main text and presented as Supplementary Figure S8.

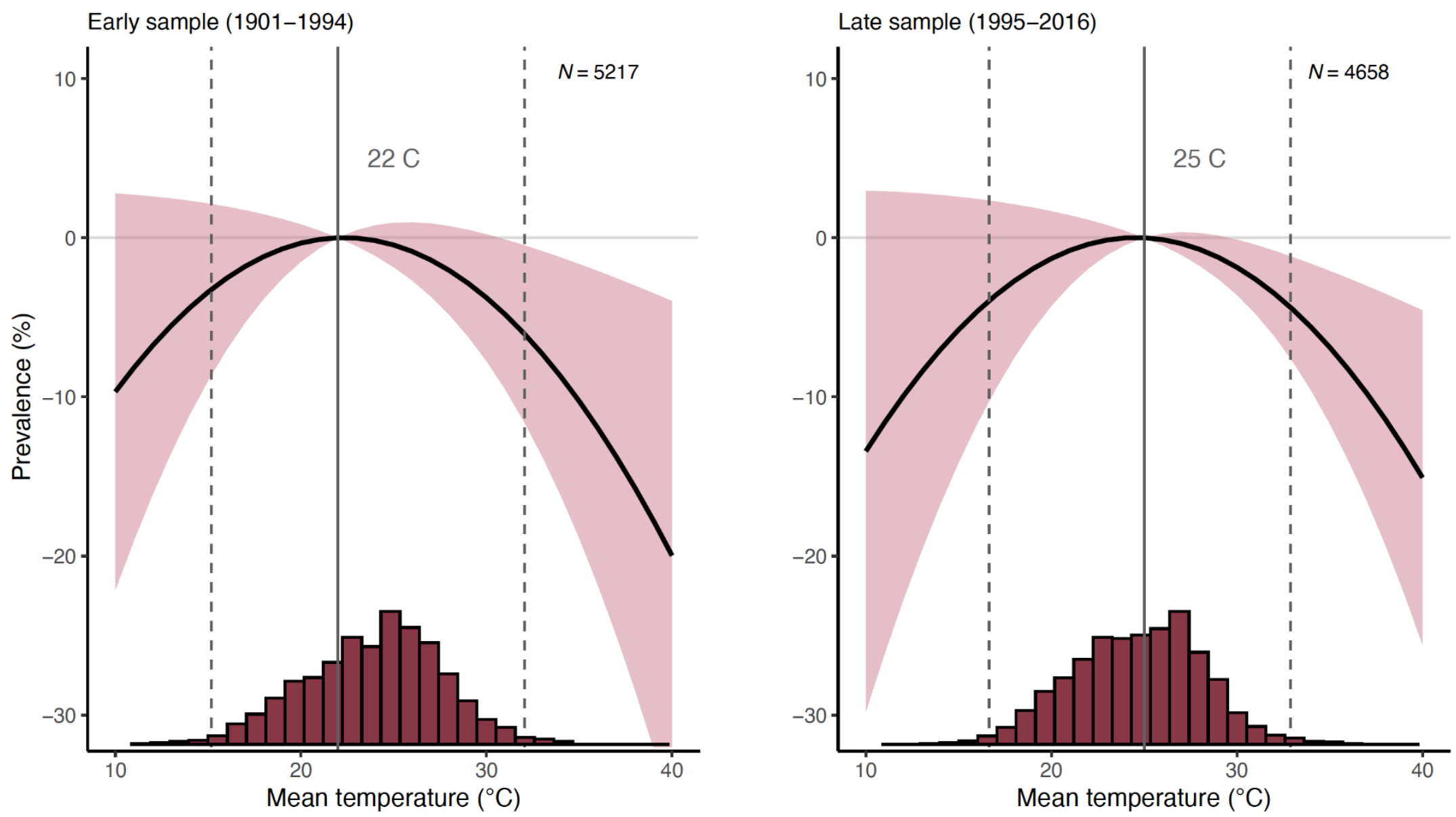

Figure R9 (in text Figure S8): Heterogeneous effects of temperature on malaria prevalence over time. Panels show the estimated relationship between malaria prevalence for children aged 2-10 and monthly average temperature along with a histogram of the associated mean monthly temperature data with the 1st and 99th percentiles of the temperature distributions shown as dashed vertical lines. (Left) The relationship between prevalence and mean monthly temperature for years 1901 to 1995. (Right) The relationship between prevalence and mean monthly temperature for years 1995 to 2014. Both regressions include fixed effects for administrative units and regional seasonality, with standard errors clustered at the administrative level.

Minor Comments

(a) line 399 ”as good as randomly assigned”: This is an overstatement. As remarked above, there might be issues with the dependence structure of the data.

In response to this and other reviewer comments, we have rewritten the methods section detailing the statistical model to more clearly and precisely describe our approach (see lines 514-622 of the Methods). As described above, we respond directly to the reviewer’s concern regarding dependence structure through new residual analysis. In response to this specific concern regarding language, we now write (edited text in red):

> **Lines 533-545**
> This approach is designed to approximate controlled experiments by semi-parametrically accounting for unobservable spatial and temporal confounding factors, isolating variation in the climate system that is less likely to be correlated with other socioeconomic factors[99]. This approach is often referred to as “reduced-form”, as it allows for a plausibly causal interpretation of recovered relationships between socioeconomic conditions and the climate, but it does not easily enable the researcher to isolate individual *mechanisms* linking a changing climate to shifts in outcomes (e.g., mosquito population dynamics or

parasite development rates). However, causal estimates enable counterfactual simulation in which climate is changed and all other factors are held constant; this is the exercise conducted here and in many applications of climate econometric frameworks, including estimating the impacts of climate change on dengue cases[91], international human migration[100], all-cause mortality[90,48], and more.

(b) Figure 2 A-C: The bootstrap uncertainties are depicted by showing all bootstrap runs. But it is rather difficult to read a quantitative uncertainty value from these plots. For B and C, box plots would be more informative. For A, one could plot, e.g., the 5th and 95th percentile as dashed lines.

See response to (d) below.

(c) Figure 2D: This plot shows the 5th and 95th percentiles, but the overlapping shaded areas are confusing and it is hard to distinguish which area belongs to which curve. Maybe plot the borders of the shaded areas in thin (dashed?) lines of the same color as the median line.

See response to (d) below.

(d) Figure 2A: It might be informative to extend Figure 2A similar to [BHM15, Figure 2a] and show a histogram of the observed temperatures below the parabola. The reason is that the parabolic approximation is probably not valid outside (or at the borders of) the observed temperature zone. Furthermore, you could use vertical lines to indicate median temperature (maybe even past and present) of western, southern, central, and eastern Africa.

We appreciate the reviewer's feedback on each panel of this figure. We agree that these changes would help improve clarity and we have implemented each suggestion directly. Specifically, the 5th and 95th percentile of the bootstrapped runs are now shown as dotted lines in panel A and as the whiskers on the new boxplots in panel B. The distribution of monthly average temperature has been added to panel A, along with vertical lines for medians for each region. Panel D has been relabeled as panel E due to the insertion of a new panel addressing a separate reviewer comment. Borders are added to the shaded areas in panel E. We liked the addition of the borders to the shaded areas and also incorporated this change into Figure 3D, Figure 4D, Figure S10, and Figure S13.

The new Figure 2 is reproduced here as Figure R10 (caption changes in red):

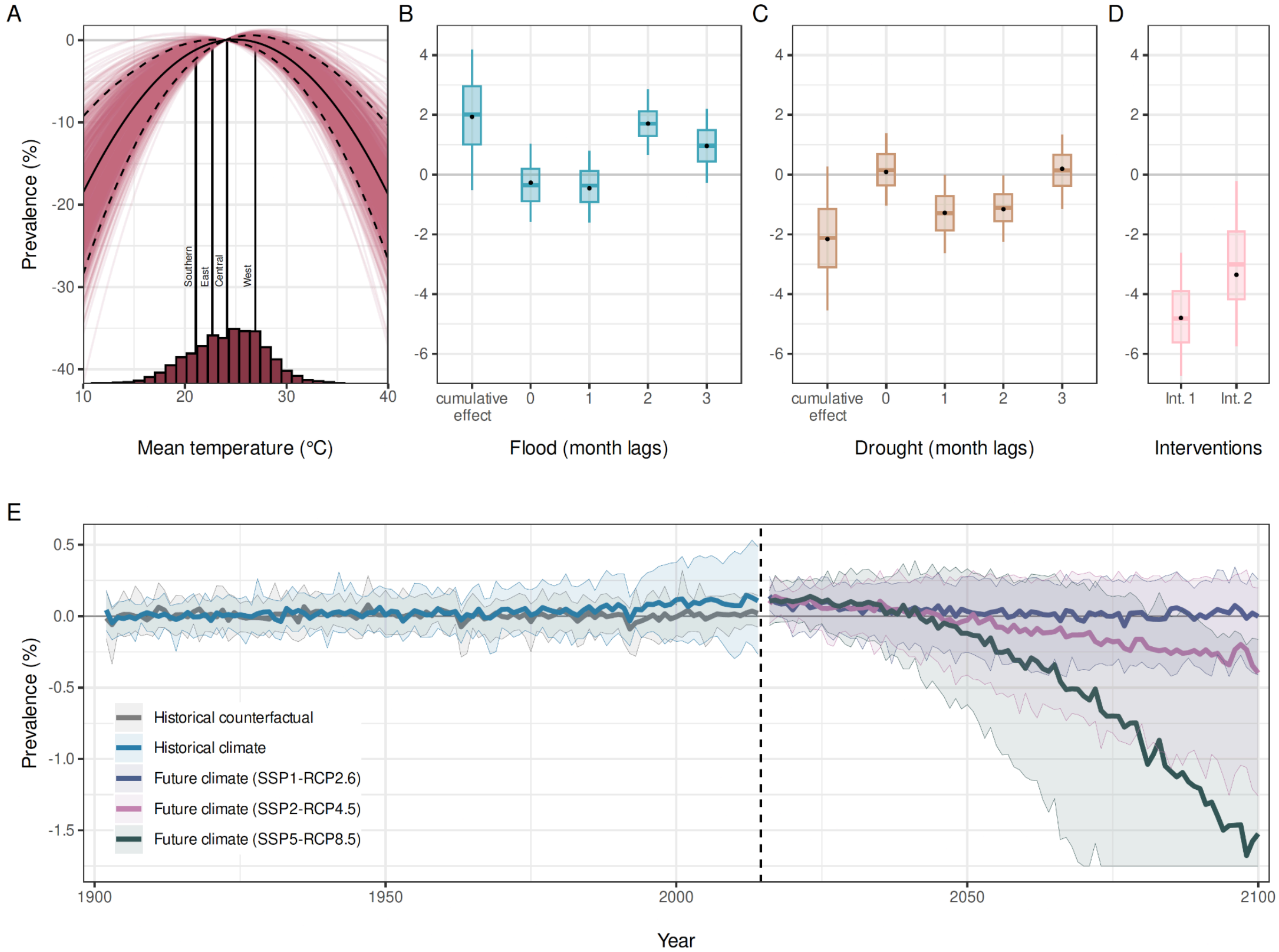

Figure R10 (in text Figure 2): Empirical estimates of prevalence-climate relationships and predictions of climate change impacts from 1901 to 2100. (A) The estimated relationship between temperature and prevalence (point estimate in solid black; 90% CI in dashed black; bootstrapped estimates in red). Histogram shows the distribution of monthly temperatures across the estimation sample, with regional median temperatures shown as vertical lines. (B,C) The effects of extreme precipitation events (flood and drought) in the month they occur (0 lag) and after time has passed (1, 2, 3 month lags), as well as the cumulative impact across the first four months. (D) The effects of two key intervention periods (Int. 1 = 1955-1969; Int. 2 = 2000-2015) during which large-scale malaria prevention programs were implemented across the sub-continent. (B-D) Point estimates from the main model (black) are accompanied by bootstrap estimates shown as boxplots where the box spans the 25th to 75th percentiles, the center line indicates the median, and whiskers extend to the 5th and 95th percentiles (blue, brown, pink). (E) Predicted change in prevalence attributable to anthropogenic climate change in the recent past (real historical climate given in blue; counterfactual without anthropogenic warming in grey) and in the future for low (blue: SSP1-RCP2.6), intermediate (pink: SSP2-RCP4.5), and high (green: SSP5-RCP8.5) emissions scenarios. Thick lines are the median estimates across all 10,000 simulations; shading indicates the 5th and 95th percentiles of this distribution, and is truncated at the lower axis limits for visualization purposes only (the full interval is shown in Figure S13A). Historical estimates are shown relative to an average baseline across 1901 to 1930. Future estimates are shown relative to a baseline across 2015 to 2019, added to the end-of-historical baseline (2010 to 2014). Years with incomplete predictions due to lag effects (1901 and 2015) are not displayed.

(e) line 373: Could you comment on your choice of how you calculate polynomial temperatures? At first glance it seems more intuitive to first aggregate and then calculate the polynomial. In your approach, you start with temperatures that are averages over grid cells, then you make the transformation, and finally average again to the ADM1 region. I.e., the grid resolution seems to have a larger influence in your approach

We thank the reviewer for making clear, along with other reviewers, that we insufficiently detailed and justified our approach to spatial aggregation. To remedy this, we have added a new section of our Methods titled "Spatial data aggregation" (lines 417-513) to explain our approach and its precedent in the climate impacts literature. In short, averaging climatological variables over heterogeneous weather experienced within administrative units before computing nonlinear transformations (such as polynomials) introduces aggregation biases that could threaten the validity of our results. Specifically, doing so would bias the relationship toward a flatter curve, as extreme conditions that generate nonlinear responses are experienced in some localities but are averaged over when aggregating to the ADM1 level. This aggregation bias is detailed in Hsiang (*Annual Review of Resource Economics,* 2016), among other places. We therefore follow a large climate impacts literature (e.g., Schlenker and Roberts, *PNAS* 2009; Rode et al., *Nature*, 2021; Hultgren et al., *Nature* 2025) in aggregating the higher-resolution[2] gridded temperature data *after* computing polynomials in order to avoid this bias. This method is reproduced below.

In addition, we have added to the paper a sensitivity analysis in which we estimate the regression model at the native grid level of the survey and climate dataset, imposing no spatial aggregation. While this model is not preferred due to its statistical properties (detailed below), we show that the recovered prevalence-temperature relationship is very similar to that recovered in our main specification, demonstrating that aggregation biases are unlikely to be driving our results. We reproduce this new Methods section and new sensitivity analysis below.

Our new Methods section on spatial data aggregation now reads (new text in red):

> **Lines 417-513**
> **Spatial data aggregation**
>
> Our statistical analysis is designed to isolate variation in the weather that is uncorrelated with other socioeconomic and/or environmental factors that influence malaria prevalence. As detailed in the next section, we build on a large body of climate econometrics research[45,47,85] to do so, estimating a model that leverages variation over time in weather conditions within the same location. To estimate such a model, we require observations of malaria prevalence covering the same region in multiple time periods. In contrast, the raw prevalence data we

[2] We use the highest resolution climate data that are available and reliable for our entire sample period, beginning in 1901. These data from the Climatic Research Unit (CRU) are 0.5 degree resolution and a monthly time step – while higher resolution data may be preferred, limited station data for Africa especially in early years of the sample make this infeasible.

obtain from ref.[4] are point data observations from individual surveys conducted at different times, such that single geolocations are not observed repeatedly over time. Therefore, we aggregate the point-level data from ref.[4] by averaging *Pf*$PR_{2\text{-}10}$ observations to the first administrative level within each country (i.e., state or province level, or as shorthand, ADM1), using shapefiles provided by the Database of Global Administrative Areas dataset version 3.6 (www.gadm.org). This level of aggregation provides sufficient granularity to capture differences in climate impacts within countries and to control for local heterogeneity in confounders, while ensuring sufficient data coverage within these units. This aggregation scale is also supported by previous work that models this dataset at the same spatial resolution[4]. For robustness, we also show results from a statistical model that does *not* aggregate data, and instead uses the prevalence data at its native resolution (see below for details).

To compute average prevalence values at the scale of the first administrative unit (i.e., ADM1), we use an unweighted arithmetic mean over all prevalence surveys observed in the corresponding ADM1-month. This approach imposes minimal assumptions on the spatiotemporal process of malaria transmission and requires no additional high-resolution data (e.g., population) for use as weights, which are unavailable for sub-Saharan Africa for years as early as 1901. Although prior work aiming to construct comprehensive high-resolution estimates of health outcomes using point data often employs spatio-temporal smoothing methods (e.g., ref.[86]), doing so here would artificially introduce spatial and temporal correlations that could bias recovered regression coefficients and threaten inference[87].

We similarly aggregate monthly 0.5° grid-level weather data to the ADM1-month level. To do so without introducing aggregation biases, we apply methods from previous research demonstrating that it is possible to statistically recover nonlinear relationships that take place at high spatial and temporal resolution, even when the resolution of available outcome data is relatively coarse (i.e., ADM1-month level average malaria prevalence)[47,88,89,90,91]. In our setting, this is achieved by computing nonlinear polynomial transformations of temperature at the grid-cell-by-month level *before* aggregating these values across administrative units. Such an approach ensures the temperature variables used for estimation reflect the full distribution of temperatures experienced across administrative regions of varying sizes and terrains. For example, many of Ethiopia's 12 ADM1 regions include both hot low-elevation zones and cold highlands, such that temperatures can vary substantially within an ADM1 during the same month. We compute second-order polynomials at each grid cell before aggregating across such diverse landscapes to ensure the regressor variables capture both extreme cold and extreme heat, even when they occur simultaneously within the ADM1's boundaries.

To see this method in practice, let $PfPR_{git}$ denote average malaria prevalence in children aged 2-10 in grid cell *g* located within administrative unit *i* during month *t* and let T_{git} indicate temperature observed at the same spatiotemporal scale. Following prior studies recovering local-level quadratic responses between malaria prevalence and temperature[92,93], we assume that prevalence in grid cell *g* in month *t* is a quadratic function of the temperature experienced in that same grid cell and month (noting that we show results relaxing this assumption, such as other nonlinear functional forms and the possibility of temporal lags):

$$PfPR_{git} = \beta_1 T_{git} + \beta_2 T_{git}, \tag{1}$$

where β_1 and β_2 are constant average coefficients. As discussed above, we cannot empirically estimate a model like Equation 1 reliably because we do not observe prevalence over multiple months *t* for the same grid cell *g*. Instead, our empirical specification relies on average ADM1-month level prevalence variables $PfPR_{it}$. Thus, we must aggregate Equation 1 in a manner that allows us to recover the same β_1 and β_2 coefficients that describe the local-level temperature response, and which we would have recovered had we been able to estimate Equation 1 directly. Specifically, average ADM1-month prevalence can be written as:

$$\begin{aligned} PfPR_{it} &= \sum_{g\in i} PfPR_{git}\omega_{gi} = \sum_{g\in i}(\beta_1 T_{git} + \beta_2 T_{git}^2)\omega_{gi} \\ &= \beta_1 \sum_{g\in i} T_{git} w_{gi} + \beta_2 \sum_{g\in i} T_{git}^2 w_{gi}, \end{aligned} \tag{2}$$

where ω_{gi} denotes a grid-by-ADM1 weight. In our setting, we estimate an area-weighted average prevalence value by setting ω_{gi} equal to the share of administrative unit *i*'s area that falls into grid cell *g*, as the lack of high-resolution population data make population weighting infeasible.

Equation 2 shows that a regression of average prevalence in ADM1 unit *i* and month *t* on variables that are ADM1-month weighted aggregates of the nonlinear temperature terms T_{git} and T_{git}^2 will, in expectation, recover the same coefficients β_1 and β_2 that describe the fundamental grid-level relationship described by Equation 1. This same procedure has been used to estimate the relationship between: monthly administrative-level dengue incidence and daily grid-level temperature[91]; annual all-cause mortality and daily grid-level temperature[48,90]; annual country-level crop yields and daily grid-level soil moisture[94]; among many other examples. As in these other cases, our approach mitigates aggregation bias up to the level of the grid resolution of the climate data. While climate and prevalence likely vary across space within each grid cell, we cannot resolve such dynamics here, given the lack of reliable higher-resolution weather data in Africa over the extended time frame of our analysis[77].

We note that one could, alternatively, construct nonlinear weather variables *after* aggregating to the administrative unit, thus estimating:

$$PfPR_{it} = \tilde{\beta}_1 \sum_{g \in i} T_{git}\omega_{gi} + \tilde{\beta}_2 (\sum_{g \in i} T_{git}\omega_{gi})^2, \quad (3)$$

where recovered parameters $\tilde{\beta}_1$ and $\tilde{\beta}_2$ are biased relative to the fundamental relationship in Equation 1 because $(\sum_{g \epsilon i} T_{git}\omega_{gi})^2 < \sum_{g \epsilon i} T^2_{git}\omega_{gi}$, due to Jensen's Inequality.

Thus, we construct a vector of ADM1-month temperature variables by computing nonlinear transformations at the grid level before aggregating across space, following Equation 2. While we could, in principle, follow the same procedure for precipitation, we instead compute drought and flood variables at the ADM1-month level, due to high rates of mismeasurement in grid-level rainfall estimates[77] and due to the likelihood that prevalence-precipitation relationships occur over larger spatial scales than a single grid cell (i.e., water flows through hydrological systems linking precipitation in one location to water availability and prevalence downstream). We show in a robustness exercise detailed below that using grid-level precipitation data generates similar results as our aggregated model, but increases uncertainty, consistent with evidence on rainfall mismeasurement in Africa.

All main results rely on the spatial aggregation procedure described above. However, we additionally estimate an alternative model that leverages the point-level prevalence data directly, introducing no spatial aggregation beyond the resolution of the weather data (0.5°). As we detail below and show in Supplementary Figure S15, the recovered prevalence-temperature results are very similar using these two distinct methods.

Our new sensitivity analysis in which we estimate the statistical model at the native grid-level resolution now reads (new text in red):

Lines 685-718
Estimating a grid-level regression

As described above, our main analysis relies on an ADM1-level regression in which malaria prevalence and weather data are aggregated from higher spatial resolutions to the ADM1 scale. Here, we show the results from an alternative approach, in which point-level malaria prevalence survey observations are matched to corresponding 0.5° resolution CRU climate data grid cells and the regression is estimated at this grid level.

Using these disaggregated data, we estimate a regression model analogous to Equation 4, but modified to fit the spatial scale of the data. Specifically, we estimate:

$$
\begin{aligned}
Pf\mathrm{PR}_{git} = {} & \eta_1 T_{git} + \eta_2 T_{git}^2 \\
& + \sum_{\ell=0}^{L} \lambda_\ell \mathbb{1}\{\mathrm{drought}_{gi,t-\ell}\} + \sum_{\ell=0}^{L} \xi_\ell \mathbb{1}\{\mathrm{flood}_{gi,t-\ell}\} \\
& + \alpha_i + \gamma_{rm} + \sum_{c \in C} \left[\phi_{1c} t + \phi_{2c} t^2\right] \\
& + \delta_1 \mathbb{1}\{\text{intervention 1}\}_t + \delta_2 \mathbb{1}\{\text{intervention 2}\}_t + \varepsilon_{git},
\end{aligned} \tag{5}
$$

where all variables are defined as above for Equation 4. In particular, $Pf\mathrm{PR}_{git}$ represents prevalence reported for a survey located in grid g falling within ADM1 unit i during month t, T_{git} denotes temperature in the same grid and month, and precipitation extremes are captured via dummy variables $\mathbb{1}\{drought_{i,t-\ell}\}$ and $\mathbb{1}\{flood_{i,t-\ell}\}$ that indicate when each grid cell's monthly rainfall total is less than 10% of its long-run month-specific mean (drought) or more than 90% (flood).

Two features render this estimating equation distinct from the main analysis. First, temperature and precipitation variables are matched exactly to the grid cell within which the malaria survey was conducted, such that no aggregation is necessary. This increases the precision of the match between weather and outcome variables. Second, the spatial "fixed effects" denoted by α_i – that is, indicator variables for each of the ADM1 units in our sample – are estimated at a lower spatial resolution (ADM1) than the data itself (grid). This implies that the weather variation used to identify coefficient vectors $\boldsymbol{\eta}$, $\boldsymbol{\lambda}$ and $\boldsymbol{\xi}$ includes both variation over time within an ADM1, but also across survey locations located within the same ADM1. Thus, while this model has the benefit of leveraging high spatial resolution in weather, it potentially suffers from omitted variables bias, as weather conditions in different survey locations may be correlated with other unobservable determinants of malaria prevalence (e.g., access to health care, rates of poverty, proximity to water bodies).

We show in Figure S15 that the malaria prevalence-temperature relationship recovered from estimation of Equation 5 is incredibly similar to that estimated from our main regression model in Equation 4, suggesting that aggregation of the underlying survey data does not influence the key results of the paper. In contrast, the estimated drought and flood coefficients recovered from the grid-level regression are highly imprecise, consistent with substantial measurement error in local-level precipitation datasets in Africa for much of the 20th century[77].

And the associated figure is (Figure S15):

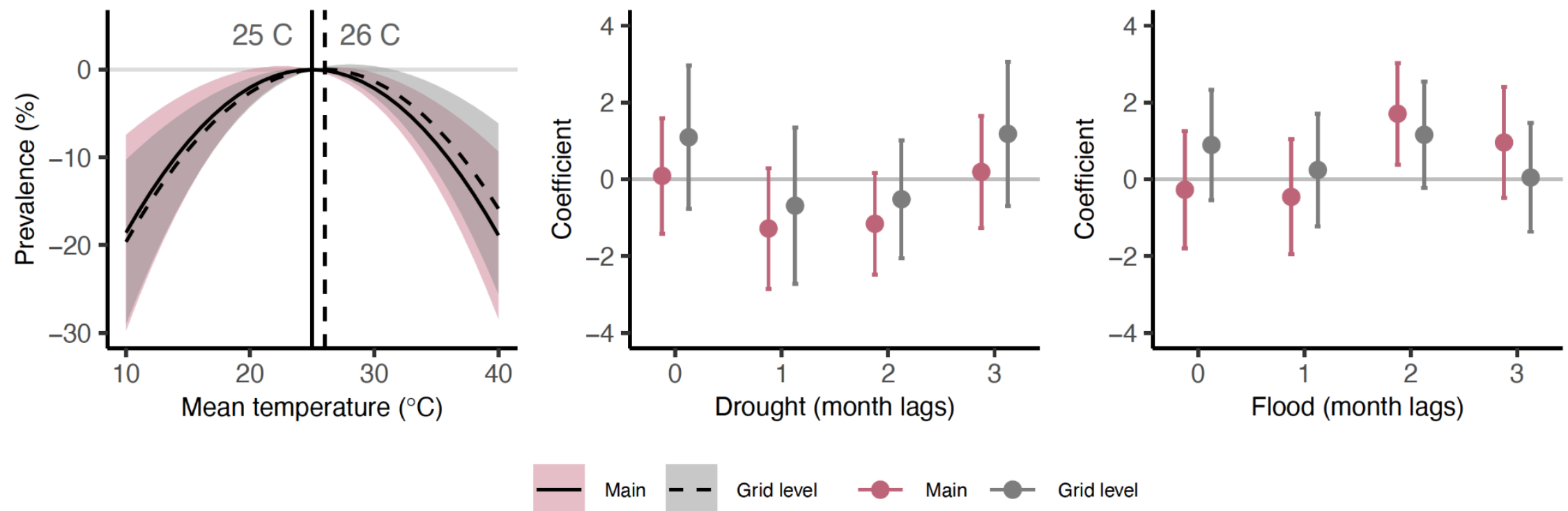

Figure R11 (in text Figure S15): Empirical estimates of climate-prevalence relationships at administrative versus grid cell spatial resolution. Panels show the estimated relationship between malaria prevalence for children aged 2-10 and monthly average temperature (left), drought (middle), and flood (right), estimated separately for our main model specification, aggregated to the first level of administrative division, and for a high-resolution model aggregated to the CRU climate data grid (resolution 0.5°). (Left) The relationship between prevalence and mean monthly temperature for the main ADM1 model (black solid line and pink shading) and high resolution grid-level model (dashed line and gray shading), with shading indicating 95% confidence intervals. (Middle, right) Estimated coefficients and 95% confidence intervals for the effects of drought (middle) and flood (right) events across contemporaneous and lagged months for both model specifications. Positive coefficients indicate higher malaria prevalence associated with extreme climate events. Models are estimated following Equation 4 (main) and Equation 5 (grid level) and all standard errors are clustered at the administrative level (ADM1).

(f) On equation (1):

(i) The main specification should be written out in full, i.e., f, g, and hc, should be replaced by what you actually use. Either completely replace Equation (1) by the explicit main specification or have the main specification additionally to the more general form (which you could then also put into the section Statistical model sensitivity and robustness).

Thank you for this feedback. We now write out what was Equation 1 and is now Equation 4 (due to additions requested by this and other reviewers) fully. We appreciate the many helpful suggestions below, each of which helped make our new notation cleaner, simpler, and more transparent. Equation 4 and the surrounding text now reads (new text in red):

> **Lines 550-564**
> We estimate prevalence as a flexible function of monthly temperature and precipitation variables as follows:

$$
\begin{aligned}
PfPR_{it} = {} & \beta_1 \sum_{g \in i} T_{git}\omega_{gi} + \beta_2 \sum_{g \in i} T^2_{git}\omega_{gi} \\
& + \sum_{\ell=0}^{L} \rho_\ell \mathbb{1}\{\text{drought}_{i,t-\ell}\} + \sum_{\ell=0}^{L} \psi_\ell \mathbb{1}\{\text{flood}_{i,t-\ell}\} \\
& + \alpha_i + \gamma_{rm} + \sum_{c \in C} [\phi_{1c}t + \phi_{2c}t^2] \\
& + \delta_1 \mathbb{1}\{\text{intervention 1}\}_t + \delta_2 \mathbb{1}\{\text{intervention 2}\}_t + \varepsilon_{it},
\end{aligned} \tag{4}
$$

where *g* subscripts denote grid cells, which fall within administrative units *i*, and ω_{gi} are area weights equal to the share of unit *i*'s area covered by grid cell *g*, such that $\sum_{g\in i} T_{git}\omega_{gi}$ equals the area-weighted average monthly temperature across all grid cells falling within administrative unit *i*. Together, parameters β_1 and β_2 recover a quadratic response between prevalence and monthly average temperature. As described above, polynomials are computed before aggregating across grid cells in order to preserve local nonlinearities and avoid aggregation bias. Precipitation extremes are captured by a vector of dummy variables $\mathbb{1}\{drought_{i,t-\ell}\}$ and $\mathbb{1}\{flood_{i,t-\ell}\}$, which indicate whether an administrative unit's monthly rainfall total can be categorized as drought (defined as ≤ 10% of the long-run location- and month-specific mean) or flood (defined as ≥ 90% of the long-run location- and month-specific mean) during month-year $t - \ell$.

(ii) You often have four indices for a variable, e.g., Picmt. For the lagged effects, you subtract l from the third variable, Picm−lt. This is hard to read. Maybe add commas: Pi,c,m,t and Pi,c,m−l,t.

As shown above, we have added commas to the subscripts of Equation 4 (formerly Equation 1) for the lagged effects.

(iii) You write gl. But from the description, I seems like g does not depend on l.

As shown above, we no longer use the notation *g(l)*, as we have followed the reviewer's prior suggestion and written out Equation 4 (formerly Equation 1) fully. We hope it is now clear that the function – now represented as a vector of coefficients ρ and ψ – does depend on the lag length *l*, as the marginal effect of drought and flood differs for each lag length.

(iv) The interventions are indexed by mt. But from the description, I seems like they only depend on the year t.

The reviewer is correct that the intervention periods vary only by year. As shown above, we have updated the notation following the reviewer's comment below (vi) and now use a

month-year time index. We therefore use that index – *t* – as the only subscript on the intervention indicator variables in Equation 4 (formerly Equation 1).

(v) The index i determines the ADM1 region and, by that, also the country. So, for all variables which have an index i, you can remove the index c.

As shown above, we have updated the subscript indices accordingly.

(vi) Consider using t as month-year time index (and keep m as month index only for γrm). Then you can write PfPRi,t = f(Ti,t) + ..., which seems cleaner to me as it better represents the spatio-temporal panel structure of your data.

We appreciate this suggestion and, as shown above, have implemented it.

(g) In your statistical model, previous times only influence the current PfPR2−10 through flood and drought events and you also test robustness against lagged temperature effects (dynamic temperature effects). But from a naive population dynamics perspective, it seems plausible that the prevalence of the previous month(s) could be a strong predictor for the prevalence of the current month. Did you consider such an autoregressive model, i.e. PfPRi,c,m,t = PfPRi,c,m−1,t +...?

We appreciate this suggestion and did indeed consider conducting such an analysis. However, there are two key limitations. The first, and more important, is data availability: lag effects can only be calculated when an ADM1 unit has data in adjacent months, which is not common in most years of the dataset. That is, our panel is highly unbalanced. Using an autoregressive model would force us to drop 7,648 of 9,875 observations (~77% loss of data), significantly reducing our statistical power. Second, autoregressive models are biased when spatial fixed effects are used (Nickell, *Econometrica* 1981), threatening the validity of our regression coefficients. We are ultimately comfortable not including an autoregressive component given these data limitations, and the fact that we do not aim to predict overall prevalence or include these predictions in our findings.

Referee #2 (Remarks on code availability):

The code for the statistical analysis is available. But reproduction seems laborious as only raw data can be downloaded but not pre-processed data, which is the input to the statistical analysis. (Or the description is lacking, so that I was not able to find it).

We thank the reviewer for this suggestion. Please see pages 38-39 in this reviewer response document or the updated "Data Availability" statement for a detailed response. Replication data will be provided with resubmission.

References

Abadie, A., Athey, S., Imbens, G. W., & Wooldridge, J. M. (2023). When Should You Adjust Standard Errors for Clustering? The Quarterly Journal of Economics, 138(1), 1–35. https://doi.org/10.1093/qje/qjac038

Cressie, N. (2015). *Statistics for spatial data*. John Wiley & Sons.

Hsiang, S. (2016). Climate econometrics. *Annual Review of Resource Economics*, *8*(1), 43-75.

Hultgren, A., Carleton, T., Delgado, M., Gergel, D. R., Greenstone, M., Houser, T., Hsiang, S., Jina, A., Kopp, R. E., Malevich, S. B., McCusker, K. E., Mayer, T., Nath, I., Rising, J., Rode, A., & Yuan, J. (2025). Impacts of climate change on global agriculture accounting for adaptation. Nature, 642(8068), 644–652. https://doi.org/10.1038/s41586-025-09085-w

Nickell, S. (1981). Biases in dynamic models with fixed effects. Econometrica: Journal of the econometric society, 1417-1426.

Rode, A., Carleton, T., Delgado, M., Greenstone, M., Houser, T., Hsiang, S., ... & Yuan, J. (2021). Estimating a social cost of carbon for global energy consumption. *Nature*, *598*(7880), 308-314.

Schlenker, W., & Roberts, M. J. (2009). Nonlinear temperature effects indicate severe damages to US crop yields under climate change. *Proceedings of the National Academy of sciences*, *106*(37), 15594-15598.

Schötz, C. (2025). Matters Arising: Spatial correlation in economic analysis of climate change. Nature, 644(8076), E27–E30. https://doi.org/10.1038/s41586-025-09206-5

Referee #3 (Remarks to the Author):

This paper is an ambitious analysis of a large data set on the important question of climate change attribution of malaria burden in Sub-Saharan Africa (SSA). The authors analyze a recently published data set on Plasmodium falciparum clinical prevalence for the last century and for SSA which encompasses most of the global disease burden. The analysis combines a statistical model with observational and simulated climate variables for temperature and precipitation, to consider attribution of change up to 2015 and in simulated future scenarios. Main conclusions are that climate change has had a historical impact, which is regionally differentiated and most evident in East African highlands and South Africa, but small when measured as relative percent change and in comparison to previous reported assessment of intervention effects. Furthermore, future climate change should decrease malaria prevalence in West Africa and only moderately continue to increase it in East and South Africa.

(I am not a climate modeling expert and will therefore only comment on the attribution analysis).

My main concern is whether the question posed here can be addressed in a meaningful way with the malaria survey data, given the spatial and temporal averaging over large areas and across multiple years and transmission seasons. This issue is not convincingly addressed. We are told that the spatial “aggregation scale is supported by previous work that models this data set at the same spatial resolution”. This is not a sufficient argument as the goal of the other study was different, and so were the models. On space, one would want to consider what is the degree of variation in temperature over the areas considered for the main transmission seasons. For example, in regions like the East African highlands the analysis may be smoothing considerable variation in both monthly temperature and malaria prevalence. Perhaps there is no way to do better given the spatial extent that can be considered and the nature of the survey data. But there is no consideration of how the averaging can reduce climate effects. This is a difficult question of course because the global data set itself that is used for the observational climate has also a coarse resolution relative to changes in malaria transmission dynamics in some of these regions.

We thank the reviewer for raising the issue of spatial and temporal aggregation. We address spatial aggregation first here and then we address temporal aggregation below, in response to the reviewer’s second comment.

We agree with the reviewer that spatial aggregation of processes that unfold at higher resolution can introduce bias into statistical models and generate inaccurate projections of long-run climate change impacts. It is clear that our initial manuscript failed to fully investigate and explain how such aggregation influenced our analysis. In response, we have made two substantial changes. First, we add a new Methods section (lines 417-513) that depicts exactly how we conduct spatial aggregation using a method explicitly designed to mitigate aggregation bias. Second, we conduct a fully new analysis involving no spatial aggregation above the resolution of the climate data (referenced on main text lines 685-718 with details in Methods and results in the Supplement). We detail both changes in the following text.

First, we have written a new Methods section detailing how our approach to aggregation ensures that the statistical model is not biased by the need to aggregate malaria prevalence survey data and weather data across space. We show that this method is very common in the climate econometrics literature and has been used to recover local-level climate impacts with spatially-coarse outcome data in settings ranging from dengue (Childs et al., *PNAS* 2025) to crop yields (Hultgren et al., *Nature* 2025) to human mortality (Carleton et al., *Quarterly Journal of Economics* 2022). We apologize that our method of and justification for such aggregation were insufficiently clear in the original manuscript. As noted by the reviewer, and like in all other climate econometric studies, this lack of bias applies only up to the spatial resolution of the climate data grid (0.5° resolution). Thus, while there may be aggregation bias occurring due to processes that unfold below this resolution, we are unable to address these due to limited availability of long-run historical weather data back to 1901 at high-resolution in Africa (Harris et al., 2020), a limitation that we now make clear in the text (see copied text below).[3] However, given that our research question is focused on a large-scale assessment of the effect of long-run climate change across the subcontinent, as opposed to predictive modeling of highly localized prevalence dynamics, we feel that this necessary approximation does not undermine the core contribution of the study.

Our new Methods section on spatial data aggregation now reads (new text in red):

Lines 417-513
Spatial data aggregation

Our statistical analysis is designed to isolate variation in the weather that is uncorrelated with other socioeconomic and/or environmental factors that influence malaria prevalence. As detailed in the next section, we build on a large body of climate econometrics research[45,47,85] to do so, estimating a model that leverages variation over time in weather conditions within the same location. To estimate such a model, we require observations of malaria prevalence covering the same region in multiple time periods. In contrast, the raw prevalence data we obtain from ref.[4] are point data observations from individual surveys conducted at different times, such that single geolocations are not observed repeatedly over time. Therefore, we aggregate the point-level data from ref.[4] by averaging *Pf*$PR_{2\text{-}10}$ observations to the first administrative level within each country (i.e., state or province level, or as shorthand, ADM1), using shapefiles provided by the Database of Global Administrative Areas dataset version 3.6 (www.gadm.org). This level of aggregation provides sufficient granularity to capture differences in climate impacts within countries and to control for local heterogeneity in confounders, while ensuring sufficient data coverage within these units. This aggregation scale is also supported by previous work that models this dataset at

[3] We use the highest resolution climate data that are available and reliable for our entire sample period, beginning in 1901. These data from the Climatic Research Unit (CRU) are 0.5 degree resolution and a monthly time step – while higher resolution data may be preferred, limited station data for Africa especially in early years of the sample make this infeasible.

the same spatial resolution[4]. For robustness, we also show results from a statistical model that does *not* aggregate data, and instead uses the prevalence data at its native resolution (see below for details).

To compute average prevalence values at the scale of the first administrative unit (i.e., ADM1), we use an unweighted arithmetic mean over all prevalence surveys observed in the corresponding ADM1-month. This approach imposes minimal assumptions on the spatiotemporal process of malaria transmission and requires no additional high-resolution data (e.g., population) for use as weights, which are unavailable for sub-Saharan Africa for years as early as 1901. Although prior work aiming to construct comprehensive high-resolution estimates of health outcomes using point data often employs spatio-temporal smoothing methods (e.g., ref.[86]), doing so here would artificially introduce spatial and temporal correlations that could bias recovered regression coefficients and threaten inference[87].

We similarly aggregate monthly 0.5° grid-level weather data to the ADM1-month level. To do so without introducing aggregation biases, we apply methods from previous research demonstrating that it is possible to statistically recover nonlinear relationships that take place at high spatial and temporal resolution, even when the resolution of available outcome data is relatively coarse (i.e., ADM1-month level average malaria prevalence)[47,88,89,90,91]. In our setting, this is achieved by computing nonlinear polynomial transformations of temperature at the grid-cell-by-month level *before* aggregating these values across administrative units. Such an approach ensures the temperature variables used for estimation reflect the full distribution of temperatures experienced across administrative regions of varying sizes and terrains. For example, many of Ethiopia's 12 ADM1 regions include both hot low-elevation zones and cold highlands, such that temperatures can vary substantially within an ADM1 during the same month. We compute second-order polynomials at each grid cell before aggregating across such diverse landscapes to ensure the regressor variables capture both extreme cold and extreme heat, even when they occur simultaneously within the ADM1's boundaries.

To see this method in practice, let $Pf\mathrm{PR}_{git}$ denote average malaria prevalence in children aged 2-10 in grid cell *g* located within administrative unit *i* during month *t* and let T_{git} indicate temperature observed at the same spatiotemporal scale. Following prior studies recovering local-level quadratic responses between malaria prevalence and temperature[92,93], we assume that prevalence in grid cell *g* in month *t* is a quadratic function of the temperature experienced in that same grid cell and month (noting that we show results relaxing this assumption, such as other nonlinear functional forms and the possibility of temporal lags):

$$Pf\mathrm{PR}_{git} = \beta_1 T_{git} + \beta_2 T_{git}, \quad (1)$$

where β_1 and β_2 are constant average coefficients. As discussed above, we cannot empirically estimate a model like Equation 1 reliably because we do not observe prevalence over multiple months t for the same grid cell g. Instead, our empirical specification relies on average ADM1-month level prevalence variables $Pf\text{PR}_{it}$. Thus, we must aggregate Equation 1 in a manner that allows us to recover the same β_1 and β_2 coefficients that describe the local-level temperature response, and which we would have recovered had we been able to estimate Equation 1 directly. Specifically, average ADM1-month prevalence can be written as:

$$\begin{aligned} Pf\text{PR}_{it} &= \sum_{g \in i} Pf\text{PR}_{git}\omega_{gi} = \sum_{g \in i}(\beta_1 T_{git} + \beta_2 T_{git}^2)\omega_{gi} \\ &= \beta_1 \sum_{g \in i} T_{git} w_{gi} + \beta_2 \sum_{g \in i} T_{git}^2 w_{gi}, \end{aligned} \tag{2}$$

where ω_{gi} denotes a grid-by-ADM1 weight. In our setting, we estimate an area-weighted average prevalence value by setting ω_{gi} equal to the share of administrative unit i's area that falls into grid cell g, as the lack of high-resolution population data make population weighting infeasible.

Equation 2 shows that a regression of average prevalence in ADM1 unit i and month t on variables that are ADM1-month weighted aggregates of the nonlinear temperature terms T_{git} and T_{git}^2 will, in expectation, recover the same coefficients β_1 and β_2 that describe the fundamental grid-level relationship described by Equation 1. This same procedure has been used to estimate the relationship between: monthly administrative-level dengue incidence and daily grid-level temperature[91]; annual all-cause mortality and daily grid-level temperature[48,90]; annual country-level crop yields and daily grid-level soil moisture[94]; among many other examples. As in these other cases, our approach mitigates aggregation bias up to the level of the grid resolution of the climate data. While climate and prevalence likely vary across space within each grid cell, we cannot resolve such dynamics here, given the lack of reliable higher-resolution weather data in Africa over the extended time frame of our analysis[77].

We note that one could, alternatively, construct nonlinear weather variables *after* aggregating to the administrative unit, thus estimating:

$$Pf\text{PR}_{it} = \tilde{\beta}_1 \sum_{g \in i} T_{git}\omega_{gi} + \tilde{\beta}_2 (\sum_{g \in i} T_{git}\omega_{gi})^2, \tag{3}$$

where recovered parameters $\tilde{\beta}_1$ and $\tilde{\beta}_2$ are biased relative to the fundamental relationship in Equation 1 because $(\sum_{g \epsilon i} T_{git}\omega_{gi})^2 < \sum_{g \epsilon i} T^2_{git}\omega_{gi}$, due to Jensen's Inequality.

Thus, we construct a vector of ADM1-month temperature variables by computing nonlinear transformations at the grid level before aggregating across space, following Equation 2. While we could, in principle, follow the same procedure for precipitation, we instead compute drought and flood variables at the ADM1-month level, due to high rates of mismeasurement in grid-level rainfall estimates[77] and due to the likelihood that prevalence-precipitation relationships occur over larger spatial scales than a single grid cell (i.e., water flows through hydrological systems linking precipitation in one location to water availability and prevalence downstream). We show in a robustness exercise detailed below that using grid-level precipitation data generates similar results as our aggregated model, but increases uncertainty, consistent with evidence on rainfall mismeasurement in Africa.

All main results rely on the spatial aggregation procedure described above. However, we additionally estimate an alternative model that leverages the point-level prevalence data directly, introducing no spatial aggregation beyond the resolution of the weather data (0.5°). As we detail below and show in Supplementary Figure S15, the recovered prevalence-temperature results are very similar using these two distinct methods.

Second, we conduct an entirely new analysis in which we estimate the regression model at the native grid level of the climate dataset, imposing very minimal spatial aggregation of malaria data (while in theory more than one survey may be present in a given climate grid cell, in practice this is very rare, leading to minimal aggregation across surveys). While this model is not our preferred specification due to its statistical properties, as we discuss below, we show that the recovered prevalence-temperature relationship is very similar to that recovered in our main specification, demonstrating that aggregation biases are unlikely to be driving our results. We reproduce this new Methods section and new sensitivity analysis below.

The results of this new analysis are shown in Supplementary Figure S15 and the added text now reads (new text in red):

Lines 685-718
Estimating a grid-level regression

As described above, our main analysis relies on an ADM1-level regression in which malaria prevalence and weather data are aggregated from higher spatial resolutions to the ADM1 scale. Here, we show the results from an alternative approach, in which point-level malaria prevalence survey observations are matched to corresponding 0.5° resolution CRU climate data grid cells and the regression is estimated at this grid level.

Using these disaggregated data, we estimate a regression model analogous to Equation 4, but modified to fit the spatial scale of the data. Specifically, we estimate:

$$\begin{aligned} Pf\text{PR}_{git} &= \eta_1 T_{git} + \eta_2 T_{git}^2 \\ &\quad + \sum_{\ell=0}^{L} \lambda_\ell \mathbb{1}\{\text{drought}_{gi,t-\ell}\} + \sum_{\ell=0}^{L} \xi_\ell \mathbb{1}\{\text{flood}_{gi,t-\ell}\} \\ &\quad + \alpha_i + \gamma_{rm} + \sum_{c \in C} \left[\phi_{1c} t + \phi_{2c} t^2\right] \\ &\quad + \delta_1 \mathbb{1}\{\text{intervention 1}\}_t + \delta_2 \mathbb{1}\{\text{intervention 2}\}_t + \varepsilon_{git}, \end{aligned} \tag{5}$$

where all variables are defined as above for Equation 4. In particular, $Pf\text{PR}_{git}$ represents prevalence reported for a survey located in grid *g* falling within ADM1 unit *i* during month *t*, T_{git} denotes temperature in the same grid and month, and precipitation extremes are captured via dummy variables $\mathbb{1}\{drought_{i,t-\ell}\}$ and $\mathbb{1}\{flood_{i,t-\ell}\}$ that indicate when each grid cell's monthly rainfall total is less than 10% of its long-run month-specific mean (drought) or more than 90% (flood).

Two features render this estimating equation distinct from the main analysis. First, temperature and precipitation variables are matched exactly to the grid cell within which the malaria survey was conducted, such that no aggregation is necessary. This increases the precision of the match between weather and outcome variables. Second, the spatial "fixed effects" denoted by α_i – that is, indicator variables for each of the ADM1 units in our sample – are estimated at a lower spatial resolution (ADM1) than the data itself (grid). This implies that the weather variation used to identify coefficient vectors $\boldsymbol{\eta}$, $\boldsymbol{\lambda}$ and $\boldsymbol{\xi}$ includes both variation over time within an ADM1, but also across survey locations located within the same ADM1. Thus, while this model has the benefit of leveraging high spatial resolution in weather, it potentially suffers from omitted variables bias, as weather conditions in different survey locations may be correlated with other unobservable determinants of malaria prevalence (e.g., access to health care, rates of poverty, proximity to water bodies).

We show in Figure S15 that the malaria prevalence-temperature relationship recovered from estimation of Equation 5 is incredibly similar to that estimated from our main regression model in Equation 4, suggesting that aggregation of the underlying survey data does not influence the key results of the paper. In contrast, the estimated drought and flood coefficients recovered from the grid-level regression are highly imprecise, consistent with substantial measurement error in local-level precipitation datasets in Africa for much of the 20th century[77].

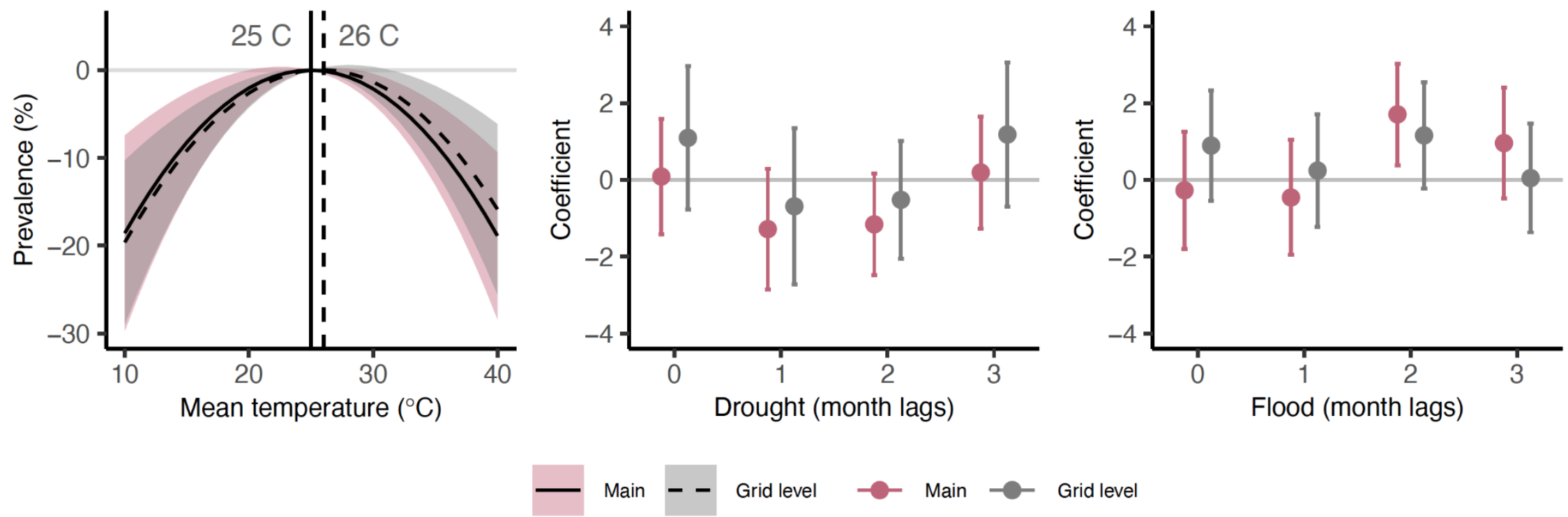

Figure R12 (in text Figure S15): Empirical estimates of climate-prevalence relationships at administrative versus grid cell spatial resolution. Panels show the estimated relationship between malaria prevalence for children aged 2-10 and monthly average temperature (left), drought (middle), and flood (right), estimated separately for our main model specification, aggregated to the first level of administrative division, and for a high-resolution model aggregated to the CRU climate data grid (resolution 0.5°). (Left) The relationship between prevalence and mean monthly temperature for the main ADM1 model (black solid line and pink shading) and high resolution grid-level model (dashed line and gray shading), with shading indicating 95% confidence intervals. (Middle, right) Estimated coefficients and 95% confidence intervals for the effects of drought (middle) and flood (right) events across contemporaneous and lagged months for both model specifications. Positive coefficients indicate higher malaria prevalence associated with extreme climate events. Models are estimated following Equation 4 (main) and Equation 5 (grid level) and all standard errors are clustered at the administrative level (ADM1).

Similarly, when computing the % increase or decrease attributable to climate change, the monthly malaria prevalence is averaged across months and therefore seasons, and over a window of years, for the baseline and the most recent period respectively. The locations where climate variables are expected to have the strongest effects will be necessarily epidemic and seasonal. Isn't this approach averaging peaks and throughs in a way that washes out the interesting signal? Wouldn't the % change be larger if considered only for the months of the transmission season in that location? Asynchrony of seasonality adds a further potential source of smoothing of the signal. One could argue that it is valid to ask the question of attribution at any scale of choice, but I am not clear that this is so and independent from the spatial and temporal scales of malaria population dynamics and temperature variation.

We appreciate the reviewer's insights regarding the potential pitfalls of temporal aggregation – we have now added an analysis of the seasonality of climate change impacts and we feel it substantially strengthens the paper.

In the original manuscript, we focused on displaying the long-run aggregate impacts of climate change on malaria prevalence given that our primary research question pertains to the long-run effects of climate change. However, our statistical model is estimated using high-frequency month-to-month variations in temperature and precipitation and our projected impacts are conducted at the monthly time scale (we temporally and spatially aggregate time series results in the main text for display purposes only). Given this methodology, we can decompose our

estimated impacts of climate change into effects that occur on seasonal time scales. We thank the reviewer for encouraging us to do so; we now include such an analysis in the supplement (we chose not to incorporate this decomposition in our main figures because displaying monthly predictions for the entire historical and future time period visually obscures the long-run trends that are the focus of our analysis). This new analysis reveals that some seasonal effects are indeed large, as hypothesized by the reviewer, and contribute to our understanding of shifting seasonal burdens of malaria. For example, we estimate that by 2014 anthropogenic climate change elevated the prevalence of malaria during the tail end of the malaria season (June and July) in southern Africa by 2.3 percentage points, while the annual average effect in this same region was just 0.6 percentage points. In contrast, warming *lowered* prevalence in west Africa during the lowest point in the transmission cycle (April-May).

We now discuss these insights in the main text (new text in red):

> **Lines 196-207**
> Decomposing these impacts to the monthly level reveals an interplay between space, seasons, and shifting burdens (Figure S12; Table S3). In southern Africa, rising temperatures have extended the potential tail end of the malaria season into the winter months (June and July). This supports the long-standing idea that climate change-driven poleward expansion of vector-borne diseases can emerge from shifting season lengths, and the constraints they impose on endemicity[9,10,65]. In the rest of sub-Saharan Africa, however, climate change impacts generally align with existing seasonality: for example, in lowland central and east Africa, climate change impacts are distributed much more evenly across the year, but have a stronger peak in July and August and a weaker peak in December to February[66]. Conversely, in west Africa, the negative effects of temperature are concentrated in the hottest months (April and May), at the lowest point in the transmission cycle.

The associated Supplementary figure and table read as follows:

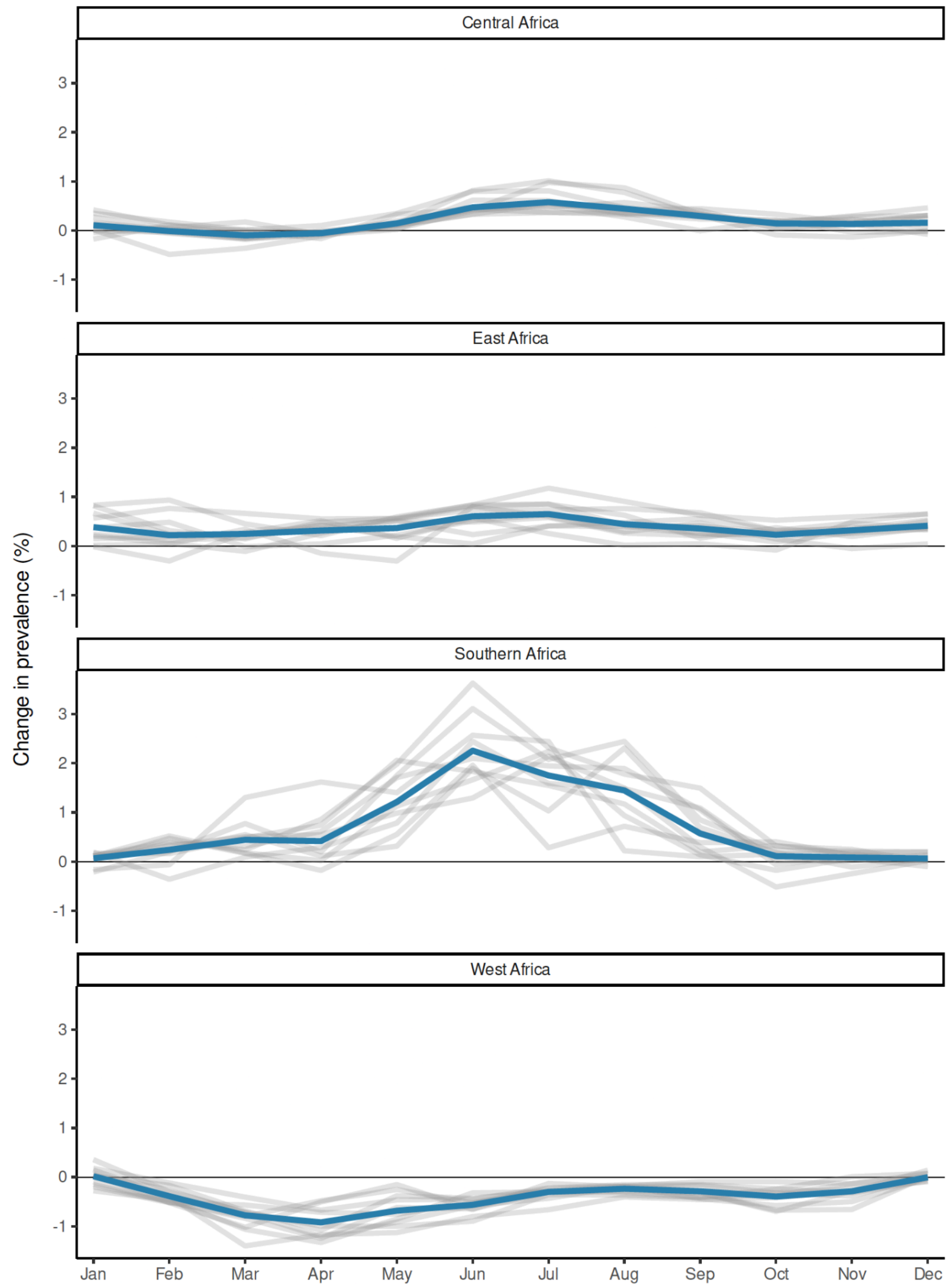

Figure R13 (in text Figure S12): Seasonality in the impacts of anthropogenic climate change on malaria prevalence. Line plots show, for each Global Burden of Disease region, the difference between predicted monthly prevalence under the true historical climate and a counterfactual climate without anthropogenic warming. Positive values indicate that historical climate change elevated malaria prevalence during the indicated month. Impacts are shown as averages by month over the last 5 years (2010-2014) of the historical sample. Each grey line represents one climate model projection, with the blue line showing the median across all models. Associated tabular summaries are shown in Table S3.

Region	Month of Peak Impact	Impact Size (% points)	Average Annual Impact (% points)
Central Africa	Jul	0.58 (-0.13, 1.52)	0.18 (-0.19, 0.61)
East Africa	Jul	0.65 (-0.26, 1.76)	0.33 (-0.14, 0.87)
Southern Africa	Jun	2.26 (0.35, 4.71)	0.63 (-0.04, 1.40)
West Africa	Apr	-0.92 (-2.35, 0.13)	-0.38 (-0.90, 0.02)

Table R6 (in text Table S3): Seasonal peaks in the impacts of historical anthropogenic climate on malaria prevalence. Table summarizes key statistics from line plots in Figure S12. For each Global Burden of Disease region, the month of peak impact indicates the month with the largest absolute difference between predicted prevalence with (historical true climate) and without (counterfactual climate) anthropogenic climate change. Impact size represents the increase or decrease in predicted prevalence due to climate change during the month of peak impact, averaged over the 2010-2014 time period. 95% confidence intervals are shown in parentheses. For comparison, average annual impacts and their confidence intervals are shown in the last column (these values correspond to those shown at the end of the annual time series for each region in Figure 3D).

As a final cautionary note on this analysis: the authors discussed including a parallel analysis for the future, given that there has been previous interest in seasonality-driven future geographic shifts. However, we are extremely hesitant to make these predictions, because – whereas we have recent data and modeling available (published within the last few months) that allows us to know the overall seasonality of malaria transmission today – we do not have the same insights into “baseline seasonality” in the future. Previous work has often treated climate as the only constraint on seasonality, but other factors such as human mobility and interventions are almost certainly implicated as well, and could be substantially different in the future. (As an extreme example, as malaria gets close to elimination in any given community, small climate-driven effects could become mostly irrelevant to the timing of cases within the year.)

Is it possible to focus on particular regions and address whether the survey data allows higher resolution, and/or whether the topography and associated variation of climate parameters justifies the spatial aggregation in that location? CRU data may not allow an answer. I agree with the authors that attribution remains “challenging for the downstream impacts of anthropogenic climate change” including in epidemiology. While the authors do formulate the statistical model in ways that should account for effects other than climate, this is done within the limitation of granularity. I am not convinced the question itself is meaningful at the studied granularity, and would have liked to see either convincing discussion of the limitations, or some analysis of the effects of averaging.

We appreciate the reviewer’s concerns regarding aggregation. We hope that the new text and multiple new analyses detailed above (see pages 52-57 of this document) make clear that our methodology allows for the most granular analysis possible, given the spatial (0.5 degree resolution) and temporal (monthly) data constraints of CRU. As detailed above, we now include new analyses showing our statistical model does not suffer from spatial aggregation bias (Supplementary Figure S15), and that our method does allow for seasonal decomposition of projection results – a decomposition we had not done, but that we now show reveals valuable new insights as suggested by the reviewer (Supplementary Figure S12 and Table S3).

Other comments:

1) A country-specific quadratic time trend is included in the model. What is the justification for this functional form? Or at least its interpretation?

We include these trends in order to control for possibly nonlinear country-specific long-run trends in prevalence that could be correlated with spatially varying long-run trends in precipitation and/or temperature. The functional form is a parsimonious means of capturing long-run trending behavior that could introduce spurious correlation; however, we show in Supplementary Figure S7 and Table S2 that our main results are highly robust to alternative (and more flexible) temporal controls. We provide interpretation and justification for this functional form on lines 593-599 of Methods. Note that Figure S7 is reproduced on page 64 of this response document as Figure R14.

2) What criterium was used to select the final form among the possibilities?

The final form was chosen based on the statistical significance of increasingly restrictive spatiotemporal controls, alongside attempts to maximize degrees of freedom while controlling for key plausible confounders. For example, Global Burden of Disease (GBD) region interactions with month fixed effects were highly statistically significant, indicating that controlling for regional heterogeneity in prevalence seasonality is important to avoid spurious correlations between temperature and prevalence that could arise due to aligned seasonal cycles (this is discussed in more detail in response to the next comment on regional seasonality). In contrast, country-month indicators were statistically underpowered, as the median number of observations per country-month is just 11. Similarly, country trends in prevalence were observed to be nonlinear and to statistically differ from average trends within a GBD region, indicating that country-specific heterogeneity in long-run temporal controls were necessary to fully address the possibility that spatially heterogeneous gradual trends in prevalence coincided with spatially heterogeneous gradual climate change.

3) What is regional seasonality meant to capture?

Global Burden of Disease (GBD) region-by-month fixed effects control for average seasonal patterns in malaria prevalence in different (broad) parts of the continent. If prevalence is seasonal for reasons unrelated to temperature or precipitation patterns – for example, different regional seasonality of vector biology or human behavior – this could confound empirical estimates of the effects of temperature and precipitation if such seasonal controls were omitted. We allow these seasonal controls to vary across regions, reflecting the fact that month-specific seasonality in prevalence and in climatological conditions differs across regions. We describe these controls on lines 584-599 of Methods.

4) We are told in several places that Figure S6 shows that other specifications of the model do not make a difference. I am not clear on how one should consider the evidence in this figure to

reach that conclusion. While the form of the curves is similar, the maximum does move and the uncertainty at the extremes also varies considerably among the panels.

We thank the reviewer for asking for more clarity on this point. Of course, changing spatiotemporal controls can affect the recovered point estimates of the effects of temperature and precipitation on prevalence. This is particularly the case with extremely restrictive specifications, like those that allow each country to have its own monthly fixed effect (i.e., country-specific seasonal controls) in addition to regional year fixed effects (i.e., controls for regionally heterogeneous annual shocks). Thus, we selected the main specification using criteria described above. However, we also show robustness to alternative sets of controls to demonstrate that key results (namely, the overall effect of climate change, which is determined mostly by peak temperature) do not hinge critically on our selection. Please note that what was Figure S6 has been changed to Figure S7 during the revisions. We note that other figures and tables additionally show robustness, not only Figure S7, including Figures S2-6, S18 and Table S2.

Given these many sensitivity analyses, we reach the conclusion that the prevalence-temperature relationship is robust to alternative specifications because the recovered 95% confidence interval from the main specification contains the point estimate of other specifications estimated using alternative spatiotemporal controls (Figure R14). We now discuss this and present this figure in the text as Figure S7. Throughout the paper, we clearly communicate that drought and flood results are more uncertain in the main specification and exhibit more variability across specifications (e.g., see Table S2). Given large uncertainty regarding the impacts of anthropogenic emissions on rainfall patterns, this statistical uncertainty does not have much influence over uncertain long-run estimates of rainfall-induced changes in prevalence.

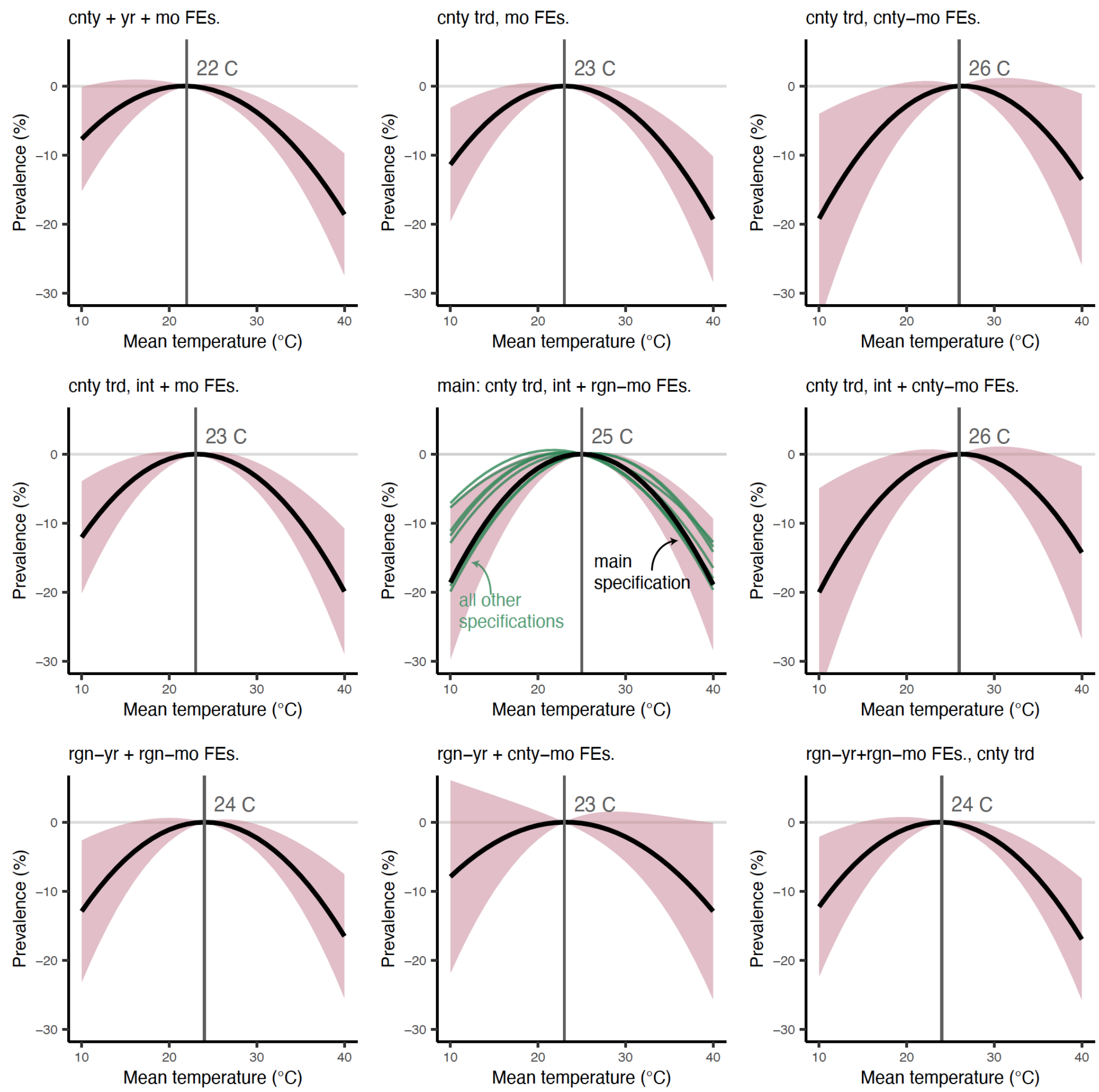

Figure R14 (in text Figure S7): Sensitivity of the *Pf*PR$_{2\text{-}10}$-temperature relationship to alternative spatiotemporal controls. All panels show the estimated relationship between malaria prevalence for children aged 2-10 and monthly average temperature and all include fixed effects (i.e., dummy variables) at the scale of the first administrative unit (i.e., ADM1). All temperature responses are plotted relative to the model-specific temperature at which prevalence is maximized; this peak temperature is indicated in grey text and with a vertical grey line in each panel. From top-left to bottom-right, model controls are: country, year, and month fixed effects; country-specific quadratic time trends and month fixed effects; country-specific quadratic time trends and country-by-month fixed effects; country-specific quadratic time trends and intervention period and month fixed effects; country-specific quadratic time trends and intervention period and region-by-month fixed effects; country-specific quadratic time trends and intervention period and country-by-month fixed effects; region-by-year and region-by-month fixed effects; region-by-year and country-by-month fixed effects; and region-by-year and region-by-month fixed effects and country-specific linear time trends. The preferred specification used throughout the main text is the center panel, which includes country-specific quadratic time trends and intervention period and region-by-month fixed effects. In this center panel, the main specification is shown in black (with pink shaded 95% confidence intervals) and all alternative specifications from other panels are overlaid in green. In all panels, "region" refers to the Global Burden of Disease regional definitions of western, southern, central, and eastern Africa. All standard errors are clustered at the first administrative unit (e.g., province) level.

5) It would be valuable to include some Discussion of malaria control in the context of climate change, decadal and interannual variability, instead of emphasizing the “climate vs. control” view.

We very much appreciate this suggestion. While our primary focus is indeed on reconciling long-term progress towards elimination with climate change impacts, our findings also confirm that climate has intervention-relevant effect sizes at local scales – and therefore, malaria control efforts should be informed by and responsive to weather and climate. To this end, we have added a few sentences on this to our first Results section, in addition to adding panel D to Figure 2, which enables a direct comparison between climate effects on prevalence and historical malaria prevention programs. Edited text reads (new text in red):

> **Lines 157-165**
> Overall, we find a robust relationship between malaria prevalence and climate, including both temperature and rainfall, that is consistent with expectations based on experimental and ecological evidence. Although climate change is unlikely to be the strongest driver of global trends in malaria endemicity (see next section), at a local scale, the month-to-month and year-to-year impacts of climate variability can be comparable in scale to the impacts of major interventions (Figure 2D). These findings suggest that malaria programs should be responsive to climate at local and national scales, and weather-based early warning systems could be useful to anticipate near-term disease dynamics[63,64].

Figure 2 now reads (edited caption text in red):

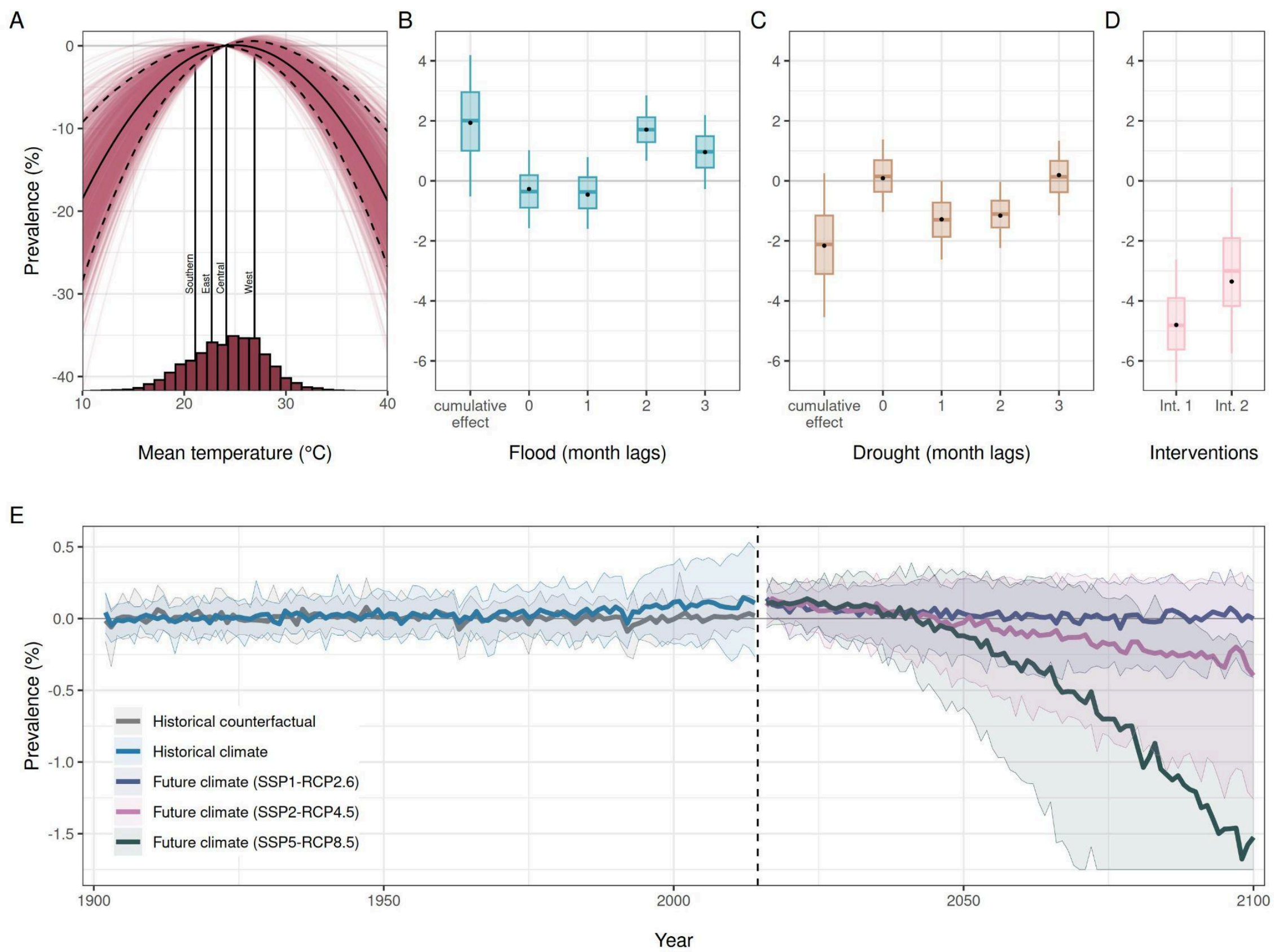

Figure R15 (in text Figure 2): Empirical estimates of prevalence-climate relationships and predictions of climate change impacts from 1901 to 2100. (A) The estimated relationship between temperature and prevalence (point estimate in solid black; 90% CI in dashed black; bootstrapped estimates in red). Histogram shows the distribution of monthly temperatures across the estimation sample, with regional median temperatures shown as vertical lines. (B,C) The effects of extreme precipitation events (flood and drought) in the month they occur (0 lag) and after time has passed (1, 2, 3 month lags), as well as the cumulative impact across the first four months. (D) The effects of two key intervention periods (Int. 1 = 1955-1969; Int. 2 = 2000-2015) during which large-scale malaria prevention programs were implemented across the sub-continent. (B-D) Point estimates from the main model (black) are accompanied by bootstrap estimates shown as boxplots where the box spans the 25th to 75th percentiles, the center line indicates the median, and whiskers extend to the 5th and 95th percentiles (blue, brown, pink). (E) Predicted change in prevalence attributable to anthropogenic climate change in the recent past (real historical climate given in blue; counterfactual without anthropogenic warming in grey) and in the future for low (blue: SSP1-RCP2.6), intermediate (pink: SSP2-RCP4.5), and high (green: SSP5-RCP8.5) emissions scenarios. Thick lines are the median estimates across all 10,000 simulations; shading indicates the 5th and 95th percentiles of this distribution, and is truncated at the lower axis limits for visualization purposes only (the full interval is shown in Figure S13A). Historical estimates are shown relative to an average baseline across 1901 to 1930. Future estimates are shown relative to a baseline across 2015 to 2019, added to the end-of-historical baseline (2010 to 2014). Years with incomplete predictions due to lag effects (1901 and 2015) are not displayed.

6) Also, some discussion of the temporal scales of variation that are not considered here, specifically for effects of climate variability, given that climate change will alter decadal and interannual variation as well as extreme events. These are the time scales at which climate will

most directly impact public health. Given the approach, this kind of variation is not taken into account here.

We appreciate this point, but respectfully disagree with the reviewer's interpretation of our study. Variability within and across years and decades is used in our estimation of prevalence-climate relationships and is accounted for in all simulations of past and future. Interannual and decadal variation due to internal climate variability (i.e., not due to anthropogenic forcing) is captured in both the historical reconstructions and future projections: this is an advantage of using global climate models, instead of e.g., detrending weather station data to create our historical-natural counterfactuals (as some heat mortality studies do). Deeper analysis, such as quantification of the specific impacts of the Pacific Decadal Oscillation, would require new climate modeling that is substantially beyond the scope of this study.

Similarly, short-term extreme weather events are handled in some depth in our study; we account for floods and droughts, which are the short-term climate variability that is expected to most dramatically affect malaria dynamics. Our historical weather data are monthly, and therefore our analysis does omit daily variability within individuals months (i.e., daily temperature extremes), but recent work suggests that this variability has limited explanatory power (Shocket et al., *Nature Communications* 2025). To the extent that climate change is altering daily extremes conditional on monthly average conditions, these changes would not be captured by our results; but, unlike other health outcomes such as heat-related mortality, we do not have any expectation that this should have a pronounced effect on our results.

References

Carleton, T., Jina, A., Delgado, M., Greenstone, M., Houser, T., Hsiang, S., Hultgren, A., Kopp, R. E., McCusker, K. E., Nath, I., Rising, J., Rode, A., Seo, H. K., Viaene, A., Yuan, J., & Zhang, A. T. (2022). Valuing the Global Mortality Consequences of Climate Change Accounting for Adaptation Costs and Benefits*. The Quarterly Journal of Economics, 137(4), 2037–2105. https://doi.org/10.1093/qje/qjac020

Childs, M. L., Lyberger, K., Harris, M. J., Burke, M., & Mordecai, E. A. (2025). Climate warming is expanding dengue burden in the Americas and Asia. Proceedings of the National Academy of Sciences, 122(37), e2512350122. https://doi.org/10.1073/pnas.2512350122

Harris, I., Osborn, T. J., Jones, P., & Lister, D. (2020). Version 4 of the CRU TS monthly high-resolution gridded multivariate climate dataset. Scientific Data, 7(1), 109. https://doi.org/10.1038/s41597-020-0453-3

Hultgren, A., Carleton, T., Delgado, M., Gergel, D. R., Greenstone, M., Houser, T., Hsiang, S., Jina, A., Kopp, R. E., Malevich, S. B., McCusker, K. E., Mayer, T., Nath, I., Rising, J., Rode, A., & Yuan, J. (2025). Impacts of climate change on global agriculture accounting for adaptation. Nature, 642(8068), 644–652. https://doi.org/10.1038/s41586-025-09085-w

Shocket, M. S., Bernhardt, J. R., Miazgowicz, K. L., Orakzai, A., Savage, V. M., Hall, R. J., Ryan, S. J., & Murdock, C. C. (2025). Mean daily temperatures predict the thermal limits of malaria transmission better than hourly rate summation. Nature Communications, 16(1), 3441. https://doi.org/10.1038/s41467-025-58612-w

Referee #4 (Remarks to the Author):

As the authors point out, consideration of the likely impact of climate change on malaria incidence has generated diverse views, often because broad conclusions have been drawn from data collected in a limited area and/or over only a limited period. Thus, the study described in this paper, the most comprehensive attempt to address this issue, that makes use of the unique data set collected by Prof Snow and his colleagues is important and will be of great interest to the malaria community and to others interested in the potential impact of climate change on other vector borne diseases.

The overall conclusions of the study on the likely future impact of climate change on malaria in sub-Saharan Africa are in agreement with the overall perceptions of most malaria epidemiologists with broad experience of sub-Saharan Africa, namely that there will be an increase in incidence of malaria in some areas of East and Southern Africa, especially mountainous areas, and a reduction in incidence in the already hot and dry areas or West Africa. However, it is very helpful to have these suppositions supported by detailed modelling based on results from a very large data set that has been comprehensively analysed, including the analyses of the relative importance of changes in temperature or changes in rainfall.

We thank the reviewer for these positive comments.

This reviewer is not qualified to comment on the details of the methods used for the climate science, statistical and modelling approaches that have been used. However, these are clearly described in the methods section of the paper and thus accessible to other reviewers better qualified to comment on these issues. My comments and suggestions are restricted primarily to issues that might be raised by those involved in the more practical aspects of malaria control and how the findings of this study have relevance to their work.

a. Parasite prevalence and age. The prevalence of malaria parasitaemia in children aged 2-10 years has been used widely in epidemiological studies as a measure of the intensity of malaria transmission in a community because in most parts of sub-Saharan Africa a high proportion of infections occur in children in this age group. However, as the intensity of infection declines or increases, the proportion of all infections in a community in those aged 2-10 years will increase or decline but the overall incidence in the community may remain unchanged. This is a potential risk in using the 2-10 age group as the variable of interest and this could be mentioned.

We appreciate this point. Throughout our paper (including in the title), we have tried to be clear that our data and analysis only addresses childhood malaria. This population experiences the majority of mortality in sub-Saharan Africa, and reductions in parasite prevalence in children do correspond to overall progress towards elimination in the general population (e.g., Giglioli, *Bulletin of the WHO* 1972). However, the reviewer is correct that compensatory dynamics can theoretically emerge from changes in early-life exposure: high prevalence in children can reduce susceptibility both to infection and to severe disease in adults, creating a “see-saw” dynamic between the two. Theory suggests that it is unlikely that this dynamic leads to a fully

compensatory shift (i.e., reductions in childhood malaria should correspond to overall reductions in prevalence most of the time, even if incidence in adults increases; for example, see Pemberton-Ross et al., *Malaria Journal* 2015). Similarly, as we emphasize throughout (e.g., see lines 208-222, 291-306, 307-328), the non-climate drivers of long-term malaria trends are currently orders of magnitude stronger than the climate-driven trends, so these should be the primary determinant of age structure. However, to acknowledge this point, we have added a discussion of this limitation at the top of the Methods, citing the Pemberton study (edited text in red):

> **Lines 362-371**
> The Snow *et al.* data cover all available prevalence surveys, including all age ranges, but were converted by the authors of the original study to a standardized estimate of prevalence in children aged 2-10 (*Pf*$PR_{2\text{-}10}$), using a catalytic conversion Muench model. We chose to use these standardized estimates of childhood malaria prevalence because *falciparum* malaria has the highest mortality in children and pregnant women. The trends that we infer should generally be representative of broader transmission across age groups. In some cases, we note that declines in early-life exposure can lead to smaller increases in incidence in adults[76]; however, these impacts are likely to be small, particularly given that active and passive improvements in malaria prevention, control, and treatment much more directly determine trends in adult malaria risk.

b. Parasite prevalence and clinical malaria. As mentioned above, parasite prevalence in children aged 2-10 years has proved a useful marker for epidemiological studies but the variable of most interest to the malaria control community is the number of cases. The parasite prevalence in children aged 2-10 years is used as the indicator for most of the analyses described in the paper but on page 6 changes in numbers of cases are mentioned. How were these numbers generated – was this an extrapolation from prevalence and, if so, how was this done?

There is no extrapolation being conducted here. We only report prevalence throughout the paper. When we report the number of cases, they are simply rescaled: i.e., we report cases per 100,000 children by multiplying the percent prevalence by 1,000. We now clarify this in the Introduction (edited text in red):

> **Lines 86-91**
> We analyze a recently published dataset with unparalleled resolution and scope (Figure 1), consisting of 50,425 surveys spanning more than a century (1900 to 2015)[4], which we aggregate to 9,875 monthly average values at the first administrative (state or province) level. These data capture a snapshot of population-wide prevalence at a moment in time (i.e., cases of active malaria infection per child; versus, e.g., incidence rate: new cases per child per year).

c. Urbanization. Urbanization is likely to continue to have an increasingly important impact on the malaria burden in the coming decades. Movement of rural residents to cities which have

little malaria transmission, as is happening in many parts of sub-Saharan Africa, sometimes as a result of climate change, can have a major impact on the parasite rate of a district or regional population. In the past, it could be assumed that moving to a city would reduce the chance of a malaria infection but, as the authors mention in the discussion, the worrying emergence of Anopheles stephensi in African cities may change this assumption. Was urbanization taken into account in the climate change predictions in some way?

This is a fantastic question, we thank the reviewer for raising it. Here we first detail how urbanization is controlled for in our main statistical model, but make clear that it is not explicitly investigated as a driver of prevalence or as a modifier of the prevalence-climate relationship. We then present a new analysis we have added to the paper (cited in the main text on lines 152-156 and 335-343, with results shown in Supplementary Figure S9) that probes this relationship further, showing some evidence that rates of urbanization can influence the effects of climate on prevalence.

Like other sources of spatial heterogeneity, average rates of urbanization are currently accounted for as controls in our regression model through the use of semiparametric spatio-temporal control variables (specifically, we include administrative unit fixed effects, region-specific month fixed effects, and country-specific quadratic time trends, as detailed in our Methods lines 573-612). This accounts for different long-run average mosquito ecology in urban and rural settings, and ensures our estimates of the response of prevalence to climate are not conflated by differential rates of urbanization over time or space.

However, this method does *not* allow for the possibility that urbanization is also a mediator on the prevalence-climate relationship. We cannot explicitly solve this problem for our entire analysis, because land cover data back to 1901 are not reliably available. However, we have collected data from the Global Human Settlement Layer Urban Centre Database (European Commission, Joint Research Centre, 2024) to estimate a regression model that distinguishes between climate effects on prevalence in rural versus urban areas for a subset of our sample. To classify each ADM1 as urban or rural, we spatially joined point-level malaria prevalence observations to the Urban Centre Database polygons and assigned an observation as urban if the survey year occurred after the recorded year of birth (YOB) of the overlapping urban center. Because the UCDB YOB data begin in 1975, this definition restricts our analysis to post-1975 observations. We then aggregated these classifications within each ADM1 to compute the share of urban points, and defined an ADM1 as urban when this share was greater than zero.

This analysis, shown in Figure R16 below, reveals that urban areas display substantially *lower* sensitivity to temperature than do rural areas, although urban effects are highly uncertain. This is consistent with the long-standing phenomenon that malaria has primarily been a problem of rural areas, with less range for variability in urban environments, where the force of infection is limited by non-climate factors such as higher income, greater distance from breeding habitat, and better housing quality (Robert et al., *AJTMH* 2003) . In contrast, no statistically significant differences between urban and rural areas can be discerned for the effects of drought and flood.

We now discuss these findings in the first Results section (new text in red):

Lines 152-156

Temperature and flood effects are strongest in rural areas (Figure S9), consistent with negative direct relationships between urbanization and malaria prevalence identified in prior work[60,61], as well as specific risks associated with proximity to natural water bodies or rainfed cropland in rural areas[62]. However, drought effects are highly uncertain, particularly in urban areas.

We also now include this new test in our Discussion section, as follows (edited text in red):

Lines 335-343

Concerns about climate-linked resurgence are also more credible given the ongoing invasion of the *An. stephensi* mosquito, which thrives in cities, has already been reported in several locations in east Africa, and may be able to transmit *P. falciparum* up to much higher temperatures 37°C) than *An. gambiae* can 30°C)[8,73]. While our data provide suggestive evidence that temperature has historically had a *smaller* effect on prevalence in urban areas than in rural ones (Figure S9), if *An. stephensi* were to become a dominant vector across the continent, climate change might become an even more pressing concern[74,75].

Our new analysis is presented in Supplementary Figure S9:

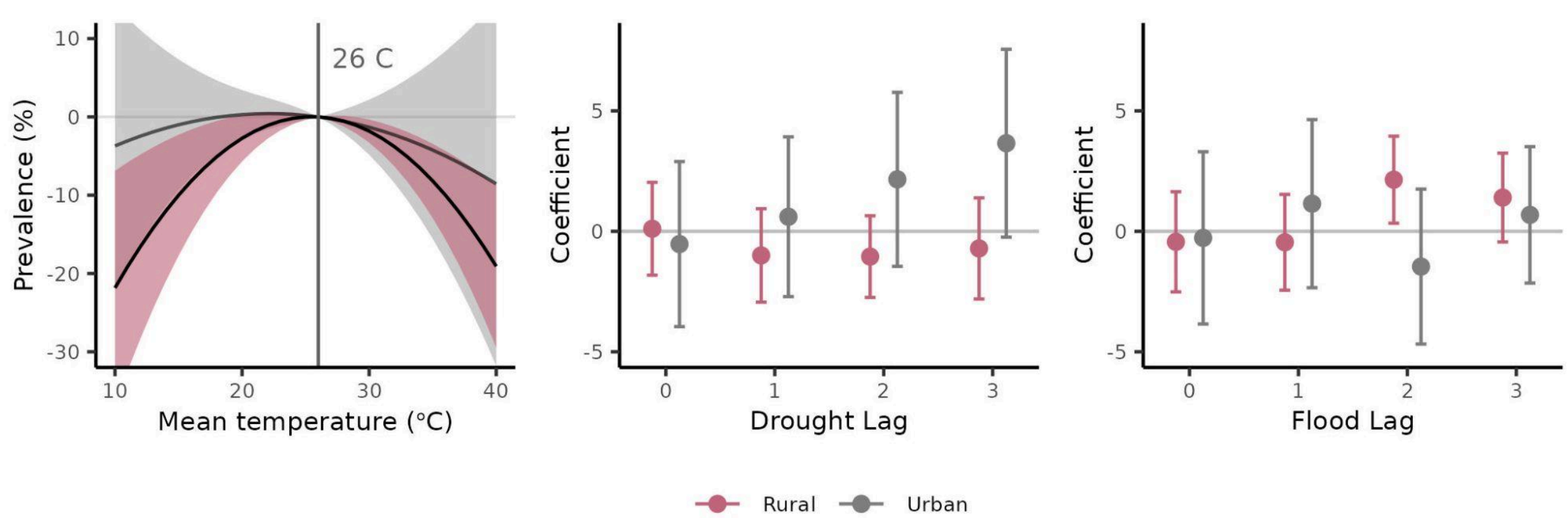

Figure R16 (in text Figure S9). Heterogeneous effects of temperature and extreme precipitation on malaria prevalence by urban and rural status. All panels show the estimated relationship between malaria prevalence for children aged 2-10 estimated separately for rural (pink) and urban (gray) areas. (Left) The relationship between prevalence and mean monthly temperature with shading indicating 95% confidence intervals. (Middle, Right) Estimated coefficients and 95% confidence intervals for the effects of drought (middle) and flood (right) events across contemporaneous and lagged months. Positive coefficients indicate higher malaria prevalence associated with extreme climate events. Model estimates include fixed effects for administrative units and regional seasonality, with standard errors clustered at the

administrative level. Urban–rural classifications are based on the Global Human Settlement Layer Urban Centre Database (European Commission, Joint Research Centre, 2024).

d. Heterogeneity of effect. It is helpful in the analyses undertaken to characterize the major ecologically different regions of sub-S[a]haran with different climate change impacts predicted across a wide region. However, the intensity of malaria transmission can vary substantially over a small area depending on local characteristics, which side of a mountain one lives on, how near a river etc. so that climate change may have a very different impact on communities that are living quite near to each other, and it might be helpful to make this point in the discussion.

We agree with the reviewer that spatial heterogeneity is first-order and we have addressed it in the updated manuscript to the best of our abilities, given data availability constraints. We note that we are constrained by climate data quality across Africa, which is notably poor, especially in the first half of the 20th century (Donat et al. *BAMS* 2013). In addition, counterfactual and future climate simulations from global climate models have coarse spatial resolution, further limiting our ability to speak to some of the hyperlocal characteristics mentioned by the reviewer. However, our priority goal is understanding the large-scale impacts of anthropogenic climate change across the subcontinent over more than a century, while other analyses have been focused on local-level predictions during isolated time periods.

Despite these limitations, our analysis *does* resolve climate change impacts at the scale of first administrative units (ADM1s) across sub-Saharan Africa. Leveraging this spatial scale, we focus throughout the manuscript on decomposing results *within* (not just across) broad ecological regions. We do so in three ways:

Maps of historical and future impacts at the scale of ADM1. While we report summary statistics and show line plots for four broad Global Burden of Disease regions, we also present results at ADM1 scale in the form of maps (Figures 3A, 4A, S11, and S14), revealing large heterogeneity in results even within GBD regions.

Discussion of historical impacts of anthropogenic climate change at local scales. In discussing and interpreting the results in Figures 3A and S11, we describe many patterns that unfold at local scales (see lines 178-195). In particular, on lines 189-195 we discuss that while historical anthropogenic climate change has elevated prevalence across east Africa, changes locally within this region are distributed unevenly along steep elevational gradients, with increases in the Ethiopian highlands and greater Rift Valley but declines in lowland areas in Ethiopia, Sudan, and elsewhere.

Discussion of future impacts of anthropogenic climate change at local scales. Similarly to our discussion of historical impacts of climate change, when describing our future simulations we highlight key localized features within larger GBD regional patterns. For example, on lines 250-253, we discuss how different results are in cooler parts of the Ethiopian highlands than in other parts of east Africa, as well as the differences between coastal southern Africa and the rest of the southern region. We highlight on lines 261-269 that impacts of achieving mitigation in

line with the Paris Agreement are estimated to generate localized benefits that are an order of magnitude larger than their regional averages.

e. Magnitude of the climate change effects. Overall, the expected changes in parasite prevalence, although statistically significant and important, are relatively modest in relation to the changes that are likely to occur as a consequence of other factors. For example deployment of a highly effective malaria vaccine could reduce malaria prevalence in 2-10 from 50% to 5% whilst invasion of many large African cities by An. stephensi could have a major deleterious effect. It would be helpful to stress this it in the discussion for the benefit of the general reader so that the relatively modest, although important, changes likely to be induced by climate change can be put into a broader perspective of what is likely to happen to the incidence of malaria in sub-Saharan Africa in the coming decades. It is important that the predicted changes in malaria incidence are presented in an appropriate way in general discussions on the need for mitigation of climate change.

We fully agree with the reviewer. We have ensured this sentiment is expressed clearly throughout the manuscript, as detailed below.

In the description of our results on estimated malaria-climate relationships, we write (new text in red):

> **Lines 157-165**
> Overall, we find a robust relationship between malaria prevalence and climate, including both temperature and rainfall, that is consistent with expectations based on experimental and ecological evidence. Although climate change is unlikely to be the strongest driver of global trends in malaria endemicity (see next section), at a local scale, the month-to-month and year-to-year impacts of climate variability can be comparable in scale to the impacts of major interventions (Figure 2D). These findings suggest that malaria programs should be responsive to climate at local and national scales, and weather-based early warning systems could be useful to anticipate near-term disease dynamics[63,64].

At the end of the results section describing historical impacts of anthropogenic climate change, we write (text unchanged):

> **Lines 208-219**
> While these effects are meaningful, we caution that they are also far smaller than the reduction achieved through healthcare, mosquito nets, vector control, and economic development: previous work with the same dataset has estimated a reduction since 1900 of 16 *p.p.* (that is, a continent-wide decline in average *Pf*PR$_{2\text{-}10}$ from 40% in 1900-1929 to 24% by 2010-2015[4]), while our estimates of historical climate change-attributable changes rarely exceed 1.5 *p.p.* for any individual administrative region. Additionally, we estimate that average reductions in prevalence realized during the Global Malaria Eradication Program

(1955-1969; estimated reduction averaged over the entire period: -4.80 *p.p.*) and recent programs like Roll Back Malaria and the Global Technical Strategy (2000-2014; estimated reduction averaged over the entire period: -3.35 *p.p.*) were substantially larger than the cumulative effects of anthropogenic climate change (Table S2).

Additionally, in the Discussion we write (text unchanged):

Lines 291-297
Spanning multiple centuries, our analysis is the most comprehensive look to date at the impact of climate change on any infectious disease, and brings new clarity to a decades-long debate in malaria research. Whereas some work has questioned the plausibility that overall declines in continent-wide prevalence would conceal a climate-linked increase[4,19], the 0.087 percentage point increase in $Pf\mathrm{PR}_{2\text{-}10}$ that we attribute to historical anthropogenic climate change could easily be masked by the nearly 200-fold greater overall reduction observed across sub-Saharan Africa over the same period.

Similarly, our Discussion emphasized the possible impacts of the invasion of *Anopheles stephensi* and the potential for this to dramatically change the impacts of climate change. However, one area that the reviewer identified that we did not discuss in sufficient depth is the possible impact of interventions into the future. We have added this to the Discussion, completing a broader discussion of the subject (edited text in red):

Lines 314-330
In spite of climate change, elimination campaigns have already achieved substantial reductions in malaria endemicity over the last century (and not just in sub-Saharan Africa[68]). Our study underscores that the combined benefits of disease surveillance, healthcare, vector control, and economic development can easily counter-balance climate change impacts in most places—and that malaria elimination within the next generation remains plausible, even in the face of climate change. Another recent study found that passive changes in climate, land use, and development will lead to modest reductions in malaria prevalence over the next 25 years, but with 80% effective coverage of chemotherapy, indoor residual spraying, and insecticide-treated nets, malaria could be nearly eliminated in sub-Saharan Africa by 2050[69]. In the last few years, the odds of success have become substantially higher thanks to the new RTS,S and R21 malaria vaccines: a four-dose R21 schedule could prevent between one-third and one-half of all malaria cases in children under 5[70]. Even in places where climate change is increasing malaria transmission, the combined use of classic and new interventions should have a significantly greater effect—provided that these interventions are able to continue.

At the time of writing, progress towards malaria elimination hangs in the balance, as global health financing faces an unprecedented moment of resource scarcity.

Lines 338-342

While our data provide suggestive evidence that temperature has historically had a *smaller* effect on prevalence in urban areas than in rural ones (Figure S9), if *An. stephensi* were to become a dominant vector across the continent, climate change might become an even more pressing concern[74,75].

References

Donat, M. G., Alexander, L. V., Yang, H., Durre, I., Vose, R., & Caesar, J. (2013). Global land-based datasets for monitoring climatic extremes. Bulletin of the American Meteorological Society, 94(7), 997-1006.

European Commission, Joint Research Centre, Melchiorri, M., Mari Rivero, I., Florio, P., Schiavina, M., Krasnodebska, K., Politis, P., Uhl, J., Pesaresi, M., Maffenini, L., Sulis, P., Crippa, M., Guizzardi, D., Pisoni, E., Belis, C., Oom, D., Branco, A., Mwaniki, D., Githira, D., Kochulem, E., Tommasi, P., Carioli, A., Ehrlich, D., Kemper, T. and Dijkstra, L., Stats in the City – the GHSL Urban Centre Database 2025, Publications Office of the European Union, Luxembourg, 2024, https://data.europa.eu/doi/10.2760/3046391, JRC139768.

Giglioli, G. (1972). Changes in the pattern of mortality following the eradication of hyperendemic malaria from a highly susceptible community. Bulletin of the World Health Organization, 46(2), 181–202.

Pemberton-Ross, P., Smith, T. A., Hodel, E. M., Kay, K., & Penny, M. A. (2015). Age-shifting in malaria incidence as a result of induced immunological deficit: A simulation study. Malaria Journal, 14(1), 287. https://doi.org/10.1186/s12936-015-0805-1

Robert, V., Macintyre, K., Keating, J., Trape, J.-F., Duchemin, J.-B., Warren, M., & Beier, J. C. (2003). Malaria transmission in urban sub-Saharan Africa. The American Journal of Tropical Medicine and Hygiene, 68(2), 169–176.

Dear Dr. Fehervari,

Thank you for the chance to make some final revisions to our manuscript based on your feedback and the comments of Reviewers 2 and 3.

In this revision we have:

- At your request, included a comparison of our findings (based on the CRU climate dataset) with another climate data source (ERA5), which shows that our key findings are highly robust to the source of input climate data; we have also ensured that climate data are aggregated with correct area-weighting based on grid cell size.
- At Reviewer 2's request, we have replaced our main model with an estimator that better captures statistical uncertainty, and included several more sensitivity analyses demonstrating the robustness of the statistical model to different specifications and error structures.
- At Reviewer 3's request, we have added a more nuanced discussion of the history of debates around elevational shifts in malaria in East Africa, and a greater discussion of alternative timescales where weather and climate prediction can inform malaria control.
- We have made some final light edits to text and figures to ensure clear and accurate presentation of results.

Please find a point-by-point explanation of these changes below, as well as a copy of the manuscript with track changes marked in red.

As a final note in service of transparency: in the process of reproducing every analysis in the paper based on our new main model, we uncovered a small inconsistency in the presentation of results in the prior manuscript. Specifically, while all in-text statistics summarizing central moments of the probabilistic climate simulation results used the *mean*, the figures displayed the *median* (e.g., central moments in Figure 2E). We have now made these consistent by updating figures to show *means*, which now align with the in-text statistics. Of course, in both the in-text statistics and figures, we additionally report confidence intervals alongside central moments.

Thanks so much, and we hope that this revision satisfies all outstanding concerns.

Editor

Following further discussion with the Refs. they also indicated that the work would be made more robust by including reanalysis with an additional dataset such as ERA-5. It would be helpful in our decision making if you could provide a response to the remaining issues including the feasibility of reanalysis using ERA-5.

Thank you for this suggestion. We have added a sensitivity analysis in our revision that demonstrates that our results are highly robust to the use of ERA5 historical weather data. We note that the ERA5 product starts in 1940, and so when executing this test, we necessarily discard the first 40 years of malaria prevalence data (Hersbach et al. 2023). Thus, the

combination of different malaria data and different climate data should lead to some expected differences.

Three sets of results show robustness to the use of ERA5 weather data. First, in Figure R1 (below) we show that CRU and ERA5 historical weather data, aggregated to our analysis scale of administrative unit by month resolution, are strongly positively correlated within our estimation sample, although this correlation is much stronger for temperature (R^2=0.93) than for precipitation (R^2=0.69); this is expected, given known challenges in accurately representing precipitation where station data are sparse (Harris et al., 2020).

Second, in Table R1 (now Table S1 in the Supplementary Information) we show regression coefficients for our main statistical model using three specifications: our main specification using CRU over the full sample (column 1); a subset of our main specification that uses CRU weather data but limits to years after 1940 to match the available sample for ERA5 (column 2); and a replication using ERA5 for years after 1940. All relevant coefficients are similar in sign and magnitude, with very similar statistical significance. Figure R2 (now Figure S9 in the Supplementary Information) represents these results visually, showing that the resulting prevalence-temperature response function and the temporal dynamics of prevalence responses to drought and flood events are very similar between regressions estimated using CRU data and those estimated using ERA5. In particular, the response of prevalence to high temperatures, which are most relevant for future climate change projections, is virtually identical across the two sets of results, and rainfall events continue to have uncertain but very similar dynamic effects on prevalence, with consistent statistically significant effects of floods on prevalence with a 2-3 month lag.

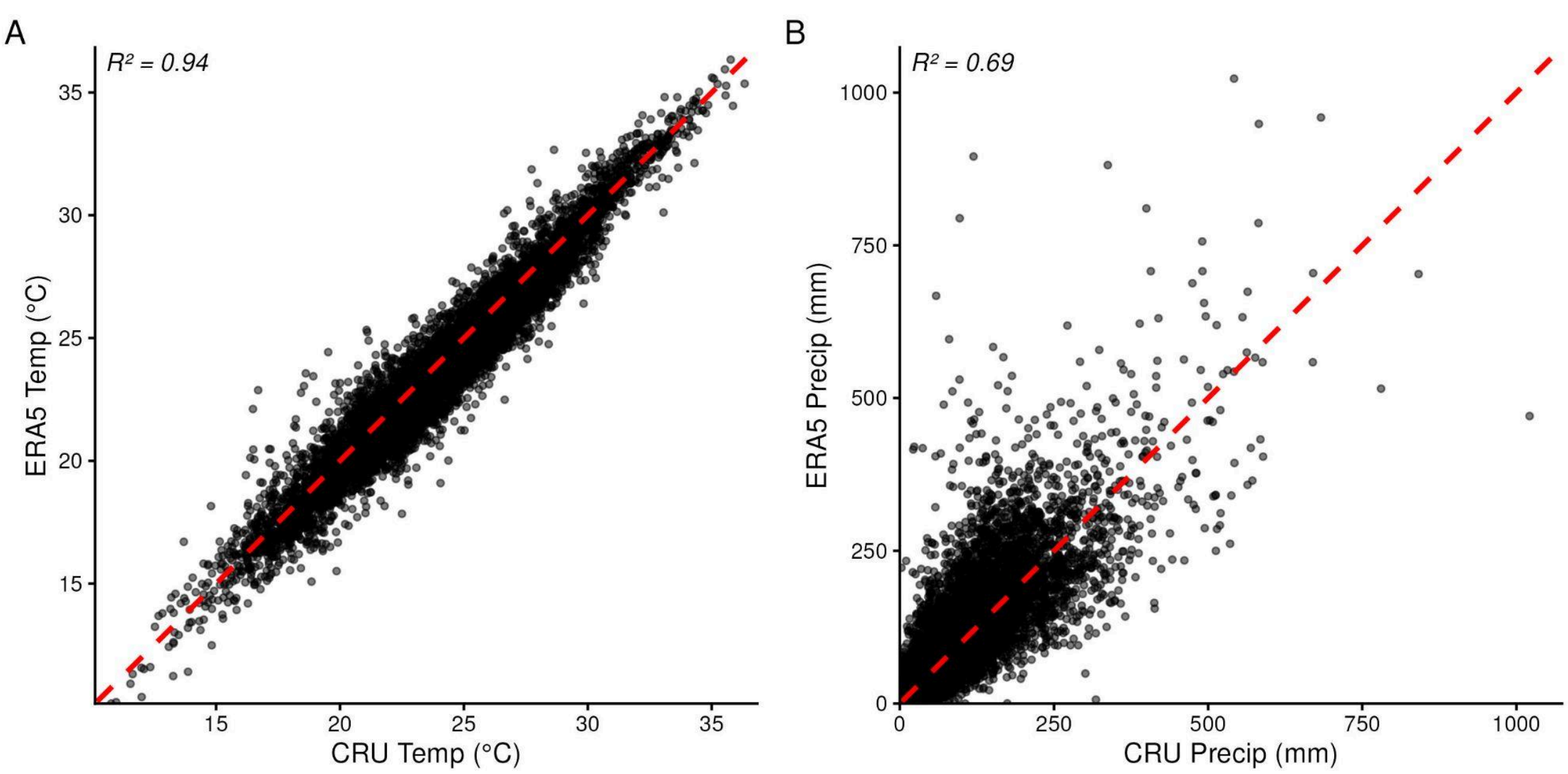

Figure R1. Comparison of Climate Weather Unit (CRU-TS) and European Centre for Medium-Range Weather Forecasts Reanalysis v5 (ERA5) weather data. Our main analysis relies on CRU weather data, but here we show ERA5 weather aggregates (vertical axis) against CRU weather aggregates

(horizontal axis); each point is an area-weighted average of (A) monthly average temperature or (B) monthly total precipitation for all grid cells overlapping with each first order administrative unit in our estimating sample in a given year and month. All data is limited to the years 1940-2015, the time overlap of the two datasets and the estimation sample. The coefficient of determination (R^2) is displayed to show similarity. Red dashed lines are at 45°.

	Dependent variable:		
	$Pf\mathrm{PR}_{2-10}$		
	CRU (full sample)	CRU (post 1940)	ERA5 (post 1940)
	(1)	(2)	(3)
Avg temp. (°C)	4.36**	4.35**	2.83*
	(1.52)	(1.56)	(1.25)
Avg temp.^{2} (°C)	−0.09**	−0.09**	−0.06*
	(0.03)	(0.03)	(0.02)
Flood	−0.15	−0.17	−0.22
	(0.91)	(0.92)	(1.01)
Flood (lag 1)	−0.48	−0.65	0.53
	(0.78)	(0.80)	(0.82)
Flood (lag 2)	2.03*	2.13**	1.48
	(0.79)	(0.81)	(0.80)
Flood (lag 3)	0.72	1.07	2.03*
	(0.85)	(0.85)	(0.82)
Drought	−0.26	−0.45	0.84
	(0.76)	(0.78)	(0.88)
Drought (lag 1)	−1.07	−1.10	−0.85
	(0.88)	(0.88)	(0.83)
Drought (lag 2)	−2.14**	−2.46***	−0.16
	(0.69)	(0.69)	(0.84)
Drought (lag 3)	0.59	0.86	0.89
	(0.92)	(0.96)	(0.85)
Intvn. 1955-69	−4.84**	−3.75*	−3.67*
	(1.68)	(1.65)	(1.65)
Intvn. 2000-15	−3.36	−0.82	−0.72
	(2.91)	(2.94)	(2.95)
Observations	9,875	9,293	9,293
R^2	0.53	0.54	0.54
Adjusted R^2	0.49	0.50	0.50

Note: $^*p < 0.05$; $^{**}p < 0.01$; $^{***}p < 0.001$

Table R2 (Table S1 in revised manuscript): Sensitivity of estimated effects of temperature and rainfall on *Pf*$\mathrm{PR}_{2–10}$ to climate input data. All columns show regression results for a dependent variable of malaria prevalence among children aged 2–10 (*Pf*$\mathrm{PR}_{2–10}$). Column (1) reproduces the preferred specification from Table 2, column (5). Column (2) subsets the Climate Weather Unit (CRU) data to the

same time span as the European Centre for Medium-Range Weather Forecasts Reanalysis v5 (ERA5) data to make a more direct comparison. Column (3) replaces CRU temperature and precipitation aggregates with those derived from ERA5 monthly data from 1940 to present. Standard errors are clustered at the country-by-5-year group level.

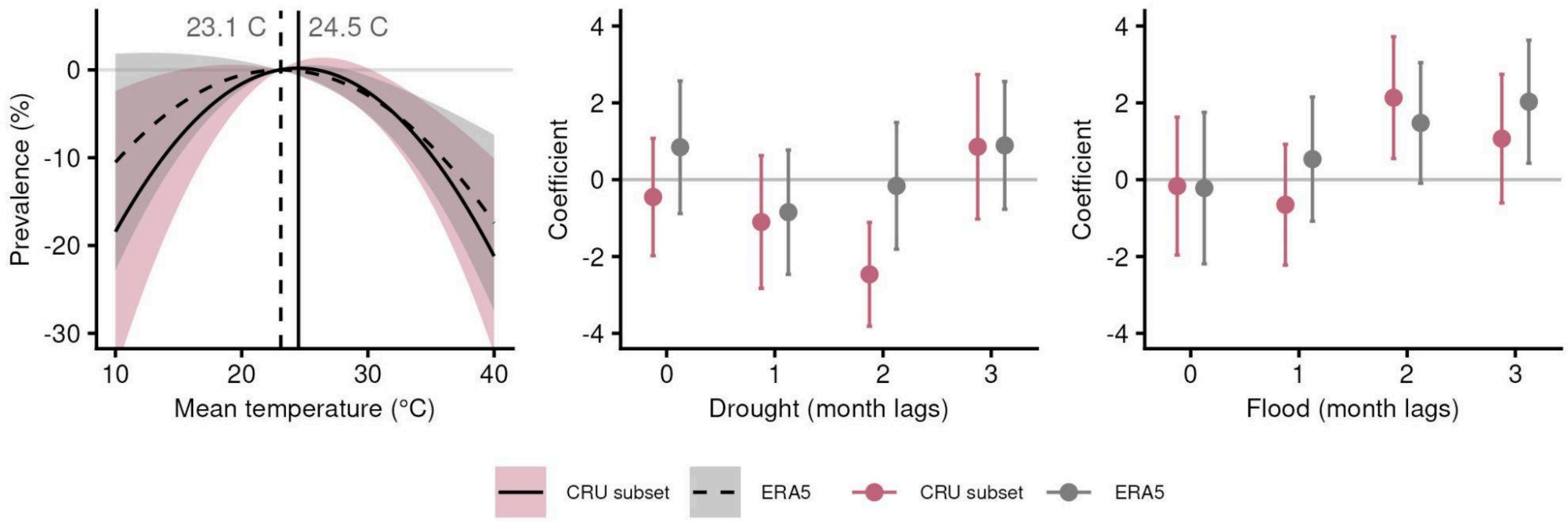

Figure R2 (Figure S9 in revised manuscript). Empirical estimates of prevalence-climate relationships using alternative climate input datasets. Panels show the estimated relationship between malaria prevalence for children aged 2-10 and monthly average temperature (left), drought (middle), and flood (right). Regressions are estimated separately for our main model specification using Climate Weather Unit (CRU) climate data or an alternative climate dataset, the European Centre for Medium-Range Weather Forecasts Reanalysis v5 (ERA5). Both regressions are limited to the years 1940-2015, the time overlap of the two datasets and available malaria prevalence data. (Left) The relationship between prevalence and mean monthly temperature for a CRU-based model (black solid line and pink shading) and for an ERA5-based model (dashed line and grey shading), with shading indicating 95% confidence intervals. (Middle, right) Estimated coefficients and 95% confidence intervals for the effects of drought (middle) and flood (right) events across contemporaneous and lagged months for both model specifications. Positive coefficients indicate higher malaria prevalence associated with extreme climate events. All standard errors are clustered at the country-by-5-year group level.

Reviewer 2

Summary

The authors have responded constructively to all of my previous comments.

I maintain that the uncertainty reported in the main specification is likely underestimated due to the choice of standard error clustering. However, the authors have presented additional (and, in my view, more appropriate) standard error calculations. Although these yield larger uncertainties, they confirm that the regression results remain statistically significant. Furthermore, independent robustness checks I performed using the provided data also confirm the statistical significance of the coefficients for the main variables (temperature and temperature squared).

Overall, while the magnitude of the effect appears small, the results robustly demonstrate statistically significant effects of temperature on malaria prevalence for children aged 2–10.

Aside from statistical uncertainty calculations, I identify two further (minor) issues: The authors should provide additional intermediate data files to ensure full reproducibility of their analysis. Furthermore, statistical information is not always given in a clear way or does not follow *nature* guidelines.

Below, I detail my remaining concerns, which the authors should be able to address in a revision of their manuscript.

We thank the reviewer for their incredibly careful feedback and for the thorough additional analyses they conducted to further strengthen our paper. As we detail below, we have updated the entire paper's analysis to rely on the reviewer's recommended method of characterizing uncertainty. We have also added all recommended robustness checks, provided intermediate data files and a much cleaner and more replicable computational pipeline, and updated presentation of statistical information.

1 Reproducibility

The authors provided additional processed data to facilitate the reproduction of their regression results. While this is helpful, the provided code references further processed data files that are currently unavailable (e.g., ADM1-centroids.csv, PrecipKey.csv, CRU-Reextraction-Aug2022.csv). These files are required to fully reproduce the analysis. In their data availability statement, the authors note that raw climate model simulation data are too large for standard public data repositories, but are available upon request. While this is reasonable, intermediate data files (which typically fit easily within repository limits, such as Zenodo's 50GB cap) should be made available directly.

Thank you for this feedback. All intermediate data files, as well as the previously provided preprocessed analysis-ready dataset, will be available directly in Zenodo or Figshare upon publication. We have included a .zip file here of this updated replication dataset. Raw climate data are large and available publicly. We therefore include guidance for how to download the publicly available raw climate datasets, alongside all processing scripts to convert these raw files into the intermediate outputs we publicly share. These intermediate outputs include CMIP6 data that has been bias corrected against CRU 4.03, the bias corrected ADM1 aggregate data, ERA5 ADM1 aggregate data, CRU ADM1 aggregate data, prediction summary files of varying spatial degrees, and other supporting files. We anticipate these files will require ~35 GB of storage space and will be made available on either Zenodo or Figshare.

We would also like to note that in this revision, we have substantially improved the structure and clarity of our replication codebase, such that replicating the entire paper's analysis is easier for future users. These changes provide a simplified and streamlined codebase that will work with the data release, requiring only minimal modifications in the configuration file found in pipeline section A of the GitHub repository. Users seeking to replicate our results will find instructions in the README file at the root of the repository. These changes will be merged to the main branch of the GitHub repository prior to publication.

2 Star-Marks in Tables

Tables S1, S2, and S6 are missing notes defining the standard errors (in parentheses) and the significance stars. The stars appear to indicate statistical significance based on the thresholds *** < 0.01 < ** < 0.05 < * < 0.1. This deviates from the standard convention of *** < 0.001 < ** < 0.01 < * < 0.05. Conventionally, values where 0.05 < p < 0.1 are not considered statistically significant. This is particularly problematic given that the meaning of the symbols is not explicitly defined in the table notes.

According to https://www.nature.com/nature/for-authors/initial-submission section Statistical information, exact p-values instead of star marks should be provided.

Thank you for catching this - this was simply a byproduct of using the `stargazer` package in R to programmatically generate our LaTeX tables, which uses these cutoffs by default. We have adjusted these cutoff values to follow the reviewer's guidance and added corresponding notes to the bottom of all regression tables. For all in-text statistical estimates, we report confidence intervals, p-values, and/or the proportion of simulation runs (which represents both statistical and climate uncertainty) that are positive, depending on what is most relevant to the statistic presented.

3 Correlation, Uncertainties, and Robustness

3.1 Correlation Analysis

The correlation analysis conducted by the authors reveals both serial and spatial correlation. While the spatial correlation values (e.g., same country or <500km in Table R3) are slightly lower than the serial correlation values (1 month lag), they are of a similar magnitude and significantly larger than correlations one would obtain from randomly permuted observations (which serve as a baseline for the null hypothesis of no spatial correlation). Therefore, I do not see why the authors draw such different conclusions for the two types of correlation, accepting the serial but dismissing the spatial.

It appears that there is both spatial and temporal autocorrelation. While it is difficult to account for both perfectly without inflating the volatility of the uncertainty estimate itself, I believe that clustering by ADM1 underestimates uncertainty. Better options are available, such as the Conley standard errors shown by the authors. However, since correlations with one- or two-year lags also shows some persistence (this calculation was done by me and so far not shown in the authors' Table R3; but the authors' own analysis in Table R4 also suggests at least one lag year of dependence), a pragmatic compromise could be clustering by country–decade (or country–5-year-period). While imperfect, as it may miss spatial correlations across borders or temporal correlations across decade cutoffs, this approach would capture the bulk of the correlation structure. As detailed below, the temperature terms remain significant under this specification.

I recommend country–decade or country–5-year-period clustering. Choosing a different clustering scheme also warrants a respective change in the bootstrap block scheme. In this

regard, country–time-period blocks are easy to implement, in contrast to Conley standard errors which, to my knowledge, do not have a direct bootstrap counterpart.

As we noted in our original reviewer response, we interpret the results of these correlational analyses with caution, given the unbalanced panel nature of our data and therefore limited data with which to estimate any given set of spatial or temporal correlations in model residuals. That said, we agree with the reviewer that the tests indicate some spatial correlation, and defer to their suggestion. In response, we have updated our main model specification to use country-by-5-year clusters, and we show country-by-decade, alongside other alternative clustering approaches, in an expanded sensitivity analysis in the Supplement (see below for details in response to comment #3.4.). While we also explored using Conley standard errors, which can be modified to allow for temporal correlations with specified temporal lag lengths in addition to spatial correlation (e.g., using the `time` and `lag_cutoff` arguments in `conleyreg` in `R`), we added new rows to Table S7 (formerly, Table S5) that show spatial correlation is dominated by country boundaries. For example, mean correlation across residuals from ADM1 units within 500km of one another is 0.30 within country borders, but just 0.08 across country borders. While we don't show all cutoffs so as not to overcomplicate the table, the same finding holds with other spatial cutoffs: mean correlation for ADM1s within 1000km of one another is 0.28 within countries, but 0.06 across countries. Given this result, and that we find little evidence of serial correlation in model residuals beyond five years (see new rows added to Table S7, as suggested by the reviewer, as well as results from the prior revision in Table R4), we feel that the reviewer's recommendation of country-by-5-year clustering is the most appropriate choice.

This adjustment to statistical uncertainty requires corresponding changes to the climate attribution and projection simulations, which account for both statistical and climatological uncertainties. While we previously implemented this propagation of uncertainty using a block bootstrap approach, in the revision we have replaced the bootstrap with a standard resampling method, which simply draws 1,000 instances from the multivariate normal distribution described by the clustered variance-covariance matrix recovered using the sandwich estimator. Both bootstraps and resampling are common practice in the statistical literature on climate impacts (e.g., Rode et al. (*Nature* 2022) and Hultgren et al. (*Nature*, 2025) use resampling, while Diffenbaugh and Burke (*PNAS,* 2019) and Burke et al. (*Nature* 2026) use bootstrapping). We made this change for two reasons. First, the resampling method allows us to easily accommodate any of the reviewer's suggested methods for estimating uncertainty, including Conley standard errors, for which there is no bootstrap counterpart (as the reviewer states). Should the reviewer decide they prefer Conley, we would be happy to implement it not only in the statistical modeling, but also in the climate simulations.

Second, we found that the requested country-by-5-year clustering produces very similar but, importantly, *asymmetric* confidence intervals when estimated via the block bootstrap (see Figure R4 below). These necessarily differ (although only slightly) from the clustered standard errors recovered via the sandwich estimator. In contrast, the bootstrap and the resampling method are identical, and both symmetric, under the prior ADM1 level clustering. We confirmed the

asymmetry persists with additional bootstrap samples and stems from the large heterogeneity in block sample sizes across country-by-5-year groupings: the coefficient of variation of block sample size exceeds 1.6, implying that individual blocks can exert high leverage. Although methods such as the wild cluster bootstrap (Cameron, Gelbach & Miller, 2008) can theoretically address such heterogeneity, to our knowledge no implementation exists in R, Python, Stata, or Julia that accommodates high-dimensional fixed effects and the need for repeated simulation runs to propagate uncertainty through climate simulations. We therefore elect to use resampling for our climate simulations. Of course, we continue to show results for alternative clustering approaches in both figures and in tabular format.

Because of the change in clustering, we have updated every associated figure and table in the paper: as expected, point estimates do not change but uncertainty in both regression results and climate change simulations increases. All in-text statistics have been updated to reflect this increased uncertainty. We show our new Figure 2 below as it is the most directly relevant to this update in methodology, but Figures 3 and 4, as well as all associated figures and tables in the Supplement, have also been updated.

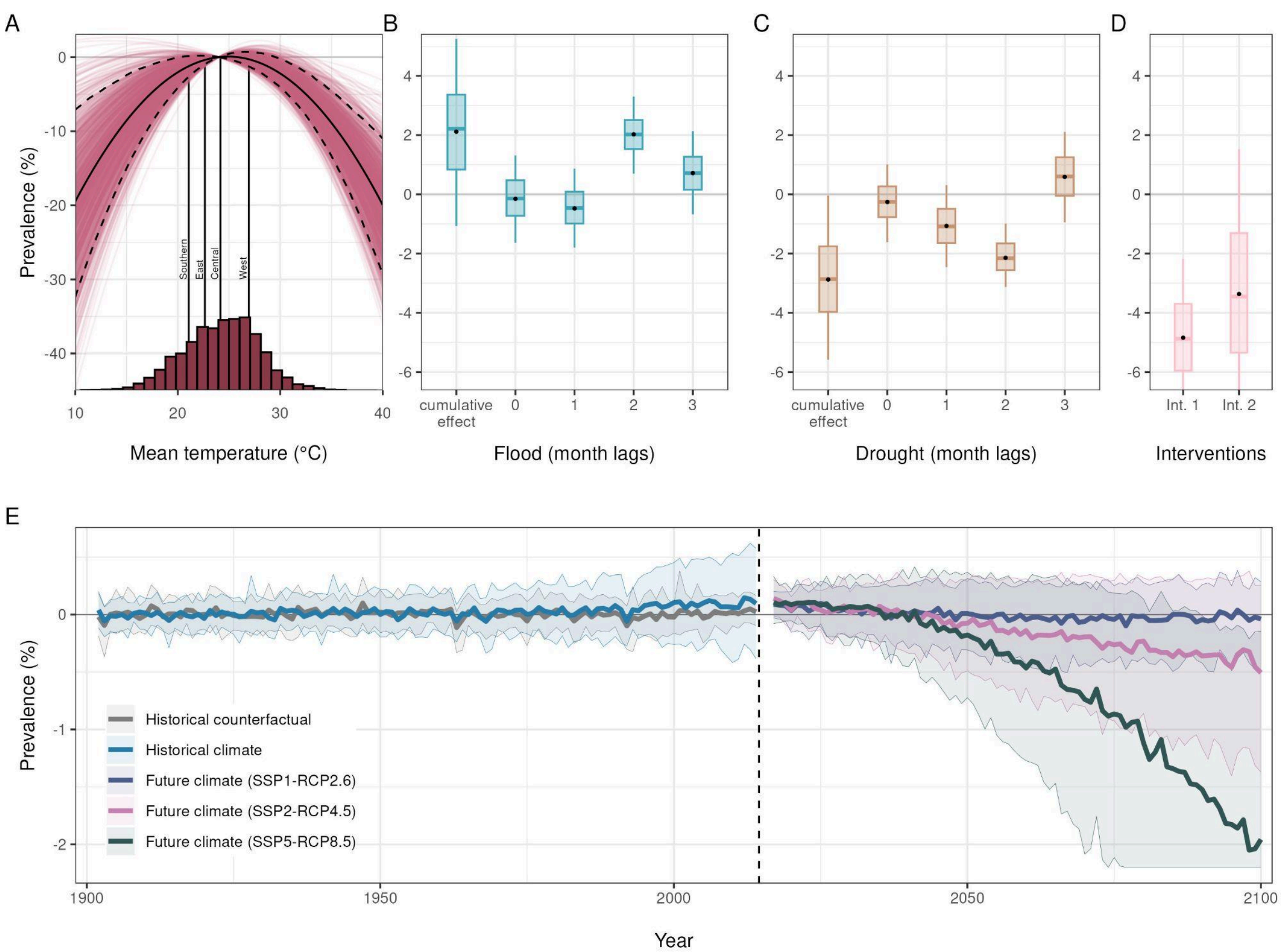

Figure R3 (Figure 2 in revised manuscript). Empirical estimates of prevalence-climate relationships and predictions of climate change impacts from 1901 to 2100. (A) The estimated relationship between temperature and prevalence (point estimate in solid black; 90% CI in dashed black; 1,000 random draws from the variance-covariance estimate in red). Histogram shows the distribution of

monthly temperatures across the estimation sample, with regional median temperatures shown as vertical lines. (B,C) The effects of extreme precipitation events (flood and drought) in the month they occur (0 lag) and after time has passed (1, 2, 3 month lags), as well as the cumulative impact across the first four months. (D) The effects of two key intervention periods (Int. 1 = 1955-1969; Int. 2 = 2000-2015) during which large-scale malaria prevention programs were implemented across the sub-continent.(B-D) Point estimates (black) are accompanied by resampled draws shown as boxplots, where the box spans the 25th to 75th percentiles, the center line indicates the median, and whiskers extend to the 5th and 95th percentiles (blue, brown, pink). (E) Predicted change in prevalence attributable to anthropogenic climate change in the recent past (real historical climate given in blue; counterfactual without anthropogenic warming in grey) and in the future for low (blue: SSP1-RCP2.6), intermediate (pink: SSP2-RCP4.5), and high (green: SSP5-RCP8.5) emissions scenarios. Thick lines are the mean estimates accounting for both statistical and climate uncertainty; shading indicates the 5th and 95th percentiles and is truncated at the lower axis limits for visualization purposes only (the full interval is shown in Figure S18A). Historical estimates are shown relative to an average baseline across 1901 to 1930. Future estimates are shown relative to a baseline across 2015 to 2019, added to the end-of-historical baseline (2010 to 2014). Years with incomplete predictions due to lag effects (1901 and 2015) are not displayed.

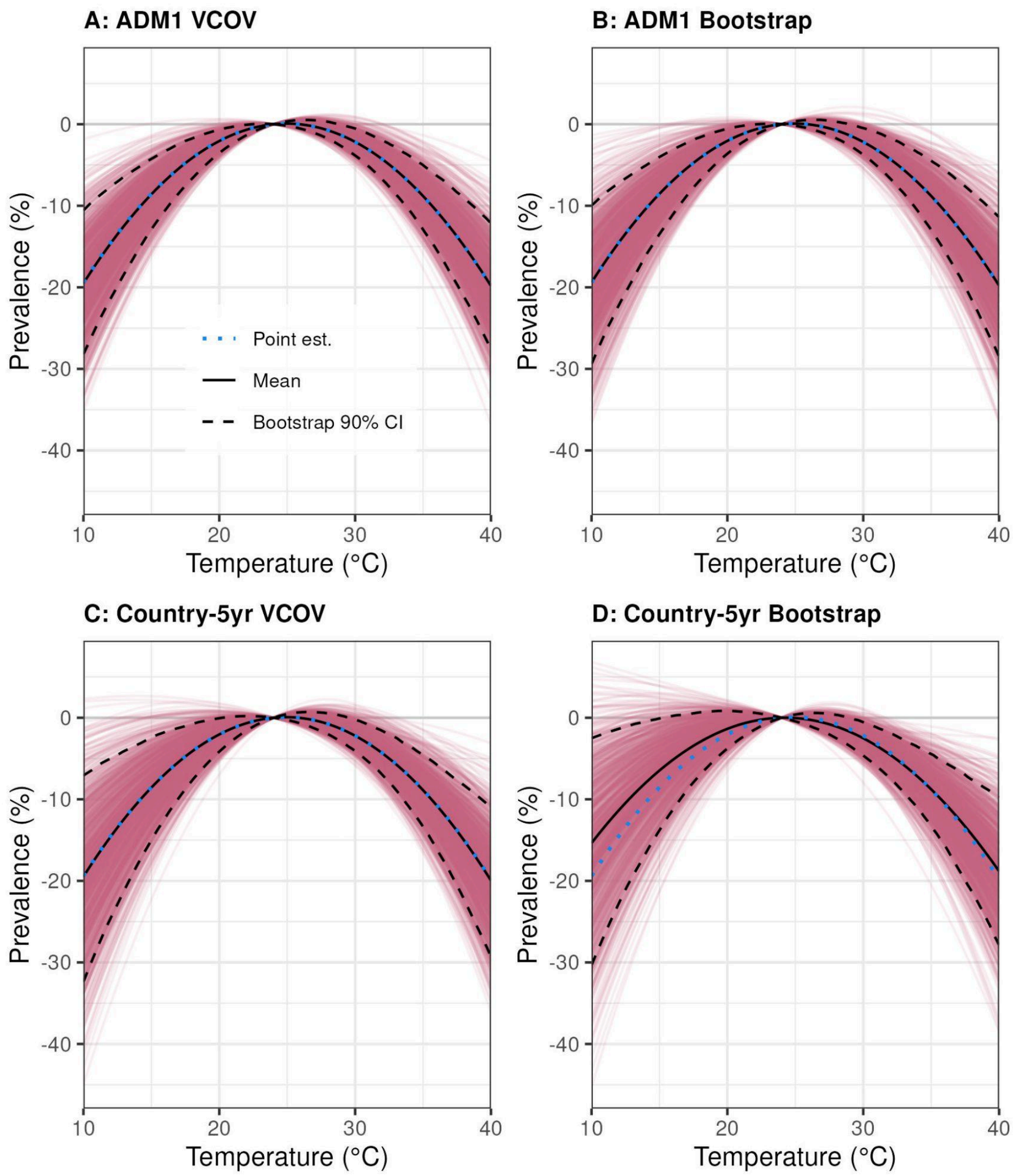

Figure R4. Clustered standard errors estimated via resampling and block bootstrapping. All panels show the estimated relationship between temperature and prevalence with the point estimate in the dotted blue line and a 90% confidence interval in dashed black. In panels A and C, 1,000 random draws from the variance-covariance estimate are shown in red, while in panels B and D, 1,000 block bootstrap estimates are shown in red; in both panels, means of these distributions are shown in solid black. Panels A and B use ADM1-level clustering of standard errors (panel A) and a bootstrap that is blocked by ADM1 (panel B). Panels C and D use country-by-5-year clustering of standard errors (panel C) and a bootstrap that is blocked by country-by-5-year groups (panel D). Means over the 1,000 draws diverge from point estimates only in panel D, due to large heterogeneity in group size across the country-5-year groups.

3.2 Variogram

The authors present a variogram to assess spatial autocorrelation. However, the results in Figure R7 (showing a flat variogram for the residuals) appear to contradict the authors' own correlation analysis in Table R3, which indicated positive spatial correlations (e.g., .ρ = 0.30 for units <500km). I suspect this discrepancy stems from how the variogram was constructed.

By collapsing the temporal dimension, pooling all time periods for a given region into the same spatial bin, the authors effectively treat temporal variation as instantaneous noise (or ”nugget” variance). Since the variance at distance zero now includes the entire temporal variance of the series, the initial semivariance is artificially inflated. This high baseline likely drowns out the signal of spatial dependence, making the curve appear flat even if spatial correlation exists.

As an additional note, the authors describe the variogram as a ”higher-powered test.” A variogram is an exploratory diagnostic tool, not a formal statistical hypothesis test; therefore, it does not have ”statistical power” in the standard sense. Using such terminology in this context may be misleading. Even if the authors merely want to claim that the variogram approach is more appropriate and informative, I did not find specific reasons in their text to support this claim clearly (e.g, sparsity of the panel may also affect the variogram not only the correlation analysis).

The reviewer is correct that the difference between the variogram and the spatial correlational analyses in former Table S5/R3 (now Table S7) is that the temporal dimension is collapsed. Given that we now include detailed analyses that separately explore spatial and temporal correlations in model residuals, and that we estimate model uncertainty accounting for spatial correlation in residuals, we have removed the variogram from the text to avoid any confusion about the difference between two different spatial correlation calculations.

3.3 Abadie Study

The results of Abadie et al. (2023) mentioned by the authors to reason against spatial correlation robust clustering are not applicable in this context. As the authors themselves acknowledge, the setting in that study differs fundamentally from the panel data regression employed in the present manuscript. Consequently, the findings of Abadie et al. cannot be applied here. Instead, established literature demonstrates that clustered standard errors are consistent asymptotically and even tend to underestimate uncertainty when the number of clusters is small [BDM04; CM15].

We agree with the reviewer that the Abadie et al. (2023) study is relevant, but not directly applicable, to our setting. Thus, while we mentioned it in our previous reply to the reviewer, we do not cite it or leverage its insights in the manuscript.

3.4 Independent Robustness Checks

I conducted several independent robustness checks regarding the statistical significance of the estimated coefficients in the authors’ regression model. These checks support the authors’ findings of a statistically significant relationship between temperature and malaria prevalence. I recommend that the authors replicate at least some of these checks in the supplementary material to further substantiate the robustness of the article’s main results.

- Alternative Clustering: Clustering standard errors by ADM1, country, year, country–year, country–5-year-period, country–decade, or month–year consistently results in significant coefficients for temperature and its squared term, as well as for the first intervention

variable. The flood.lag2 variable remains significant in some, though not all, specifications.

- Sensitivity to Controls (expanding on Figure S7):
  - Adding month–year fixed effects retains the statistical significance of the squared temperature term and flood.lag2 for all clustering schemes mentioned above.
  - Using ADM1 and country–year fixed effects removes the statistical significance of the temperature variables. This is not concerning, as data within the same country and year are highly correlated, meaning the country–year fixed effect absorbs most of the identifying variation. However, this provides further evidence of non-negligible correlation in the temporal and spatial dimensions.
  - Using ADM1–decade and region–month–decade fixed effects retains the statistical significance of the squared temperature term and flood.lag2 for all clustering schemes mentioned above, except when clustering by country (which is generally not recommended here due to the relatively low number of countries).
- Temporal Restrictions (expanding on Figure S8):
  - Restricting the sample to data after 1980 retains the statistical significance of the temperature terms for all clustering schemes mentioned above, with the exception of clustering by country.
  - Restricting the sample to data after 1950 retains the statistical significance of the temperature terms for all clustering schemes mentioned above.
- Influence Analysis (Leave-one-out):
  - Removing any single country from the dataset retains the statistical significance of the temperature terms for all clustering schemes mentioned above.
  - Removing any single year from the dataset retains statistical significance for all clustering schemes.
  - Removing any single calendar month from the dataset retains statistical significance for all clustering schemes (except for the linear term when clustering by country).
- Outliers: The data does not appear to contain strong outliers. A normal Q-Q plot of the residuals from the main model indicates a distribution that is close to normal, albeit with slightly heavier tails (excess kurtosis ≈ 0.8). Given the large sample size, these slightly heavy tails are not a major concern, and the application of ordinary least squares (OLS) regression appears appropriate.

We thank the reviewer for their careful replication and extensions of our results. We have included all of these robustness and sensitivity analyses in our updated manuscript. We summarize each change here and replicate key updated or new figures in the revised manuscript.

- **Alternative clustering** (expanding former Figure S17 and Table S6). We have added country, year, country–year, country–5-year-period, and country–decade clustering to what is now Figure S10, with corresponding tabular results shown in Table S3. Note that we did not add month-year clustering as we already include year clustering (although, as

the reviewer notes, our variables of interest remain statistically significant under this approach). Figure R5 replicates this new figure below and Table S6 can be found in the revised Supplement. These results are mentioned on line 150 of the main text.

- **Sensitivity to controls** (expanding former Figure S7 and Table S2). We have added all three additional fixed effects specifications requested by the reviewer. Figure R6 below replicates what is now Figure S11 in the revised Supplement, with tabular results in Table S4 is in the revised Supplement. These results are discussed on lines 151-153 in the main text, and the fact that the shape of the prevalence-temperature response changes with country-year fixed effects is mentioned on lines 660-662 of the Methods section.
- **Temporal restrictions** (expanding former Figure S8). We now show in Figure S9 (replicated above as Figure R2) sensitivity to input climate dataset, as requested by the Editor. As part of this analysis, we restrict the sample to post-1940, as our alternative climate data source from ERA5 is only available after this date, and show that results are very similar to the full sample. We view this as sufficient for demonstrating robustness to temporal subsampling, as we don't see a clear econometric or institutional reason for choosing other cutoffs and given that the reviewer has already replicated our results at other temporal cutoffs. We now show the full sample, post-1940 due to ERA5 restrictions, and pre- and post-1994, given half the data lie before this date.
- **Influence analysis.** We have implemented all the recommended influence analysis tests and show the results in Figure S12, replicated below as Figure R7. These show that the point estimates and p-values on temperature coefficients are highly insensitive to dropping individual countries, years, or calendar months. Coefficients across the samples lie within a small range of one another and all p-values are below 0.05, with the vast majority falling below 0.01. These results are now mentioned on lines 154-155 of the main text.
- **Outliers.** We have added a distribution of model residuals and a QQ-plot, now composing Figure S20, replicated below as Figure R8. We have added a note on lines 633-635 in the Methods section regarding the lack of outliers.

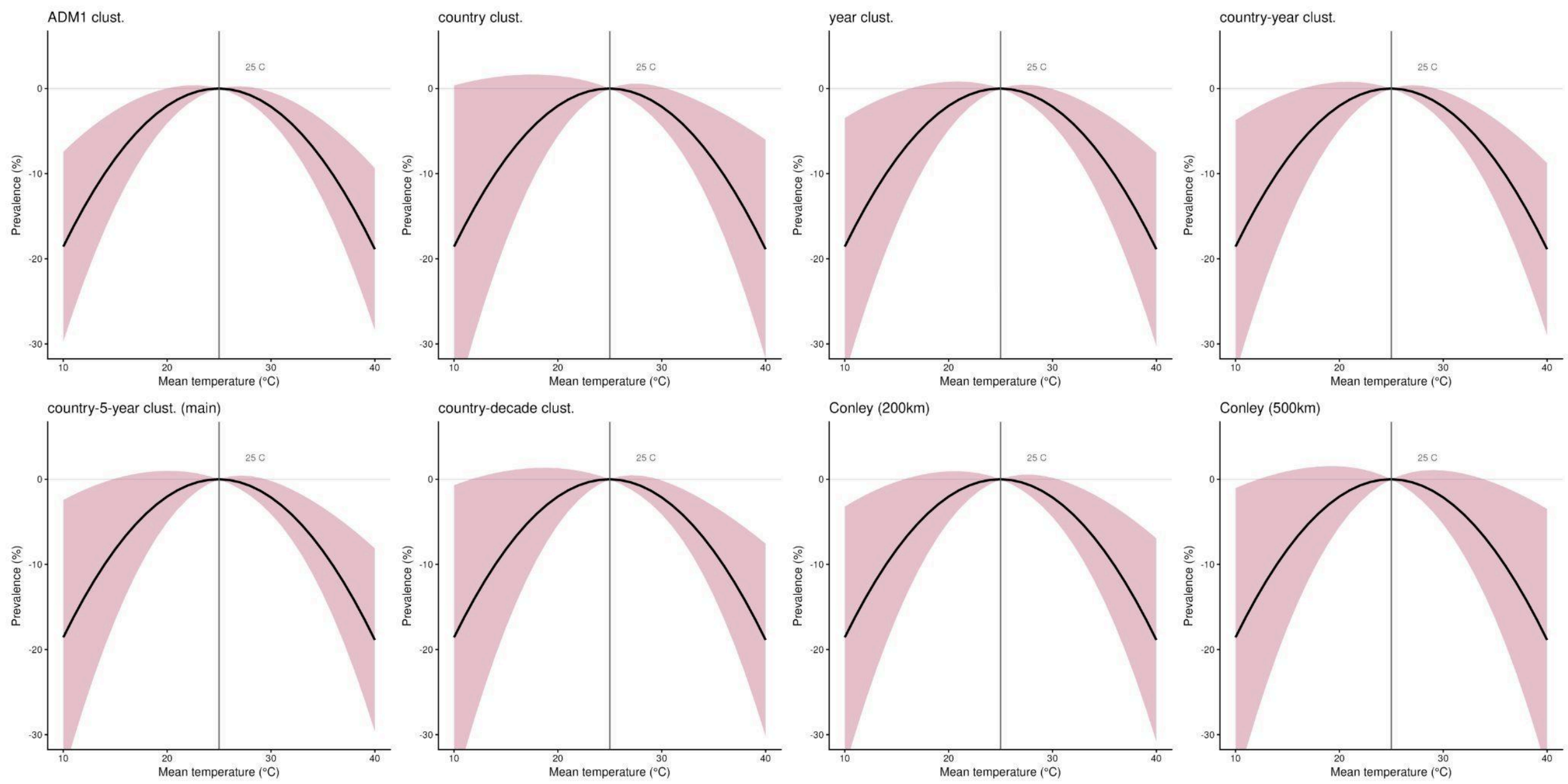

Figure R5 (Figure S10 in revised manuscript): Alternative estimates of model uncertainty in the *Pf*$PR_{2\text{-}10}$-temperature relationship. All panels show the estimated relationship between malaria prevalence for children aged 2-10 and monthly average temperature as estimated following Equation 4. All temperature responses are plotted relative to 25°C, the temperature at which prevalence is maximized. From top-left to bottom-right, standard errors are estimated using: ADM1-level clustering; country clustering; year clustering; country-by-year level clustering; country-by-5-year level clustering; country-by-10-year level clustering; Conley standard errors with a cutoff of 200 km; and Conley standard errors with a cutoff of 500 km. Tabular results, including associated estimates of precipitation extremes, are shown in Table S3.

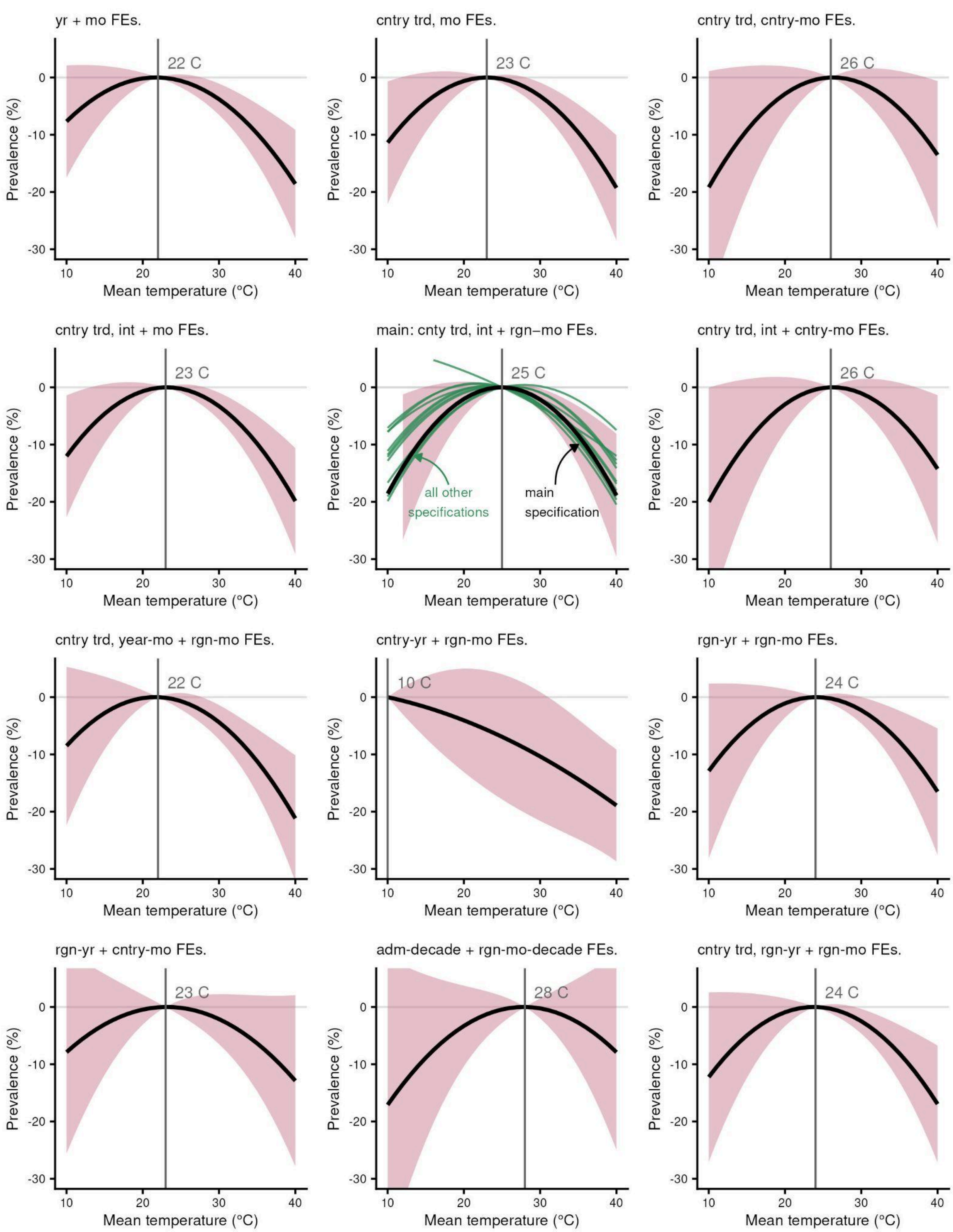

Figure R6 (Figure S11 in revised manuscript) Sensitivity of the *Pf*PR$_{2\text{-}10}$-temperature relationship to alternative spatiotemporal controls. All panels show the estimated relationship between malaria prevalence for children aged 2-10 and monthly average temperature and all include fixed effects (i.e.,

dummy variables) at the scale of the first administrative unit (i.e., ADM1). All temperature responses are plotted relative to the model-specific temperature at which prevalence is maximized; this peak temperature is indicated in grey text and with a vertical grey line in each panel. From top-left to bottom-right, model controls are: year and month fixed effects ("FEs"); country-specific quadratic time trends ("country trends") and month FEs; country trends and country-by-month FEs; country trends and intervention period and month FEs; country trends and intervention period and region-by-month FEs; country trends and intervention period and country-by-month FEs; country trends and year-by-month and region-by-month FEs; country-by-year and region-by-month FEs; region-by-year and region-by-month FEs; region-by-year and country-by-month FEs; ADM1-by-decade and region-by-month-by-decade FEs; and region-by-year and region-by-month FEs with country-specific linear time trends. The preferred specification used throughout the main text is the fifth panel, where the main specification is shown in black (with pink shaded 95% confidence intervals) and all alternative specifications from other panels are overlaid in green. "Region" refers to the Global Burden of Disease definitions of western, southern, central, and eastern Africa. All standard errors are clustered at the country-by-5-year level.

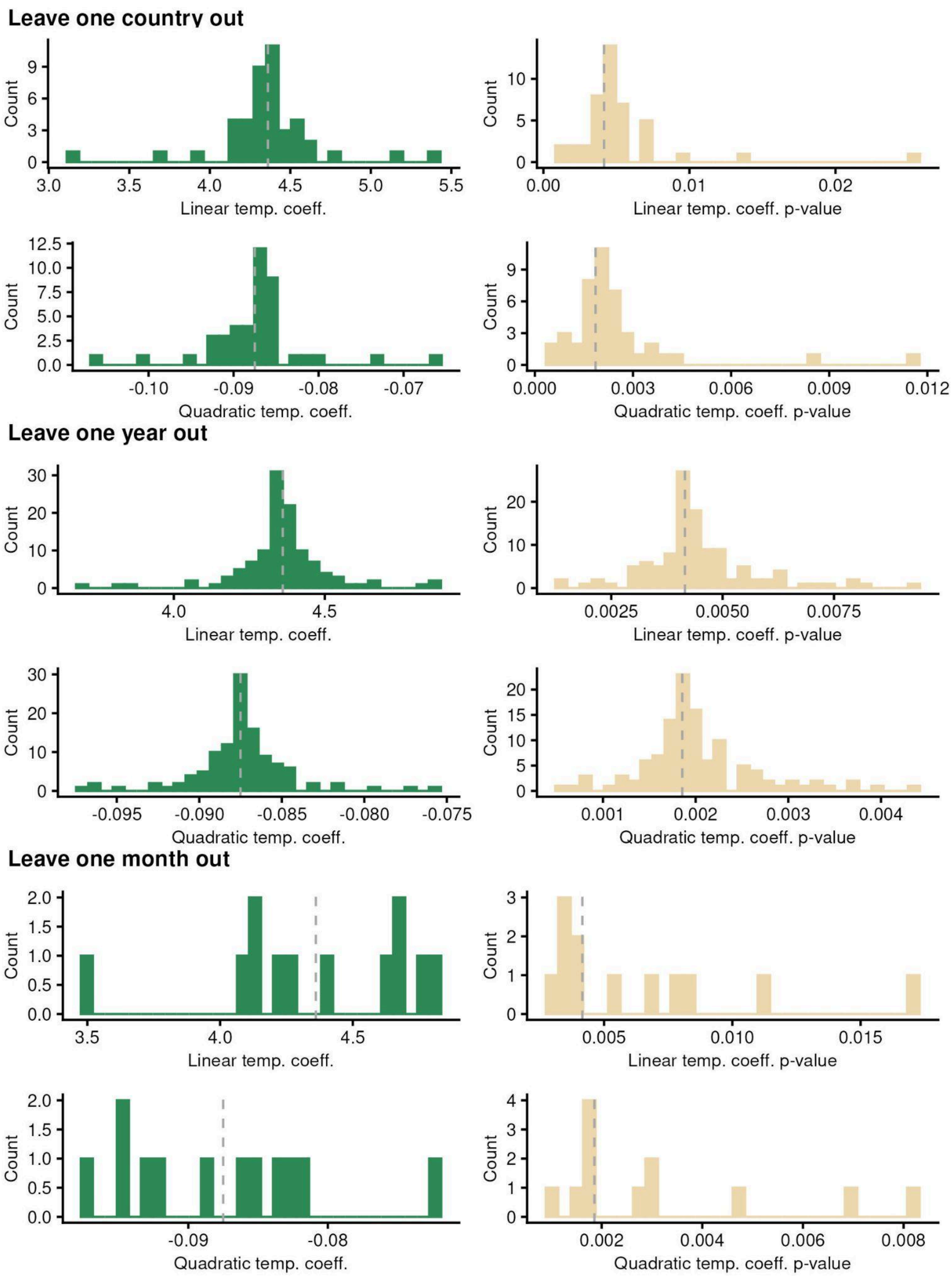
Leave one country out
Count
0
3
6
9
3.0
3.5
4.0
4.5
5.0
5.5
Linear temp. coeff.
Count
0
5
10
0.00
0.01
0.02
Linear temp. coeff. p-value
Count
0.0
2.5
5.0
7.5
10.0
12.5
-0.10
-0.09
-0.08
-0.07
Quadratic temp. coeff.
Count
0
3
6
9
0.000
0.003
0.006
0.009
0.012
Quadratic temp. coeff. p-value
Leave one year out
Count
0
10
20
30
4.0
4.5
Linear temp. coeff.
Count
0
10
20
0.0025
0.0050
0.0075
Linear temp. coeff. p-value
Count
0
10
20
30
-0.095
-0.090
-0.085
-0.080
-0.075
Quadratic temp. coeff.
Count
0
5
10
15
20
0.001
0.002
0.003
0.004
Quadratic temp. coeff. p-value
Leave one month out
Count
0.0
0.5
1.0
1.5
2.0
3.5
4.0
4.5
Linear temp. coeff.
Count
0
1
2
3
0.005
0.010
0.015
Linear temp. coeff. p-value
Count
0.0
0.5
1.0
1.5
2.0
-0.09
-0.08
Quadratic temp. coeff.
Count
0
1
2
3
4
0.002
0.004
0.006
0.008
Quadratic temp. coeff. p-value

Figure R7 (Figure S12 in revised manuscript) Influence of individual countries, years, and months on the *Pf*PR$_{2-10}$-temperature relationship. All plots show the results from influence analyses in which individual countries (top two rows), years (middle two rows), or calendar months (bottom two rows) are omitted from the sample and the statistical model in Equation 4 is re-estimated. Plots in green show the distribution of regression coefficients on the linear and quadratic temperature terms across all restricted samples, with the full-sample point estimate indicated by the grey dashed line. Plots in beige in the second column show the distribution of associated *p*-values on each temperature coefficient across all restricted samples, again with the full-sample *p*-value indicated by the grey dashed line. All regressions were run with standard errors clustered by country-by-5-year group.

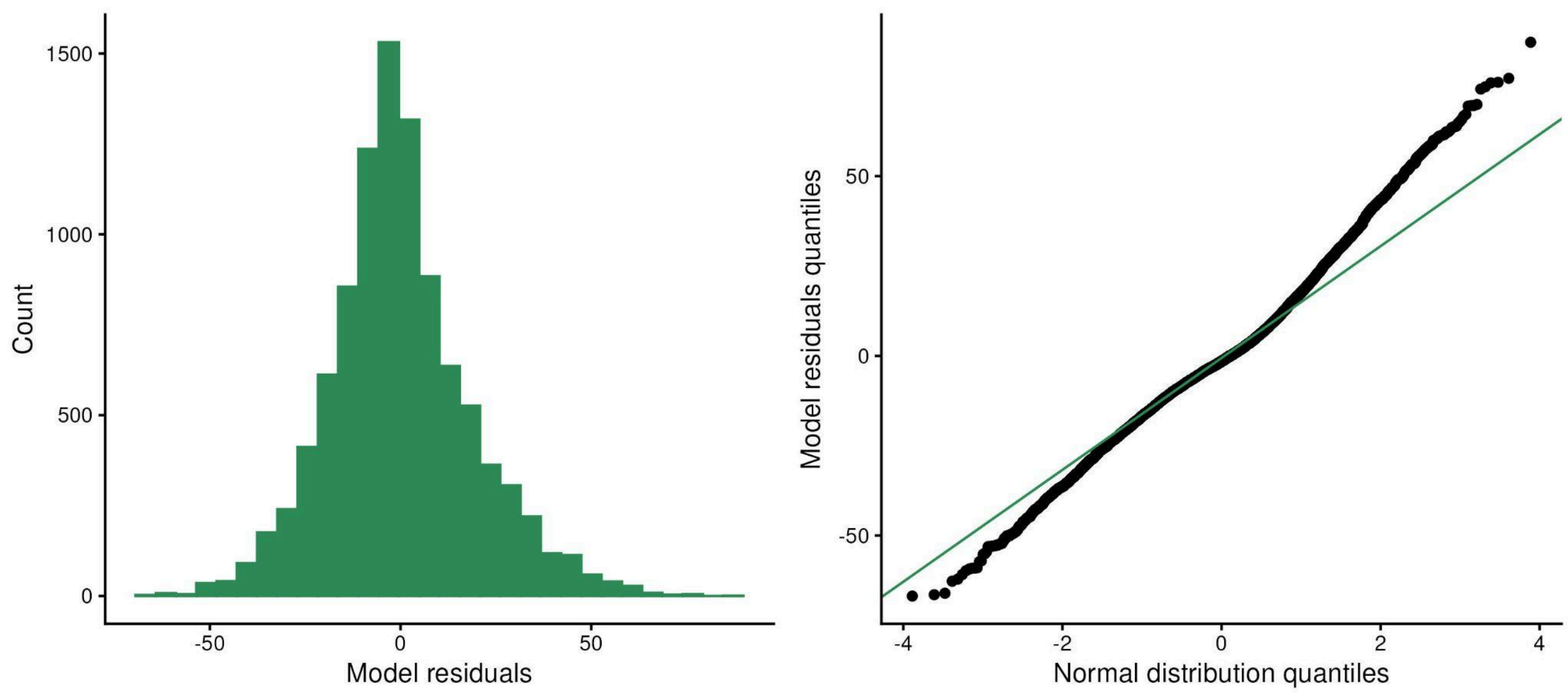

Figure R8 (Figure S20 in revised manuscript) Distribution of model residuals. (Left) The distribution of regression model residuals from estimating the statistical model in Equation 4. (Right) Q-Q plot showing how the quantiles of the model residuals distribution compare to those from a normal distribution. Residuals are close to normal, with moderately heavier tails indicated by residual quantiles falling below the normal distribution on the lower end, and vice versa on the higher end.

References

[BDM04] M. Bertrand, E. Duflo, and S. Mullainathan. "How Much Should We Trust Differences-In-Differences Estimates?*". In: The Quarterly Journal of Economics 119.1 (Feb. 2004), pp. 249–275. doi: 10.1162/003355304772839588.

[CM15] C. M. Cameron and D. L. Miller. "A Practitioner's Guide to Cluster-Robust Inference". In: The Journal of Human Resources 50 (2015), pp. 317–372.

Reviewer 3

1) I understand the relevance of pointing out the debate on the effect of climate change in East African highlands. It is not completely accurate to say that the highest profile studies focused on

a single data set. It was indeed the case that there was a focus on the Kericho data set because of the sparsity of long retrospective records in the form of time series at a given location. There were additional data for Ethiopia and those studies are cited but only in the Discussion. This is a bit of a mis-representation of what has been done and whether the debate was resolved. The study by Siraj et al. (Science 2014) did put an end to the debate of whether warmer temperatures explained trends in highland falciparum malaria, and to whether one could explain those trends with drug resistance (since a similar trend was observed in vivax malaria , in the absence of such resistance). The study by Rodo et al. (Nature communications 2021) for these same data further show the relevance of temperatures to the temporal patterns of the disease up to the time of the enhanced intervention efforts around 2005.

I would suggest that the following text be edited some:
"Malaria experts have often claimed that observed warming trends are incompatible with long-term reductions in malaria prevalence across Africa, and warned that other factors like drug resistance and funding instability pose a more serious threat to malaria eradication[16,18,19]. Empirical evidence to test these assumptions is sparse, with the highest profile studies focusing on a single dataset of malaria incidence over several decades at a tea plantation in Kericho, Kenya. Since 2000, over a dozen studies have argued that these data either support[20,21,22,23,24] or undermine[25,26,27,28,29,30,31] the broader hypothesis that climate change is responsible for a resurgence of malaria in the east African highlands[28,32,33,34,35]"

Thank you for raising this important point. Our focus on the Kericho debate did indeed cause us to neglect these important contributions based on data from Ethiopia. We have rewritten this section as follows (lines 53-62; new and edited text in red):

> At the turn of the century, many malaria experts argued that observed warming trends were incompatible with long-term reductions in malaria prevalence across Africa, and warned that other factors like drug resistance and funding instability posed a more serious threat to malaria eradication. Malaria resurgence in the east African highlands became a particular point of contention, with over a dozen studies arguing for or against climate change as a significant driver. Today, malaria experts generally agree that climate change has contributed to elevational shifts in malaria epidemics [Siraj 2014, Rodo 2021] and the geographic ranges of mosquito vectors [Carlson 2023 *Biology Letters*]. However, the cumulative impact of climate change on the total burden of malaria is still an open question: recently, Snow….

2) The authors have included text on the effect of climate variability as requested. It may be useful to point out in the Discussion that future climate scenarios do not allow the consideration of changes in climate variability at interannual (and decadal) time scales, and that those scales are the ones most relevant to public health interventions. Given the visibility of publications in this journal and the importance of the subject, it would be detrimental to public health efforts for the message to be mis-interpreted as saying that the effects of climate "changes" at the time scales most relevant to their interplay with control will not matter. It will also be useful to point

out for readers not familiar with climate science, that climate variability and extremes will also change with climate change, and that the effects of such changes are not examined here.

We think this may be a miscommunication. As we discussed in our first revision, the global climate models (GCMs) we use in this study do indeed capture internal variability due to natural processes (e.g., El Niño Southern Oscillation, Pacific Decadal Oscillation) and stochasticity in the Earth system. This is one of the key reasons we use multiple GCMs – to capture uncertainty about this variability, as each GCM represents such variations differentially. Similarly, the GCMs that we use do indeed capture the effects of climate change on extreme weather, such as floods and droughts, and we examine them here: this is what drives, for example, the long-term effects of changes in flood and drought frequency, and the downstream effects on malaria, in Figures S18 rows 3 and 4 (note the subtle positive trend in the effects of floods in RCP8.5, particularly in the late 21st century).

What these future climate simulations *cannot* capture is the kind of short-term future change that one would be able to predict based on an annual weather forecast (e.g., Lowe 2014 *Lancet Infectious Diseases*) or decadal weather prediction (e.g., O'Kane 2023 *Frontiers in Climate*) – which is, as the reviewer says, the kind of predictive timescale most relevant to public health interventions. We have added this point to the Discussion in a way that hopefully cuts across differences in terminology (lines 311-324; new text in red):

> Our study therefore reconciles three long-standing ideas that are sometimes treated as paradoxical: anthropogenic climate change is not the primary force shaping past, or probably future, trends in malaria prevalence. However, anthropogenic climate change has probably increased the burden of malaria in sub-Saharan Africa, and at high elevations and latitudes, will continue to for several more decades. Nevertheless, rising temperatures at lower latitudes and elevations in Africa will mostly align with future efforts to eradicate *Plasmodium falciparum* from sub-Saharan Africa. Future work will be needed to situate these global trends in local contexts, particularly through work that leverages longitudinal data from malaria-endemic communities. Similarly, our study provides a long view of future climate change impacts under different emissions scenarios, but cannot be used as a forecast of year-to-year variation in malaria transmission. Future work should explore emerging methods for near-term climate prediction, which could give public health decision-makers information about what to expect over the next year [Lowe 2014] to decade [O'Kane 2023] – the timescale most relevant to malaria control.

3) Finally, I understand that the spatial resolution of CRU is the best one can do for the time span of interest here, and I appreciate the addition of the analysis down to that resolution, without the spatial aggregation. I still believe that spatial resolution can be a limiting factor in this study, especially for highlands, but also understand the response of the authors underscoring that the goal is to capture the trends over broad spatial extents. It will be interesting to consider in future studies how these findings compare to conclusions from more detailed studies of

longitudinal records (rather than assembled surveys) for shorter periods of time but for higher spatial resolution.

We absolutely agree, and have work in preparation looking at the site-specific effects of climate change based on longitudinal time series! We have added a reference to the need for this work to the Discussion, as part of our response to the above point.

References

Burke, Marshall, Mustafa Zahid, Noah S. Diffenbaugh, and Solomon Hsiang. "Quantifying climate loss and damage consistent with a social cost of carbon." *Nature* 651, no. 8107 (2026): 959-966.

Diffenbaugh, Noah S., and Marshall Burke. "Global warming has increased global economic inequality." *Proceedings of the National Academy of Sciences* 116, no. 20 (2019): 9808-9813.

Cameron, A. Colin, Jonah B. Gelbach, and Douglas L. Miller. "Bootstrap-based improvements for inference with clustered errors." *The review of economics and statistics* 90, no. 3 (2008): 414-427.

Harris, I., Osborn, T. J., Jones, P., & Lister, D. (2020). Version 4 of the CRU TS monthly high-resolution gridded multivariate climate dataset. *Scientific Data*, *7*(1), 109.

Hersbach, H., B. Bell, P. Berrisford, G. Biavati, A. Horányi, J. Muñoz Sabater, J. Nicolas, C. Peubey, R. Radu, I. Rozum, D. Schepers, A. Simmons, C. Soci, D. Dee, and J-N. Thépaut, "ERA5 Monthly Averaged Data on Single Levels from 1940 to Present" (Copernicus Climate Change Service (C3S) Climate Data Store (CDS), 2023), accessed April 11, 2026, https://doi.org/10.24381/cds.f17050d7.

Hultgren, Andrew, Tamma Carleton, Michael Delgado, Diana R. Gergel, Michael Greenstone, Trevor Houser, Solomon Hsiang et al. "Impacts of climate change on global agriculture accounting for adaptation." *Nature* 642, no. 8068 (2025): 644-652.

Rode, Ashwin, Tamma Carleton, Michael Delgado, Michael Greenstone, Trevor Houser, Solomon Hsiang, Andrew Hultgren et al. "Estimating a social cost of carbon for global energy consumption." *Nature* 598, no. 7880 (2021): 308-314.

Referee #2 (Remarks to the Author):

The authors have addressed all of my previous points. The effort they have made to verify the robustness of the climate change-prevalence relationship is exemplary, and the temperature effect is a statistically significant and important finding.

I have one remaining concern. While the temperature response function is well-supported, several downstream attribution claims are not statistically significant, and the current reporting of these in the abstract and discussion risks being misunderstood. I want to flag three passages.

Passage 1, Abstract:
"Comparing historical climate reconstructions to counterfactual simulations without anthropogenic climate forcings, we find a ~60% likelihood that human-caused climate change has increased the overall prevalence of childhood malaria across sub-Saharan Africa since 1901, with >90% likelihood in southern Africa and high-elevation east Africa."

The word "likelihood" implies a probability that the claim is true in the Bayesian sense. The supporting statistic is P+, the fraction of paired simulations showing a positive effect, derived from a frequentist sampling distribution (the clustered variance-covariance matrix of the estimator) combined with a 10-member GCM ensemble. P+ corresponds approximately to (1 − one-sided p-value) for the null hypothesis of no positive effect.

For the continent-wide claim, P+ = 0.59 corresponds to a point estimate of 0.07 p.p. with a 95% CI of (-0.41, 0.60), clearly not statistically significant. For the southern Africa claim, P+ = 0.91 corresponds to a point estimate of 0.60 p.p. with a 95% CI of (-0.24, 1.61); the one-sided p-value is approximately 0.09, which also does not meet conventional significance thresholds.

Passage 2, Discussion:
"We find a 59% likelihood that anthropogenic climate change since 1901 has increased malaria burden; on average across Africa, an estimated 74 excess malaria cases per 100,000 people can be attributed to historical human-caused climate change."

The same issue with "likelihood" applies. Additionally, the 74 excess cases figure appears without its 95% CI of (-411, 599), which is roughly 14x wider than the point estimate and centered barely above zero. A reader might interpret "74 excess cases" as a clear effect, while in fact the underlying estimate is not statistically distinguishable from zero.

Passage 3, Discussion:
"However, anthropogenic climate change has probably increased the burden of malaria in sub-Saharan Africa, and at high elevations and latitudes, will continue to for several more decades."

Most readers will hear "probably" as substantially stronger than a central estimate of 0.07 p.p. with a 95% CI of (-0.41, 0.60), and as implying a Bayesian probability claim that the underlying frequentist analysis does not support.

I want to be clear that I am not asking the authors to suppress these results. Non-significant findings should be reported, and the regional claims for southern Africa and the east African highlands are genuinely better supported than the continental aggregate. My concern is purely about phrasing. I would recommend:

(i) not reporting P+ as a "likelihood." Instead, describe its meaning directly, e.g., "59% of simulations showed an increase." In the quoted passages, another option would be to report central estimates with confidence intervals directly, in place of P+.

(ii) whenever point estimates are given, confidence intervals should also be reported, particularly for the 74 excess cases figure.

(iii) avoiding "probably" and similar adverbs when reporting frequentist results (particularly when they are not statistically significant) as these imply an unwarranted Bayesian interpretation.

These changes would not weaken the paper. The temperature response function and the regional findings remain compelling, and the honest framing would, if anything, strengthen the study's credibility.

Thank you for raising these important points. Throughout the manuscript, we have ensured that we use likelihood definitions consistent with the Intergovernmental Panel on Climate Change (IPCC) guidance for terminology based on probability distributions (rather than frequentist statistical significance). These definitions are used in both the 5th and 6th Assessment reports and are stated (as of AR6) as:

“Each finding is grounded in an evaluation of underlying evidence and agreement. A level of confidence is expressed using five qualifiers: very low, low, medium, high and very high, and typeset in italics, for example, medium confidence. The following terms have been used to indicate the assessed likelihood of an outcome or result: virtually certain 99–100% probability; very likely 90–100%; likely 66–100%; about as likely as not 33–66%; unlikely 0–33%; very unlikely 0–10%; and exceptionally unlikely 0–1%. Additional terms (extremely likely 95–100%; more likely than not >50–100%; and extremely unlikely 0–5%) are also used when appropriate.” (IPCC 6th Assessment Report, 2021).

In addition, we now ensure that all point estimates are also accompanied by confidence intervals, including when they are repeated in the Discussion, as pointed out by the reviewer. (Previously, we had not repeated confidence intervals multiple times in the text).

In sum, we have done a final pass to (1) add confidence intervals to the Discussion, (2) replace mentions of “likelihood” *per se* with “probability” to avoid any confusion, and (3) ensure language describing confidence aligns with the IPCC definitions, which is relevant in only three locations:

1. In the abstract (passage 1), we state that "We estimate that rising temperatures have likely increased malaria in east and southern Africa" consistent with a probability well above 66% in both cases. We otherwise show confidence intervals for point estimates (including the 74 cases per 100,000 which for simplicity we now report as 1 per 1,000).
2. The first sentence of "Historical impacts of climate change" is correct as-is: "We find that anthropogenic climate change has, more likely than not, been responsible for a small increase in the average prevalence of childhood malaria across sub-Saharan Africa since 1901."
3. The reviewer's passage 3 has been rewritten to align with the same IPCC language: "However, it is more likely than not that anthropogenic climate change has increased the burden of childhood malaria in sub-Saharan Africa, and at high elevations and latitudes, we predict this trend will continue to for several more decades."

This should address all concerns throughout.

References

IPCC, 2021: *Climate Change 2021: The Physical Science Basis.* Contribution of Working Group I to the Sixth Assessment Report. Cambridge University Press, Cambridge, UK and New York, NY, USA. Chapter 1, Box 1.1. doi:10.1017/9781009157896.
